# Long-Time Stability of a Stably Stratified Rest State in the Inviscid 2D Boussinesq Equation

**DOI:** 10.1007/s00205-026-02166-8

**Published:** 2026-04-06

**Authors:** Catalina Jurja, Klaus Widmayer

**Affiliations:** 1 Institute of Mathematics, University of Zurich, Zurich, Switzerland; 2 Institute of Mathematics, University of Zurich, Zurich, Switzerland; 3 Faculty of Mathematics, Institute of Mathematics, University of Vienna, Vienna, Austria

**Keywords:** 35Q35, 35Q86, 35B35, 76B55, 76B15, 76E20

## Abstract

We establish the nonlinear stability on a timescale $O(\varepsilon ^{-2})$ of a linearly, stably stratified rest state in the inviscid Boussinesq system on $\mathbb {R}^2$. Here, $\varepsilon >0$ denotes the size of an initially sufficiently small, Sobolev regular and localized perturbation. A similar statement also holds for the related dispersive SQG equation.

At the core of this result is a dispersive effect due to anisotropic internal gravity waves. At the linearized level, this gives rise to amplitude decay at a rate of $t^{-1/2}$, as observed in Elgindi and Widmayer (SIAM J. Math. Anal. 47(6):4672–4684, 2015). We establish a refined version of this, and propagate nonlinear control via a detailed analysis of nonlinear interactions using the method of partial symmetries developed in Guo et al. (Invent. Math. 231(1):169–262, 2023).

## Introduction

The main focus of this work is the study of stability of certain steady states of the 2D inviscid Boussinesq system

1.1 $$\begin{aligned} \left\{ \begin{aligned}&\partial _t v +v\cdot \nabla v=-\nabla p -\varrho \vec {e_2},\\&\partial _t\varrho +v\cdot \nabla \varrho =0, \\&\textrm{div}v=0, \end{aligned} \right. \end{aligned}$$

which models the dynamics of an incompressible fluid $v:\mathbb {R}^+\times \mathbb {R}^2\rightarrow \mathbb {R}^2$ with pressure $p:\mathbb {R}^+\times \mathbb {R}^2\rightarrow \mathbb {R}$ and scalar density $\varrho :\mathbb {R}^+\times \mathbb {R}^2\rightarrow \mathbb {R}$ under the influence of gravity. This is a widely used simplified model for geophysical flow: the system (1.1) arises from the *Boussinesq approximation* of the inhomogeneous Euler system (see [50, §2.4]), in which the variation of density is assumed to be small compared to the effects of gravity (described by the buoyant force term $-\varrho \vec {e}_2$).

Due to parallels with the 3D axisymmetric Euler equations (see e.g. [43, §5.4.1], [7, 19, 20]), the system (1.1) has seen a lot of attention in recent years: while local well-posedness and blow-up criteria of Beale-Kato-Majda type for initial data in $H^s$, $s>2$, have been shown via classical methods e.g. in [12], the long-time dynamics of solutions to this system are in general not understood, and may include rapid growth or even blow-up scenarios (see e.g. [5, 42] and [8–10, 20, 21]). In view of this, the study of stable dynamics is a natural step towards a fuller understanding of the behavior of solutions to (1.1).

In this work, we focus on dynamics near the stratified steady state

1.2 $$\begin{aligned} (v_s,\varrho _s)=(0,-x_2),\qquad p_s(x_1,x_2)=x_2^2/2; \end{aligned}$$

that is, for $v=v_s+u=u,\varrho =\varrho _s+\rho =-x_2+\rho$ we consider solutions to

1.3 $$\begin{aligned} \left\{ \begin{aligned}&\partial _t u +u\cdot \nabla u=-\nabla p -\rho \vec {e_2},\\&\partial _t\rho +u\cdot \nabla \rho =u_2, \\&\textrm{div}u=0. \end{aligned} \right. \end{aligned}$$

The setting of the steady state (1.2) is a prototypical setting of a *stably stratified*^1^ fluid, where the density of the fluid increases in the direction of gravity (i.e. ${\partial _{x_2}}\varrho _s(x)<0$). This is a natural setting for many atmospheric and oceanic flows (under appropriate averaging, see e.g. [18, Ch. III], [50, Ch. II]). In particular, here buoyant forces give rise to internal gravity waves, which act as a restoring mechanism. More precisely, as shown in [22], the linear dynamics in (1.3) are waves with dispersion relation given by the symbol of a Riesz transform, and feature dispersive amplitude decay at a rate of $t^{-1/2}$. Together with a basic blow-up criterion for the energy, this allowed the authors of [22] to show that the time of existence of solutions extends from the trivial local-wellposedness time scale $O(\varepsilon ^{-1})$ to $O(\varepsilon ^{-4/3})$, where $\varepsilon \ll 1$ denotes the size of the initial data (see also [51] for a lower regularity setting).

In this article, we use a refined analysis of nonlinear interactions to show that stability (and thus also existence) of solutions to (1.3) in fact holds on the longer timescale $O(\varepsilon ^{-2})$. As discussed further below, this is the natural timescale of energy estimates given the rate of amplitude decay, and corresponds to that of a cubic nonlinearity. We summarize our main result as follows:

### Theorem 1.1

There exist a norm *Y*, $N_0\in \mathbb {N}$ and an $\varepsilon _0>0$ such that if for some $0<\varepsilon <\varepsilon _0$

$$\begin{aligned}&\left\Vert u_0\right\Vert _{H^{N_0}}+\left\Vert \rho _0\right\Vert _{H^{N_0}}\le \varepsilon ,  &   \left\Vert u_{0}\right\Vert _Y+\left\Vert \rho _0\right\Vert _Y\le \varepsilon , \end{aligned}$$

then there exist $T\gtrsim \varepsilon ^{-2}$ and a unique solution $(u,\rho )\in C([0,T],H^{N_0}(\mathbb {R}^2,\mathbb {R}^2))\times C([0,T],H^{N_0}(\mathbb {R}^2))$ of (1.3) with initial data $(u_0,\rho _0)$. Moreover, for $t\in [0,T]$, this solution remains small in the above norms and decays in amplitude:

$$\begin{aligned} \left\Vert u(t)\right\Vert _{H^{N_0}}+\left\Vert \rho (t)\right\Vert _{H^{N_0}}\lesssim \varepsilon , \quad \left\Vert \nabla u(t)\right\Vert _{L^\infty }+\left\Vert \nabla \rho (t)\right\Vert _{L^\infty }\lesssim t^{-\frac{1}{2}}\varepsilon . \end{aligned}$$

In particular, the corresponding unique solution of (1.1) with initial data $(u_0,-x_2+\rho _0)$ exists on the same timescale.

To the best of our knowledge, this is the longest known timescale of existence for solutions to (1.3). We give a detailed overview of the proof of Theorem 1.1 in Sect. 1.1 below, while a more precise version of our result is stated in Theorem 2.4.

We comment on some points of immediate relevance.

1. (Assumptions on the initial data) Our analysis proceeds in the spirit of quasilinear, dispersive partial differential equations, in particular as developed in the “method of partial symmetries” of [32], and thus relies heavily on the precise structure of nonlinear interactions in (1.3). The norm *Y* in Theorem 1.1 is a sum of norms *B* and *X* defined in (2.22)–(2.23) that capture anisotropic localization and regularity in frequency space, see Sect. 2.3. Moreover, they include enough regularity in terms of a natural scaling vector field of the system (1.3) and ensure the decay of solutions at the linear rate of $t^{-1/2}$, see also the discussion in Sect. 1.1. That a restriction on the class of initial data is necessary for Theorem 1.1 to hold is clear from the work [3], which shows that there exist $L^\infty$-small initial data producing $L^\infty$-norm inflation of $\partial _x\rho$ in arbitrarily short time.
2. (Dispersive structure) The linearization of (1.3) is an anisotropic dispersive system. In [22] it is shown that its dispersion relation is given by $\pm i\Lambda (\xi ):=\pm i\xi _1\left\lvert \xi \right\rvert ^{-1}$, $\xi \in \mathbb {R}^2$. This is degenerate and leads to the sharp decay rate $t^{-\frac{1}{2}}$, see [22], which together with the energy estimates is the key limiting factor for the timescale in our result. In fact, invoking the standard blow-up criterion shows that $L^2$-based energies can only be expected to remain small on a timescale $O(\varepsilon ^{-2})$, whereas our other nonlinear arguments (to bound the norms *B*, *X*) could go slightly beyond this timescale. We remark that anisotropy does not necessarily lead to degeneracy, as witnessed in another classical geophysical model: the $\beta$-plane equations, a tangent plane model for Eulerian flows on the surface of a rotating 2D sphere. Thereby, rotation gives rise to linear waves with an anisotropic dispersion relation $i\xi _1\left\lvert \xi \right\rvert ^{-2}$, which however leads to decay at the full rate $t^{-1}$. Thanks to the presence of strong cancellations in the nonlinearity (via a “double null structure”), stability was shown to hold globally in time in this model, see [23, 45].
3. (Good unknowns) The system nature of (1.3) poses a challenge, and in particular the fluid variables $u,\rho$ are not convenient from a perturbative point of view. Noting that due to incompressibility, the system (1.3) has only two degrees of freedom, we will instead work with two scalar unknowns $Z_\pm$, which diagonalize the linearized evolution. Through a suitable choice the crucial energy structure, symmetry properties and a certain “null structure” of the equations can be preserved (see the discussion in Sect. 1.1).
4. (Strong stratification) There are many parallels between the effect of constant rotation in homogeneous three-dimensional fluids and that of linear, stable stratification with constant gravity in two- or three-dimensional inhomogeneous fluids. In particular, the dispersion relations in all these cases are zero-homogeneous, anisotropic and degenerate. Moreover, similarly as one can investigate the effect of a fast speed of rotation on existence timescales (see e.g. [6, §5] for the 3D Navier-Stokes, or [48] for the 3D Euler equations), one can also track the strength of the stratification-gravity coupling. This is relevant for (more) steeply stratified versions $(v_s,\varrho _s)=(0,-\alpha x_2)$, $\alpha >0$, of the steady state (1.2), or when quantifying gravity through a constant $g>0$ in the buoyant force term $-g\varrho \vec {e}_2$ in the momentum equation of (1.1). Taking for simplicity $\alpha =g$, hereby $\alpha ^{-1}$ plays the role of a small parameter that can be used to prolong existence times. In close analogy to the aforementioned references, this has been carried out in the context of (1.3) in [52] (see also [49, 53] for the 3D setting): given initial data $(u_0,\rho _0)$ and a time $T>0$, the authors use Strichartz estimates to derive a lower bound for $\alpha$ that guarantees the existence of solutions until at least time *T*. Via the time-scaling symmetry^2^ of (1.3), for initial data of size $\varepsilon$ this agrees with the $O(\varepsilon ^{-4/3})$ timescale of [22, 51], albeit in lower regularity $H^s$, $s>3$. Here, our result should allow to quantitatively improve these arguments, but we do not pursue this here.
5. (Related results) Natural interest also concerns other steady states of (1.1), in particular those including a shearing motion transversal to the direction of gravity and general gravity profiles (i.e. steady states of the form $v_s=f(x_2)\vec {e}_1,\varrho _s=g(x_2)\vec {e}_2$), as well as other domain geometries. However, in general not even linearized dynamics are fully understood. The prototypical example in this context is the “stably stratified Couette flow”, a steady state of (1.1) with fluid velocity $v_s=x_2\vec {e}_1$ and stable stratification profile $\varrho _s=-x_2$. Here, linearized dynamics can be understood explicitly. In the case of a channel domain $\mathbb {T}\times \mathbb {R}$, the background shear flow plays a dominant, strongly stabilizing role via inviscid damping, a classical mixing mechanism. As demonstrated in [2], this guarantees the nonlinear stability of stably stratified Couette flow on a timescale $O(\varepsilon ^{-2})$, provided the initial perturbations are of size $\varepsilon$ and Gevrey regular. Contrary to our setting without background flow, thereby the oscillatory effects of buoyant forces do not stabilize perturbations and instead lead to a slow growth, suggesting that the aforementioned timescale is optimal for the result in [2]. This is also related to echo chains in the linearized equations, see [55, 56]. (It is only in the setting of a 3D channel $\mathbb {T}\times \mathbb {R}\times \mathbb {T}$ that the dispersive effects of internal gravity waves have been shown to improve stability of the stably stratified Couette flow, albeit in the presence of viscosity [13].) For the analogue of (1.2) on $\mathbb {R}^3$, a related dispersive structure has been uncovered in [53] and used to establish a layered 2D Euler dynamic in the singular limit of strong gravity (see also [49]), but stability beyond the basic $O(\varepsilon ^{-1})$ timescale remains an open problem.
6. (Open question) The behavior of solutions beyond the “cubic” time scale $T\gtrsim \varepsilon ^{-2}$ as given by Theorem 1.1 remains a challenging open problem (as mentioned above, $\varepsilon ^{-2}$ is the optimal time scale for bounded energy solutions given the sharp amplitude decay at rate $t^{-1/2}$ and the standard blow-up criterion). While it may seem natural to conjecture that solutions will eventually leave the perturbative realm described here, we are not able to give a concrete description of how this would happen. Similar questions have also been raised and are open for many classical dispersive equations, see e.g. [1, 17, 34, 38–40, 54]. In particular, it may be that solutions become singular after finite time as e.g. in [44], or global regularity may follow from a refined energy estimate relying on new insights into the nonlinear structure as e.g. in [15, 16].

In fact, our arguments also apply to a simpler, closely related setting, namely that of the dispersive surface quasi-geostrophic (SQG) equation

1.4 $$\begin{aligned} {\left\{ \begin{array}{ll} \partial _t \theta +u \cdot \nabla \theta = R_1\theta , \\ u=\nabla ^\perp (-\Delta )^{-1/2}\theta , \\ \theta (0,x)=\theta _0(x), \end{array}\right. } \end{aligned}$$

where $\theta :\mathbb {R}_+ \times \mathbb {R}^2\rightarrow \mathbb {R}$ is the temperature of the fluid and $\widehat{R_1f}(\xi ):=-i{\xi _1}{\left\lvert \xi \right\rvert ^{-1}}\widehat{f}$ is the Riesz transform in the first coordinate. This model has been suggested for certain wave turbulence interactions [47], and adds to the classical inviscid SQG equation the linear right hand side term $R_1\theta$, which has exactly the same dispersive structure as the Boussinesq system. Due to other structural parallels with the 3D Euler equations (in particular a “vortex stretching” dynamic of $\nabla ^\perp \theta$, see e.g. [11]), the dynamics of the inviscid SQG equation are of natural interest, but only understood in few cases (see e.g. [4, 4, 33, 41] and references therein). Even in the dispersive version (1.4), the long-time behavior of initially small solutions remains to be understood. However, close parallels between (1.4) and (1.3) (in terms of both the dispersive and energy structure) have already been exploited in [22] to show that the basic existence timescale of solutions extends to $O(\varepsilon ^{-4/3})$.

The structural features used to establish Theorem 1.1 also include the setting of (1.4) – more precisely, $\theta$ can be viewed as analogous to one of the Boussinesq unknowns $Z_\pm$, with the additional simplification of having only one single nonlinearity (with a similar null structure). Thus, we can extend the time of existence to the timescale $O(\varepsilon ^{-2})$:

### Theorem 1.2

With *Y*, $N_0\in \mathbb {N}$ and $\varepsilon _0>0$ as in Theorem 1.1, it holds that if, for some $0<\varepsilon <\varepsilon _0$,

$$\begin{aligned}&\left\Vert \theta _0\right\Vert _{H^{N_0}}+\left\Vert \theta _0\right\Vert _Y\le \varepsilon , \end{aligned}$$

then there exists a unique solution $\theta \in C([0,T],H^{N_0}(\mathbb {R}^2))$ of (1.4) with $T\gtrsim \varepsilon ^{-2}$. Moreover, for $t\in ]0,T]$ this solution remains small in the above norms and decays in amplitude:

$$\begin{aligned} \left\Vert \theta (t)\right\Vert _{H^{N_0}}\lesssim \varepsilon , \qquad \left\Vert \nabla \theta (t)\right\Vert _{L^\infty }\lesssim t^{-\frac{1}{2}}\varepsilon . \end{aligned}$$

### Outline of the Proof

In what follows, we give an overview of the proof of Theorem 1.1 (and consequently also of Theorem 1.2), highlighting the key features of our approach while referring to the later sections containing the full mathematical details.

Our proof relies on and adapts the method of partial symmetries, as developed in [32] (see also [46]), to the present 2D setting. This in turn builds on a long history of ideas and techniques used in the study of the long-time behaviour of quasilinear dispersive equations with small initial data, in particular as they originate in the method of space-time resonances [28, 30] and many important further developments, e.g. [14, 16, 26, 27, 29, 31, 35–37, 45], an adequate discussion of which goes beyond the scope of this article.

### Structure of the equations

We discuss first the features inherent to the system (1.3) and the equation (1.4) that lay the foundation for our approach.

*Dispersive structure.* To start with, we recall from [22] that (1.3) and (1.4) exhibit dispersion at the linearized level, the dispersion relation $\pm \Lambda$ of which is the symbol of the Riesz transform $R_1$, i.e.

$$\begin{aligned} \Lambda (\xi ) = \frac{\xi _{1}}{\left\lvert \xi \right\rvert }, \hspace{1.5cm} \xi \in \mathbb {R}^2. \end{aligned}$$

For the dispersive SQG equation the dispersive operator is directly apparent through the Riesz transform on the right-hand side of (1.4), for the Boussinesq system this requires a short computation. We note that $\Lambda$ is zero-homogeneous, anisotropic and degenerate, in the sense that ${\det }\textrm{Hess}\Lambda (\xi )=-{\xi _2^2}{\left\lvert \xi \right\rvert ^{-6}}$ vanishes along $\{\xi _2=0\}$, which also leads to the comparatively slow dispersive decay rate $t^{-1/2}$.

In order to facilitate a proper nonlinear analysis also in the Boussinesq system, it is useful to choose suitable dispersive unknowns $Z_\pm$ (see Sect. 2.1). These diagonalize the linearized equation (see Proposition 2.1), and are moreover chosen such that energy balances remain intact, e.g.

$$\begin{aligned} \left\Vert u\right\Vert _{L^2}^2+\left\Vert \rho \right\Vert _{L^2}^2=\frac{1}{2}\left\Vert Z_+\right\Vert _{L^2}^2+\frac{1}{2}\left\Vert Z_-\right\Vert _{L^2}^2. \end{aligned}$$

The nonlinear equations (1.3) can then be recast as

1.5 $$\begin{aligned}&\partial _t Z_\pm + \mathcal {N}_\pm (Z_+,Z_-)=\pm R_1Z_\pm , \end{aligned}$$

where $\mathcal {N}_\pm (Z_+,Z_-)$ are quadratically nonlinear terms. (This is naturally already the form of the dispersive SQG equation (1.4).)

*Scaling symmetry and vector fields.* In addition to a time scaling symmetry, the systems (1.3) and (1.4) have the following spatial scaling symmetry: if $(u,\rho )$ solves (1.3) (resp. $\theta$ solves (1.4)), then so do $(\lambda u(t,\lambda ^{-1}x),\lambda \rho (t,\lambda ^{-1}x))$ (resp. $\lambda \theta (t,\lambda ^{-1}x)$) for $\lambda >0$ (see Sect. 2.2). In our approach, we take advantage of the natural derivative *S* arising from this scaling symmetry,

1.6 $$\begin{aligned} Sf(x)=x\cdot \nabla _xf(x). \end{aligned}$$

The vector field *S* commutes in a favourable way with the equations, allowing us to propagate “regularity” in terms of many of copies *S*, in particular in the form of $L^2$-energies (see Sects. 4.1, 4.2). However, due to the anisotropy, *S* is the only such natural derivative.

To span the full tangent space at any $x\in \mathbb {R}^2$, we complement *S*, a radial derivative in polar coordinates, with another vector field *W*, which in polar coordinates corresponds to an angular derivative, see (2.15). This vector field however, *does not* commute with the equations. As a result, one of the main difficulties of the article is to control sufficient regularity in this angular direction, i.e. to propagate certain bounds along *W*, as they are captured in the *X*-norm discussed below.

*Null structure of the nonlinearity.* A key ingredient that allows us to control the nonlinear interactions is the presence of a null structure. Concretely, the symbols of the quadratic nonlinearities vanish (in a quantifiable fashion) for frequency configurations for which the dispersion of the output and that of the inputs is degenerate. More precisely, all Fourier symbols of the various quadratic nonlinearities contain a factor $\zeta _2\left\lvert \zeta \right\rvert ^{-1}$ for some $\zeta \in \{\xi ,\xi -\eta ,\eta \}$ (see Lemma 5.5), which in turn is related to the degeneracy of the dispersion $\Lambda$. This can be seen directly in the case of the SQG nonlinearity, and follows with a short computation also for the Boussinesq system – see (2.12) and (2.6). This null structure derives from the skew structure of 2D Eulerian nonlinearities of the type $u\cdot \nabla \omega$ with $u=\nabla ^\perp \Delta ^{-\alpha }\omega$ and $\alpha >0$. In our case, it is relatively weak as it leads to cancellations of nonlinear interactions only if all input and output frequencies are located in a degenerate region.

### Setup of the proof

By considering the Duhamel formulation of equations of the form (1.5) and filtering out the linear evolution, it suffices to study bilinear terms of the form

1.7 $$\begin{aligned} \widehat{\mathcal {B}_\mathfrak {m}(f,g)}(t,\xi )=\int _0^t\int _{\mathbb {R}^2}e^{is\Phi (\xi ,\eta )}\mathfrak {m}(\xi ,\eta )\widehat{f}(\xi -\eta )\widehat{g}(\eta )d\eta ds, \end{aligned}$$

where $\Phi =\pm \Lambda (\xi )\pm \Lambda (\xi -\eta )\pm \Lambda (\eta )$ is a phase function, $\mathfrak {m}$ a Fourier multiplier that encodes the nonlinearity and $f,\,g$ are either the profiles $\mathcal {Z}_\pm :=e^{\pm i t\Lambda }Z_\pm$ of the dispersive unknowns for the Boussinesq system or the profile $\Theta :=e^{it\Lambda }\theta$ in the setting of the SQG equation – see Sect. 2.1.

We then prove Theorems 1.1 and 1.2 via a bootstrap argument involving a hierarchy of energy estimates with many ($N_0\gg 1$) derivatives and vector fields *S* (of order $M\ll N_0$), and *B*- and *X*-norms of aforementioned profiles with fewer derivatives and vector fields (of order $N\ll M$) – see Proposition 2.7.

**Localizations.** Our norms quantify localization and regularity, and are $L^2$-based with suitable weights in terms of frequency localization parameters – see Sect. 2.3. On one hand, in addition to the standard Littlewood-Paley projectors $P_k$ for the size $\left\lvert \xi \right\rvert$ of a frequency $\xi \in \mathbb {R}^2$, we quantify the vertical components $\xi _2\left\lvert \xi \right\rvert ^{-1}$ of the interacting frequencies through Littlewood-Paley projections $P_{k,p}$, $k\in \mathbb {Z},\; p\in \mathbb {Z}^-$. We highlight that these quantify exactly the degree of degeneracy of the dispersion relations $\Lambda$, as well as the aforementioned null structure. On the other hand, we introduce an *angular Littlewood-Paley decomposition* $R_l, \; l\in \mathbb {Z}^+$, to capture the angular regularity along *W*. In particular, we show in Proposition 2.3 that there holds $\left\Vert WR_lf\right\Vert _{L^2}\simeq 2^l\left\Vert R_lf\right\Vert _{L^2}$. This approach parallels the setup introduced in [32], and enables us to control and propagate fractional powers in the angular direction – see below.

**Choice of norms.** We define in (2.22), (2.23) the *B*- and *X*-norms for a function $f:\mathbb {R}^2\rightarrow \mathbb {R}$ as

$$\begin{aligned} \left\| f\right\| _{B}&=\sup _{k\in \mathbb {Z},\, p\in \mathbb {Z}^-} 2^{4k^+}2^{-\frac{k^-}{2}}2^{-\frac{p}{2}}\left\| P_{k,p}f\right\| _{L^2}, \\\left\| f\right\| _{X}&=\sup _{\begin{array}{c} k\in \mathbb {Z},\, l\in \mathbb {Z}^+\\ p \in \mathbb {Z}^-, l+p\ge 0 \end{array}}\hspace{-0.3cm}2^{4k^+}2^{(1+\beta )l}2^{(\frac{1}{2}+\beta ) p}\left\| P_{k,p}R_lf\right\| _{L^2}, \end{aligned}$$

where $k^-=\min \{k,0\}$ and $k^+=\max \{k,0\}$. The *B*-norm weighs the parameters $p\in \mathbb {Z}^-$ negatively and scales like the Fourier transform in $L^\infty$, whereas the *X*-norm weighs the parameter *p* positively and gives control of $(1+\beta )$-derivatives in *W* (expressed in terms of the angular localization parameter *l*). While propagating higher powers of *W* nonlinearly is more difficult, it is also clear that a certain minimal power is needed in order to have a chance to obtain optimal decay estimates: In particular, we note that slightly more than one order of *W* is needed in order to ensure control of the Fourier transform in $L^\infty$, see Lemma 3.1.

**Linear decay and choice of norms.** A first key step of our proof is a refined *linear* decay estimate for the semigroup $e^{\pm it\Lambda }$ in terms of our norms, see Proposition 3.2. In general, it is known that the sharp $L^\infty$ decay rate is $t^{-1/2}$ (see [22], reflecting the degeneracy of the dispersion), and we capture this as

$$\begin{aligned} \Vert P_ke^{\pm it\Lambda }f\Vert _{L^\infty }\lesssim t^{-\frac{1}{2}}\sup _{0\le n\le 2}{(\left\Vert S^nf\right\Vert _B +\left\Vert S^nf\right\Vert _X)}, \end{aligned}$$

where the vector field *S* is defined in (1.6). However, here it is important to track more detailed information that in particular allows us to obtain *faster decay away from the degeneracy* of $\Lambda$. More precisely, in Proposition 3.2 we split the action of the semigroup in two components: one corresponds to high angular frequencies and decays *in* $L^2$, whereas the other gives an $L^\infty$ decay, both quantified in terms of time and the parameter *p* relating to the degeneracy. In particular, for $p>-10$ we obtain the almost full $t^{-1}$ decay rate in $L^\infty$, while for small *p* this degenerates to scale at worst as $t^{-1/2}$.

**Energy estimates.** In our bootstrap setting, the $L^\infty$ decay of solutions can be directly used to establish $H^{N_0}$ energy estimates as well as $L^2$ estimates for many vector fields $S^n$, $n\le M$, applied to a solution $(u,\rho )$ of (1.3) (or solution $\theta$ to (1.4), respectively), see Sect. 4. The proof is standard for the $H^{N_0}$ energies, and proceeds through an inductive argument building on the commutator rule $[S,\nabla ]=-\nabla$ for the vector fields (see (4.2) for an iterated version). The corresponding blow-up criteria show that these energies grow with the exponential of the time integral of amplitudes, which in our bootstrap leads to a growth factor of the form $\exp (\int _0^t \varepsilon (1+s)^{-1/2} ds)$. The natural timescale for this to be uniformly bounded is thus $t\lesssim \varepsilon ^{-2}$ (see also Corollaries 4.3 resp. 4.5).

In what follows, the $H^{N_0}$ energy estimates are used chiefly to obtain the desired bounds for high frequencies (called “simple cases” below), whereas the $S^n$, $n\le M$, energy estimates are a key tool for iterated integration by parts along *S*; for more on this, see below.

**Oscillatory toolbox: integration by parts along** *S* **and normal forms.** To exploit oscillations in the bilinear terms (1.7), we develop a framework for repeated integration by parts along the vector field *S*. To that end, it is important to understand the iterated action of the vector fields $S,\; W$ on the objects involved, and in particular on the multipliers. To systematically treat these, in Sect. 5.2 we introduce a class of symbols that includes the building blocks involved in the multipliers and the phases, and is closed under the action of the vector fields. For this class, in Lemmas 5.3–5.6 we establish bounds (in terms of our localization parameters $k_i,p_i,l_i$, $i=1,2$, corresponding to the variables $\xi -\eta$ and $\eta$ involved in (1.7)) for the iterated action of the vector fields $S,\; W$. As a simple yet important observation, we find a suitable algebraic skew structure (see (5.2)) that shows that whenever there is a “gap" in the localization parameters $p,p_i$, then $S\Phi$ is bounded from below. Moreover, we encounter a rich structure that links lower bounds of $S\Phi$ with smallness of the phase $\Phi$ itself (see Proposition 5.8). Roughly speaking, this implies – in quantifiable terms – that we either have a lower bound for $S\Phi$ and thus iterative integrations by parts, or the phase $\Phi$ is comparatively large – see Sect. 5.3. Assuming a lower bound for the action of *S* on the phases, we collect this information in Lemma 5.10, where we present bounds for iterated integration by parts along *S*. A further version of this is presented in the subsequent Lemma 5.11, and Lemma 5.12 follows along similar lines.

To complement these arguments, we show in Sect. 5.7 how largeness of the phase function $\Phi$ can be taken advantage of via normal forms (i.e. an integration by parts in time), in particular in combination with other restrictions on the frequency configurations (see also Sect. 5.6).

In the context of this framework, with a proper organization of cases (see also Lemma 5.13), the rough overall structure of the proofs of the various bounds on bilinear terms (1.7) can be sketched as follows:

1. *Simple cases:* We observe that for very large or very small frequencies, we obtain the desired bounds via energy estimates and set size bounds.
2. *Gap in p:* Here we can integrate by parts according to Lemma 5.10 to obtain the desired bounds for certain ranges of the localization parameters. In the remaining cases, we can use a balance of the *B*- and *X*-norms, depending on the size of the parameters or else normal forms, accompanied by set size estimates.
3. *No gap:* The refined linear decay estimates allow us to take advantage of the comparability of localization parameters *p*. This already suffices to establish the corresponding *B* norm bounds, but additional arguments are necessary for the *X* norm.

**Improved decay for the time derivative of profiles in** $L^2$**.** The first instance where the aforementioned tools are used is in Sect. 6, where we establish a decay rate of almost $t^{-3/4}$ for the $L^2$ norm of the time derivative of the profiles $F_i\in \{\mathcal {Z}_\pm ,\Theta \}$ (contrast this with the simple direct estimate, which only yields a decay at rate $t^{-1/2}$). The improved decay of the time derivative is particularly useful when employing normal forms in the nonlinear analysis discussed below. To prove this result, we follow the scheme described above, and after dealing with the simple cases we localize the profiles inside the integrals (1.7) as $f_i=R_{l_i}P_{k_i,p_i}S^{b_i}F_i$, $i=1,2$, $b_1+b_2\le N$. In the *gap in p* case, we integrate by parts when feasible. Otherwise, we are in the setting where angular parameters $-l_i$ yield the decay at the cost of parameters $-p,-p_i$. We note that the parameters $p\in \mathbb {Z}^-$ come with a negative sign and to compensate for these “losses” we invoke the null structure of the nonlinearity, see e.g. Lemma 6.1 Case B.1.1. The *no gap* case is easily covered by the refined linear decay from Proposition 3.2, Lemma 3.4.

**Bounds on the** *B***- and** *X***-norm.** Finally, in Sects. 7 and 8, respectively, we control bilinear terms of the form (1.7) in the *B*- and *X*-norms: we show that

$$\begin{aligned} \left\Vert \mathcal {B}_\mathfrak {m}(F_1,F_2)\right\Vert _B\lesssim t^{\frac{1}{6}+\delta }\varepsilon ^2,\qquad \left\Vert \mathcal {B}_\mathfrak {m}(F_1,F_2)\right\Vert _X \lesssim t^{\frac{1}{2}-\delta }\varepsilon ^2, \end{aligned}$$

where $F_i=S^{b_i}\mathcal {Z}_\pm$, or $F_i=S^{b_i}\Theta$, for $\delta \ll 1, b_1+b_2\le N$. (In particular, this shows that with the present arguments the *B*-norm bound itself could be propagated on a time interval of almost order $\varepsilon ^{-6}\gg \varepsilon ^{-2}$, provided suitable improvements for the the energy estimates and the *X*-norm are established.) The proof of theses estimates follows the strategy outlined above, and refines the techniques employed already to establish the improved decay of the time derivative of profiles. In particular, for the *B*-norm bounds the *gap in* *p* cases become more delicate and we complement integrating by parts along vector fields with a normal form transform in certain configurations (in particular when the phase satisfies $\left\lvert \Phi \right\rvert \gtrsim 1$). The latter yields bounds of the form

$$\begin{aligned} \left\| P_{k,p}\mathcal {B}_{\mathfrak {m}}(f_1,f_2)\right\| _{L^2}&\lesssim \left\| P_{k,p}\mathcal {Q}_{\mathfrak {m}\Phi ^{-1}}(f_1,f_2)\right\| _{L^2}+\left\| P_{k,p}\mathcal {B}_{\mathfrak {m}\Phi ^{-1}}(\partial _tf_1,f_2)\right\| _{L^2} \\  &\qquad +\left\| P_{k,p}\mathcal {B}_{\mathfrak {m}\Phi ^{-1}}(f_1,\partial _tf_2)\right\| _{L^2}, \end{aligned}$$

where $f_i=P_{k_i,p_i}R_{l_i}F_i$ are the localized profiles and $\mathcal {Q}$ is a bilinear term of the form (1.7) but without time integral. The two last terms above are handled using the bound on the time derivative described above and are of cubic order. On the other hand, the first term contains one less time parameter and previous arguments suffice to estimate it. The proof of the *X*-norm bounds is yet more delicate, requiring refinements of the aforementioned tools and we refer to the beginning of Sects. 8.1, 8.2 for more details.

### Plan of the Article

In Sect. 2 we introduce the necessary background to proceed with the proof of Theorems 1.1, 1.2. We describe in detail the choice of dispersive unknowns for the Boussinesq system in Sect. 2.1 and present the natural vector fields arising from the scaling symmetry of the equations in Sect. 2.2. Moreover, we introduce the necessary localizations in Sect. 2.3. The detailed statements of the main results are presented in Theorems 2.4, 2.5 and proven in Proposition 2.7 using tools from subsequent sections. The linear decay estimate is presented in Sect. 3. The available energy estimates are discussed in Sect. 4.

The technical tools involving the vector fields and in particular iterated integration by parts along vector fields, set-size estimates and normal forms are presented in Sect. 5. The improved decay of the time derivative of our unknowns is proved in Sect. 6. Estimates on the *B*- and *X*-norms are shown in Sects. 7, 8. Appendix A contains auxiliary results such as the control of the Fourier transform in $L^\infty$ and multiplier bounds.

## Functional Framework and Main Result

In this section, we introduce the basic framework for our arguments and present the main results Theorems 1.1 and 1.2 in more detail. In particular, with a suitable functional framework and through an adequate choice of scalar dispersive unknowns for the Boussinesq system, we will show that the proof of the main results reduces to the study of a bootstrap argument involving certain bilinear expressions, the essential features of which are common to both the Boussinesq and SQG systems.

### Choice of Scalar Unknowns

Consider solutions to the Boussinesq system (1.3) written as a system for the two scalar unknowns of vorticity and density $\omega , \rho :\mathbb {R}^+\times \mathbb {R}^2\rightarrow \mathbb {R}$ as

2.1 $$\begin{aligned} \left\{ \begin{aligned}&\partial _t\omega +u\cdot \nabla \omega =-\partial _{x_1}\rho ,\\&\partial _t\rho +u\cdot \nabla \rho =\partial _{x_1}\Delta ^{-1}\omega , \\&u=\nabla ^\perp \Delta ^{-1}\omega , \end{aligned} \right. \end{aligned}$$

where by convention $\nabla ^\perp =(-\partial _{x_2},\partial _{x_1})$. The following result provides a choice of scalar unknowns that diagonalize the associated linear system:

#### Proposition 2.1

Let $(\omega ,\rho )\in C([0,T],(H^{-1}\cap H^s)\times H^s)$ solve (2.1). Define the dispersive unknowns $Z_\pm$ and their profiles $\mathcal {Z}_\pm$ by

2.2 $$\begin{aligned} Z_{\pm }:=\left\lvert \nabla \right\rvert ^{-1}\omega \pm \rho ,\qquad \mathcal {Z}_\pm :=e^{\pm it\Lambda }Z_\pm , \end{aligned}$$

where the dispersive operator is given by

$$\begin{aligned} \Lambda (\xi ):=\frac{\xi _1}{\left\lvert \xi \right\rvert }. \end{aligned}$$

Then $$\mathcal {Z}_\pm $$ satisfy

2.3 $$\begin{aligned}&\mathcal {Z}_\pm (t)=\mathcal {Z}_\pm (0)+\sum _{\mu \in \{+,-\}}\int _0^t\mathcal {Q}_{\mathfrak {m}^{\mu \mu }_\pm }(\mathcal {Z}_{\mu },\mathcal {Z}_{\mu })(s)ds+\int _0^t\mathcal {Q}_{\mathfrak {m}^{+-}_\pm }(\mathcal {Z}_{+},\mathcal {Z}_{-})(s)ds, \end{aligned}$$

where

2.4 $$\begin{aligned} \mathcal {F}(\mathcal {Q}_{\mathfrak {m}^{\mu \nu }_\pm }(\mathcal {Z}_{\mu },\mathcal {Z}_{\nu }))(s,\xi )=\int _{\mathbb {R}^2} e^{ is\Phi ^{\mu \nu }_\pm (\xi ,\eta )}\mathfrak {m}^{\mu \nu }_\pm (\xi ,\eta ) {\widehat{\mathcal {Z}_{\mu }}}(s,\xi -\eta )\widehat{\mathcal {Z}_{\nu }}(s,\eta )d\eta , \end{aligned}$$

with phase functions

2.5 $$\begin{aligned}&\Phi ^{\mu \nu }_\pm (\xi ,\eta )=\pm \Lambda (\xi )-\mu \Lambda (\xi -\eta )-\nu \Lambda (\eta ), \end{aligned}$$

and multipliers given for $\mu \in \{-,+\}$:

2.6 $$\begin{aligned} \begin{aligned}&\mathfrak {m}_\pm ^{\mu \mu }(\xi ,\eta )=-\frac{1}{8}\frac{\xi (\xi -\eta )^\perp }{\left\lvert \xi \right\rvert \left\lvert \xi -\eta \right\rvert }\Big (\frac{\left\lvert \eta \right\rvert ^2-\left\lvert \xi -\eta \right\rvert ^2}{\left\lvert \eta \right\rvert }\Big )\mp \mu \frac{1}{8}\frac{(\xi -\eta )^\perp \eta }{\left\lvert \xi -\eta \right\rvert \left\lvert \eta \right\rvert }\big (\left\lvert \xi -\eta \right\rvert -\left\lvert \eta \right\rvert \big ),\\  &\mathfrak {m}^{+-}_\pm (\xi ,\eta )=-\frac{1}{4}\frac{\xi (\xi -\eta )^\perp }{\left\lvert \xi \right\rvert \left\lvert \xi -\eta \right\rvert }\Big (\frac{\left\lvert \eta \right\rvert ^2-\left\lvert \xi -\eta \right\rvert ^2}{\left\lvert \eta \right\rvert }\Big )\pm \frac{1}{4}\frac{(\xi -\eta )^\perp \eta }{\left\lvert \xi -\eta \right\rvert \left\lvert \eta \right\rvert }\big (\left\lvert \xi -\eta \right\rvert +\left\lvert \eta \right\rvert \big ). \end{aligned} \end{aligned}$$

Moreover, a direct computation using that

2.7 $$\begin{aligned} u=-\frac{1}{2}\nabla ^\perp \left\lvert \nabla \right\rvert ^{-1}(Z_++Z_-),\quad \rho =\frac{1}{2}(Z_+-Z_-), \end{aligned}$$

shows that this choice of unknowns preserves the energy structure in the sense that

2.8 $$\begin{aligned} \left\Vert u\right\Vert _{\dot{H}^k}^2+\left\Vert \rho \right\Vert _{\dot{H}^k}^2=\frac{1}{2}\left\Vert Z_+\right\Vert _{\dot{H}^k}^2+\frac{1}{2}\left\Vert Z_-\right\Vert _{\dot{H}^k}^2=\frac{1}{2}\left\Vert \mathcal {Z}_+\right\Vert _{\dot{H}^k}^2+\frac{1}{2}\left\Vert \mathcal {Z}_-\right\Vert _{\dot{H}^k}^2,\quad k\in \mathbb {N}_0. \end{aligned}$$

#### Proof

By a direct computation, the system (2.1) is equivalent to

$$\begin{aligned} \begin{aligned}&\partial _tZ_\pm +\frac{1}{4}\left\lvert \nabla \right\rvert ^{-1}\textrm{div}\Big (\nabla ^\perp \left\lvert \nabla \right\rvert ^{-1}(Z_++Z_-)\cdot \left\lvert \nabla \right\rvert (Z_++Z_-)\Big )\\&\hspace{3.2cm}\pm \frac{1}{4}\nabla ^\perp \left\lvert \nabla \right\rvert ^{-1}(Z_++Z_-)\cdot \nabla (Z_+-Z_-)=\pm R_1Z_+. \end{aligned} \end{aligned}$$

This can be rewritten compactly as follows

2.9 $$\begin{aligned} (\partial _t\mp R_1)Z_\pm = \mathcal {N}_{\mathfrak {n}^{++}_\pm }(Z_+,Z_+)+\mathcal {N}_{\mathfrak {n}^{+-}_\pm }(Z_+,Z_-)+\mathcal {N}_{\mathfrak {n}^{-+}_\pm }(Z_-,Z_+)+\mathcal {N}_{\mathfrak {n}^{--}_\pm }(Z_-,Z_-), \end{aligned}$$

where for $$\mu ,\nu \in \{+,-\}$$

$$\begin{aligned} \mathcal {F}(\mathcal {N}_{\mathfrak {n}^{\mu \nu }_\pm }(f,g))(\xi ):=\int _{\mathbb {R}^2} \mathfrak {n}^{\mu \nu }_\pm \hat{f}(\xi -\eta )\hat{g}(\eta )d\eta , \end{aligned}$$

with multipliers

$$\begin{aligned} \begin{aligned}&\mathfrak {n}^{++}_{\pm }=\mathfrak {n}^{-+}_\pm =-\frac{1}{4}\frac{\xi (\xi -\eta )^\perp }{\left\lvert \xi \right\rvert \left\lvert \xi -\eta \right\rvert }\left\lvert \eta \right\rvert \mp \frac{1}{4}\frac{(\xi -\eta )^\perp \eta }{\left\lvert \xi -\eta \right\rvert \left\lvert \eta \right\rvert }\left\lvert \eta \right\rvert ,\\&\mathfrak {n}^{--}_{\pm } = \mathfrak {n}^{+-}_\pm =-\frac{1}{4}\frac{\xi (\xi -\eta )^\perp }{\left\lvert \xi \right\rvert \left\lvert \xi -\eta \right\rvert }\left\lvert \eta \right\rvert \pm \frac{1}{4}\frac{(\xi -\eta )^\perp \eta }{\left\lvert \xi -\eta \right\rvert \left\lvert \eta \right\rvert }\left\lvert \eta \right\rvert . \end{aligned} \end{aligned}$$

Observe that since $\mathcal {F}(R_1f)(\xi )=-i\Lambda (\xi )\widehat{f}$, $\Lambda (\xi )=\frac{\xi _1}{\left\lvert \xi \right\rvert }$, for the profiles $\mathcal {Z}_\pm$ there holds

$$\begin{aligned}&\widehat{\mathcal {Z}_+}=e^{it\Lambda }\widehat{Z_+},  &   \widehat{\mathcal {Z}_-}=e^{-it\Lambda }\widehat{Z_-}, \end{aligned}$$

and by the Duhamel formulation we obtain that

2.10 $$\begin{aligned}&\mathcal {Z}_\pm (t)=\mathcal {Z}_\pm (0)+\sum _{\mu ,\nu \in \{+,-\}}\mathcal {B}_{\mathfrak {n}_\pm ^{\mu \nu }}(\mathcal {Z_\mu },\mathcal {Z_\nu })(t), \end{aligned}$$

with

$$\begin{aligned} \mathcal {F}(\mathcal {B}_{\mathfrak {n}_\pm ^{\mu \nu }}(\mathcal {Z_\mu },\mathcal {Z_\nu }))(t,\xi )&: =\int _0^t\mathcal {F}(\mathcal {Q}_{\mathfrak {n}^{\mu \nu }_\pm }(\mathcal {Z}_{\mu },\mathcal {Z}_{\nu }))(s,\xi )ds, \\\mathcal {F}(\mathcal {Q}_{\mathfrak {n}^{\mu \nu }_\pm }(\mathcal {Z}_{\mu },\mathcal {Z}_{\nu }))(s,\xi )&:=\int _{\mathbb {R}^2} e^{is\Phi _\pm ^{\mu \nu }(\xi ,\eta )}\mathfrak {n}^{\mu \nu }_\pm (\xi ,\eta ) \widehat{\mathcal {Z}_{\mu }}(s,\xi -\eta )\widehat{\mathcal {Z}_{\nu }}(s,\eta )d\eta \end{aligned}$$

and phase functions as in (2.5). To arrive at the further simplified expression in (2.3) we symmetrize and collect terms: Observe that by symmetry of $\mathfrak {n}^{\mu \mu }_\pm$ and $\Phi _{\mu \mu }$ under the change of variables $\eta \leftrightarrow \xi -\eta$ there holds that

$$\begin{aligned} \mathcal {F}(\mathcal {Q}_{\mathfrak {n}^{\mu \mu }_\pm }(\mathcal {Z}_{\mu },\mathcal {Z}_{\mu }))(s,\xi )&=\int _{\mathbb {R}^2} e^{ is\Phi _\pm ^{\mu \mu }(\xi ,\eta )}\mathfrak {n}^{\mu \mu }_\pm (\xi ,\eta ) \widehat{\mathcal {Z}_{\mu }}(s,\xi -\eta )\widehat{\mathcal {Z}_{\mu }}(s,\eta )d\eta \\&=\int _{\mathbb {R}^2} e^{ is\Phi _\pm ^{\mu \mu }(\xi ,\xi -\eta )}\mathfrak {n}^{\mu \mu }_\pm (\xi ,\xi -\eta ) \widehat{\mathcal {Z}_{\mu }}(s,\eta )\widehat{\mathcal {Z}_{\mu }}(s,\xi -\eta )d\eta \\&=\mathcal {F}(\mathcal {Q}_{\mathfrak {m}^{\mu \mu }_\pm }(\mathcal {Z}_{\mu },\mathcal {Z}_{\mu }))(s,\xi ). \end{aligned}$$

On the other hand, with the same change of variables and the symmetry $\Phi ^{+-}_\pm (\xi ,\eta )=\Phi ^{-+}_\pm (\xi ,\xi -\eta )$ we compute that

$$\begin{aligned}&\mathcal {F}({\mathcal {Q}}_{\mathfrak {n}^{+-}_\pm }(\mathcal {Z}_+,\mathcal {Z}_-))(s,\xi )+ \mathcal {F}(\mathcal {Q}_{\mathfrak {n}^{-+}_\pm }(\mathcal {Z}_-,\mathcal {Z}_+))(s,\xi )\\&\quad =\int _{\mathbb {R}^2} e^{is\Phi ^{+-}_\pm (\xi ,\eta )}\mathfrak {n}^{+-}_\pm (\xi ,\eta )\widehat{\mathcal {Z}_+}(s,\xi -\eta )\widehat{\mathcal {Z}_-}(s,\eta )d\eta \\&\qquad +\int _{\mathbb {R}^2} e^{is\Phi ^{-+}_\pm (\xi ,\eta )}\mathfrak {n}^{-+}_\pm (\xi ,\eta )\widehat{\mathcal {Z}_-}(s,\xi -\eta )\widehat{\mathcal {Z}_+}(s,\eta )d\eta \\&\quad =\int _{\mathbb {R}^2} e^{is\Phi ^{+-}_\pm (\xi ,\eta )}\mathfrak {n}^{+-}_\pm (\xi ,\eta )\widehat{\mathcal {Z}_+}(s,\xi -\eta )\widehat{\mathcal {Z}_-}(s,\eta )d\eta \\&\qquad +\int _{\mathbb {R}^2} e^{is\Phi ^{+-}_\pm (\xi ,\eta )}\mathfrak {n}^{-+}_\pm (\xi ,\xi -\eta )\widehat{\mathcal {Z}_-}(s,\eta )\widehat{\mathcal {Z}_+}(s,\xi -\eta )d\eta \\&\quad =\mathcal {F}(\mathcal {Q}_{\mathfrak {m}^{+-}_\pm }(\mathcal {Z}_{+},\mathcal {Z}_{-}))(s,\xi ). \end{aligned}$$

$$\square $$

Similarly we can reformulate the problem for the dispersive SQG equation (1.4). If $\theta (t)$ solves (1.4) and $\Theta (t):=e^{it\Lambda }\theta (t)$ is the associated profile, then

2.11 $$\begin{aligned} \Theta (t)=\Theta (0)+\mathcal {B}_{\mathfrak {m}_0}(\Theta ,\Theta )(t), \end{aligned}$$

where

2.12 $$\begin{aligned} \mathcal {F}\mathcal {B}_{\mathfrak {m}_0}(\Theta ,\Theta )(t,\xi )&=\int _0^t \int _{\mathbb {R}^2}e^{is\Phi _+^{++}(\xi , \eta )}\mathfrak {m}_0(\xi ,\eta )\widehat{\Theta }(\xi -\eta )\widehat{\Theta }(\eta )d\eta ds, \nonumber \\ \mathfrak {m}_0(\xi ,\eta )&:=\frac{1}{2} \frac{(\xi -\eta )\cdot \eta ^\perp }{\left\lvert \xi -\eta \right\rvert \left\lvert \eta \right\rvert }(\left\lvert \xi -\eta \right\rvert -\left\lvert \eta \right\rvert ). \end{aligned}$$

#### Proof of (2.11)–(2.12)

By Duhamel’s formula we have that

$$\begin{aligned} \theta (t)=e^{tR_1}\theta _0+ \int _0^t e^{(t-s)R_1}u\cdot \nabla \theta (s)ds, \end{aligned}$$

and thus

$$\begin{aligned} \widehat{\Theta }(t,\xi )&=\widehat{\Theta }_0(\xi )+\int _0^t \int _{\mathbb {R}^2}e^{is\Phi ^{++}_{+}(\xi , \eta )}\frac{\eta ^\perp \cdot (\xi -\eta )}{\left\lvert \eta \right\rvert }\widehat{\Theta }(s,\eta )\widehat{\Theta }(s,\xi -\eta )d\eta ds, \end{aligned}$$

and the change of variables $\eta \leftrightarrow \xi -\eta$ as above gives the claim. $\square$

### Scaling Symmetry and Vector Fields

In this section we discuss the presence of natural derivatives arising from a scaling symmetry. Observe that the perturbed Boussinesq system (1.3) ((2.1) resp. ) has the following scaling symmetry for $\lambda >0$:

$$\begin{aligned}&u_\lambda (t,x)=\lambda u(t,\lambda ^{-1}x),  &   \rho _\lambda (t,x)=\lambda \rho (t,\lambda ^{-1}x), \\&\omega _\lambda (t,x)=\omega (t,\lambda ^{-1}x),  &   p_\lambda (t,x)=\lambda ^2p(t,\lambda ^{-1}x). \end{aligned}$$

That is, if $(u,\rho )$ solve (1.3) with pressure *p*, then $(u_\lambda ,\rho _\lambda )$ solve (1.3) with pressure $p_\lambda$. Similarly, if $(\omega ,\rho )$ solves (2.1), then so does $(\omega _\lambda ,\rho _\lambda )$. Solutions of the dispersive SQG equation satisfy an analogous scaling: if $\theta$ solves (1.4), then so does $\theta _\lambda (t,x)=\lambda \theta (t, \lambda ^{-1} x)$ for $\lambda >0$. This symmetry group is generated by the vector field $\mathcal {S}$ acting on functions *f* as

2.13 $$\begin{aligned} \mathcal {S}f:= - f+ Sf, \hspace{1.5cm} Sf:=x\cdot \nabla _xf. \end{aligned}$$

In particular, (as can be verified also directly since $S\Lambda =0$) we have that *S* commutes with the linear semigroup of the Boussinesq resp. SQG equations,

2.14 $$\begin{aligned} {[}S,e^{it\Lambda }]=0. \end{aligned}$$

In order to span the full tangent space at each point, we complement the natural vector field *S* with

2.15 $$\begin{aligned} Wf:=x^{\perp }\cdot \nabla _xf. \end{aligned}$$

In polar coordinates $x \mapsto (r\cos \tau ,r\sin \tau )$ these derivatives are given as the radial and angular derivative respectively, $S=r\partial _r,\; W=\partial _\tau .$ This will be useful in the following sections.

Moreover, we observe that the decomposition (2.2) of the Boussinesq unknowns $(u,\rho )$ into dispersive unknowns $Z_\pm$ and profiles $\mathcal {Z}_\pm$ interfaces naturally with the vector fields *S* in $L^2$: By direct computation and using (2.14) we have that

2.16 $$\begin{aligned} \Vert S^ku\Vert _{L^2}^2+\Vert S^k\rho \Vert _{L^2}^2&=\frac{1}{2}\Vert S^kZ_+\Vert _{L^2}^2+\frac{1}{2}\Vert S^kZ_-\Vert _{L^2}^2\nonumber \\&=\frac{1}{2}\Vert S^k\mathcal {Z}_+\Vert _{L^2}^2+\frac{1}{2}\Vert S^k\mathcal {Z}_-\Vert _{L^2}^2,\quad k\in \mathbb {N}. \end{aligned}$$

### Localizations

In this section we introduce localizations in frequency and angle, which will allow us to quantify the nonlinear interactions.

To define the Littlewood-Paley projections, let $\psi \in C^\infty (\mathbb {R},[0,1])$ a radially symmetric bump function with ${{\,\textrm{supp}\,}}\psi \subset [-\frac{8}{5},\frac{8}{5}]$ and $\psi |_{[-\frac{4}{5},\frac{4}{5}]}\equiv 1$. Moreover, we let $\varphi (x):=\psi (x)-\psi (2x)$ and define for $a \in \mathbb {Z}, \; b,\; c \in \mathbb {Z}^-$ and $\Lambda$ as in (2.5)

$$\begin{aligned} \varphi _{a,b}(\zeta )&:=\varphi (2^{-a}\left\lvert \zeta \right\rvert )\varphi (2^{-b}\sqrt{1-\Lambda ^2(\zeta )}), \\\varphi _{a,b,c}(\zeta )&:=\varphi (2^{-a}\left\lvert \zeta \right\rvert )\varphi (2^{-b}\sqrt{1-\Lambda ^2(\zeta )})\varphi (2^{-c}\Lambda (\zeta )). \end{aligned}$$

For $k\in \mathbb {Z}$, $p,q\in \mathbb {Z}^-$ we define the associated Littlewood-Paley projections by

$$\begin{aligned} \mathcal {F}(P_{k,p}f)(\xi )=\varphi _{k,p}(\xi )\widehat{f}(\xi ),  &   \mathcal {F}(P_{k,p,q}f)(\xi )=\varphi _{k,p,q}(\xi )\widehat{f}(\xi ). \end{aligned}$$

In later sections, we will use the localization projections simultaneously for the variables $\xi , \, \xi -\eta$ and $\eta$, and thus introduce the following short-hand notation

2.17 $$\begin{aligned} \chi (\xi ,\eta )&=\varphi _{k,p}(\xi )\varphi _{k_1,p_1}(\xi -\eta )\varphi _{k_2,p_2}(\eta ),\nonumber \\ \widetilde{\chi }(\xi ,\eta )&=\varphi _{k,p,q}(\xi )\varphi _{k_1,p_1,q_1}(\xi -\eta )\varphi _{k_2,p_2,q_2}(\eta ). \end{aligned}$$

#### Remark 2.2

Throughout this paper, we will denote by $\overline{\chi }$ (resp. $\overline{\widetilde{\chi }}, \overline{\varphi }$) a function with similar support properties as $\chi$ (resp. $\widetilde{\chi }, \varphi$). For simplicity of notation we do not distinguish the corresponding localization operators $P_{a,b}$, $P_{a,b,c}$ arising from $\varphi$ or $\overline{\varphi }$.

Next we introduce Littlewood-Paley-type localizations in order to quantify regularity in the polar coordinate angle. To that end, let $f\in L^2$ and consider polar coordinates $x\mapsto (r\cos \tau ,r\sin \tau )$. Then we can expand

2.18 $$\begin{aligned} f(x)=\sum _{n\in \mathbb {Z}}f_n(r)e^{in\tau },\quad f_n(r)=\frac{1}{2\pi }\int _0^{2\pi } f(r\cos \tau ,r\sin \tau )e^{-in\tau }d\tau . \end{aligned}$$

We recall here that by Parseval’s theorem it holds that

2.19 $$\begin{aligned} \left\Vert f\right\Vert _{L^2}^2=2\pi \sum _{n\in \mathbb {Z}}\left\Vert f_n\right\Vert ^2_{L^2(\mathbb {R}^+,rdr)}. \end{aligned}$$

Changing back to Cartesian coordinates in (2.18), for $l\in \mathbb {Z}$ we define angular projections as

$$\begin{aligned} (\bar{R}_{\le l}f)(x)&:=\sum _{n\in \mathbb {Z}} \psi (2^{-l}n) \int _{\mathbb {S}^1} f(\left\lvert x\right\rvert y)e^{-in \arccos {(y\cdot \frac{x}{\left\lvert x\right\rvert })}}d\text {vol}_{\mathbb {S}^1}(y),\\ (\bar{R}_{l}f)(x)&:=\sum _{n\in \mathbb {Z}} \varphi (2^{-l}n) \int _{\mathbb {S}^1} f(\left\lvert x\right\rvert y)e^{-in \arccos {(y\cdot \frac{x}{\left\lvert x\right\rvert })}}d\text {vol}_{\mathbb {S}^1}(y). \end{aligned}$$

#### Proposition 2.3

Let $f\in L^2$, $\bar{R}_{\le l}$ and $\bar{R}_l$ defined as above with $l\in \mathbb {Z}$, and *W* as in (2.15). Then following properties hold:

1. $$f=\sum _{l\ge 0} \bar{R}_lf$$    and    $$\left\Vert f\right\Vert _{L^2}^2 \sim \sum _{l\ge 0}\left\Vert \bar{R}_lf\right\Vert _{L^2}^2$$;
2. The operators $\bar{R}_{\le l}$ and $\bar{R}_l$ are bounded in $L^\ell$ for $1\le \ell \le \infty$;
3. The Bernstein property reads:
  $$\begin{aligned} \left\Vert W \bar{R}_lf\right\Vert _{L^{\ell }} \sim 2^l\left\Vert \bar{R}_lf\right\Vert _{L^{\ell }}. \end{aligned}$$

#### Proof

The first property in (1) follows from (2.18) and the fact that $\sum _{l\ge 0} \varphi (2^{-l}\cdot )$ is a partition of unity. Moreover, with (2.19) and the fact that $\varphi ^2$ has similar support properties as $\varphi$, it holds that

$$\begin{aligned} \left\Vert f\right\Vert _{L^2}^2&=\int _0^\infty \int _0^{2\pi } \left\lvert f(r\cos \tau ,r\sin \tau )\right\rvert ^2d\tau rdr\\&=\int _0^\infty 2\pi \sum _{n\in \mathbb {Z}} \left\lvert f_n(r)\right\rvert ^2rdr\\&=2\pi \int _0^\infty \sum _{n\in \mathbb {Z}}\sum _{l\ge 0} \varphi ^2(2^{-l}n)\left\lvert f_n(r)\right\rvert ^2rdr\\&{\sim } \sum _{l\ge 0} \sum _{n\in \mathbb {Z}}\int _{\mathbb {R}^2}\varphi ^2(2^{-l}n)\left\lvert \int _{\mathbb {S}^1}f(\left\lvert x\right\rvert y)e^{-in\arccos {(y\cdot \frac{x}{\left\lvert x\right\rvert })}}d\text {vol}_{\mathbb {S}^1}(y)\right\rvert ^2dx\\&=\sum _{l\ge 0}\left\Vert \bar{R}_l f\right\Vert _{L^2}^2. \end{aligned}$$

We proceed with the proof of (2) for $\bar{R}_l$ and the result for $R_{\le l }$ follows similarly. We view the operator $\bar{R}_l$ as a singular integral operator with kernel ${K_l}(x,y)=\sum _{n\in \mathbb {Z}}\varphi (2^{-l}n)e^{-in\arccos {(y\cdot x})}$ as follows:

$$\begin{aligned} \bar{R}_lf(x)&=\int _{\mathbb {S}^1}f(\left\lvert x\right\rvert y)\sum _{n\in \mathbb {Z}}\varphi (2^{-l}n)e^{-in\arccos {(y\cdot \frac{x}{\left\lvert x\right\rvert })}}d\text {vol}_{\mathbb {S}^1}(y)\\&=\int _{\mathbb {S}^1}f(\left\lvert x\right\rvert y){K_l}\Big (\frac{x}{\left\lvert x\right\rvert },y\Big )d\text {vol}_{\mathbb {S}^1}(y). \end{aligned}$$

Since $|e^{-in\arccos {(y\cdot \frac{x}{\left\lvert x\right\rvert })}}|=1$ and the telescoping sum present in $K_l$ is bounded, it holds that

$$\begin{aligned} \sup _{x}\Vert {K_l}(x,y)\Vert _{L^1{(\mathbb {S}^1,d\text {vol}(y))}}+\sup _{y}\Vert {K_l}(x,y)\Vert _{L^1{(\mathbb {S}^1,d\text {vol}(x))}}\lesssim 1. \end{aligned}$$

The claim follows then by Young’s inequality for integral operators.

As for the proof of (3) recall that in polar coordinates $W=\partial _\tau$. Using the properties of the Fourier transform and the equivalent polar coordinate representation above we see that

$$\begin{aligned} \Vert W\bar{R}_lf\Vert _{L^\ell _{x}}&=\Vert W\bar{R}_lf\Vert _{L^\ell ( r dr d\tau )}\\&=2\pi \big \Vert {\partial _\tau \sum _{n\in \mathbb {Z}}\varphi (2^{-l}n)\int _{0}^{2\pi }f(r\cos \tau ,r\sin \tau )e^{-in\tau }d\tau }\big \Vert _{L^\ell (\mathbb {R}^+, rdr)}\\&=2\pi \big \Vert {\sum _{n\in \mathbb {Z}}\varphi (2^{-l}n)in\int _0^{2\pi }f(r\cos \tau ,r\sin \tau )e^{-in\tau }d\tau }\big \Vert _{L^\ell }\\&\sim 2^l \Vert \bar{R}_lf\Vert _{L^{\ell }}. \end{aligned}$$

$$\square $$

Throughout the paper we will use polar coordinates in frequency space

2.20 $$\begin{aligned} \xi \mapsto (\rho \cos \tau ,\rho \sin \tau )=(\rho \Lambda , \pm \rho \sqrt{1-\Lambda ^2}), \end{aligned}$$

and without loss of generality we consider the upper hemisphere $(\rho ,\tau ) \in \mathbb {R}_+\times [0,\pi ],$ so that $\xi =(\rho \Lambda ,\rho \sqrt{1-\Lambda ^2})$. Then it holds that

2.21 $$\begin{aligned}&\varphi _{k,p}(\xi )=\varphi _{k,p}(\rho ,\tau )=\varphi (2^{-k}\rho )\varphi (2^{-p}\sqrt{1-\Lambda ^2}),  &   \Lambda (\xi )=\cos \tau . \end{aligned}$$

To understand the interplay of the various projections, we observe that with $\xi =\rho \partial _\rho \xi ,\; \xi ^\perp =-\sqrt{1-\Lambda ^2}\partial _\Lambda \xi$, it holds that

$$\begin{aligned} \left\| [W,P_{k,p}]f\right\| _{L^{\ell }}&=\Big \Vert \varphi (2^{-k}\rho )\sqrt{1-\Lambda ^2}2^{-p}\varphi '(2^{-p}\sqrt{1-\Lambda ^2})\partial _\Lambda (\sqrt{1-\Lambda ^2})f \\  &\qquad +\sqrt{1-\Lambda ^2}\varphi (2^{-k}\rho )\varphi (2^{-p}\sqrt{1-\Lambda ^2})\partial _\Lambda f \\  &\qquad -\sqrt{1-\Lambda ^2}\varphi (2^{-k}\rho )\varphi (2^{-p}\sqrt{1-\Lambda ^2})\partial _\Lambda f\Big \Vert _{L^\ell }\\  &\lesssim 2^{-p}. \end{aligned}$$

In particular,

$$\begin{aligned} \Vert WP_{k,p}\bar{R}_lf\Vert _{L^\ell }&=\Vert [W,P_{k,p}]\bar{R}_lf+P_{k,p}W\bar{R}_lf\Vert _{L^\ell }\lesssim 2^{-p}\Vert \bar{R}_lf\Vert _{L^\ell }+2^l\Vert \bar{R}_lf\Vert _{L^\ell }, \end{aligned}$$

and thus for simultaneous localizations in *k*, *p*, *l* the analogue of the above Bernstein property in 2.3(3) can only hold if $-p\le l$. To automatically take this into account we define the operators

$$R_l^p:={\left\{ \begin{array}{ll} 0, &  p+l<0 \\ \bar{R}_{\le l}, &  p+l=0 \\ \bar{R}_l, &  p+l>0. \end{array}\right. }$$

In the following, we will suppress the superscript *p* and note that these operators satisfy properties analogous to those in Proposition 2.3, so that, in particular,

$$\begin{aligned}&P_kf=\sum _{\begin{array}{c} l\in \mathbb {Z}^+, p \in \mathbb {Z}^- \\ l+p\ge 0 \end{array}}P_{k,p}R_lf,  &   P_kf=\sum _{\begin{array}{c} l\in \mathbb {Z}^+, p \in \mathbb {Z}^-, q\in \mathbb {Z}^- \\ l+p\ge 0 \end{array}}P_{k,p,q}R_lf. \end{aligned}$$

These projections satisfy favorable commutation relations with the vector field *S*:

$$\begin{aligned}&{[}S,P_k]f=-{P}_kf,&[S,P_{k,p}f]=-{P}_{k,p}f, \\  &[S,P_{k,p,q}f]=-{P}_{k,p,q}f,&[S,R_l]f=0. \end{aligned}$$

To see this, we compute that

$$\begin{aligned} \widehat{SP_kf}(\xi )&=(-2-S_\xi )\widehat{P_kf}(\xi )=-2^{-k}\left\lvert \xi \right\rvert \varphi '(2^{-k}\left\lvert \xi \right\rvert )\widehat{f}(\xi )+\widehat{P_kSf}(\xi ), \end{aligned}$$

and upon using that $S\Lambda =0$, the claims for the projections $P_{k,p}$ and $P_{k,p,q}$ also follow. Finally, for the angular projections, the claim follows from the definition of $R_l$ by recalling that in polar coordinates $S=r\partial _r$.

To fix notation, in our analysis we make the following notational conventions for the sizes of relevant quantities in terms of the localization parameters:

$$\begin{aligned}&\left\lvert \xi \right\rvert \sim 2^k,  &   \left\lvert \frac{\xi _2}{\left\lvert \xi \right\rvert }\right\rvert =\sqrt{1-\Lambda ^2(\xi )}\sim 2^p,  &   \left\lvert \frac{\xi _1}{\left\lvert \xi \right\rvert }\right\rvert =\left\lvert \Lambda (\xi )\right\rvert \sim 2^q,\\&\left\lvert \xi -\eta \right\rvert \sim 2^{k_1},  &   \left\lvert \frac{\xi _2-\eta _2}{\left\lvert \xi -\eta \right\rvert }\right\rvert =\sqrt{1-\Lambda ^2(\xi -\eta )}\sim 2^{p_1},  &   \left\lvert \frac{\xi _1-\eta _1}{\left\lvert \xi -\eta \right\rvert }\right\rvert =\left\lvert \Lambda (\xi -\eta )\right\rvert \sim 2^{q_1},\\&\left\lvert \eta \right\rvert \sim 2^{k_2},  &   \left\lvert \frac{\eta _2}{\left\lvert \eta \right\rvert }\right\rvert =\sqrt{1-\Lambda ^2(\eta )}\sim 2^{p_2},  &   \left\lvert \frac{\eta _1}{\left\lvert \eta \right\rvert }\right\rvert =\Lambda (\eta )\sim 2^{q_2}. \end{aligned}$$

### Main Result

For $\beta >0$ to be determined (see also Remark 2.6), we define the following weighted norms using the notation $k^+=\max \{0,k\}$ and $k^-=\min \{0,k\}$:

2.22 $$\begin{aligned}&\left\Vert f\right\Vert _{B}:=\sup _{k\in \mathbb {Z},\, p\in \mathbb {Z}^-} 2^{4k^+}2^{-\frac{k^-}{2}}2^{-\frac{p}{2}}\left\Vert P_{k,p}f\right\Vert _{L^2}, \end{aligned}$$

2.23 $$\begin{aligned}&\left\Vert f\right\Vert _{X}:=\sup _{\begin{array}{c} k\in \mathbb {Z},\, l\in \mathbb {Z}^+,\, p \in \mathbb {Z}^- \\ l+p\ge 0 \end{array}}2^{4k^+}2^{(1+\beta )l}2^{(\frac{1}{2}+\beta ) p}\left\Vert P_{k,p}R_lf\right\Vert _{L^2}. \end{aligned}$$

The *B*-norm captures the anisotropic localizations (with respect to the degeneracy of the phase, via the parameter *p*) and scales like the Fourier transform in $L^{\infty }$, whereas the *X*-norm accounts for a certain amount of angular regularity in *W* (measured through the weight in $2^l$).

In this framework, Theorem 1.1 for the Boussinesq system (1.3) can be stated for the corresponding dispersive unknowns in detail as follows:

#### Theorem 2.4

Let $N>5$. There exist $M, N_0\in \mathbb {N}$, $\beta , \delta >0$ satisfying $N_0\gg M\gg N+\beta ^{-2}$, $\delta \ll \beta$ and an $\varepsilon _0>0$ such that if for some $0<\varepsilon <\varepsilon _0$ we have

2.24 $$\begin{aligned} \begin{aligned} \left\Vert Z_{\pm ,{0}}\right\Vert _{H^{N_0}}+\left\Vert S^aZ_{\pm ,0}\right\Vert _{L^2}&\le \varepsilon ,\hspace{1.5cm} 0\le a \le M, \\ \Vert {S^bZ_{\pm ,{0}}}\Vert _B+\Vert {S^bZ_{\pm ,{0}}}\Vert _X&\le \varepsilon , \hspace{1.5cm} 0\le b\le N, \end{aligned} \end{aligned}$$

then there exist $T\gtrsim \varepsilon ^{-2}$ and a unique solution $(Z_{+},Z_{-} ) \in (C([0,T],H^{N_0}(\mathbb {R}^2))^2$ of (2.9) with initial data $(Z_+(0),Z_-(0))=(Z_{+,{0}},Z_{-,{0}})$, and therefore a unique solution $(u,\rho )\in C([0,T],H^{N_0}(\mathbb {R}^2,\mathbb {R}^2))\times C([0,T],H^{N_0}(\mathbb {R}^2))$ of (1.3) with initial data $(u_0,\rho _0)=\frac{1}{2}(-\nabla ^\perp \left\lvert \nabla \right\rvert ^{-1}(Z_{+,0}+Z_{-,0}), Z_{+,0}-Z_{-,0})$.

Analogously, Theorem 1.2 for the dispersive SQG equation (1.4) is stated in detail as follows:

#### Theorem 2.5

Let $N>5$. There exist $M, N_0\in \mathbb {N}$, $\beta ,\delta >0$ satisfying $N_0\gg M\gg N+\beta ^{-2}$, $\delta \ll \beta$ and an $\varepsilon _0>0$ such that if $\theta _0$ satisfies

2.25 $$\begin{aligned} \begin{aligned} \left\Vert \theta _0\right\Vert _{H^{N_0}}+\left\Vert S^a\theta _0\right\Vert _{L^2}&\le \varepsilon ,\hspace{1.5cm} 0\le a \le M, \\ \Vert {S^b\theta _0}\Vert _B+\Vert {S^b\theta _0}\Vert _X&\le \varepsilon , \hspace{1.5cm} 0\le b\le N \end{aligned} \end{aligned}$$

for some $0<\varepsilon <\varepsilon _0$, then there exist $T\gtrsim \varepsilon ^{-2}$ and a unique solution $\theta \in C([0,T],{H^{N_0}}(\mathbb {R}^2))$ of (1.4).

#### Remark 2.6

1. As part of the proof of Theorem 2.4 (Theorem 2.5 resp.) via the continuity method based on Proposition 2.7 below, the solutions to the corresponding problems remain small of order $\varepsilon$ in the considered norms on the interval [0, *T*] with *T* as in Proposition 2.7:
  $$\begin{aligned} \Vert Z_\pm (t)\Vert _{H^{N_0}}+\sum _{a=0}^M\Vert S^aZ_\pm (t)\Vert _{L^2}+\sum _{b=0}^N\Vert S^b\mathcal {Z}_\pm (t)\Vert _{B}+ \Vert S^b\mathcal {Z}_\pm (t)\Vert _{X}\lesssim \varepsilon , \end{aligned}$$
  resp.
  $$\begin{aligned} \Vert \theta (t)\Vert _{H^{N_0}}+\sum _{a=0}^M\Vert S^a\theta (t)\Vert _{L^2}+\sum _{b=0}^N\Vert S^b\Theta (t)\Vert _{B}+ \Vert S^b\Theta (t)\Vert _{X}\lesssim \varepsilon , \quad 0\le t\le T. \end{aligned}$$
  In particular, for $0<t\le T$ the solutions decay as follows:
  $$\begin{aligned} \Vert S^bZ_\pm (t)\Vert _{L^\infty }\lesssim t^{-\frac{1}{2}}\varepsilon , \quad \text {resp.} \quad \Vert S^b\theta (t)\Vert _{L^\infty }\lesssim t^{-\frac{1}{2}}\varepsilon , \qquad 0\le b\le N-2. \end{aligned}$$
2. We can choose the parameters in the above theorems as $\beta =10^{-2}$, $N_0\sim 10^9$, and $\delta =2M^{-\frac{1}{2}}$, such that $N_0\gg M\gg M^{\frac{1}{2}}\gg \beta ^{-2}$. Moreover, $\delta _0=2N_0^{-1}$ is an useful parameter in subsequent Sects. 6–8. These are convenient choices from a technical point of view (see the proofs of Propositions 7.1, 8.1 and 8.2), but no effort has been made at optimizing them.

Theorems 2.4, 2.5 follow via a continuity argument using the local well-posedness of the Boussinesq system (1.3) (SQG equation (1.4) respectively) and the following proposition (recall that with the scalar unknowns $Z_\pm$ and their respective profiles $\mathcal {Z}_\pm$, the system (1.3) is equivalent to (2.3), and the SQG equation (1.4) for $\theta$ is equivalent to (2.11) for the SQG profile $\Theta$):

#### Proposition 2.7

Let $C>0$, and $T\le C\varepsilon ^{-2}$. Assume $\mathcal {Z}_\pm \in C([0,T],H^{N_0}(\mathbb {R}^2))$ solve (2.3) resp. $\Theta \in C([0,T],H^{N_0}(\mathbb {R}^2))$ solves (2.11) with initial data satisfying (2.24) resp. (2.25). If for $t\in [0,T]$ it holds that

2.26 $$\begin{aligned} \Vert {S^b\mathcal {Z}_\pm (t)}\Vert _B+\Vert {S^b\mathcal {Z}_\pm (t)}\Vert _X&\le 100\varepsilon \quad \text{ resp. }\quad \\\Vert {S^b\Theta (t)}\Vert _B+\Vert {S^b\Theta (t)}\Vert _X\nonumber&\le 100\varepsilon , \quad 0\le b\le N, \end{aligned}$$

then for $F\in \{\mathcal {Z}_+,\mathcal {Z}_-\}$ resp. $F=\Theta$ we have

2.27 $$\begin{aligned} \Vert {F(t)}\Vert _{H^{N_0}}+\sum _{a=0}^M\Vert {S^aF(t)}\Vert _{L^2}\lesssim \varepsilon , \end{aligned}$$

and in fact there holds the improved bound

2.28 $$\begin{aligned} \Vert {S^bF(t)}\Vert _B+\Vert {S^bF(t)}\Vert _X&\le 10\varepsilon . \end{aligned}$$

We outline next the proof of Proposition 2.7 to show how it combines the remaining arguments of the paper.

#### Proof

Without loss of generality, we consider the setting of the Boussinesq system. Under the bootstrap assumption (2.26) and by Corollary 3.3 it holds that

$$\begin{aligned} \Vert S^b{Z}_{\pm }(t)\Vert _{L^\infty }\lesssim t ^{-\frac{1}{2}}\varepsilon , \hspace{1.5cm} 0\le b<N-2. \end{aligned}$$

Together with the initial data assumption this implies the bound (2.27) on the energy as shown in Corollary 4.3, as long as $T\lesssim \varepsilon ^{-2}$. In order to prove (2.28), we note that from the Duhamel formula (2.3) and for $0\le b\le N$ we have

$$\begin{aligned} \Vert {S^b\mathcal {Z}_{\pm }(t)}\Vert _B+\Vert {S^b\mathcal {Z}_{\pm }(t)}\Vert _X&\le \Vert {S^b\mathcal {Z}_{\pm }(0)}\Vert _B+\Vert {S^b\mathcal {Z}_{\pm }(0)}\Vert _X \\  &\quad +\Vert {S^b\mathcal {B}_{\mathfrak {m}_{\pm }^{+-}}(\mathcal {Z}_{+},\mathcal {Z}_{-})}\Vert _B+\Vert {S^b\mathcal {B}_{\mathfrak {m}_{\pm }^{+-}}(\mathcal {Z}_{+},\mathcal {Z}_{-})}\Vert _X \\  &\quad +\sum _{\mu \in \{+,-\}}\Vert {S^b\mathcal {B}_{\mathfrak {m}_{\pm }^{\mu \mu }}(\mathcal {Z}_{\mu },\mathcal {Z}_{\mu })}\Vert _B +\Vert {S^b\mathcal {B}_{\mathfrak {m}_{\pm }^{\mu \mu }}(\mathcal {Z}_{\mu },\mathcal {Z}_{\mu })}\Vert _X. \end{aligned}$$

Therefore, to prove (2.28) it suffices to show that under the bootstrap assumption (2.26) and for $\mathfrak {m}\in \left\{ \mathfrak {m}^{\mu \nu }_{\pm } \mid \mu ,\nu \in \{-,+\} \right\}$ it holds that

$$\begin{aligned} \Vert {S^b\mathcal {B}_{\mathfrak {m}}(\mathcal {Z}_{\mu },\mathcal {Z}_{\nu })}\Vert _B+\Vert {S^b\mathcal {B}_{\mathfrak {m}}(\mathcal {Z}_{\mu },\mathcal {Z}_{\nu })}\Vert _X \le 9\varepsilon ,&\hspace{1.5cm} 0\le b\le N. \end{aligned}$$

Since *S* derives from a symmetry of the equation (see the below Lemma 2.8 for an explicit computation), it suffices to show that for $b_1,\;b_2\ge 0$ with $b_1+b_2\le N$ there holds

2.29 $$\begin{aligned} \Vert {\mathcal {B}_{\mathfrak {m}}(S^{b_1}\mathcal {Z}_{\mu },S^{b_2}\mathcal {Z}_{\nu })}\Vert _B+\Vert {\mathcal {B}_{\mathfrak {m}}(S^{b_1}\mathcal {Z}_{\mu },S^{b_2}\mathcal {Z}_{\nu })}\Vert _X\lesssim 9\varepsilon . \end{aligned}$$

To handle such expressions, we also localize the time variable: for $t\in [0,T]$ we decompose the indicator function $1\!\!1_{[0,t]}$ in functions $\tau _0,...\tau _{L+1}:\mathbb {R}\rightarrow [0,1]$ with $\left\lvert L-\log _2(2+t)\right\rvert \le 2$ such that for $m\in \{1,...,L\}$

$$\begin{aligned}&{{\,\text {supp}\,}}\tau _0\subset [0,2],\hspace{0.5cm} {{\,\text {supp}\,}}\tau _m \subset [2^{m-1},2^{m+1}], \; {{\,\text {supp}\,}}\tau _{L+1}\subset [t-2,t],\\  &\sum _{m=0}^{L+1}\tau _m(s)=1\!\!1_{[0,t]}, \hspace{0.5cm}\tau _m(s)\in C^1(\mathbb {R}), \hspace{0.5cm}\int _0^t\left\lvert \tau _m(s)\right\rvert ds\lesssim 1, \; m\in \{1,..., L\}. \end{aligned}$$

Then for a bilinear expression with multiplier $\mathfrak {m}$ as in (2.6) it holds that

2.30 $$\begin{aligned} \mathcal {B}_{\mathfrak {m}}(f,g)=\int _0^t\mathcal {Q}_{\mathfrak {m}}(f,g)ds=\sum _m\int _0^t\tau _m(s)\mathcal {Q}_{\mathfrak {m}}(f,g)ds=\sum _m\mathcal {B}_{\mathfrak {m}}^m(f,g), \end{aligned}$$

where $\mathcal {B}_{\mathfrak {m}}^m(f,g):=\int _0^t\tau _m(s)\mathcal {Q}_{\mathfrak {m}}(f,g)ds$. Bounds on such time-localized bilinear terms are shown in the subsequent Sects. 7 and 8: In Proposition 7.1 we prove that

$$\begin{aligned} \Vert \mathcal {B}_{\mathfrak {m}}^m(S^{b_1}\mathcal {Z}_{\mu },S^{b_2}\mathcal {Z}_{\nu })\Vert _B\lesssim 2^{(\frac{1}{6}+\delta ) m}\varepsilon ^2, \end{aligned}$$

whereas Propositions 8.1 and 8.2 show that

$$\begin{aligned} \Vert \mathcal {B}_{\mathfrak {m}}^m(S^{b_1}\mathcal {Z}_{\mu },S^{b_2}\mathcal {Z}_{\nu })\Vert _X\lesssim 2^{(\frac{1}{2}-\frac{\delta }{8})m}\varepsilon ^2, \end{aligned}$$

where $\delta =2M^{-\frac{1}{2}}$. Therefore, with $C_1>0$ and $t\in [0,T]$ with $T\le C\varepsilon ^{-2}$ we obtain

$$\begin{aligned} \Vert {S^b\mathcal {B}_{\mathfrak {m}}(\mathcal {Z}_{\mu },\mathcal {Z}_{\nu })}\Vert _B+\Vert {S^b\mathcal {B}_{\mathfrak {m}}(\mathcal {Z}_{\mu },\mathcal {Z}_{\nu })}\Vert _X \le C_1t^{\frac{1}{2}-\frac{\delta }{8}}\varepsilon ^2\le C_1C^{\frac{1}{2}-\frac{\delta }{8}}\varepsilon ^{{\delta ^2}/{16}}\varepsilon . \end{aligned}$$

Choosing $\varepsilon _0>0$ such that $C_1C^{\frac{1}{2}-\frac{\delta }{8}}\varepsilon _0^{{\delta ^2}/{16}}<9$ yields (2.28). $\square$

We conclude this section with a short lemma that records the interplay of the scaling vector field *S* and bilinear terms.

#### Lemma 2.8

Let $N\in \mathbb {N}$, *S* be the vector field defined in (2.13), and $\mathcal {Q}_{\mathfrak {m}}(f,g)$ a bilinear expression as in (2.4), $\mathfrak {m}\in \{\mathfrak {m}_0,\mathfrak {m}_\pm ^{\mu \nu }\}$. Then it holds that

$$\begin{aligned} S^N\mathcal {Q}_{\mathfrak {m}}(f,g)=\sum _{\begin{array}{c} b_1,b_2\in \mathbb {N}_0,\\ 0\le b_1+b_2\le N \end{array}}c_{b_1b_2}\mathcal {Q}_{\mathfrak {m}}(S^{b_1}f,S^{b_2}g), \end{aligned}$$

for universal constants $c_{b_1b_2}\in \mathbb {Z}$.

#### Proof

We begin by observing that $S_\xi \Lambda (\xi )=0$, and since $S_\eta \Lambda (\xi -\eta )=-S_\xi \Lambda (\xi -\eta )$ it follows that $(S_{\xi }+S_\eta )\Phi =0$. Furthermore, by a direct computation we have that $(S_\xi +S_\eta )\mathfrak {m}=0$ for $\mathfrak {m}\in \{\mathfrak {m}_0,\mathfrak {m}_\pm ^{\mu \nu }\}$. Integration by parts in $S_\eta$ then gives

$$\begin{aligned} S_\xi \mathcal {F}{(\mathcal {Q}_{\mathfrak {m}}(f,g))}(\xi )&=\int _{\mathbb {R}^2}e^{it\Phi }(S_\xi +S_\eta )(\mathfrak {m}(\xi ,\eta ))\widehat{f}(\xi -\eta )\widehat{g}(\eta )d\eta \\&\quad +\int _{\mathbb {R}^2}e^{it\Phi }\mathfrak {m}(\xi ,\eta )(S_\xi +S_\eta )\widehat{f}(\xi -\eta )\widehat{g}(\eta )d\eta \\&\quad +\int _{\mathbb {R}^2}e^{it\Phi }\mathfrak {m}(\xi ,\eta )\widehat{f}(\xi -\eta )S_\eta \widehat{g}(\eta )d\eta \\&=\int _{\mathbb {R}^2}e^{it\Phi }\mathfrak {m}(\xi ,\eta )(S\widehat{f})(\xi -\eta )\widehat{g}(\eta )d\eta \\&\quad +\int _{\mathbb {R}^2}e^{it\Phi }\mathfrak {m}(\xi ,\eta )\widehat{f}(\xi -\eta )(S\widehat{g})(\eta )d\eta , \end{aligned}$$

and the claim follows by iteration. $\square$

## Linear Decay

In this section we establish amplitude decay estimates for the semigroup $e^{it\Lambda }$ that build on our choice of norms. In particular, we collect the relevant information in a “decay norm”^3^

3.1 $$\begin{aligned} \left\Vert f\right\Vert _D:=\sup _{0\le n\le 2}{(\left\Vert S^nf\right\Vert _B +\left\Vert S^nf\right\Vert _X)}. \end{aligned}$$

As a basic ingredient, this norm allows us to control the $L^\infty$ norm of the Fourier transform of suitably localized versions of *f*, i.e.

$$\begin{aligned} \Vert \widehat{P_{k,p}f}\Vert _{L^\infty }&\lesssim 2^{-4k^+}2^{-k} \left\Vert f\right\Vert _D. \end{aligned}$$

This can be seen directly from the following lemma:

### Lemma 3.1

For any $f\in L^2$ there holds

$$\begin{aligned} \left\Vert \widehat{P_{k,p}f}\right\Vert _{L^\infty } \lesssim 2^{-4k^+} 2^{-k}\Big [\left\Vert P_kf\right\Vert _B+\left\Vert SP_kf\right\Vert _B+\left\Vert P_kf\right\Vert _X+\left\Vert SP_kf\right\Vert _X \Big ]. \end{aligned}$$

The proof of this statement follows from the fundamental theorem of calculus and is detailed in Appendix A.1.

The following establishes a decomposition of the action of the semigroup $e^{it\Lambda }$ and gives precise decay estimates in relation to the degeneracy of the corresponding linear phase.

### Proposition 3.2

(Linear decay) Let $f:\mathbb {R}^2\rightarrow \mathbb {R}$ and consider the decay norm defined as in (3.1). For $0<\beta ^\prime <\beta$, we can decompose

$$\begin{aligned} P_{k,p}e^{it\Lambda }f= I_{k,p}(f)+II_{k,p}(f) \end{aligned}$$

such that the following bounds hold: for $I_{k,p}$ we have

3.2 $$\begin{aligned}&p\le -10:  &   \left\Vert I_{k,p}(f)\right\Vert _{L^\infty }\lesssim 2^{\frac{3}{4}k}2^{-\frac{15}{4}k^+}\min {\{2^p,2^{-p}\left\lvert t\right\rvert ^{-1}\}}\left\Vert f\right\Vert _D, \nonumber \\&p\ge -10:  &   \left\Vert I_{k,p}(f)\right\Vert _{L^\infty }\lesssim 2^{\frac{3}{4}k}2^{-\frac{15}{4}k^+}\log (\left\lvert t\right\rvert )\left\lvert t\right\rvert ^{-1}\left\Vert f\right\Vert _D, \end{aligned}$$

while the term $II_{k,p}(f)$ satisfies

3.3 $$\begin{aligned} \left\Vert II_{k,p}(f)\right\Vert _{L^2}&\lesssim 2^{-4k^+}2^{-(\frac{1}{2}+2\beta ^\prime )p}\left\lvert t\right\rvert ^{-(\frac{1}{2}+\beta ^\prime )} 1\!\!1_{2^p\gtrsim \left\lvert t\right\rvert ^{-1/2}}\left\Vert f\right\Vert _D. \end{aligned}$$

In particular, since $\left\Vert II_{k,p}(f)\right\Vert _{L^\infty }\lesssim \left\Vert \varphi _{k,p}\right\Vert _{L^2}\left\Vert II_{k,p}(f)\right\Vert _{L^2}$ the $L^\infty$ bound for $II_{k,p}(f)$ is given by

$$\begin{aligned} \left\Vert II_{k,p}(f)\right\Vert _{L^\infty }\lesssim 2^k2^{-4k^+}2^{-2\beta ^\prime p}\left\lvert t\right\rvert ^{-(\frac{1}{2}+\beta ^\prime )} 1\!\!1_{2^p\gtrsim \left\lvert t\right\rvert ^{-1/2}}\left\Vert f\right\Vert _D. \end{aligned}$$

Before we proceed with the proof, we record the following useful corollary, which shows that Proposition 3.2 entails the sharp linear decay rate (see [22, §2.2]).

### Corollary 3.3

For $t>0$, the semigroup $e^{\pm it\Lambda }$ satisfies

$$\begin{aligned} \Vert P_ke^{\pm it\Lambda }f\Vert _{L^\infty }\lesssim 2^{\frac{3}{4}k}2^{-\frac{15}{4}k^+} t^{-\frac{1}{2}}\left\Vert f\right\Vert _D. \end{aligned}$$

In particular, under the bootstrap assumption (2.26), for the Boussinesq system (1.3) there holds

$$\begin{aligned}&\left\Vert \nabla u(t)\right\Vert _{L^\infty }+\left\Vert Su(t)\right\Vert _{L^\infty }+\left\Vert \nabla \rho (t)\right\Vert _{L^\infty }\lesssim t^{-\frac{1}{2}}\varepsilon , \end{aligned}$$

and analogously for the SQG equation (1.4):

$$\begin{aligned}&\left\Vert \nabla \theta (t)\right\Vert _{L^\infty }+\left\Vert u\right\Vert _{L^\infty }+\left\Vert Su(t)\right\Vert _{L^\infty }\lesssim t^{-\frac{1}{2}}\varepsilon . \end{aligned}$$

### Proof

A direct set size estimate (see (3.5) below) shows that

$$\begin{aligned} \left\Vert P_{k,p}e^{\pm it\Lambda }f\right\Vert _{L^\infty }\lesssim 2^{\frac{3}{4}k}2^{-\frac{15}{4}k^+}2^p\left\Vert f\right\Vert _D. \end{aligned}$$

Together with Proposition 3.2 it follows that

$$\begin{aligned} \left\| P_ke^{\pm it\Lambda }f\right\| _{L^\infty }&\le \sum _{p\in \mathbb {Z}^-}\left\| P_{k,p}e^{\pm it\Lambda }f\right\| _{L^\infty }=\sum _{ 2^{p}\lesssim t^{-1/2}}\left\| P_{k,p}e^{\pm it\Lambda }f\right\| _{L^\infty } \\  &\quad +\sum _{ 2^{p}\gtrsim t^{-1/2}}\left\| P_{k,p}e^{\pm it\Lambda }f\right\| _{L^\infty }\\  &\lesssim \sum _{2^{p}\lesssim t^{-1/2}}2^{\frac{3}{4}k}2^{-\frac{15}{4}k^+}2^p\left\| f\right\| _D +\sum _{ 2^{p}\gtrsim t^{-1/2}}(\left\| I_{k,p}\right\| _{L^\infty }+\left\| II_{k,p}\right\| _{L^\infty })\\  &\lesssim 2^{\frac{3}{4}k}2^{-\frac{15}{4}k^+} t^{-\frac{1}{2}}\left\| f\right\| _D+ \sum _{2^{p}\gtrsim t^{-1/2}, p\le -10}\left\| I_{k,p}\right\| _{L^\infty } \\  &\quad +\sum _{2^{p}\gtrsim t^{-1/2}, p\ge -10}\left\| I_{k,p}\right\| _{L^\infty } \\  &\quad +\sum _{ 2^{p}\gtrsim t^{-{1}/{2}}}2^k2^{-4k^+}2^{-2\beta 'p}t^{-(\frac{1}{2}+\beta ')}\left\| f\right\| _D\\  &\lesssim 2^{k}2^{-4k^+} t^{-\frac{1}{2}}\left\| f\right\| _D+\hspace{-0.4cm}\sum _{p\le -10, 2^p\gtrsim t^{-1/2}}\hspace{-0.7cm}2^{\frac{3}{4}k-\frac{15}{4}k^+}\min \{2^p,2^{-p}t^{-1}\}\left\| f\right\| _D\\  &\quad +\sum _{p\ge -10}2^{\frac{3}{4}k-\frac{15}{4}k^+}\log (t)t^{-1}\left\| f\right\| _D\\  &\lesssim 2^{\frac{3}{4}k}2^{-\frac{15}{4}k^+} t ^{-\frac{1}{2}}\left\| f\right\| _D. \end{aligned}$$

As for the SQG equation, recall that $\theta (t)=e^{-it\Lambda }\Theta (t),u(t)=e^{-it\Lambda }\nabla ^\perp (-\Delta )^{-\frac{1}{2}}\Theta (t)=:e^{-it\Lambda }u_\Theta (t)$. Moreover observe that $[S,\nabla ^\perp ]=-\nabla ^\perp$, $[S,\left\lvert \nabla \right\rvert ^{-1}]=-\left\lvert \nabla \right\rvert ^{-1}$ and $\Vert \nabla ^\perp (-\Delta )^{-\frac{1}{2}}g\Vert _{L^2}\lesssim \left\Vert g\right\Vert _{L^2}$. Then it holds that

$$\begin{aligned} \left\| \nabla \theta (t)\right\| _{L^\infty }+\left\| u\right\| _{L^\infty }+\left\| Su(t)\right\| _{L^\infty }&\lesssim \sum _{k\in \mathbb {Z}}\left\| P_k\nabla e^{-it\Lambda } \Theta \right\| _{L^\infty }+\left\| P_ke^{-it\Lambda }u_\Theta \right\| _{L^\infty }\\  &\qquad \quad +\left\| P_ke^{-it\Lambda }Su_\Theta \right\| _{L^\infty }\\  &\lesssim \sum _{k\in \mathbb {Z}} 2^{\frac{3}{4}k}2^{-\frac{15}{4}k^+} t^{-\frac{1}{2}}(2^k\left\| \Theta \right\| _D+ \left\| \Theta \right\| _D+\left\| S\Theta \right\| _D)\\  &\lesssim t^{-\frac{1}{2}}\varepsilon . \end{aligned}$$

The bound for the Boussinesq system follows analogously by recalling the definition of the dispersive unknowns $Z_\pm$ and their respective profiles $\mathcal {Z}_\pm$ in (2.2). Indeed, by (2.7) we have that

$$\begin{aligned} u(t)=-\frac{1}{2}\nabla ^ \perp \left\lvert \nabla \right\rvert ^{-1}(e^{-it\Lambda }\mathcal {Z}_+(t)+e^{it\Lambda }\mathcal {Z}_-(t)),\quad \rho (t)=\frac{1}{2}(e^{-it\Lambda }\mathcal {Z}_+(t)-e^{it\Lambda }\mathcal {Z}_-(t)), \end{aligned}$$

and for

$$\begin{aligned} \tilde{A}(t):= \left\Vert \nabla u(t)\right\Vert _{L^\infty } +\left\Vert Su(t)\right\Vert _{L^\infty }+ \left\Vert \nabla \rho (t)\right\Vert _{L^\infty }, \end{aligned}$$

we obtain as above using the commuting properties between derivatives and *S* that

$$\begin{aligned} \tilde{A}(t)&\lesssim \sum _{k\in \mathbb {Z}}\Vert P_k\nabla \nabla ^ \perp \left\lvert \nabla \right\rvert ^{-1}(e^{-it\Lambda }\mathcal {Z}_+(t)+e^{it\Lambda }\mathcal {Z}_-(t))\Vert _{L^\infty } \\  &\qquad \quad +\left\| P_k \nabla (e^{-it\Lambda }\mathcal {Z}_+(t)-e^{it\Lambda }\mathcal {Z}_-(t))\right\| _{L^\infty }\\  &\qquad \quad +\Vert P_kS\nabla ^\perp \left\lvert \nabla \right\rvert ^{-1}(e^{-it\Lambda }\mathcal {Z}_+(t)+e^{it\Lambda }\mathcal {Z}_-(t))\Vert _{L^\infty }\\  &\lesssim \sum _{k\in \mathbb {Z}} \left\| P_ke^{\mp it \Lambda }\mathcal {Z}_\pm \right\| _{L^\infty }+2^k\left\| P_ke^{\mp it \Lambda }\mathcal {Z}_\pm \right\| _{L^\infty }+\left\| P_ke^{\mp it \Lambda }S\mathcal {Z}_\pm \right\| _{L^\infty }\\  &\lesssim t^{-\frac{1}{2}}\varepsilon . \end{aligned}$$

$$\square $$

### Proof of Proposition 3.2

Without loss of generality let $t>0$, and consider the semigroup given by

3.4 $$\begin{aligned} P_{k,p}e^{it\Lambda }f(x)&=\int _{\mathbb {R}^2}e^{it\Lambda (\xi )+ix\cdot \xi }\widehat{P_{k,p}f}(\xi )d\xi \nonumber \\&=\int _0^\infty \int _{-1}^1 e^{i\Psi (\rho ,\Lambda )}\varphi (2^{-k}\rho )\varphi (2^{-p}\sqrt{1-\Lambda ^2})\hat{f}(\rho ,\Lambda )\frac{\rho }{\sqrt{1-\Lambda ^2}}d\Lambda d\rho ,\nonumber \\ \Psi&:=t\Lambda +x_1\rho \Lambda +x_2\rho \sqrt{1-\Lambda ^2}, \end{aligned}$$

where we have used the polar coordinates notation (2.20).

To begin with, assume that for some $C>0$

$$\begin{aligned} t^{\frac{1}{2}}2^p\le C, \quad \text { or } \quad t2^{-k}\le 1. \end{aligned}$$

Observe that if $\sqrt{1-\Lambda ^2}\sim 2^p\le C t^{-\frac{1}{2}}\ll 1$, on the support of $\varphi _{k,p}$ there holds $\left\lvert \Lambda \right\rvert \ge \frac{1}{2}$. Letting $\overline{\varphi }_{k,p}$ be a function with similar support properties as $\varphi _{k,p}$, by a change of variables $\Lambda \mapsto 2^{-p}\sqrt{1-\Lambda ^2}=y$ and Lemma 3.1 we obtain

3.5 $$\begin{aligned} \begin{aligned} \left\lvert P_{k,p}e^{it\Lambda }f\right\rvert&\lesssim \int _0^\infty \int _{-1}^1 |\varphi (2^{-k}\rho )\varphi (2^{-p}\sqrt{1-\Lambda ^2})\hat{f}|\frac{\rho }{\sqrt{1-\Lambda ^2}}d\Lambda d\rho \\&\lesssim \int _0^\infty |\overline{\varphi }(2^{-k}\rho )|\rho d\rho \int _{-1}^1\left\lvert \overline{\varphi }(y)\right\rvert 2^pdy\Vert \widehat{P_{k,p}f}\Vert _{L^\infty }\\&\lesssim 2^{2k}2^{p}\Vert \widehat{P_{k,p}f}\Vert _{L^\infty }\\&\lesssim 2^{k-4k^+}2^p\left\Vert f\right\Vert _D. \end{aligned} \end{aligned}$$

From now on we assume that

3.6 $$\begin{aligned} t^{\frac{1}{2}}2^p> C \iff 2^{-p}< C^{-1}t^{\frac{1}{2}}, \quad \text {and}\quad t2^{-k}>1. \end{aligned}$$

We decompose

$$\begin{aligned} f=R_{\le l_0}f+(\textrm{Id}-R_{\le l_0})f, \end{aligned}$$

where $l_0$ is the largest integer such that

$$\begin{aligned} 2^{l_0}\le t2^p(t2^{2p})^{-\kappa },\hspace{1cm} 0<\kappa <\frac{(\beta -\beta ^\prime )}{1+\beta }, \end{aligned}$$

and $0<\beta '<\beta$. We then let

$$\begin{aligned} P_{k,p}e^{it\Lambda }f=P_{k,p}R_{\le l_0}e^{it\Lambda }f+P_{k,p}(\textrm{Id}-R_{\le l_0})e^{it\Lambda }f=:I_{k,p}(f)+II_{k,p}(f). \end{aligned}$$

We can estimate the high angular frequencies using the *X*-norm (2.23) to obtain claim (3.3):

$$\begin{aligned} \left\Vert II_{k,p}(f)\right\Vert _{L^2}&\lesssim \sum _{l> l_0,\; p+l\ge 0}\left\Vert P_{k,p}R_lf\right\Vert _{L^2}\lesssim \sum _{l>l_0, \; p+l\ge 0}2^{-4k^+}2^{-(1+\beta )l}2^{-\frac{p}{2}}2^{-\beta p}\left\Vert f\right\Vert _X\\&\lesssim 2^{-4k^+}2^{-(1+\beta )(l_0+1)}2^{-\frac{p}{2}}2^{-\beta p}\left\Vert f\right\Vert _X\\&\lesssim 2^{-4k^+}[t2^p(t2^{2p})^{-\kappa }]^{-(1+\beta )}2^{-\frac{p}{2}}2^{-\beta p}\left\Vert f\right\Vert _X\\&\lesssim 2^{-4k^+} t^{-(1+\beta )}2^{-(1+\beta )p}(t2^{2p})^{(\beta -\beta ')}2^{-\frac{p}{2}-\beta p}\left\Vert f\right\Vert _X\\&\lesssim 2^{-4k^+}t^{-\frac{1}{2}-\beta '}2^{-\frac{p}{2}-2\beta 'p}\left\Vert f\right\Vert _X, \end{aligned}$$

where we have used $\beta ^\prime <\beta$ and $t^{\frac{1}{2}}2^p\ge C$.

From now we assume that $f=R_{\le l_0}f$ and note that by the Bernstein property Proposition 2.3(3) for any $a,b\in \mathbb {N}_0$ it holds that

3.7 $$\begin{aligned} \Vert S^b\partial ^a_\Lambda \hat{f}\Vert _{L^\infty }\lesssim t^a2^{ap}(t2^{2p})^{-\kappa a}\Vert S^b\hat{f}\Vert _{L^\infty }. \end{aligned}$$

In the following we will integrate by parts in the expression (3.4) in different directions. To that end, we compute the derivatives

3.8 $$\begin{aligned}&\partial _\Lambda \Psi =t+ x_1\rho - x_2\rho \frac{\Lambda }{\sqrt{1-\Lambda ^2}},  &   \partial ^2_\Lambda \Psi = -x_2\rho \frac{1}{(1-\Lambda ^2)^{\frac{3}{2}}}, \nonumber \\&\partial _\Lambda \partial _\rho \Psi =x_1-x_2\frac{\Lambda }{\sqrt{1-\Lambda ^2}},  &   \partial _\rho \Psi =x_1\Lambda +x_2\sqrt{1-\Lambda ^2},  &   \partial _\rho ^2\Psi =0. \end{aligned}$$

**Part 1:** Let $p\le -10$. In particular, on the support of $\varphi _{k,p}$ $\left\lvert \Lambda \right\rvert \ge \frac{1}{2}$.

**Case A:** For some $c>2$

$$\begin{aligned} \left\lvert x_1\right\rvert <c^{-1}t2^{2p-k}, \hspace{1.5cm} \left\lvert x_2\right\rvert \le c^{-2}t2^{p-k}. \end{aligned}$$

With (3.8) this implies the following bounds on derivatives of $\Psi$:

$$\begin{aligned} \left\lvert \partial _\Lambda \Psi \right\rvert \ge \left\lvert t+x_1\rho \right\rvert -\left\lvert x_2\right\rvert \rho \frac{\Lambda }{\sqrt{1-\Lambda ^2}}\gtrsim t,\hspace{0.5cm}\left\lvert \partial _\Lambda ^2\Psi \right\rvert \le c^{-2}t2^{p-k}2^k2^{-3p}. \end{aligned}$$

Next we integrate by parts in the expression (3.4) *N* times until $N\kappa \ge 1$. To that end, let

$$\begin{aligned} h(\rho ,\Lambda ):=\varphi (2^{-k}\rho )\varphi (2^{-p}\sqrt{1-\Lambda ^2})\frac{\rho }{\sqrt{1-\Lambda ^2}} \end{aligned}$$

and compute

$$\begin{aligned} \partial _\Lambda h(\rho ,\Lambda )&=\varphi (2^{-k}\rho )\rho \Bigg [2^{-p}\overline{\varphi }(2^{-p}\sqrt{1-\Lambda ^2})\frac{-\Lambda }{1-\Lambda ^2}\\  &\qquad \qquad \qquad +\overline{\varphi }(2^{-p}\sqrt{1-\Lambda ^2})\frac{\Lambda }{(1-\Lambda ^2)^{3/2}}\Big ], \end{aligned}$$

and in particular

3.9 $$\begin{aligned}&\int _0^\infty \int _{-1}^1\left\lvert h\right\rvert d\Lambda d\rho \lesssim 2^{2k}2^p,  &   \int _0^\infty \int _{-1}^1\left\lvert \partial _\Lambda h\right\rvert d\Lambda d\rho \lesssim 2^{2k}2^{-p}. \end{aligned}$$

Using that $e^{i\Psi }=\frac{1}{i\partial _\Lambda \Psi }\partial _\Lambda e^{i\Psi }$ we integrate by parts repeatedly in (3.4) and observe that boundary terms vanish because of the condition (3.6) and $\varphi (0)=0$, we obtain

$$\begin{aligned} I_{k,p}(f)&=\int _0^\infty \int _{-1}^1 e^{i\Psi }h\hat{f}d\Lambda d\rho =-\int _0^\infty \int _{-1}^1 e^{i\Psi }\partial _\Lambda \left( \frac{1}{i\partial _\Lambda \Psi }h\hat{f}\right) d\Lambda d\rho \\&=-\iint e^{i\Psi }\left[ \partial _\Lambda \left( \frac{1}{i\partial _\Lambda \Psi }\right) h-\frac{1}{i\partial _\Lambda \Psi }\partial _\Lambda h\right] \hat{f}d\Lambda d\rho + \iint e^{i\Psi }\frac{1}{i\partial _\Lambda \Psi }h\partial _\Lambda \hat{f}d\Lambda d\rho \\&=-\iint e^{i\Psi }\left[ \frac{-i\partial _\Lambda ^2\Psi }{(i\partial _\Lambda \Psi )^2}h+\frac{1}{i\partial _\Lambda \Psi }\partial _\Lambda h \right] \hat{f}d\Lambda d\rho - \iint e^{i\Psi }\frac{1}{i\partial _\Lambda \Psi }h\partial _\Lambda \hat{f}d\Lambda d\rho . \end{aligned}$$

We integrate by parts again in the second integral and obtain

$$\begin{aligned} I_{k,p}(f)&=-\iint e^{i\Psi }\left[ \frac{-i\partial _\lambda ^2\Psi }{(i\partial _\Lambda \Psi )^2}h +\frac{\partial _\Lambda h}{i\partial _\Lambda \Psi } \right] \hat{f}d\Lambda d\rho \\  &\quad +\iint e^{i\Psi }\left[ \frac{-2i\partial _\Lambda ^2\Psi }{(i\partial _\Lambda \Psi )^3}h+ \frac{\partial _\Lambda h}{(i\partial _\Lambda \Psi )^2}\right] \partial _\Lambda \hat{f} d\Lambda d\rho +\iint e^{i\Psi }\frac{h}{(i\partial _\Lambda \Psi )^2}\partial _\Lambda ^2\hat{f}d\Lambda d\rho . \end{aligned}$$

Continuing *N*-times this integration by parts in the integral with the highest order derivative of $\hat{f}$ until $N\kappa \ge 1$ yields

$$\begin{aligned} I_{k,p}(f)&=\int _0^\infty \int _{-1}^1 e^{i\Psi }h(\rho ,\Lambda )\hat{f}d\Lambda d\rho \\  &= \int _0^\infty \int _{-1}^1 e^{i\Psi }\Bigg [\sum _{j=0}^{N-1}\frac{(-1)^j}{(i\partial _\Lambda \Psi )^j}\Bigg ( \frac{\partial _\Lambda h}{i\partial _\Lambda \Psi } -\frac{(j+1)ih \partial _\Lambda ^2\Psi }{(i\partial _\Lambda \Psi )^2}\Bigg )\partial _\Lambda ^j \hat{f}\\  &\qquad \qquad \qquad \quad + \frac{(-1)^{j+1}h}{(i\partial _\Lambda \Psi )^N} \partial _\Lambda ^N \hat{f}\Bigg ] d\Lambda d\rho \\  &= \sum _{j=0}^{N} I_j. \end{aligned}$$

For $0\le j\le N-1$, with (3.6), (3.7) and (3.9) we can estimate the terms $I_j$ in the sum above as

$$\begin{aligned} \left\lvert I_j\right\rvert&\lesssim \left\lvert i\partial _\Lambda \Psi \right\rvert ^{-j} \int _0^\infty \int _{-1}^1 \Bigg ( \left\lvert \frac{\partial _\Lambda h}{i\partial _\Lambda \Psi }\right\rvert + \left\lvert \frac{h\partial _\Lambda ^2 \Psi }{(i\partial _\Lambda \Psi )^2}\right\rvert \Bigg ) d\Lambda d\rho \left\| \widehat{P_{k,p}\partial _\Lambda ^jf}\right\| _{L^\infty }\\  &\lesssim t^{-j}\Bigg [t^{-1}\int _0^\infty \int _{-1}^1 \left\lvert \partial _\Lambda h\right\rvert d\Lambda d\rho \\  &\quad \qquad +t^{-2}c^{-2}t2^{p-k}2^k 2^{-3p} \int _0^\infty \int _{-1}^1\left\lvert h\right\rvert d\Lambda d\rho \Bigg ] t^{j}2^{jp}(t2^{2p})^{-j\kappa }\Vert \widehat{P_{k,p}f}\Vert _{L^\infty }\\  &\lesssim 2^{2k}t^{-1}2^{-p}\Vert \widehat{P_{k,p}f}\Vert _{L^\infty }\\  &\lesssim 2^{-4k^+}2^{k}t^{-1}2^{-p}\left\| f\right\| _D, \end{aligned}$$

where we have used Lemma 3.1 in the last estimate. On the other hand, when $j=N$ we obtain

$$\begin{aligned} \left\lvert I_N\right\rvert&\lesssim t^{-N}2^{2k}2^pt^N2^{pN}(t2^{2p})^{-N\kappa }\Vert \widehat{P_{k,p}\partial _\Lambda ^Nf}\Vert _{L^\infty }\lesssim 2^{-4k^+}2^kt^{-1}2^{-p}\left\Vert f\right\Vert _D. \end{aligned}$$

**Case B:** Assume

$$\begin{aligned} \left\lvert x_1\right\rvert \ge c^{-1}t2^{2p-k} \text { and } \left\lvert x_2\right\rvert \le c^{-2}t2^{p-k}. \end{aligned}$$

We have the following bound on the radial derivative of $\Psi$

3.10 $$\begin{aligned} \left\lvert \partial _\rho \Psi \right\rvert \gtrsim t2^{2p-k}, \end{aligned}$$

as can be seen from (3.8): Using that $p\le -10$ (and thus $\left\lvert \Lambda \right\rvert \ge \frac{1}{2}$) it follows that $\left\lvert \partial _\rho \Psi \right\rvert \ge \left\lvert x_1\right\rvert \Lambda -\left\lvert x_2\right\rvert \sqrt{1-\Lambda ^2}\gtrsim t2^{2p-k}.$ This allows us to integrate by parts in $\rho$ with $e^{i\Psi }=\frac{1}{i\partial _\rho \Psi }\partial _\rho (e^{i\Psi })$ and obtain

3.11 $$\begin{aligned} P_{k,p}e^{it\Lambda }f(x)&=\int _0^\infty \int _{-1}^1e^{i\Psi }h(\rho ,\Lambda )\hat{f}d\Lambda d\rho \nonumber \\  &=-\int _0^\infty \int _{-1}^1e^{i\Psi } \left[ \frac{\partial _\rho h}{i\partial _\rho \Psi }\hat{f}+\frac{h}{i\partial _\rho \Psi }\partial _\rho \hat{f}\right] d\Lambda d\rho . \end{aligned}$$

Note that

$$\begin{aligned} \partial _\rho h=\varphi (2^{-p}\sqrt{1-\Lambda ^2})\frac{1}{\sqrt{1-\Lambda ^2}}[(\partial _\rho \varphi )(2^{-k}\rho )2^{-k}\rho +\varphi (2^{-k}\rho )], \end{aligned}$$

and therefore

$$\begin{aligned} \int _0^\infty \int _{-1}^1\left\lvert \partial _\rho h\right\rvert d\Lambda d\rho&\lesssim \int _{-1}^1\frac{|\varphi (2^{-p}\sqrt{1-\Lambda ^2})|}{\sqrt{1-\Lambda ^2}}d\Lambda \int _0^\infty \Big [ 2^{-k}|\overline{\varphi }(2^{-k}\rho )\rho |+ |\varphi (2^{-k}\rho )|\Big ]d\rho \\  &\lesssim 2^{p}2^{k}. \end{aligned}$$

With this estimate, Lemma 3.1 and (3.10), the first term in (3.11) can be bounded as

$$\begin{aligned} \left\lvert \int _0^\infty \int _{-1}^1e^{i\Psi }\frac{\partial _\rho h}{i\partial _\rho \Psi }\hat{f}d\Lambda d\rho \right\rvert&\lesssim ct^{-1}2^{-2p+k}\int _0^\infty \int _{-1}^1\left\lvert \partial _\rho h\right\rvert d\Lambda d\rho \Vert \widehat{P_{k,p}f}\Vert _{L^\infty }\\&\lesssim 2^{2k}t^{-1}2^{-p}\Vert \widehat{P_{k,p}f}\Vert _{L^\infty }\\&\lesssim 2^{-4k^+}2^kt^{-1}2^{-p}\left\Vert f\right\Vert _D. \end{aligned}$$

For the second term in (3.11) we recall that $S=\rho \partial _\rho$ and obtain, with Lemma 3.1,

$$\begin{aligned} \left\lvert \int _0^\infty \int _{-1}^1e^{i\Psi } \frac{h}{i\partial _\rho \Psi }\partial _\rho \hat{f}d\Lambda d\rho \right\rvert&\lesssim ct^{-1}2^{-2p+k}\int _{-1}^1 \frac{|\varphi (2^{-p}\sqrt{1-\Lambda ^2})|}{\sqrt{1-\Lambda ^2}}d\Lambda \\  &\quad \cdot \int _0^\infty |\varphi (2^{-k}\rho )|d\rho \Vert \widehat{P_{k,p}Sf}\Vert _{L^\infty }\\  &\lesssim 2^{2k}t^{-1}2^{-p}\Vert \widehat{P_{k,p}Sf}\Vert _{L^\infty }\\  &\lesssim 2^{-4k^+}2^kt^{-1}2^{-p}\left\| f\right\| _D. \end{aligned}$$

Here we note that the decay norm $\left\Vert \cdot \right\Vert _D$ also bounds $\Vert S\hat{f}\Vert _{L^\infty }$ by its definition (3.1).

**Case C:** Here we treat the remaining case

$$\begin{aligned} \left\lvert x_2\right\rvert > c^{-2}t2^{p-k}. \end{aligned}$$

We have the following lower bound:

3.12 $$\begin{aligned} \left\lvert \partial _\rho \Psi \right\rvert + \left\lvert \partial _\rho \partial _\Lambda \Psi \right\rvert \gtrsim 2^{-k}t. \end{aligned}$$

Indeed, from (3.8) it holds that $\Lambda \partial _\Lambda \partial _\rho \Psi - \partial _\rho \Psi =-\frac{x_2}{\sqrt{1-\Lambda ^2}}$ and it follows that

$$\begin{aligned} \left\lvert \partial _\rho \Psi \right\rvert + \left\lvert \partial _\rho \partial _\Lambda \Psi \right\rvert \ge \left\lvert \partial _\rho \Psi \right\rvert + \left\lvert \Lambda \partial _\rho \partial _\Lambda \Psi \right\rvert \ge \left\lvert \partial _\rho \Psi -\Lambda \partial _\rho \partial _\Lambda \Psi \right\rvert >c^{-2}t2^{-k}. \end{aligned}$$

With this we can integrate by parts in $\rho$ or use the set-size gain when integrating in $\Lambda$. To formalize this, we decompose

$$\begin{aligned} P_{k,p}e^{it\Lambda }f=\int _0^\infty \int _{-1}^1e^{i\Psi }\varphi _{k,p}(\rho ,\Lambda )\hat{f}\frac{\rho }{\sqrt{1-\Lambda ^2}}d\Lambda d\rho =\sum _{n\ge 0}I_n, \end{aligned}$$

where $I_n=\int _0^\infty \int _{-1}^1e^{i\Psi }\varphi _{k,p}(\rho ,\Lambda )\varphi (2^{-n}\partial _\rho \Psi )\hat{f}\frac{\rho }{\sqrt{1-\Lambda ^2}}d\Lambda d\rho$. On the support of $I_0$, we have $\left\lvert \partial _\rho \Psi \right\rvert \sim 1$, hence $\left\lvert \partial _\Lambda \partial _\rho \Psi \right\rvert \gtrsim 2^{-k}t$ and with a change variables $y=\partial _\rho \Psi$ we obtain the decay:

$$\begin{aligned} \left\lvert I_0\right\rvert&\lesssim \Vert \widehat{P_{k,p}f}\Vert _{L^\infty } \iint \varphi _{k,p}(\rho ,\Lambda )\varphi (\partial _\rho \Psi )\frac{\rho }{\sqrt{1-\Lambda ^2}}d\Lambda d\rho \\&\lesssim \Vert \widehat{P_{k,p}f}\Vert _{L^\infty }\Vert \varphi _{p}(\Lambda )\sqrt{1-\Lambda ^2}^{-1}\Vert _{L^{\infty }_{\Lambda }} \iint \varphi (\partial _\rho \Psi )\varphi (2^{-k}\rho )\rho d\Lambda d\rho \\&\lesssim 2^{-p}\Vert \widehat{P_{k,p}f}\Vert _{L^\infty } \iint \varphi (y)\left\lvert \partial _\Lambda \partial _\rho \Psi \right\rvert ^{-1}\varphi (2^{-k}\rho )\rho dy d\rho \\&\lesssim 2^{2k-p}t^{-1}\Vert \widehat{P_{k,p}f}\Vert _{L^\infty } \\&\lesssim 2^{k-4k^+}2^{-p}t^{-1}\left\Vert f\right\Vert _D. \end{aligned}$$

The summation for $n\ge 1$ will be split according to (3.12). We observe that $\partial _\rho ^2\Psi =0$. Thus, integrating by parts in $\partial _\rho$ once (respectively twice) gives

$$\begin{aligned} I_n=-I_n^{(1)}=I_n^{(2)}, \end{aligned}$$

where

$$\begin{aligned} I_n^{(1)}&=\iint e^{i\Psi }\frac{\varphi (2^{-n}\partial _\rho \Psi )}{i\partial _\rho \Psi }\partial _\rho (\varphi _{k,p}\hat{f}\frac{\rho }{\sqrt{1-\Lambda ^2}})d\Lambda d\rho ,\\ I_n^{(2)}&=\iint e^{i\Psi }\frac{\varphi (2^{-n}\partial _\rho \Psi )}{(\partial _\rho \Psi )^2}\partial _\rho ^2 (\varphi _{k,p}\hat{f}\frac{\rho }{\sqrt{1-\Lambda ^2}})d\Lambda d\rho . \end{aligned}$$

For a function $\overline{\varphi }_{k,p}$ with similar support properties as $\varphi _{k,p}$ we thus have the bounds

$$\begin{aligned} |I_n^{(1)}|&\lesssim 2^{-n}2^{-p}(\Vert \widehat{P_{k,p}f}\Vert _{L^\infty }+\Vert \widehat{P_{k,p}Sf}\Vert _{L^\infty })\iint \varphi (2^{-n}\partial _\rho \Psi )\overline{\varphi }_{k,p}(\rho ,\Lambda )d\Lambda d\rho \\&\lesssim 2^{-n}2^{-p}2^{-k}2^{-4k^+}\left\Vert f\right\Vert _D\iint \varphi (2^{-n}\partial _\rho \Psi )\overline{\varphi }_{k,p}(\rho ,\Lambda )d\Lambda d\rho \end{aligned}$$

and

3.13 $$\begin{aligned} \begin{aligned} |I_n^{(2)}|&\lesssim 2^{-2n}\iint \varphi (2^{-n}\partial _\rho \Psi )\overline{\varphi }_{k,p}[|\hat{f}|2^{-k}+|\partial _\rho \hat{f}|+2^{k}|\partial _\rho ^2\hat{f}|]\sqrt{1-\Lambda ^2}^{-1}d\Lambda d\rho \\&\lesssim 2^{-2n}2^{-p}2^{-k}\sum _{a=0}^2\Vert \widehat{P_{k,p}S^af}\Vert _{L^\infty } \iint \varphi (2^{-n}\partial _\rho \Psi )\overline{\varphi }_{k,p}(\rho ,\Lambda )d\Lambda d\rho \\&\lesssim 2^{-2n}2^{-p}2^{-2k}2^{-4k^+}\left\Vert f\right\Vert _D\iint \varphi (2^{-n}\partial _\rho \Psi )\overline{\varphi }_{k,p}(\rho ,\Lambda )d\Lambda d\rho . \end{aligned} \end{aligned}$$

Now note that when $\left\lvert \partial _\rho \Psi \right\rvert \sim 2^n\le t2^{-k}$, by (3.12) there must hold that $\left\lvert \partial _\Lambda \partial _\rho \Psi \right\rvert \gtrsim 2^{-k}t$. By changing variables $y=\partial _\rho \Psi$, the set size of integration in $\Lambda$ gives

$$\begin{aligned} \begin{aligned} \iint \varphi (2^{-n}\partial _\rho \Psi )\overline{\varphi }_{k,p}(\rho ,\Lambda )d\Lambda d\rho&\lesssim \iint \varphi (2^{-n}\partial _\rho \Psi )\varphi (2^{k}t^{-1}\partial _\Lambda \partial _\rho \Psi )\overline{\varphi }_{k,p}(\rho ,\Lambda )d\Lambda d\rho \\&\lesssim 2^{n}2^{2k}t^{-1}, \end{aligned} \end{aligned}$$

so that

3.14 $$\begin{aligned} \begin{aligned} |I_n^{(1)}|&\lesssim 2^{k}2^{-4k^+}2^{-p}t^{-1}\left\Vert f\right\Vert _D. \end{aligned} \end{aligned}$$

Similarly, as long as $2^n\le t2^{-k}$, we obtain from (3.13) that

3.15 $$\begin{aligned} |I_n^{(2)}|&\lesssim 2^{-n}2^{-4k^+}2^{-p}t^{-1}\left\Vert f\right\Vert _D. \end{aligned}$$

On the other hand, a simple set size estimate yields

3.16 $$\begin{aligned} |I_n^{(2)}|&\lesssim 2^{-2n}2^{-p}2^{-2k}2^{-4k^+}\left\Vert f\right\Vert _D. \end{aligned}$$

Together, (3.14)–(3.16) show that

$$\begin{aligned} \sum _{n\ge 1}\left\lvert I_n\right\rvert&\lesssim \sum _{1\le n\le \log (t)-k}\min \{|I_n^{(1)}|,|I_n^{(2)}|\}+\sum _{n\ge \log (t)-k}|I_n^{(2)}|\\  &\lesssim 2^{-4k^+}2^{-p}t^{-1} \left\| f\right\| _D\sum _{n\le \log (t)-k}\min \{2^{k},2^{-n}\}\\  &\quad +2^{-p}2^{-2k}2^{-4k^+}\left\| f\right\| _D \sum _{n\ge \log (t)-k}2^{-2n}\\  &\lesssim 2^{\frac{3}{4}k} 2^{-4k^+}2^{-p}t^{-1}\left\| f\right\| _D+2^{\frac{3}{4}k}2^{-\frac{15}{4}k^+}2^{-p}t^{-1}\left\| f\right\| _D. \end{aligned}$$

**Part 2:** Fix $p\ge -10$. We first observe that **Cases A** and and **C** follow exactly as above, since we don’t use the largeness of $\Lambda$.

**Case B.** First note that in **Part 1** we explicitly used the smallness of the parameter *p* and the fact that $\left\lvert \Lambda \right\rvert$ was bounded from below. In the current setting, we need to invoke the horizontal localization $\left\lvert \Lambda \right\rvert \sim 2^q$, $q\in \mathbb {Z}^-$ introduced in Sect. 2.3. Moreover, we will use the $\varphi _{k,p,q}(\rho ,\Lambda )$ functions, as well as the following fact for fixed $k,\,p$:

$$\begin{aligned} \left\lvert P_{k,p}e^{it\Lambda }f\right\rvert&\le \sum _{q\in \mathbb {Z}^-}\left\lvert P_{k,p,q}e^{it\Lambda }f\right\rvert \\  &= \sum _{q\le -\log (t)}\left\lvert P_{k,p,q}e^{it\Lambda }f\right\rvert +\sum _{-\log (t)\le q\le 0}\left\lvert P_{k,p,q}e^{it\Lambda }f\right\rvert . \end{aligned}$$

First of all, we observe that if $q\le -\log (t)$ (and since $p\ge -10$ is fixed),

$$\begin{aligned} \left\lvert P_{k,p,q}e^{it\Lambda }f\right\rvert&\lesssim \iint \varphi _{k,p,q}(\rho ,\Lambda )\frac{\rho }{\sqrt{1-\Lambda ^2}}\hat{f}d\Lambda d\rho \\&\lesssim \Vert \widehat{P_{k,p}f}\Vert _{L^\infty }\iint \overline{\varphi }(2^{-k}\rho )\overline{\varphi }(2^{-q}\Lambda )\rho d\Lambda d\rho \\&\lesssim 2^{2k}2^q\Vert \widehat{P_{k,p}f}\Vert _{L^\infty }, \end{aligned}$$

which implies using Lemma 3.1 that

$$\begin{aligned} \sum _{q\le -\log (t)}\left\lvert P_{k,p,q}e^{it\Lambda }f\right\rvert \lesssim 2^{k}2^{-4k^+}t^{-1}\left\Vert f\right\Vert _D. \end{aligned}$$

To deal with the summation for $q\ge -\log (t)$, we proceed similarly to **Case C** above, however with a *q*-dependence. Observe that in the setting of **Case B**

$$\begin{aligned} 2^q\left\lvert \partial _\rho \Psi \right\rvert + \left\lvert \partial _\rho \partial _\Lambda \Psi \right\rvert \gtrsim 2^{-k}t. \end{aligned}$$

This can be seen from (3.8), which implies that

$$\begin{aligned} \partial _\Lambda \partial _\rho \Psi +\frac{\Lambda }{1-\Lambda ^2}\partial _\rho \Psi =\frac{x_1}{1-\Lambda ^2} \hspace{0.5cm} \Longrightarrow \hspace{0.5cm} (1-\Lambda ^2)\partial _\Lambda \partial _\rho \Psi +\Lambda \partial _\rho \Psi =x_1, \end{aligned}$$

so that in particular

$$\begin{aligned} \left\lvert \partial _\Lambda \partial _\rho \Psi \right\rvert +2^q\left\lvert \partial _\rho \Psi \right\rvert \ge \left\lvert (1-\Lambda ^2)\partial _\Lambda \partial _\rho \Psi +\Lambda {\partial _\rho \Psi }\right\rvert = \left\lvert x_1\right\rvert \ge c^{-1}t2^{-k}. \end{aligned}$$

We decompose the semigroup

3.17 $$\begin{aligned} P_{k,p,q}e^{it\Lambda }f=\int _0^\infty \int _{-1}^1e^{i\Psi }\varphi _{k,p,q}(\rho ,\Lambda )\hat{f}\frac{\rho }{\sqrt{1-\Lambda ^2}}d\Lambda d\rho =\sum _{n\ge 0}I_n, \end{aligned}$$

where $I_n=\int _0^\infty \int _{-1}^1e^{i\Psi }\varphi _{k,p,q}(\rho ,\Lambda )\varphi (2^{-n}\partial _\rho \Psi )\hat{f}\frac{\rho }{\sqrt{1-\Lambda ^2}}d\Lambda d\rho$. Again, we want to either integrate by parts in $\partial _\rho$ and make use of the fact that $\left\lvert \partial _\Lambda \partial _\rho \Psi \right\rvert ^{-1}\lesssim 2^{k}t^{-1}$, or employ a set size bound. To that end, observe first that on the support of $I_0$ we have $\left\lvert \partial _\rho \Psi \right\rvert \sim 1$ and thus

$$\begin{aligned} 1+\left\lvert \partial _\rho \partial _\Lambda \Psi \right\rvert \gtrsim 2^q\left\lvert \partial _\rho \Psi \right\rvert +\left\lvert \partial _\rho \partial _\Lambda \Psi \right\rvert \gtrsim 2^{-k}t \hspace{0.5cm} \Longrightarrow \hspace{0.5cm} \left\lvert \partial _\Lambda \partial _\rho \Psi \right\rvert \gtrsim 2^{-k}t. \end{aligned}$$

Therefore, by a change of variables $y=\partial _\rho \Psi$, Lemma 3.1 and $2^{-p}\lesssim 2^{10}$, we obtain

$$\begin{aligned} \left\lvert I_0\right\rvert&\lesssim \Vert \widehat{P_{k,p}f}\Vert _{L^\infty } \iint \overline{\varphi }_{k,p,q}(\rho ,\Lambda )\varphi (\partial _\rho \Psi )\frac{\rho }{\sqrt{1-\Lambda ^2}}d\Lambda d\rho \\&\lesssim \Vert \widehat{P_{k,p}f}\Vert _{L^\infty }\Vert \overline{\varphi }_{k,p,q}(\Lambda )\sqrt{1-\Lambda ^2}^{-1}\Vert _{L^{\infty }_\Lambda }\iint \varphi (\partial _\rho \Psi ) \varphi (2^{-k}\rho ) \rho d\Lambda d\rho \\&\lesssim 2^{2k}\Vert \widehat{P_{k,p}f}\Vert _{L^\infty } \int \varphi (y)\left\lvert \partial _\Lambda \partial _\rho \Psi \right\rvert ^{-1} dy\\&\lesssim 2^{k-4k^+}t^{-1}\left\Vert f\right\Vert _D. \end{aligned}$$

We highlight here that we use the $P_{k,p}$ projections to bound the Fourier transform with Lemma 3.1. For $n\ge 1$, we integrate by parts either once or twice in $\partial _\rho$ using $\partial _\rho ^2\Psi =0$ and obtain as in **Part 1**

$$\begin{aligned} |I_n^{(1)}|&\lesssim 2^{-n}2^{-k}2^{-4k^+}\left\Vert f\right\Vert _D\iint \varphi (2^{-n}\partial _\rho \Psi )\overline{\varphi }_{k,p,q}(\rho ,\Lambda )d\Lambda d\rho ,\\ |I_n^{(2)}|&\lesssim 2^{-2n}2^{-2k}2^{-4k^+}\left\Vert f\right\Vert _D\iint \varphi (2^{-n}\partial _\rho \Psi )\overline{\varphi }_{k,p,q}(\rho ,\Lambda )d\Lambda d\rho . \end{aligned}$$

Similarly as in **Part 1**, we decompose the sum over all $n\ge 1$ into the part where $q+n\le \log (t)-k$ and $q+n\ge \log (t)-k$.

If $q+n\le \log (t)-k$ then $2^q\left\lvert \partial _\rho \Psi \right\rvert \sim 2^{q+n}\lesssim 2^{-k}t$ and so necessarily $\left\lvert \partial _\Lambda \partial _\rho \Psi \right\rvert \gtrsim 2^{-k}t$. As before we obtain the bounds (3.14) and (3.15) by the change of variables $y=\partial _\rho \Psi$ and Lemma 3.1. Summing in *n* yields

$$\begin{aligned} \sum _{q+n\le \log (t)-k}\left\lvert I_n\right\rvert&\lesssim 2^{-4k^+}t^{-1}\left\Vert f\right\Vert _D\sum _{q+n\le \log (t)-k}\min \{2^k,2^{-n}\}\lesssim 2^{\frac{3}{4}k}2^{-4k^+}t^{-1}\left\Vert f\right\Vert _D. \end{aligned}$$

On the other hand, for $q+n\ge \log (t)-k$, we obtain with (3.15) that

$$\begin{aligned} \sum _{q+n\ge \log (t)-k}\left\lvert I_n\right\rvert&\lesssim \sum _{q+n\ge \log (t)-k}2^{-2n}2^{-2k}2^{-4k^+}\left\Vert f\right\Vert _D\iint \overline{\varphi }_{k,p,q}(\rho ,\Lambda )d\Lambda d\rho \\&\lesssim 2^q 2^{-k}2^{-4k^+}\left\Vert f\right\Vert _D \sum _{q+n\ge \log (t)-k}2^{-2n} \\&\lesssim 2^k2^{-4k^+}t^{-2}\left\Vert f\right\Vert _D. \end{aligned}$$

Thus from (3.17) we have

$$\begin{aligned} \left\lvert P_{k,p,q}e^{it\Lambda }f\right\rvert \lesssim 2^{\frac{3}{4}k}2^{-4k^+}t^{-1}\left\Vert f\right\Vert _D\max \{2^{\frac{k}{4}},1\}\lesssim 2^{\frac{3}{4}k}2^{-\frac{15}{4}k^+}t^{-1}\left\Vert f\right\Vert _D, \end{aligned}$$

and altogether

$$\begin{aligned} \sum _{-\log (t)\le q} \hspace{-0.3cm}\left\lvert P_{k,p,q}e^{it\Lambda }f\right\rvert&= \sum _{-\log (t)\le q}\sum _{n\ge 0}\left\lvert I_n\right\rvert \\  &\lesssim \hspace{-0.3cm} \sum _{-\log (t)\le q}\hspace{-0.3cm} 2^{\frac{3}{4}k}2^{-\frac{15}{4}k^+}t^{-1}\left\| f\right\| _D \\  &\lesssim 2^{\frac{3}{4}k}2^{-\frac{15}{4}k^+}\log (t)t^{-1}\left\| f\right\| _D. \end{aligned}$$

$$\square $$

In the bootstrap setting (2.26), the above proposition can be applied directly to $S^b\mathcal {\mathcal {Z}}_\pm (t)$ and $S^b \Theta (t)$ where $0\le b\le N-2$, as is clear from the two copies of *S* required in the decay norm (3.1). Thanks to interpolation, we can furthermore obtain some decay also for the remaining powers of vector fields on the profiles:

### Lemma 3.4

Let $F\in \{\mathcal {Z}_\pm ,\Theta \}$ and assume the bootstrap condition (2.26) holds. Moreover, assume the number of vector fields $M>0$ in (2.24) ((2.25) resp.) is sufficiently large and let $0<\kappa \ll \beta$. Then the weaker decay holds:

$$\begin{aligned} \Vert P_ke^{it\Lambda }S^bF\Vert _{L^\infty }\lesssim 2^{\frac{3}{4}k-3k^+}t^{-\frac{1}{2}+\kappa }\varepsilon , \hspace{1cm} 0\le b\le N. \end{aligned}$$

### Proof

For $b\le N-2$, the decay norm $\Vert S^b F\Vert _D$ is bounded since the bootstrap assumption (2.26) holds. Therefore by Proposition 3.2 there holds:

$$\begin{aligned} \Vert P_ke^{it\Lambda }S^bF\Vert _{L^\infty }\lesssim 2^{\frac{3}{4}k-\frac{15}{4}k^+}t^{-\frac{1}{2}}\varepsilon . \end{aligned}$$

For $N-2< b\le N$ we use interpolation: For integers $r,K\ge 1,$ $a,b\ge 0$ and $\Vert S^{\le b}F\Vert _{L^r}:=\sup _{0\le \alpha \le b}\left\Vert S^\alpha F\right\Vert _{L^r}$ a standard convexity argument (see e.g. [32, Lemma A.6]) gives that

$$\begin{aligned} \Vert S^{\le b}g\Vert _{L^{2r}}\lesssim _{K,r,b}\left\Vert g\right\Vert _{L^{2r}}^{1-\frac{1}{K}}\Vert S^{\le Kb}g\Vert _{L^{2r}}^{\frac{1}{K}}. \end{aligned}$$

Applying this with $g=P_kS^{b-2}F$, Proposition 3.2 and the energy estimates (2.27) with $M\gg 1$ sufficiently large (in particular such that $N+2(K-1)<M$), there holds with $r\sim K\gg \kappa ^{-1}$ that

$$\begin{aligned}&\Vert P_ke^{it\Lambda }S^bF\Vert _{L^\infty }\\  &\quad \lesssim 2^{\frac{k}{r}}\Vert P_ke^{it\Lambda }S^bF\Vert _{L^{2r}}\\  &\quad \lesssim 2^{\frac{k}{r}}2^{k\frac{r-1}{rK}}\Vert P_ke^{it\Lambda }S^{b-2}F\Vert _{L^\infty }^{(1-\frac{1}{r})(1-\frac{1}{K})}\Vert P_kS^{b-2}F\Vert _{L^2}^{\frac{1}{r}(1-\frac{1}{K})}\Vert P_kS^{\le b+2(K-1)}F\Vert _{L^2}^{\frac{1}{K}}\\  &\quad \lesssim 2^{\frac{k}{r}}2^{k\frac{r-1}{rK}}(2^{\frac{3}{4}k-\frac{15}{4}k^+}t^{-\frac{1}{2}}\varepsilon )^{(1-\frac{1}{r})(1-\frac{1}{K})}\varepsilon ^{\frac{1}{r}(1-\frac{1}{K})}\varepsilon ^{\frac{1}{K}}\\  &\quad \lesssim 2^{\frac{3}{4}k-3k^+}t^{-\frac{1}{2}+\frac{1}{K}}\varepsilon . \end{aligned}$$

$$\square $$

## Energy Estimates

In this section we establish energy estimates for the systems (1.3) and (1.4). We give first the full details for the Boussinesq system (1.3), where also the appropriate choice of scalar unknowns plays an important role, see Corollary 4.3. The SQG setting (1.4) can then be dealt with analogously.

### Energy Estimates Boussinesq System

We establish energy estimates for the Boussinesq system (1.3). On the one hand we have the classical $H^n$ estimates on $(u,\rho )$. On the other hand, we provide $L^2$ estimates for arbitrarily many vector fields *S*. Recall the scaling vector field $\mathcal {S}=-\textrm{id}+S$ from (2.13). If $(u,\rho )$ is a solution to (1.3), then $(\mathcal {S}^n u,\mathcal {S}^n\rho )$ satisfies

4.1 $$\begin{aligned} \left\{ \begin{aligned}&\partial _t\mathcal {S}^nu +\mathcal {S}^n(u\cdot \nabla u)=-\mathcal {S}^n\nabla p -\mathcal {S}^n\rho \vec {e_2}\\&\partial _t\mathcal {S}^n\rho + \mathcal {S}^n( u\cdot \nabla \rho )=\mathcal {S}^nu_2 \\&\textrm{div}u=0. \end{aligned} \right. \end{aligned}$$

#### Remark 4.1

1. We obtain energy estimates first on the vector field $\mathcal {S}$ using (4.1). These yield estimates on $S=x\cdot \nabla _x$ since $S^n=\sum _{k=0}^nc_k\mathcal {S}^k$ for binomial constants $c_k>0$.
2. We note that with a similar proof as below we obtain $\dot{H}^{-1}$ estimates on arbitrarily many vector fields *S* applied to a solution (*u*,$\rho$) to (1.3), provided that the initial data $(u_0,\rho _0)\in (H^{-1}\cap H^n)\times (H^{-1}\cap H^n)$.

#### Proposition 4.2

Let $(u,\rho )\in C([0,T],H^n(\mathbb {R}^2))\times C([0,T],H^n(\mathbb {R}^2))$ solve (1.3) with initial data $(u_0,\rho _0)\in C([0,T],H^n(\mathbb {R}^2))\times C([0,T],H^n(\mathbb {R}^2))$ for $T\ge 0$ and some $n\in \mathbb {N}$. Then for $0\le t\le T$

$$\begin{aligned}&\left\| u(t)\right\| _{H^{n}}^2+\left\| \rho (t)\right\| _{H^{n}}^2- \left\| u_0\right\| _{H^{n}}^2-\left\| \rho _0\right\| _{H^{n}}^2\\  &\qquad \qquad \qquad \qquad \qquad \qquad \qquad \qquad \qquad \qquad \quad \lesssim \int _0^tA(s)(\left\| u(s)\right\| _{H^n}^2 +\left\| \rho (s)\right\| _{H^{n}}^2)(1+s)^{-\frac{1}{2}}ds,\\  &\Vert {S}^nu(t)\Vert _{L^2}^2+\Vert {S}^n\rho (t)\Vert _{L^2}^2-\Vert {S}^nu_0\Vert _{L^2}^2-\Vert {S}^n\rho _0\Vert _{L^2}^2 \\  &\quad \lesssim \int _0^t A_1(s)\big (\left\| u(s)\right\| _{H^n}^2+\left\| \rho (s)\right\| _{H^n}^2+\sum _{j=0}^n\Vert {S}^ju(s)\Vert _{L^2}^2+\Vert {S}^j\rho (s)\Vert _{L^2}^2\big )(1+s)^{-\frac{1}{2}}ds, \end{aligned}$$

where for $$0\le s\le t$$

$$\begin{aligned} A_0(s)&:=(\left\| \nabla u\right\| _{L^\infty }+\left\| \nabla \rho \right\| _{L^\infty })(1+s)^{\frac{1}{2}}, \\ A_1(s)&:=(\left\| \nabla u\right\| _{L^\infty }+\Vert {S}u\Vert _{L^\infty }+\left\| \nabla \rho \right\| _{L^\infty })(1+s)^{\frac{1}{2}}. \end{aligned}$$

#### Proof

The first statement follows by standard Sobolev energy estimates, see [12, Proposition 2.5], hence we only give the details for the energy estimate involving *S*.

Observe that $[\mathcal {S},\nabla ]=\nabla$ and by iteration, we have the commutator rule

4.2 $$\begin{aligned} {[}\mathcal {S}^n,\nabla ]=\sum _{k=0}^{n-1}c_k\nabla \mathcal {S}^k=\sum _{k=0}^{n-1}\tilde{c}_k \mathcal {S}^k\nabla ,  &   c_k,\tilde{c}_k\in \mathbb {Z}. \end{aligned}$$

By taking a scalar product with $\mathcal {S}^nu$ and $\mathcal {S}^n\rho$ respectively in (4.1), we obtain

$$\begin{aligned} \left\{ \begin{aligned}&\frac{1}{2}\frac{d}{dt}\Vert \mathcal {S}^nu\Vert _{L^2}+\langle \mathcal {S}^n(u\cdot \nabla u), \mathcal {S}^nu\rangle _{L^2}=-\langle \mathcal {S}^n \nabla p, \mathcal {S}^nu\rangle _{L^2}-\langle \mathcal {S}^n\rho \vec {e_2}, \mathcal {S}^nu\rangle _{L^2}\\&\frac{1}{2}\frac{d}{dt}\Vert \mathcal {S}^n\rho \Vert _{L^2}+\langle \mathcal {S}^n(u\cdot \nabla \rho ), \mathcal {S}^n\rho \rangle _{L^2}=\langle \mathcal {S}^nu_2, \mathcal {S}^n\rho \rangle _{L^2}. \end{aligned} \right. \end{aligned}$$

Adding the two equations, we observe that the terms $-\langle \mathcal {S}^n\rho \vec {e_2}, \mathcal {S}^nu\rangle _{L^2}+\langle \mathcal {S}^nu_2, \mathcal {S}^n\rho \rangle _{L^2}$ cancel. Moreover, since $\mathcal {S}\nabla p=(\textrm{id}-S)\nabla p$ an integration by parts yields

$$\begin{aligned} -\langle \mathcal {S}^n \nabla p, \mathcal {S}^nu\rangle _{L^2}&=-\langle \sum _{k=0}^{n-1}c_k\nabla \mathcal {S}^k p, \mathcal {S}^nu\rangle _{L^2}=\langle \sum _{k=0}^{n-1}c_k\mathcal {S}^k p, \nabla \cdot \mathcal {S}^nu\rangle _{L^2}\\&=\sum _{k,j=0}^{n-1}\langle c_k\mathcal {S}^k p, \tilde{c}_j\mathcal {S}^j\nabla \cdot u\rangle _{L^2}=0. \end{aligned}$$

It remains to bound the terms $\langle \mathcal {S}^n(u\cdot \nabla u), \mathcal {S}^nu\rangle _{L^2}$ and $\langle \mathcal {S}^n(u\cdot \nabla \rho ), \mathcal {S}^n\rho \rangle _{L^2}$. We bound the first scalar product. By the Lebniz rule and commutator rule (4.2) we have

4.3 $$\begin{aligned} \langle \mathcal {S}^n(u\cdot \nabla u), \mathcal {S}^nu\rangle _{L^2}=\langle \sum _{k=0}^n\mathcal {S}^ku\sum _{j=0}^{n-k}\nabla \mathcal {S}^{j}u, \mathcal {S}^nu\rangle _{L^2}. \end{aligned}$$

Observe that for $k=0$ and $k=n$ there holds by incompressibility of *u*

$$\begin{aligned} \langle u\mathcal {S}^n\nabla u, \mathcal {S}^nu\rangle _{L^2}&=\sum _{j=0}^{n-1}\langle u\nabla \mathcal {S}^ju, \mathcal {S}^nu\rangle _{L^2} +\langle u\nabla \mathcal {S}^nu, \mathcal {S}^nu\rangle _{L^2} \\&\lesssim \left\| u\right\| _{L^\infty }\sum _{j=0}^{n-1}\Vert \nabla \mathcal {S}^ju\Vert _{L^2}\Vert \mathcal {S}^nu\Vert _{L^2},\\ \langle \mathcal {S}^nu\nabla u, \mathcal {S}^nu\rangle _{L^2}&\lesssim \left\| \nabla u\right\| _{L^\infty }\Vert \mathcal {S}^nu\Vert _{L^2}. \end{aligned}$$

Thus in order to prove the claim, with (4.3) it remains to establish the following bounds for $1\le j\le n-1$

$$\begin{aligned} \Vert \nabla \mathcal {S}^ju\Vert _{L^2}&\lesssim \sum _{k=0}^n \Vert \mathcal {S}^ku\Vert _{L^2}^2+\left\Vert u\right\Vert _{H^n}^2, \\ \langle \mathcal {S}^ju \mathcal {S}^{n-j}\nabla u, \mathcal {S}^nu\rangle _{L^2}&\lesssim (\Vert \mathcal {S}u\Vert _{L^\infty }+\Vert \nabla u\Vert _{L^\infty })(\sum _{k=0}^n\Vert \mathcal {S}^ku\Vert _{L^2}^2+\left\Vert u\right\Vert _{H^n}^2) . \end{aligned}$$

These follow from integration by parts using $\langle (\mathcal {S}-2\textrm{id})f, g\rangle _{L^2}=-\langle f, \mathcal {S}g\rangle _{L^2}$ and the commutator rule (4.2) and standard interpolation – see e.g. [25, proof of Proposition 5.1, Lemma 5.3]. $\square$

#### Corollary 4.3

Let $Z_\pm$, $\mathcal {Z}_\pm$ be the dispersive unknowns resp. profiles (2.2) of (1.3) and $t\in [0,T]$ with $T\lesssim \varepsilon ^{-2}$, *M* as in Theorem 2.4. Then under the bootstrap assumptions (2.26) that

$$\begin{aligned} \left\Vert Z_\pm (t)\right\Vert _{H^{N_0}}+\sum _{a=0}^M\Vert S^aZ_\pm (t)\Vert _{L^2}=\left\Vert \mathcal {Z}_\pm (t)\right\Vert _{H^{N_0}}+\sum _{a=0}^M\Vert S^a\mathcal {Z}_\pm (t)\Vert _{L^2}\lesssim \varepsilon . \end{aligned}$$

#### Proof

With Corollary 3.3 and under the bootstrap assumption (2.26), we observe that for $s>0$

$$\begin{aligned} A_j(s)\lesssim \left\lvert s\right\rvert ^{-\frac{1}{2}}\sum _{\mu \in \{\pm \}}(\left\Vert {\mathcal {Z}}_\mu \right\Vert _D+\left\Vert S{\mathcal {Z}}_{\mu }\right\Vert _D) (1+s)^{\frac{1}{2}}\lesssim \varepsilon ,  &   j=0,1. \end{aligned}$$

The claim then follows from (2.8) resp. (2.16) and Gronwall’s lemma: we obtain for $t\lesssim \varepsilon ^{-2}$ that

$$\begin{aligned} \left\Vert Z_\pm (t)\right\Vert _{H^{N_0}}^2+\sum _{a=0}^M\Vert S^aZ_\pm (t)\Vert _{L^2}^2\lesssim \varepsilon ^2\exp \Big (\int _0^t c \varepsilon (1+s)^{-\frac{1}{2}}ds \Big )\lesssim \varepsilon ^2. \end{aligned}$$

$$\square $$

### Energy Estimates SQG Equation

For the SQG equation (1.4) we have by (2.14) that

$$\begin{aligned} \partial _t \mathcal {S}^n\theta +\mathcal {S}^n(u\cdot \nabla \theta )=R_1\mathcal {S}^n\theta . \end{aligned}$$

The energy estimates for $\mathcal {S}^n\theta$ then yield estimates for $S^n\theta$ as in the previous section.

#### Proposition 4.4

Let $\theta$ be a solution to (1.4) on $0\le t\le T$ and $n\in \mathbb {N}$. Then

$$\begin{aligned} \left\Vert \theta (t)\right\Vert _{H^n}^2-\left\Vert \theta _0\right\Vert _{H^n}^2&\lesssim \int _0^t \tilde{A}_0(s)\left\Vert \theta (s)\right\Vert _{H^n}^2\frac{1}{(1+\left\lvert s\right\rvert )^{\frac{1}{2}}}ds,\\ \Vert {S}^n\theta (t)\Vert _{L^2}^2-\Vert {S}^n\theta _0\Vert _{L^2}^2&\lesssim \int _0^t \tilde{A}_1(s)(\left\Vert \theta (s)\right\Vert ^2_{H^n}+\sum _{j\le n}\Vert {S}^j\theta (s)\Vert _{L^2}^2)\frac{1}{(1+\left\lvert s\right\rvert )^{\frac{1}{2}}}ds, \end{aligned}$$

where $$\tilde{A_0}(s)=(\left\Vert \nabla \theta \right\Vert _{L^\infty }+\left\Vert \nabla u\right\Vert _{L^\infty })(1+\left\lvert s\right\rvert )^{\frac{1}{2}},\; \tilde{A_1}(s)=(\left\Vert \nabla \theta \right\Vert _{L^\infty }+\Vert u\Vert _{L^\infty }+\Vert \mathcal {S} u\Vert _{L^\infty })(1+|s|)^{\frac{1}{2}}.$$

#### Corollary 4.5

Under the bootstrap assumption (2.26) and with *M* as in Theorem 2.5, for $t\in [0,T]$ and $T\lesssim \varepsilon ^{-2}$

$$\begin{aligned} \left\Vert \theta (t)\right\Vert _{H^{N_0}}+\sum _{a=0}^M \left\Vert S^a\theta (t)\right\Vert _{L^2}=\left\Vert \Theta (t)\right\Vert _{H^{N_0}}+\sum _{a=0}^M \left\Vert S^a\Theta (t)\right\Vert _{L^2}\lesssim \varepsilon , \end{aligned}$$

where $\Theta (t)=e^{it\Lambda }\theta (t)$ is the profile of $\theta$.

## Oscillatory Toolbox: Integration by Parts along Vector Fields and Normal Forms

In this section we present the technical tools used to establish the main results. After some preliminary computations for vector fields in Sect. 5.1, in Sect. 5.2 we construct a class of multipliers that contains those of our bilinear terms and is closed under the action of *S*. Moreover, for these we can track bounds in terms of our localisation parameters $k,p,l,k_i,p_i,l_i$ for $i=1,2$ when iteratively applying *S*. The action of the vector fields *S*, *W* on the phases $\Phi$ are discussed in Sect. 5.3. In particular, we present a result that guarantees either largeness of $\Phi$ or lower bounds for $S\Phi$, see Proposition 5.8. A robust method for integrating by parts along *S* in bilinear expressions involving multipliers of the class previously defined is presented in Sect. 5.4, and combines the multiplier mechanics and some basic vector field algebra, quantified via the localizations introduced in Sect. 2.3. In particular, here the angular projectors $R_l$ are used to precisely capture under which conditions repeated integration by parts is feasible. The action of the vector field *W* on bilinear expressions is also discussed in Sect. 5.4. In Sect. 5.5 we present a lemma that serves to organize the proofs in the sections to follow according to the relative size of the localization parameters involved. In Sect. 5.6 we discuss possible gains due to small sets of integration, and finally, in Sect. 5.7, we present normal forms.

### Vector Field Lemmas

We will now discuss the action of *S* and *W*. Since different variables are involved we will sometimes highlight the variables on which these vector fields are acting explicitly, recalling from (2.13) that

$$\begin{aligned} S_x=x\cdot \nabla _x,\quad W_x=x^\perp \cdot \nabla _x. \end{aligned}$$

To integrate by parts in bilinear forms such as (2.4), we further define

$$\begin{aligned}&S_{\xi -\eta }:=(\xi -\eta )\nabla _\eta ,  &   W_{\xi -\eta }:=(\xi -\eta )^\perp \nabla _\eta . \end{aligned}$$

When there is no risk of confusion, we will suppress the explicit dependence of *S*, *W*.

#### Lemma 5.1

For $$x,y\in \mathbb {R}^2$$ that

1. $$\partial _{x_1}=\frac{x^\perp }{\left\lvert x\right\rvert ^2}\cdot (W_x,S_x)^T$$ and $$ \partial _{x_2}=\frac{x}{\left\lvert x\right\rvert ^2}\cdot (W_x,S_x)^T,$$
2. $$y_1\partial _{x_1}+y_2\partial _{x_2}=\frac{y\cdot x}{\left\lvert x\right\rvert ^2}S+\frac{y\cdot x^\perp }{\left\lvert x\right\rvert ^2}W_x$$.

#### Proof

We compute directly that

$$\begin{aligned}&\frac{x^\perp }{\left\lvert x\right\rvert ^2}\cdot (W,S)^T=\frac{1}{\left\lvert x\right\rvert ^2}[-x_2(-x_2\partial _{x_1}+x_1\partial _{x_2})+x_1(x_1\partial _{x_1}+x_2\partial _{x_2})]=\partial _{x_1},\\&\frac{x}{\left\lvert x\right\rvert ^2}\cdot (W,S)^T=\frac{1}{\left\lvert x\right\rvert ^2}[x_1(-x_2\partial _{x_1}+x_1\partial _{x_2})+x_2(x_1\partial _{x_1}+x_2\partial _{x_2})]=\partial _{x_2}, \end{aligned}$$

and the second statement also follows by direct computation. $\square$

Since *S*, *W* span the tangent space at any point, they allow us to resolve any derivative as follows:

#### Lemma 5.2

5.1 $$\begin{aligned} \begin{aligned}&S_\eta =\frac{\eta (\xi -\eta )}{\left\lvert \xi -\eta \right\rvert ^2}S_{\xi -\eta }-\frac{\eta (\xi -\eta )^\perp }{\left\lvert \xi -\eta \right\rvert ^2}W_{\xi -\eta }, \hspace{0.5cm}S_{\xi -\eta }=\frac{(\xi -\eta )\eta }{\left\lvert \eta \right\rvert ^2}S_\eta +\frac{(\xi -\eta )\eta ^\perp }{\left\lvert \eta \right\rvert ^2}W_\eta ,\\  &W_\xi =\frac{(\xi -\eta )\xi }{\left\lvert \xi -\eta \right\rvert ^2}W_{\xi -\eta }-\frac{(\xi -\eta )^\perp \xi }{\left\lvert \xi -\eta \right\rvert ^2}S_{\xi -\eta }. \end{aligned} \end{aligned}$$

#### Proof

The first statement in (5.1) follows from Lemma 5.1 with $x=\xi -\eta$ and $y=\eta$,

$$\begin{aligned} \partial _{\eta _1}(f(\xi -\eta ))= \frac{(\xi -\eta )^\perp }{\left\lvert \xi -\eta \right\rvert ^2}(W_{\xi -\eta },S_{\xi -\eta })^T,  &   \partial _{\eta _2}(f(\xi -\eta ))= \frac{(\xi -\eta )}{\left\lvert \xi -\eta \right\rvert ^2}(W_{\xi -\eta },S_{\xi -\eta })^T. \end{aligned}$$

so that

$$\begin{aligned} S_\eta =\eta \nabla _\eta =\frac{\eta (\xi -\eta )}{\left\lvert \xi -\eta \right\rvert ^2}S_{\xi -\eta }-\frac{\eta (\xi -\eta )^\perp }{\left\lvert \xi -\eta \right\rvert ^2}W_{\xi -\eta }. \end{aligned}$$

The second statement in (5.1) follows from Lemma 5.1 with $x=\eta$ and $y=\xi -\eta$, while to obtain the third statement take $x=\xi -\eta$ and $y=\xi$. $\square$

### Multiplier Mechanics

For repeated integration by parts in bilinear terms, it is important to understand how the vector field *S* acts on the multipliers and phase functions present. In our framework, this is quantified in terms of the localization parameters defined in Sect. 2.3.

We begin by defining the set of elementary multipliers

$$\begin{aligned} E:=\left\{ \frac{\zeta \cdot \theta ^\perp }{\left\lvert \zeta \right\rvert \left\lvert \theta \right\rvert },\frac{\zeta \cdot \theta }{\left\lvert \zeta \right\rvert \left\lvert \theta \right\rvert }, \Lambda (\zeta ), \sqrt{1-\Lambda ^2(\zeta )} \mid \zeta , \theta \in \{\xi , \xi -\eta ,\eta \} \right\} . \end{aligned}$$

Elements $e\in E$ satisfy $\left\lvert e\right\rvert \le 1$ and we will show that the set of linear combinations of products of such elements is closed under the action of $S_\eta$, $S_{\xi -\eta }$. We define the following sets to track the order of the multipliers:

$$\begin{aligned} E_0&:=\text{ span}_\mathbb {R}\left\{ \prod _{i=1}^Ne_i \mid e_i \in E,\, N\in \mathbb {N} \right\} , \\ E_a^b&:=\text{ span}_\mathbb {R}\left\{ \left\lvert \eta \right\rvert ^a\left\lvert \xi -\eta \right\rvert ^{b}e \mid e\in E_0 \right\} , \; a,\;b\in \mathbb {Z}. \end{aligned}$$

As mentioned in the introduction, the nonlinearity in our problem has a skew-symmetric structure. It turns out that the following quantity plays a central role:

5.2 $$\begin{aligned} \sigma (\xi ,\eta ):=(\xi -\eta )\cdot \eta ^\perp , \quad \sigma (\xi ,\eta )=\sigma (\xi -\eta ,\eta )= -\sigma (\xi ,\xi -\eta ). \end{aligned}$$

#### Lemma 5.3

Let $e \in E_a^b$, and consider localizations $\chi ,\;\widetilde{\chi }$ as in (2.17). Then

$$\begin{aligned} S_\eta e \in E_{a}^b\cup E_{a+1}^{b-1},\qquad S_{\xi -\eta }e \in E_a^b\cup E_{a-1}^{b+1}, \end{aligned}$$

and we have the bounds

5.3 $$\begin{aligned} \left\lvert S_\eta e\right\rvert \chi (\xi ,\eta )&\lesssim (1+2^{k_2-k_1}2^{p_{\max }})\left\Vert e\chi \right\Vert _{L^\infty }, \nonumber \\ \left\lvert S_\eta e\right\rvert \widetilde{\chi }(\xi ,\eta )&\lesssim (1+2^{k_2-k_1}(2^{q_{\max }}+2^{p_{\max }}))\left\Vert e\widetilde{\chi }\right\Vert _{L^\infty }, \end{aligned}$$

and symmetrically

$$\begin{aligned} \left\lvert S_{\xi -\eta } e\right\rvert \chi (\xi ,\eta )&\lesssim (1+2^{k_1-k_2}2^{p_{\max }}) \left\Vert e\chi \right\Vert _{L^\infty }, \\ \left\lvert S_{\xi -\eta } e\right\rvert \widetilde{\chi }(\xi ,\eta )&\lesssim (1+2^{k_1-k_2}(2^{q_{\max }}+2^{p_{\max }})) \left\Vert e\widetilde{\chi }\right\Vert _{L^\infty }. \end{aligned}$$

#### Proof

By symmetry and the product rule it suffices to consider $S_\eta e$, with $e\in E$ and prove (5.3). We have four types of elementary multipliers in *E*. First observe that with $\sigma$ as in (5.2)

5.4 $$\begin{aligned}&S_\eta \Lambda (\eta )=0,  &   S_\eta \Lambda (\xi -\eta )=-\frac{\xi _2-\eta _2}{\left\lvert \xi -\eta \right\rvert ^3}\sigma (\xi ,\eta ). \end{aligned}$$

Thus $S_\eta \Lambda (\zeta )\in E_{1}^{-1}$ for $\zeta \in \{\xi ,\eta ,\xi -\eta \}$. Similarly with (5.4)

$$\begin{aligned}&S_\eta \sqrt{1-\Lambda ^2(\xi -\eta )})=\Lambda (\xi -\eta )\frac{(\xi -\eta )\cdot \eta ^\perp }{\left\lvert \xi -\eta \right\rvert \left\lvert \eta \right\rvert }\frac{\left\lvert \eta \right\rvert }{\left\lvert \xi -\eta \right\rvert },  &   S_\eta \sqrt{1-\Lambda ^2(\eta )})=0, \end{aligned}$$

which are also elements of $$ E_{1}^{-1}$$. Thus

$$\begin{aligned}&\left\lvert S_\eta \Lambda (\xi -\eta )\right\rvert \chi (\xi ,\eta )\lesssim 2^{k_2-k_1}2^{p_{\max }},  &   \left\lvert S_\eta \sqrt{1-\Lambda ^2(\xi -\eta )}\right\rvert \chi \lesssim 2^{k_2-k_1}2^{p_{\max }}. \end{aligned}$$

Next we have the following computations

5.5 $$\begin{aligned} S_\eta \left\lvert \eta \right\rvert&=\left\lvert \eta \right\rvert , \qquad \quad S_\eta \left\lvert \xi -\eta \right\rvert =-\frac{\eta \cdot (\xi -\eta )}{\left\lvert \xi -\eta \right\rvert },\\ S_\eta \sigma&=-\eta \cdot \xi ^\perp , \nonumber \quad S_\eta ((\xi -\eta )\cdot \eta )=\eta \cdot (\xi -\eta )-\left\lvert \eta \right\rvert ^2, \end{aligned}$$

With this we prove the claim for multipliers of the form $\frac{\zeta \cdot \theta ^\perp }{\left\lvert \zeta \right\rvert \left\lvert \theta \right\rvert }$ and $\frac{\zeta \cdot \theta }{\left\lvert \zeta \right\rvert \left\lvert \theta \right\rvert }$, where by symmetry it suffices to consider $\zeta =\xi -\eta , \, \theta =\eta$:

$$\begin{aligned}&S_\eta \Big (\frac{(\xi -\eta )\cdot \eta ^\perp }{\left\lvert \xi -\eta \right\rvert \left\lvert \eta \right\rvert }\Big ) =\frac{(\xi -\eta )\cdot \eta ^\perp }{\left\lvert \xi -\eta \right\rvert \left\lvert \eta \right\rvert }\frac{\eta \cdot (\xi -\eta )}{\left\lvert \eta \right\rvert \left\lvert \xi -\eta \right\rvert }\frac{\left\lvert \eta \right\rvert }{\left\lvert \xi -\eta \right\rvert }, \\&\quad S_{\eta }\Big (\frac{(\xi -\eta )\cdot \eta }{\left\lvert \xi -\eta \right\rvert \left\lvert \eta \right\rvert } \Big ) =\frac{-\left\lvert \eta \right\rvert }{\left\lvert \xi -\eta \right\rvert }\left( 1-\left( \frac{(\xi -\eta )\cdot \eta }{\left\lvert \xi -\eta \right\rvert \left\lvert \eta \right\rvert }\right) ^2\right) . \end{aligned}$$

These are elements of $E_{1}^{-1}$ and satisfy the bounds:

$$\begin{aligned}&\left\lvert S_\eta \Big (\frac{(\xi -\eta )\cdot \eta ^\perp }{\left\lvert \xi -\eta \right\rvert \left\lvert \eta \right\rvert }\Big )\right\rvert \lesssim 2^{k_2-k_1}2^{p_{\max }},  &   \left\lvert S_{\eta }\Big (\frac{(\xi -\eta )\cdot \eta }{\left\lvert \xi -\eta \right\rvert \left\lvert \eta \right\rvert } \Big )\right\rvert \lesssim 2^{k_2-k_1}. \end{aligned}$$

Since $1\in E$, we obtain, altogether for $e\in E_0$,

5.6 $$\begin{aligned}&\left\lvert S_\eta e\right\rvert \chi \lesssim 1+2^{k_2-k_1}2^{p_{\max }}. \end{aligned}$$

Finally let $e \in E_a^b$. Then $e=\left\lvert \eta \right\rvert ^a \left\lvert \xi -\eta \right\rvert ^{b}e_0$ for an $e_0 \in E_0$, and with (5.5) and for a suitable $e_1\in E_0$ we have

$$\begin{aligned} S_\eta e&=a\left\lvert \eta \right\rvert ^{a}\left\lvert \xi -\eta \right\rvert ^be_0+b\left\lvert \eta \right\rvert ^{a+1}\left\lvert \xi -\eta \right\rvert ^{b-1}e_1+\left\lvert \eta \right\rvert ^a\left\lvert \xi -\eta \right\rvert ^bS_\eta e_0, \end{aligned}$$

which is an element of $E_a^b\cup E_{a+1}^{b-1}$. The bound follows directly using the claim (5.5). An analogous computation gives the result for $S_{\xi -\eta }$. $\square$

#### Lemma 5.4

Let $\tilde{E}_a^b:=\left\{ \left\lvert \xi \right\rvert ^{a}\left\lvert \xi -\eta \right\rvert ^{b}e_0 \mid e_0 \in E_0 \right\}$ and $e\in E_0$. Then $W_\xi e \in \tilde{E}_1^{-1}$ and

$$\begin{aligned} \left\lvert W_\xi e\right\rvert \chi \lesssim 1+ 2^{k-k_1}2^{p_{\max }}. \end{aligned}$$

Moreover, if $e_{ab}\in \tilde{E}_a^b$ then $W_\xi e_{ab}\in \tilde{E}_{a}^{b}\cup \tilde{E}_{a+1}^{b-1}$, and

$$\begin{aligned} \left\lvert W_\xi e_{ab}\right\rvert \chi \lesssim (1+ 2^{k-k_1}2^{p_{\max }}) \left\Vert e_{ab}\chi \right\Vert _{L^\infty }. \end{aligned}$$

#### Proof

The claim follows by similar computations as in the lemma above. $\square$

As discussed in the introduction of the paper, the null-structure of the nonlinearity is encoded in the multipliers (2.6), (2.12) of the bilinear terms. More precisely, the relevant bounds for us are the following:

#### Lemma 5.5

Let $\mathfrak {m}\in \{\mathfrak {m}_0,\mathfrak {m}_\pm ^{\mu \nu }\}$, where $\mathfrak {m}_0$ is the multiplier in (2.12), $\mathfrak {m}_\pm ^{\mu \nu }$ one of the multipliers in (2.6). Then $\mathfrak {m}\in E_1^0\cup E_0^1$ and the following bound holds:

$$\begin{aligned}&\left\lvert \mathfrak {m}(\xi ,\eta )\right\rvert \chi \lesssim 2^{k}2^{p_{\max }},  &   \left\lvert \mathfrak {m}(\xi ,\eta )\right\rvert \widetilde{\chi }\lesssim 2^{k}2^{p_{\max }+q_{\max }} , \end{aligned}$$

where $\chi ,\, \widetilde{\chi }$ as in (2.17).

#### Proof

We prove the first bound and note that the second one follows analogously when additionally localizing in $q,q_1,q_2\in \mathbb {Z}^-$. Since

$$\begin{aligned} \left\lvert \mathfrak {m}_0\right\rvert \chi =\frac{1}{2} \frac{\left\lvert (\xi -\eta )\cdot \eta ^\perp \right\rvert }{\left\lvert \xi -\eta \right\rvert \left\lvert \eta \right\rvert }\left\lvert \left\lvert \xi -\eta \right\rvert -\left\lvert \eta \right\rvert \right\rvert \le \frac{1}{2} (2^{p_1}+2^{p_2})2^k, \end{aligned}$$

the claim is direct for $\mathfrak {m}_0$.

For $\mathfrak {m}^{\mu \nu }_\pm$ we will establish the following bound, which implies the claim

$$\begin{aligned} \left\lvert \mathfrak {m}^{\mu \nu }_\pm \right\rvert \chi \lesssim 2^{p_{\max }}\min \{2^{k}\max \{1+2^{k_1-k_2},1+2^{k_2-k_1}\},\max \{2^{k_1},2^{k_2}\}\}. \end{aligned}$$

To see this, we bound the additional terms in (2.6) separately. On the one hand, we have the direct bounds

$$\begin{aligned} \begin{aligned} \left\lvert \frac{\xi (\xi -\eta )^\perp }{\left\lvert \xi \right\rvert \left\lvert \xi -\eta \right\rvert }\Big ( \frac{\left\lvert \eta \right\rvert ^2-\left\lvert \xi -\eta \right\rvert ^2}{\left\lvert \eta \right\rvert } \Big )\right\rvert \chi&\lesssim (2^{p}+2^{p_1})2^{k}(1+2^{k_1-k_2}),\\ \left\lvert \frac{\xi (\xi -\eta )^\perp }{\left\lvert \xi \right\rvert \left\lvert \xi -\eta \right\rvert }(\left\lvert \xi -\eta \right\rvert +\left\lvert \eta \right\rvert )\right\rvert \chi&\lesssim (2^{p_1}+2^{p_2})2^{k_1}+(2^{p_1}+2^{p_2})2^{k_2}, \end{aligned} \end{aligned}$$

while we can alternatively use (5.2) to obtain that

$$\begin{aligned} \begin{aligned} \left\lvert \frac{\xi (\xi -\eta )^\perp }{\left\lvert \xi \right\rvert \left\lvert \xi -\eta \right\rvert }\Big ( \frac{\left\lvert \eta \right\rvert ^2-\left\lvert \xi -\eta \right\rvert ^2}{\left\lvert \eta \right\rvert } \Big )\right\rvert \chi&=\left\lvert \frac{\xi (\xi -\eta )^\perp }{\left\lvert \xi \right\rvert \left\lvert \xi -\eta \right\rvert }\left\lvert \eta \right\rvert +\frac{\xi \eta ^\perp }{\left\lvert \xi \right\rvert \left\lvert \eta \right\rvert }\left\lvert \xi -\eta \right\rvert \right\rvert \chi \\&\lesssim (2^{p}+2^{p_1})2^{k_2}+(2^p+2^{p_2})2^{k_1},\\ \left\lvert \frac{(\xi -\eta )^\perp \eta }{\left\lvert \xi -\eta \right\rvert \left\lvert \eta \right\rvert }\big (\left\lvert \xi -\eta \right\rvert +\left\lvert \eta \right\rvert \big )\right\rvert \chi&=\left\lvert \frac{\xi (\xi -\eta )^\perp }{\left\lvert \xi \right\rvert \left\lvert \xi -\eta \right\rvert }\left\lvert \xi \right\rvert \Big (\frac{\left\lvert \eta \right\rvert +\left\lvert \xi -\eta \right\rvert }{\left\lvert \eta \right\rvert }\Big )\right\rvert \chi \\&\lesssim (2^p+2^{p_1})2^{k}(1+2^{k_1-k_2}). \end{aligned} \end{aligned}$$

$$\square $$

For repeated integration by parts we also want to understand how the vector fields act on a multiplier $\mathfrak {m}$. For a set *A*, we let $\left\lvert \eta \right\rvert A=\left\{ \left\lvert \eta \right\rvert \cdot a \mid a\in A \right\}$.

#### Lemma 5.6

Let $\mathfrak {m}\in \{\mathfrak {m}_0, \mathfrak {m}^{\mu \nu }_\pm \}$ be one of the multiplies defined in (2.12), (2.6). Then for $N\ge 1$ there holds $S_\eta ^N\mathfrak {m}\in \bigcup _{i=0}^{N}E_{i}^{1-i}\cup E_{1+i}^{-i}$ and $W_\xi ^N\mathfrak {m}\in \bigcup _{i=0}^{N} \left\lvert \eta \right\rvert \tilde{E}_i^{-i}\cup \tilde{E}_i^{1-i}$. Moreover, the following bounds hold:

$$\begin{aligned}&|S_\eta ^N\mathfrak {m}|\chi \lesssim 2^{k_{\max }}[1+2^{k_2-k_1}2^{p_{\max }}]^N\chi ,&|W_{\xi }^N\mathfrak {m}|\chi \lesssim 2^{k_{\max }}[1+2^{k-k_1}2^{p_{\max }}]^N\chi . \end{aligned}$$

Analogous bounds hold true when localizing in $\widetilde{\chi }$.

#### Proof

By Lemma 5.5, there holds $\mathfrak {m}\in E_0^1 \cup E_1^0$ and thus, by Lemma 5.3, we obtain by repeatedly applying $S_\eta$

$$\begin{aligned} \left\lvert S_\eta ^N\mathfrak {m}\chi \right\rvert =2^{k_{\max }}(1+2^{k_2-k_1}(1+2^{p_2-p_1}))^N\chi . \end{aligned}$$

The second claim follows similarly from Lemma 5.4 by noting that $\mathfrak {m}\in \left\lvert \eta \right\rvert E_0\cup \tilde{E}_0^1$. $\square$

### Vector Fields and the Phases

Next we discuss how the vector field *S* acts on the phase $\Phi ^{\pm }_{\mu \nu }$. Recall from (2.5) the definition

5.7 $$\begin{aligned} \Phi ^{\mu \nu }_\pm (\xi ,\eta )=\pm \Lambda (\xi )-\mu \Lambda (\xi -\eta )-\nu \Lambda (\eta ), \end{aligned}$$

and note that by (5.4) we have that

$$\begin{aligned} S_\eta \Phi ^{\mu \nu }_\pm =\mu S_\eta \Lambda (\xi -\eta ). \end{aligned}$$

Without loss of generality we will thus only consider $\Phi (\xi ,\eta ):=\Phi _{+}^{++}(\xi ,\eta )$.

#### Lemma 5.7

Let $\sigma$ as in (5.2) and $\chi$ as in (2.17). Then $S_\eta \Phi \in E_{1}^{-1}$ and $S_{\xi -\eta }\Phi \in E_{-1}^{1}$ on the support of $\chi$ there holds that

$$\begin{aligned}&S_\eta \Phi (\xi ,\eta )= \frac{\xi _2-\eta _2}{\left\lvert \xi -\eta \right\rvert ^3}\sigma (\xi ,\eta ),  &   S_{\xi -\eta }\Phi (\xi ,\eta )=\frac{\eta _2}{\left\lvert \eta \right\rvert ^3}\sigma (\xi ,\eta ), \end{aligned}$$

and

$$\begin{aligned} \left\lvert S_\eta \Phi \right\rvert \chi \sim 2^{-2k_1}2^{p_1}\left\lvert \sigma (\xi ,\eta )\right\rvert \chi , \hspace{1cm} \left\lvert S_{\xi -\eta }\Phi \right\rvert \chi \sim 2^{-2k_2}2^{p_2}\left\lvert \sigma (\xi ,\eta )\right\rvert \chi . \end{aligned}$$

The analogous bounds hold for the $\widetilde{\chi }$ localizations.

#### Proof

With (5.7), the definition (5.2) of $\sigma$ and (5.4) that

$$\begin{aligned} S_\eta \Phi =-S_\eta \Lambda (\eta )-S_\eta \Lambda (\xi -\eta )=\frac{\xi _2-\eta _2}{\left\lvert \xi -\eta \right\rvert ^3} \sigma (\xi ,\eta ). \end{aligned}$$

Therefore, on the support of $\chi$ we have

$$\begin{aligned} \left\lvert S_\eta \Phi \right\rvert \chi \sim 2^{-2k_1}2^{p_1}\left\lvert \sigma (\xi ,\eta )\right\rvert \chi . \end{aligned}$$

Similarly by symmetry of *S* and $\sigma$, we have $S_{\xi -\eta }\Phi =-S_{\xi -\eta }\Lambda (\eta )=-\frac{\eta _2}{\left\lvert \eta \right\rvert ^3}\sigma (\xi ,\eta )$ and the size estimate follows directly. $\square$

Together with (5.2), this lemma provides an important insight: whenever on the support of $\chi$ we have a size gap between any pair of the parameters $p,p_j$, $j=1,2$, we have a lower bound for $\sigma$ and thus for $S_\eta \Phi$ resp. $S_{\xi -\eta }\Phi$.

However, this can be further refined when taking also the size of the phase function (with respect to the localizations in $\Lambda$) into account. The following proposition is a key ingredient of our analysis and tells us roughly speaking that either we have a lower bound on $\left\lvert \sigma \right\rvert$ (and thus integrate by parts along *S* with Lemma 5.10) or the phase is large (and one can employ normal forms as in Sect. 5.7).

#### Proposition 5.8

Let $\Phi \in \left\{ \Phi _{\pm }^{\mu \nu } \mid \mu ,\nu \in \{+,-\} \right\}$. Then either $\left\lvert \Phi \right\rvert \widetilde{\chi }\ge 2^{q_{\max }-10}$ or $\sigma$ satisfies the lower bound

$$\begin{aligned}&\left\lvert \sigma \right\rvert \widetilde{\chi }\gtrsim 2^{k_{\min }+k_{\max }}2^{p_{\max }+q_{\max }}. \end{aligned}$$

#### Proof

Let $q_\alpha =\max \{q,q_1,q_2\}$ and $q_\beta =\min \{q,q_1,q_2\}$, and denote correspondingly $p_{{\max }}=p_{\beta }$ and $p_{{\min }}=p_{\alpha }$. Moreover let

$$\begin{aligned} \Lambda _\alpha =\max \left\{ \left\lvert \Lambda (\zeta )\right\rvert \mid \zeta \in \{\xi ,\xi -\eta ,\eta \} \right\} , \qquad \Lambda _\beta =\min \left\{ \left\lvert \Lambda (\zeta )\right\rvert \mid \zeta \in \{\xi ,\xi -\eta ,\eta \} \right\} . \end{aligned}$$

Assume that $$\left\lvert \Phi \right\rvert \widetilde{\chi }<2^{q_\alpha -10}$$.

**Case A:** Assume we have a gap in *p*, i.e. $\left\lvert p_{\alpha }-p_{\beta }\right\rvert > 10$, then there holds $2^{p_{\alpha }}<2^{-10}$. Moreover, since $\Lambda ^2_\alpha +1-\Lambda ^2_\alpha =1$ implies $2^{2q_\alpha }\gtrsim 1-2^{2p_\alpha }\gtrsim 1-2^{-20}$, it follows that $2^{q_{\max }}=2^{q_\alpha }\sim 1$. Then it follows directly from (5.2)

$$\begin{aligned} \left\lvert \sigma \right\rvert \widetilde{\chi }&\gtrsim 2^{k_\alpha +k_\beta }|\Lambda _\alpha \sqrt{1-\Lambda _\beta ^2}-\sqrt{1-\Lambda _\alpha ^2}\Lambda _\beta | \gtrsim 2^{k_\alpha +k_\beta } 2^{p_{\beta }}\sim 2^{k_{\max }+k_{\min }}2^{p_{\max }}. \end{aligned}$$

**Case B:** $$\left\lvert p_{\alpha }-p_{\beta }\right\rvert \le 10$$.

We claim that $\frac{\Lambda _\beta }{\Lambda _\alpha }<\frac{2}{3}$. Otherwise, if $\Lambda _\alpha \le \frac{3}{2}\Lambda _\beta$,

$$\begin{aligned} \left\lvert \Phi \right\rvert \widetilde{\chi }=\left\lvert \Lambda (\xi )\pm \Lambda (\xi -\eta )\pm \Lambda (\eta )\right\rvert \ge \frac{1}{3}\Lambda _\alpha \ge 2^{q_\alpha -2}, \end{aligned}$$

which contradicts the assumption $\left\lvert \Phi \right\rvert \widetilde{\chi }\le 2^{q_{\alpha }-10}$. Hence there holds $\frac{\Lambda _\beta }{\Lambda _\alpha }<\frac{2}{3}$, which implies

$$\begin{aligned} \Lambda _\alpha \sqrt{1-\Lambda _\beta ^2}>\frac{3}{2}\Lambda _\beta \sqrt{1-\Lambda _\beta ^2}>\frac{3}{2}\Lambda _\beta \sqrt{1-\Lambda _\alpha ^2}. \end{aligned}$$

From this it follows with (5.2) that

$$\begin{aligned} \left\lvert \sigma \right\rvert \widetilde{\chi }&\gtrsim 2^{k_\alpha +k_\beta }\left\lvert \Lambda _\alpha \sqrt{1-\Lambda _\beta ^2}-\sqrt{1-\Lambda _\alpha ^2}\Lambda _\beta \right\rvert \\&\gtrsim 2^{k_\alpha +k_\beta }\Lambda _\alpha \sqrt{1-\Lambda _\beta ^2}\gtrsim 2^{k_{\min }+k_{\max }}2^{p_{\max }} 2^{q_{\max }}. \end{aligned}$$

$$\square $$

We also record some basic bounds for the action of *W* on the phases $\Phi$:

#### Lemma 5.9

For the vector field $W_\xi =\xi ^\perp \cdot \nabla _\xi$ and a phase $\Phi \in \left\{ \Phi _\pm ^{\mu \nu } \mid \mu ,\nu \in \{+,-\} \right\}$ as in (2.5) there holds

$$\begin{aligned} W_\xi \Phi _{\pm }^{\mu \nu }&=\mp \frac{\xi _2}{\left\lvert \xi \right\rvert }-\mu \frac{\xi _2-\eta _2}{\left\lvert \xi -\eta \right\rvert ^3}\xi \cdot (\xi -\eta )\\  &=\mp \sqrt{1-\Lambda ^2(\xi )} -\mu \sqrt{1-\Lambda ^2(\xi -\eta )}\frac{\xi \cdot (\xi -\eta )}{\left\lvert \xi \right\rvert \left\lvert \xi -\eta \right\rvert }\frac{\left\lvert \xi \right\rvert }{\left\lvert \xi -\eta \right\rvert },\\\left\lvert W_\xi \Phi _\pm ^{\mu \nu }\right\rvert \chi&\lesssim 2^p+2^{p_1}2^{k-k_1}. \end{aligned}$$

#### Proof

We compute with $\mu ,\nu \in \{+,-\}$

$$\begin{aligned} W_\xi \Phi _\pm ^{\mu \nu }&=\pm W_\xi \Lambda (\xi )-\mu W_\xi \Lambda (\xi -\eta )=\mp \frac{\xi _2}{\left\lvert \xi \right\rvert }-\mu \frac{(\xi _2-\eta _2)}{\left\lvert \xi -\eta \right\rvert }\frac{(\xi -\eta )\cdot \xi }{\left\lvert \xi -\eta \right\rvert \left\lvert \xi \right\rvert }\frac{\left\lvert \xi \right\rvert }{\left\lvert \xi -\eta \right\rvert }. \end{aligned}$$

Then it follows that

$$\begin{aligned} \left\lvert W_\xi \Phi _\pm ^{\mu \nu }\right\rvert \chi&\lesssim 2^p+2^{p_1}2^{k-k_1}. \end{aligned}$$

$$\square $$

### Integration by Parts in Bilinear Expressions

The main goal of this section is to establish bounds for repeated integration by parts along *S* in bilinear terms of the form

5.8 $$\begin{aligned} \mathcal {F}(\mathcal {Q}_{\mathfrak {m}}(f_1,f_2))(t,\xi )=\int _{\mathbb {R}^2}e^{it\Phi (\xi ,\eta )}\mathfrak {m}(\xi ,\eta )\widehat{f_1}(t,\xi -\eta )\widehat{f_2}(t,\eta ) d\eta , \end{aligned}$$

where $\mathfrak {m}\in \{\mathfrak {m}_0,\mathfrak {m}_\pm ^{\mu \nu }; \mu ,\nu \in \{+,-\}\}$ is one of the multipliers and $\Phi \in \{\Phi _\pm ^{\mu \nu };\mu ,\nu \in \{+,-\}\}$ is one of the phases associated with the Boussinesq resp. SQG equations, and $f_1,f_2$ are corresponding profiles.

#### Integration by Parts Along *S*.

We present next the main lemma for iterated integration by parts along $S_\eta$ and $S_{\xi -\eta }$. Let $f\in L^2$ and $N\in \mathbb {N}$, then

$$\begin{aligned} \left\Vert (1,S)^Nf\right\Vert _{L^2}:=\sum _{i=0}^N\left\Vert S^if\right\Vert _{L^2}. \end{aligned}$$

##### Lemma 5.10

1. Assume that $\left\lvert \sigma \right\rvert \chi \gtrsim L\gtrsim 2^{k_{\max }+k_{\min }+p_{\max }}$. Then for $N\in \mathbb {N}$ there holds:
  $$\begin{aligned} \left\Vert \mathcal {F}(\mathcal {Q}_{\mathfrak {m}\chi }(R_{l_1}f_1,R_{l_2}f_2))\right\Vert _{L^\infty }&\lesssim 2^{k_{\max }}[t^{-1}2^{2k_1}2^{-p_1}L^{-1}(1+2^{k_2-k_1}2^{l_1})]^N\\&\quad \cdot \left\Vert P_{k_1,p_1}R_{l_1}(1,S)^Nf_1\right\Vert _{L^2}\left\Vert P_{k_2,p_2}R_{l_2}(1,S)^Nf_2\right\Vert _{L^2}, \\ \left\Vert \mathcal {F}(\mathcal {Q}_{\mathfrak {m}\chi }(R_{l_1}f_1,R_{l_2}f_2))\right\Vert _{L^\infty }&\lesssim 2^{k_{\max }} [t^{-1}2^{2k_2}2^{-p_2}L^{-1}(1+2^{k_1-k_2}2^{l_2})]^N\\&\quad \cdot \left\Vert P_{k_1,p_1}R_{l_1}(1,S)^Nf_1\right\Vert _{L^2}\left\Vert P_{k_2,p_2}R_{l_2}(1,S)^Nf_2\right\Vert _{L^2}. \end{aligned}$$
2. Assume that $\left\lvert \sigma \right\rvert \widetilde{\chi }\gtrsim \tilde{L}\gtrsim 2^{k_{\max }+k_{\min }+p_{\max }+q_{\max }}$. Then for $N\in \mathbb {N}$ there holds:
  $$\begin{aligned}&\left\Vert \mathcal {F}(\mathcal {Q}_{\mathfrak {m}\widetilde{\chi }}(R_{l_1}f_1,R_{l_2}f_2))\right\Vert _{L^\infty } \\&\quad \lesssim 2^{k_{\max }}[t^{-1}(2^{k_2-k_1-p_1-q_1}+2^{2k_1}2^{-p_1}\tilde{L}^{-1}(1+2^{k_2-k_1}(2^{q_2-q_1}+2^{l_1})))]^N\\&\quad \quad \cdot \left\Vert P_{k_1,p_1,q_1}R_{l_1}(1,S)^Nf_1\right\Vert _{L^2}\left\Vert P_{k_2,p_2,q_2}R_{l_2}(1,S)^Nf_2\right\Vert _{L^2}, \\&\left\Vert \mathcal {F}(\mathcal {Q}_{\mathfrak {m}\widetilde{\chi }}(R_{l_1}f_1,R_{l_2}f_2))\right\Vert _{L^\infty } \\&\quad \lesssim 2^{k_{\max }}[t^{-1}(2^{k_1-k_2-p_2-q_2}+2^{2k_2}2^{-p_2}\tilde{L}^{-1}(1+2^{k_1-k_2}(2^{q_1-q_2}+2^{l_2})))]^N\\&\quad \quad \cdot \left\Vert P_{k_1,p_1,q_1}R_{l_1}(1,S)^Nf_1\right\Vert _{L^2}\left\Vert P_{k_2,p_2,q_2}R_{l_2}(1,S)^Nf_2\right\Vert _{L^2}. \end{aligned}$$

##### Proof

We start by proving the first bound in (1), noting that the second one follows by symmetry and the analogous bounds for $S_{\xi -\eta }$. Let $F=R_{l_1}f_1$ and $G=R_{l_2}f_2.$ With $e^{it\Phi }=S_\eta e^{it\Phi }\frac{1}{itS_\eta \Phi }$ and Lemma 5.2, integrating by parts once in $S_\eta$ yields

5.9 $$\begin{aligned} \begin{aligned}&\mathcal {F}(\mathcal {Q}_{\mathfrak {m}\chi }(F,G))\\  &\quad =\int _{\mathbb {R}^2}e^{it\Phi (\xi ,\eta )}\mathfrak {m}(\xi ,\eta )\chi (\xi ,\eta )\widehat{F}(\xi -\eta )\widehat{G}(\eta ) d\eta \\  &\quad =it^{-1}\int _{\mathbb {R}^2}e^{it\Phi }S_\eta \Big [\frac{1}{S_\eta \Phi }\mathfrak {m}(\xi ,\eta )\chi (\xi ,\eta )\widehat{F}(\xi -\eta )\widehat{G}(\eta )\Big ]d\eta \\  &\quad =it^{-1}\Big (\int _{\mathbb {R}^2}e^{it\Phi }S_{\eta }\Big (\frac{\mathfrak {m}\chi }{S_\eta \Phi }\Big )\widehat{F}(\xi -\eta )\widehat{G}(\eta )d\eta +\int _{\mathbb {R}^2}e^{it\Phi }\frac{\mathfrak {m}\chi }{S_\eta \Phi }\widehat{F}(\xi -\eta )S_\eta \widehat{G}(\eta )d\eta \\  &\hspace{1cm}+\int _{\mathbb {R}^2}e^{it\Phi }\frac{\mathfrak {m}\chi }{S_\eta \Phi }\left( \frac{\eta (\xi -\eta )}{\left\lvert \xi -\eta \right\rvert ^2}S_{\xi -\eta }\widehat{F}(\xi -\eta )-\frac{\eta (\xi -\eta )^\perp }{\left\lvert \xi -\eta \right\rvert ^2}W_{\xi -\eta }\widehat{F}(\xi -\eta )\right) \widehat{G}(\eta )d\eta \Big )\\  &\quad =it^{-1}\left( \mathcal {Q}_{S_\eta (\mathfrak {m}\chi (S_\eta \Phi )^{-1})}(F,G)+\mathcal {Q}_{\mathfrak {m}_1\chi (S_\eta \Phi )^{-1}}(SF,G)+\mathcal {Q}_{\mathfrak {m}_2\chi (S_\eta \Phi )^{-1}}(WF,G)\right. \\  &\qquad \qquad \quad \left. +\mathcal {Q}_{\mathfrak {m}\chi (S_\eta \Phi )^{-1}}(F,SG)\right) , \end{aligned} \end{aligned}$$

where $\mathfrak {m}_1,\mathfrak {m}_2\in E_2^{-1}\cup E_1^0$. We demonstrate the first bound in (1) for $N=1$. Observe that since $\left\lvert \sigma \right\rvert \chi \gtrsim L$, by Lemma 5.7

$$\begin{aligned} {\left\lvert S_\eta \Phi \right\rvert }^{-1}\chi \lesssim 2^{2k_1}2^{-p_1}L^{-1}. \end{aligned}$$

With $\mathfrak {m}\in E_0^1\cup E_1^0$ by Lemma 5.5

$$\begin{aligned} \left\lvert \mathcal {Q}_{\mathfrak {m}\chi (S_\eta \Phi )^{-1}}(F,SG)\right\rvert&\lesssim \int _{\mathbb {R}^2}\left\lvert \frac{\mathfrak {m}\chi }{S_\eta \Phi }\widehat{F}(\xi -\eta )S_\eta \widehat{G}(\eta )\right\rvert d\eta \\&\lesssim 2^{2k_1}2^{-p_1}L^{-1}\int _{\mathbb {R}^2}|\mathfrak {m}\chi (\xi ,\eta )\widehat{F}(\xi -\eta )S_\eta \widehat{G}(\eta )|d\eta \\&\lesssim 2^{2k_1}2^{-p_1}L^{-1}\left\Vert \mathfrak {m}\chi \right\Vert _{L^\infty }\int _{\mathbb {R}^2}\chi (\xi ,\eta )|\widehat{F}(\xi -\eta )S_\eta \widehat{G}(\eta )|d\eta , \end{aligned}$$

which leads to

$$\begin{aligned} \left\Vert \mathcal {Q}_{\mathfrak {m}\chi (S_\eta \Phi )^{-1}}\right\Vert _{L^\infty }\lesssim 2^{2k_1}2^{-p_1}L^{-1}\left\Vert \mathfrak {m}\chi \right\Vert _{L^\infty }\left\Vert P_{k_1,p_1}R_{l_1}f_1\right\Vert _{L^2}\left\Vert P_{k_2,p_2}R_{l_2}Sf_2\right\Vert _{L^2}. \end{aligned}$$

For the second term on the right-hand side of (5.9), we note that with

$$\begin{aligned} \mathfrak {m}_1=\mathfrak {m}\frac{\eta (\xi -\eta )}{\left\lvert \eta \right\rvert \left\lvert \xi -\eta \right\rvert }\frac{\left\lvert \eta \right\rvert }{\left\lvert \xi -\eta \right\rvert }, \quad \mathfrak {m}_2=-\mathfrak {m}\frac{\eta (\xi -\eta )^\perp }{\left\lvert \eta \right\rvert \left\lvert \xi -\eta \right\rvert }\frac{\left\lvert \eta \right\rvert }{\left\lvert \xi -\eta \right\rvert },\quad \mathfrak {m}_1,\mathfrak {m}_2\in E_2^{-1}\cup E_1^0, \end{aligned}$$

$$\begin{aligned}&\left\lvert \mathcal {Q}_{\mathfrak {m}_1\chi (S_\eta \Phi )^{-1}}(SF,G)+\mathcal {Q}_{\mathfrak {m}_2\chi (S_\eta \Phi )^{-1}}(WF,G)\right\rvert \\&\quad \lesssim \int _{\mathbb {R}^2}\left\lvert \frac{1}{S_\eta \Phi }\left[ \mathfrak {m}_1\chi S_{\xi -\eta }\widehat{F}(\xi -\eta )+\mathfrak {m}_2\chi W_{\xi -\eta }\widehat{F}(\xi -\eta )\right] \widehat{G}(\eta )\right\rvert d\eta \\&\quad \lesssim 2^{2k_1}2^{-p_1}L^{-1}2^{k_2-k_1}\int _{\mathbb {R}^2}\left\lvert \mathfrak {m}\chi (\xi ,\eta )\left[ S_{\xi -\eta }\widehat{F}(\xi -\eta )+W_{\xi -\eta }\widehat{F}(\xi -\eta )\right] \widehat{G}(\eta ) \right\rvert d\eta . \end{aligned}$$

Altogether, using Lemma 2.3(3) we have

$$\begin{aligned}&\left\| \mathcal {Q}_{\mathfrak {m}_1\chi (S_\eta \Phi )^{-1}}(SF,G)\right\| _{L^\infty }+\left\| \mathcal {Q}_{\mathfrak {m}_2\chi (S_\eta \Phi )^{-1}}(WF,G)\right\| _{L^\infty }\\  &\qquad \qquad \qquad \lesssim 2^{2k_1}2^{-p_1}L^{-1}2^{k_2-k_1}\left\| \mathfrak {m}\chi \right\| _{L^\infty }\left( \left\| P_{k_1,p_1}R_{l_1}Sf_1\right\| _{L^2}\right. \\  &\qquad \qquad \qquad \qquad \quad \qquad \left. +2^{l_1}\left\| P_{k_1,p_1}R_{l_1}f_1\right\| _{L^2}\right) \left\| P_{k_2,p_2}R_{l_2}f_2\right\| _{L^2}. \end{aligned}$$

Finally we estimate the first term on the right-hand side of (5.9), which can also be broken down in two parts:

5.10 $$\begin{aligned} \begin{aligned} \mathcal {Q}_{S_\eta (\mathfrak {m}\chi (S_\eta \Phi )^{-1})}(F,G)&=\int _{\mathbb {R}^2}e^{it\Phi }\frac{S_\eta (\mathfrak {m}\chi )}{S_\eta \Phi }\widehat{F}(\xi -\eta )\widehat{G}(\eta )d\eta \\&\quad - \int _{\mathbb {R}^2}e^{it\Phi }\frac{\mathfrak {m}\chi S^{2}_\eta \Phi }{(S_\eta \Phi )^2}\widehat{F}(\xi -\eta )\widehat{G}(\eta )d\eta \\&=:\mathcal {Q}_{1}(\xi ,t)+\mathcal {Q}_{2}(\xi ,t). \end{aligned} \end{aligned}$$

For the second term on the right-hand side above we obtain, using Lemma 5.3 on $\Phi \in E_0$,

$$\begin{aligned} \left\lvert \mathcal {Q}_{2}(\xi ,t)\right\rvert&\lesssim \int _{\mathbb {R}^2}\left\lvert \frac{\mathfrak {m}\chi S^{2}_\eta \Phi }{(S_\eta \Phi )^2}\widehat{F}(\xi -\eta )\widehat{G}(\eta )\right\rvert d\eta \\  &\lesssim 2^{k_{\max }}(1+2^{k_2-k_1}2^{p_{\max }})2^{2k_1}2^{-p_1}L^{-1}\int _{\mathbb {R}^2}|\chi (\xi ,\eta )\widehat{F}(\xi -\eta )\widehat{G}(\eta )|d\eta . \end{aligned}$$

Now we handle $\mathcal {Q}_{1}(\xi ,t)$. Recalling the definition (2.17) of $\chi$, we have

$$\begin{aligned} S_\eta \chi (\xi ,\eta )=\varphi _{k,p}(\xi )[S_\eta (\varphi _{k_1,p_1}(\xi -\eta ))\varphi _{k_2,p_2}(\eta )+\varphi _{k_1,p_1}(\xi -\eta )S_\eta (\varphi _{k_2,p_2}(\eta ))]. \end{aligned}$$

Using Lemma 5.3 we find

$$\begin{aligned} S_\eta (\varphi _{k_1,p_1}(\xi -\eta ))&=-2^{-k_1}\frac{\eta (\xi -\eta )}{\left\lvert \xi -\eta \right\rvert }\overline{\varphi }^1_{k_1}(\xi -\eta )\varphi _{p_1}(\xi -\eta )\\&\quad +2^{-p_1}\frac{\eta (\xi -\eta )^\perp }{\left\lvert \xi -\eta \right\rvert ^2}\Lambda (\xi -\eta ){\varphi }_{k_1}(\xi -\eta )\overline{\varphi }^2_{p_1}(\xi -\eta ), \end{aligned}$$

where $\overline{\varphi }^{i}$, $i=1,2$ are functions with similar support properties as $\varphi$ (see also Remark 2.2). By abusing the notation slightly, we obtain similarly that $S_\eta \varphi _{k_2,p_2}(\eta )=2^{-k_2}\left\lvert \eta \right\rvert \overline{\varphi }_{k_2,p_2}(\eta )$. Altogether this gives

$$\begin{aligned} \left\lvert S_\eta \chi \right\rvert \lesssim (1+2^{k_2-k_1}(1+2^{-p_1}))\chi . \end{aligned}$$

This, together with Lemma 5.6 implies the following bound on $\mathcal {Q}_{1}^{1}(\xi ,t)$:

$$\begin{aligned} \left\lvert \mathcal {Q}_{1}(\xi ,t)\right\rvert&\lesssim \int _{\mathbb {R}^2} |S_\eta \Phi |^{-1}|(S_\eta \mathfrak {m}\chi +\mathfrak {m}S_\eta \chi )\widehat{F}(\xi -\eta )\widehat{G}(\eta )|d\eta \\&\lesssim 2^{k_{\max }}2^{2k_1}2^{-p_1}L^{-1}(1+2^{k_2-k_1}2^{-p_1})\int _{\mathbb {R}^2}|\chi \widehat{F}(\xi -\eta )\widehat{G}(\eta ) |d\eta . \end{aligned}$$

Hence with (5.10), $\mathcal {Q}_{S_\eta (\mathfrak {m}\chi (S_\eta \Phi )^{-1})}(F,G)$ satisfies the bound

$$\begin{aligned}&\left\Vert \mathcal {Q}_{S_\eta (\mathfrak {m}\chi (S_\eta \Phi )^{-1})}(F,G)\right\Vert _{L^\infty } \\&\quad \lesssim 2^{k_{\max }}2^{2k_1}2^{-p_1}L^{-1}(1+2^{k_2-k_1}2^{-p_1})\left\Vert P_{k_1,p_1}R_{l_1}f_1\right\Vert _{L^2}\left\Vert P_{k_2,p_2}R_{l_2}f_2\right\Vert _{L^2}. \end{aligned}$$

Finally, since $l_1+p_1\ge 0$ and $l_1\ge 0$, and $\mathfrak {m}\in E_0^1\cup E_1^0$ we obtain, from (5.9),

$$\begin{aligned} \left\| \mathcal {F}(\mathcal {Q}_{\mathfrak {m}\chi }(F,G))\right\| _{L^\infty }&\lesssim 2^{k_{\max }}t^{-1}2^{2k_1-p_1}L^{-1}[1+2^{k_2-k_1}(1+2^{l_1})+2^{k_2-k_1}2^{-p_1}]\\  &\qquad \qquad \cdot \left\| P_{k_1,p_1}(1,S)F\right\| _{L^2}\left\| P_{k_2,p_2}(1,S)G\right\| _{L^2}\\  &\lesssim 2^{k_{\max }}t^{-1}2^{2k_1-p_1}L^{-1}(1+2^{k_2-k_1}2^{l_1})\\  &\qquad \qquad \cdot \left\| P_{k_1,p_1}(1,S)F\right\| _{L^2}\left\| P_{k_2,p_2}(1,S)G\right\| _{L^2}. \end{aligned}$$

For $N\ge 2$ we proceed iteratively from (5.9), where we observe that the multipliers obtained due to integration by parts are in the admissible classes defined in Sect. 5.2. Thus, Lemmas 5.3, 5.6 and 5.7 can be applied iteratively.

As for the claim (2), the proof follows similarly using the bounds with the $\widetilde{\chi }$ localizations in Lemmas 5.3 and 5.6. The only difference arises when the vector field *S* falls on $\widetilde{\chi }(\xi ,\eta )$ (see the term $\mathcal {Q}_1$ in (5.10) for the first iteration). Here, using the fact that $S_\eta \Lambda (\eta )=0$ and $S_\eta \Lambda (\xi -\eta )=-S_\eta \Phi$ we obtain

$$\begin{aligned}&\frac{ {S_\eta \widetilde{\chi }}}{{S_\eta \Phi }}\\  &=\frac{1}{S_\eta \Phi } \left( 2^{-k_1}\frac{\eta (\xi -\eta )}{\left\lvert \eta \right\rvert \left\lvert \xi -\eta \right\rvert }\left\lvert \eta \right\rvert +2^{-p_1}\Lambda (\xi -\eta )\frac{(\xi -\eta )\eta ^\perp }{\left\lvert \xi -\eta \right\rvert \left\lvert \eta \right\rvert }\frac{\left\lvert \eta \right\rvert }{\left\lvert \xi -\eta \right\rvert }+2^{-k_2}\left\lvert \eta \right\rvert \right) {\overline{\widetilde{\chi }}^1}+2^{-q_1} {\overline{\widetilde{\chi }}}^2, \end{aligned}$$

where $\overline{\widetilde{\chi }}^1,\overline{\widetilde{\chi }}^2$ have similar support properties as $\widetilde{\chi }$. The arising multipliers are again in the admissible class defined in Sect. 5.2 and the iteration follows as above. $\square$

#### Integration by Parts Along *D*.

We also present a result on a zero-homogeneous horizontal derivative that will be useful in the proof of Proposition 8.2, see **Case B.2(b)**. Define that

$$\begin{aligned}&D_{\eta }:=\left\lvert \eta \right\rvert \partial _{\eta _1}=\Lambda (\eta )S_\eta -\sqrt{1-\Lambda ^2(\eta )} W_\eta ,  &   D_{\xi -\eta }:=\left\lvert \xi -\eta \right\rvert \partial _{\eta _1}. \end{aligned}$$

##### Lemma 5.11

Assume that $\left\lvert D_\eta \Phi \right\rvert \chi \gtrsim L$. Then for $N\in \mathbb {N}$

$$\begin{aligned} \left\Vert \mathcal {F}(\mathcal {Q}_{\mathfrak {m}\chi }(R_{l_1}f_1,R_{l_2}f_2))\right\Vert _{L^\infty }&\lesssim 2^{k_{\max }}[t^{-1}L^{-1}(2^{l_1+p_1}+2^{l_2+p_2})]^N\\&\quad \cdot \left\Vert P_{k_1,p_1}R_{l_1}(1,S)^Nf_1\right\Vert _{L^2}\left\Vert P_{k_2,p_2}R_{l_2}(1,S)^Nf_2\right\Vert _{L^2}. \end{aligned}$$

##### Proof

The proof follows the same scheme as the proof of Lemma 5.10 with $e^{it\Phi }=(it)^{-1}\frac{D_\eta e^{it\Phi }}{D_\eta \Phi }$, hence we just record the necessary computations to proceed as above.

$$\begin{aligned}&D_\eta (\Lambda (\eta ))=\frac{\eta _2^2}{\left\lvert \eta \right\rvert ^2}=1-\Lambda ^2(\eta ), \hspace{0.5cm} D_\eta (\sqrt{1-\Lambda ^2(\eta )})=-\Lambda (\eta )\sqrt{1-\Lambda ^2(\eta )},\\  &D_\eta (\Lambda (\xi -\eta ))=-\frac{\left\lvert \eta \right\rvert }{\left\lvert \xi -\eta \right\rvert }(1-\Lambda ^2(\xi -\eta )), \\  &D_\eta (\sqrt{1-\Lambda ^2(\xi -\eta )})=\frac{\left\lvert \eta \right\rvert }{\left\lvert \xi -\eta \right\rvert }\Lambda (\xi -\eta )\sqrt{1-\Lambda ^2(\xi -\eta )},\\  &D_\eta \left\lvert \eta \right\rvert =\left\lvert \eta \right\rvert \Lambda (\eta ), \hspace{0.5cm} D_\eta \left\lvert \xi -\eta \right\rvert =-\left\lvert \eta \right\rvert \Lambda (\xi -\eta ), \hspace{0.5cm} D_\eta =\frac{\left\lvert \eta \right\rvert }{\left\lvert \xi -\eta \right\rvert }D_{\xi -\eta }. \end{aligned}$$

With these computations and $\overline{\varphi }^1,\overline{\varphi }^2$ functions with similar support properties as $\varphi$, we have

$$\begin{aligned} D_\eta \chi (\xi ,\eta )&= \Big (-2^{-k_1}\left\lvert \eta \right\rvert \Lambda (\xi -\eta )\\  &\qquad +2^{-p_1}\frac{\left\lvert \eta \right\rvert }{\left\lvert \xi -\eta \right\rvert }\Lambda (\xi -\eta )\sqrt{1-\Lambda ^2(\xi -\eta )} \Big )\varphi _{k,p,q}(\xi )\overline{\varphi }^1_{k_1,p_1}(\xi -\eta )\varphi _{k_2,p_2}(\eta )\\  &\qquad +\left( 2^{-k_2}\left\lvert \eta \right\rvert \Lambda (\eta )-2^{-p_2}\Lambda (\eta )\sqrt{1-\Lambda ^2(\eta )}\right) \varphi _{k,p}(\xi )\varphi _{k_1,p_1}(\xi -\eta )\overline{\varphi }^2_{k_2,p_2}(\eta ). \end{aligned}$$

Together with the Bernstein property Proposition 2.3(3), this implies that

$$\begin{aligned}&D_\eta \mathcal {F}(P_{k_1,p_1}R_{l_1}F_1)(\xi -\eta )\\&\qquad \sim 2^{k_2-k_1}[\Lambda (\xi -\eta )\mathcal {F}(P_{k_1,p_1}R_{l_1}(1,S)F_1)(\xi -\eta )+2^{l_1+p_1}\mathcal {F}(P_{k_1,p_1}R_{l_1}F_1)(\xi -\eta )],\\&D_\eta \mathcal {F}(P_{k_2,p_2}R_{l_2}F_2)(\eta )\sim \Lambda (\eta )\mathcal {F}(P_{k_2,p_2}R_{l_2}(1,S)F_2)(\eta )+2^{l_2+p_2}\mathcal {F}(P_{k_2,p_2}R_{l_2}F_2)(\eta ). \end{aligned}$$

Moreover, in order to control $D_\eta ^M\Phi$ we compute that

$$\begin{aligned}&\left\lvert D_\eta ^M\Lambda (\xi )\right\rvert =0,\hspace{0.5cm} \left\lvert D_\eta ^M\Lambda (\eta )\right\rvert \lesssim 1-\Lambda ^2(\eta ),\\  &\left\lvert D_\eta ^M\Lambda (\xi -\eta )\right\rvert \lesssim \frac{\left\lvert \eta \right\rvert ^M}{\left\lvert \xi -\eta \right\rvert ^M}(1-\Lambda ^2(\xi -\eta )). \end{aligned}$$

$$\square $$

#### Towards Finite Speed of Propagation

In the proof of Proposition 8.1, where we bound the *X*-norm in the case that the parameter *l* is large, we also need to understand how the vector field $W_\xi$ acts on bilinear expressions (5.8).

##### Lemma 5.12

Let $\mathcal {Q}_\mathfrak {m}$ be a bilinear expression as in (5.8). Then for $N\in \mathbb {N}$ there holds:

$$\begin{aligned}&\left\| R_l \mathcal {Q}_\mathfrak {m}(f_1,f_2)\right\| _{L^2}\\  &\quad \lesssim 2^{k_{\min }}2^{k+p_{\max }}2^{-Nl}[t(2^p+2^{k-k_1}2^{p_1})+2^{-p} +2^{k-k_1+l_1}]^N\left\| (1,S)^2f_1\right\| _{L^2}\left\| f_2\right\| _{L^2}\\  &\qquad +2^{k_{\min }}2^{k+p_{\max }}2^{-3l}\left\| S^3f_1\right\| _{L^2}\left\| f_2\right\| _{L^2}. \end{aligned}$$

The analogous bound holds with the roles of $f_1$ and $f_2$ (and their respective localizations) interchanged.

##### Proof

The core of the proof is the Bernstein property for the vector field *W* from Proposition 2.3(3):

$$\begin{aligned} \left\Vert R_l \mathcal {Q}_\mathfrak {m}(f_1,f_2)\right\Vert _{L^2}\lesssim 2^{-l}\left\Vert W_{\xi }\mathcal {Q}_{\mathfrak {m}}(f_1,f_2)\right\Vert _{L^2}. \end{aligned}$$

By changing variables we can assume w.l.o.g. that $k_2\le k_1$. We begin by proving that

5.11 $$\begin{aligned} W_\xi R_l \mathcal {F}(\mathcal {Q}_{\mathfrak {m}}(f_1,f_2))&=\mathcal {F}(\mathcal {Q}_{\mathfrak {m}_1^{(1)}}(f_1,f_2))-\mathcal {F}(\mathcal {Q}_{\mathfrak {m}_2^{(1)}}(Sf_1,f_2))\nonumber \\&\quad +\mathcal {F}(\mathcal {Q}_{\mathfrak {m}_3^{(1)}}(Wf_1,f_2)), \end{aligned}$$

where $\mathfrak {m}_i^{(1)} \in \left\lvert \eta \right\rvert \tilde{E}_a^b\cup \tilde{E}_c^d$, for some $a,b,c,d\in \mathbb {Z}$ for $i=1,2,3$. We compute, using Lemma 5.2,

$$\begin{aligned} W_\xi \mathcal {F}(\mathcal {Q}_{\mathfrak {m}}(f_1,f_2))&=\int _{\mathbb {R}^2} W_\xi (e^{-it\Phi }\mathfrak {m})\widehat{f_1}(\xi -\eta )\widehat{f_2}(\eta )d\eta \\&\quad +\int _{\mathbb {R}^2} e^{-it\Phi }\mathfrak {m}W_\xi \widehat{f_1}(\xi -\eta )\widehat{f_2}(\eta )d\eta \\&=\int _{\mathbb {R}^2}W_\xi (e^{-it\Phi }\mathfrak {m})\widehat{f_1}(\xi -\eta )\widehat{f_2}(\eta )d\eta \\&\quad +\int _{\mathbb {R}^2} e^{-it\Phi }\mathfrak {m}\frac{(\xi -\eta )\xi }{\left\lvert \xi -\eta \right\rvert ^2}W_{\xi -\eta }\widehat{ f_1}(\xi -\eta )\widehat{f_2}(\eta )d\eta \\&\quad -\int _{\mathbb {R}^2} e^{-it\Phi }\mathfrak {m}\frac{(\xi -\eta )^\perp \xi }{\left\lvert \xi -\eta \right\rvert ^2}S_{\xi -\eta } \widehat{f_1}(\xi -\eta )\widehat{f_2}(\eta )d\eta \\&=\mathcal {F}(\mathcal {Q}_{\mathfrak {m}^{(1)}_1}(f_1,f_2))-\mathcal {F}(\mathcal {Q}_{\mathfrak {m}^{(1)}_2}(Sf_1,f_2))+\mathcal {F}(\mathcal {Q}_{\mathfrak {m}^{(1)}_3}(Wf_1,f_2)), \end{aligned}$$

where

$$\begin{aligned}&\mathfrak {m}_1^{(1)}=-itW_\xi \Phi \mathfrak {m}+ W_\xi \mathfrak {m},  &   \mathfrak {m}_2^{(1)}=\frac{(\xi -\eta )^\perp \xi }{\left\lvert \xi -\eta \right\rvert ^2}\mathfrak {m},  &   \mathfrak {m}_3^{(1)}=\frac{(\xi -\eta )\xi }{\left\lvert \xi -\eta \right\rvert ^2}\mathfrak {m}. \end{aligned}$$

Using Lemmas 5.6, 5.9, the multipliers satisfy

$$\begin{aligned} \begin{aligned} \Vert \mathfrak {m}_1^{(1)}\chi \Vert _{L^\infty }&\lesssim 2^m(2^p+2^{k-k_1}2^{p_1})\left\lvert \mathfrak {m}\right\rvert +2^k(1+2^{k-k_1}(1+2^{p-p_1})\\&\lesssim (2^m(2^p+2^{k-k_1}2^{p_1})+2^{-p}+2^{k-k_1-p}(1+2^{p-p_1}))\left\Vert \mathfrak {m}\chi \right\Vert _{L^\infty },\\ \Vert \mathfrak {m}_2^{(1)}\chi \Vert _{L^\infty }&\lesssim 2^{k-k_1}\left\Vert \mathfrak {m}\chi \right\Vert _{L^\infty },\\ \Vert \mathfrak {m}_3^{(1)}\chi \Vert _{L^\infty }&\lesssim 2^{k-k_1}\left\Vert \mathfrak {m}\chi \right\Vert _{L^\infty }. \end{aligned} \end{aligned}$$

Hence from (5.11), the following bound holds:

$$\begin{aligned}&\left\| W_\xi R_l\mathcal {F}(\mathcal {Q}_{\mathfrak {m}}(f_1,f_2))\right\| _{L^2}\\  &\quad \lesssim 2^{k_{\min }}2^{k+p_{\max }}\Big [(t(2^p+2^{k-k_1}2^{p_1})+2^{-p} + 2^{k-k_1}(2^{-p_1}+2^{l_1}))\left\| f_1\right\| _{L^2}\left\| f_2\right\| _{L^2} \\  &\qquad \qquad \qquad \qquad \quad +\left\| Sf_1\right\| _{L^2}\left\| f_2\right\| _{L^2}\Big ], \end{aligned}$$

Iterating this process, we see that taking $W^j\mathcal {Q}_{\mathfrak {m}}(f_1,f_2)$ generates $3^j$ bilinear expressions. Inductively it follows that

$$\begin{aligned} W\mathcal {Q}_{\mathfrak {m}^{(j)}}(f_1,f_2)= \mathcal {Q}_{\mathfrak {m}^{(j+1)}_1}(f_1,f_2)+\mathcal {Q}_{\mathfrak {m}^{(j+1)}_2}(Sf_1,f_2)+\mathcal {Q}_{\mathfrak {m}^{(j+1)}_3}(Wf_1,f_2), \end{aligned}$$

where the multipliers are

$$\begin{aligned}&\mathfrak {m}_1^{(j+1)}=-itW_\xi \Phi \mathfrak {m}^{(j)}+W_\xi \mathfrak {m}^{(j)},\\  &\mathfrak {m}_2^{(j+1)}=\frac{(\xi -\eta )^\perp \xi }{\left\lvert \xi -\eta \right\rvert ^2}\mathfrak {m}^{(j)}, \qquad \mathfrak {m}_3^{(j+1)}=\frac{(\xi -\eta )\xi }{\left\lvert \xi -\eta \right\rvert ^2}\mathfrak {m}^{(j)}. \end{aligned}$$

Furthermore, with Lemmas 5.6, 5.9 and 5.12, we see by induction that the multipliers satisfy the following bounds:

$$\begin{aligned}&\Vert {\mathfrak {m}_1^{(j+1)}\chi }\Vert _{L^{\infty }}\lesssim (2^m(2^p+2^{k-k_1}2^{p_1})+2^{-p}+2^{k-k_1-p_1})^j2^{k+p_{\max }}, \\&\Vert {\mathfrak {m}_2^{(j+1)}\chi }\Vert _{L^{\infty }}\lesssim \Vert {\mathfrak {m}^{(j)}\chi }\Vert _{L^{\infty }}, \qquad \Vert {\mathfrak {m}_3^{(j+1)}\chi }\Vert _{L^{\infty }}\lesssim \Vert {\mathfrak {m}^{(j)}\chi }\Vert _{L^{\infty }}. \end{aligned}$$

At each step we have a bound on the $L^2$ norm:

$$\begin{aligned} \begin{aligned} \left\Vert W\mathcal {Q}_{\mathfrak {m}^{(j)}}(f_1,f_2)\right\Vert _{L^2}&\lesssim \Vert \mathcal {Q}_{\mathfrak {m}^{(j+1)}_1}(f_1,f_2)\Vert _{L^2}+\Vert \mathcal {Q}_{\mathfrak {m}^{(j+1)}_2}(Sf_1,f_2)\Vert _{L^2}+\Vert \mathcal {Q}_{\mathfrak {m}^{(j+1)}_3}(Wf_1,f_2)\Vert _{L^2}\\&\lesssim 2^{k+p_{\max }}(t(2^p+2^{k-k_1}2^{p_1})+2^{-p}+2^{k-k_1-p_1})^j\left\Vert f_1\right\Vert _{L^2}\left\Vert f_2\right\Vert _{L^2}\\&\quad +\Vert {\mathfrak {m}^{(j)}\chi }\Vert _{L^{\infty }}\left\Vert Sf_1\right\Vert _{L^2}\left\Vert f_2\right\Vert _{L^2}+2^{l_1}\Vert {\mathfrak {m}^{(j)}\chi }\Vert _{L^{\infty }}\left\Vert f_1\right\Vert _{L^2}\left\Vert f_2\right\Vert _{L^2}. \end{aligned} \end{aligned}$$

Observe that when the vector field $W_\xi$ produces an $Sf_1$ term (multipliers of the type $\mathfrak {m}_2^{(j+1)}$), we have no additional losses in $m,p,l_1$. Hence, for such terms we stop after three iterations, while for the rest we can continue the iteration as above. Altogether with the Bernstein property we obtain

$$\begin{aligned}&\left\| R_l \mathcal {Q}_\mathfrak {m}(f_1,f_2)\right\| _{L^2}\\  &\lesssim 2^{k_{\min }}2^{k+p_{\max }}2^{-Nl}\big [t(2^p+2^{k-k_1}2^{p_1})+2^{-p} +2^{k-k_1+l_1}\big ]^N\left\| S^{\le 2}f_1\right\| _{L^2}\left\| f_2\right\| _{L^2}\\  &\quad +2^{k_{\min }}2^{k+p_{\max }}2^{-3l}\left\| S^3f_1\right\| _{L^2}\left\| f_2\right\| _{L^2}. \end{aligned}$$

$$\square $$

### Case Organisation and a Reduction Lemma

**Fig. 1:**
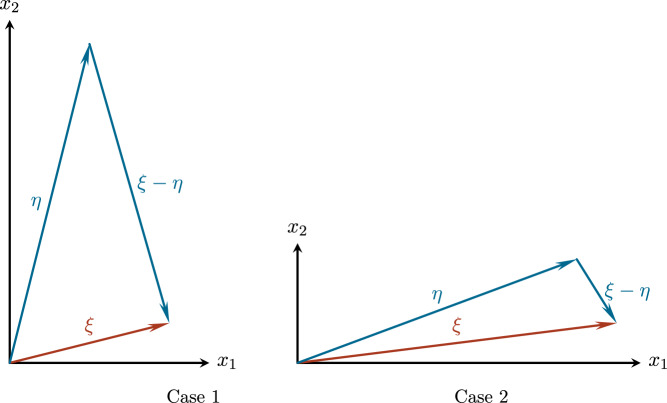
Two possible scenarios from Lemma 5.13 in Cartesian coordinates

The following lemma gives an overview of the relation between different localisation parameters depending on their relative size to one another (see e.g., Fig. 1).

#### Lemma 5.13

Assume $p_{\min }=p\le p_{\max }-10$. Then on the support of $\chi$ the following configurations are possible:

1. $$\left\lvert k_1-k_2\right\rvert \le 4$$ then $$p\le \min \{p_1,p_2\}-3$$ and $$\left\lvert p_1-p_2\right\rvert \le 5$$,
2. $k_1<k_2-4$, then $\left\lvert k-k_2\right\rvert \le 2$ and $p_{\max }=p_1$; moreover there holds either $p\le p_2-10\le p_1-12$ and $p_2+k_2-2\le p_1+k_1\le p_2+k_2+2$, or $\left\lvert p-p_2\right\rvert \le 10$ and ${p_1+k_1}\le p_2+k_2+3$,
3. $k_2<k_1-4$, then $\left\lvert k-k_1\right\rvert \le 2$ and $p_{\max }=p_2$; moreover there holds either $p\le p_1-10\le p_2-12$ and $p_2+k_2-2\le p_1+k_1\le p_2+k_2+2$, or $\left\lvert p-p_1\right\rvert \le 2$ and $p_2+k_2\le p_1+k_1+3$.

#### Remark 5.14

1. The analogous result holds with the roles of *p*, $p_i$ for $i=1,2$ interchanged.
2. The analogous result holds for the localization parameters $q,q_i$ for $i=1,2$ in the “gap in *q*" case, that is when $q_{\min }\le q_{\max }-10$.
3. In the following we will use the notation $\ll$, $\sim$, $\lesssim$ that includes both multiplicative bounds on the dyadic scale $2^n$ and additive constants at the level of the parameter $n\in \mathbb {Z}$. For example $2^p\ll 2^{p_1}$ implies there exist constants $C,C_1>0$ such that $2^{p}\le C_12^{p_1-C}$. Similarly, $2^{p}\sim 1$ (equivalently $p\sim 0$) implies $-C<p\le 0$ for a constant $C\in \mathbb {N}$.

#### Proof of Lemma 5.13

We prove each case separately.

*(1)* Observe that by triangle inequality and $\xi =(\xi -\eta )+\eta$ there holds $2^{k}\le 2^{k_1}+2^{k_2}\le 2^{k_2+5}$. Assume w.l.o.g. that $p_2=p_{\max }$. Then if $p_1<p+3$ there holds that $p_1<p_2-7$, and since $\eta _2=\xi _2-(\xi _2-\eta _2)$ we obtain

$$\begin{aligned} 2^{p_2+k_2}\le 2^{p+k}+2^{p_1+k_1}< 2^{p_2-10+k_2+5}+2^{p_2-7+4+k_2}\le 2^{p_2+k_2}(2^{-5}+2^{-3}), \end{aligned}$$

which leads to a contradiction. Hence $p_1\ge p+3$. Moreover, since $\eta _2=\xi _2-(\xi _2-\eta _2)$ and thus

$$\begin{aligned} 2^{p_2+k_2}\le 2^{p+k}+2^{p_1+k_1}\le 2^{p_1-3+k_2+5}+2^{p_1+k_2+4}\le 2^{p_1+k_2+5}, \end{aligned}$$

and thus $$p_1\le p_2\le p_1+5$$.

*(2)* First observe that $2^{k}\le 2^{k_1}+2^{k_2}\le 2^{k_2+2}$. If $p\le p_2-10$ then

$$\begin{aligned}&2^{p_2+k_2}\le 2^{p+k}+2^{p_1+k_1}\le 2^{p_2-10+k_2+2}+2^{p_1+k_1}, \\&2^{p_1+k_1}\le 2^{p+k}+2^{p_2+k_2}\le 2^{p_2+k_2+2}. \end{aligned}$$

Moreover, there holds $p_{\max }=p_1$ and $p\le p_2-10\le p_1-12$. If on the other hand $\left\lvert p-p_2\right\rvert \le 10$ then only the following inequality holds $2^{p_1+k_1}\le 2^{p+k}+2^{p_2+k_2}\le 2^{p_2+k_2+3}$. By symmetry, the proof of *(3)* is analogous to that of *(2)*. $\square$

### Set-Size Estimates

In this section we present a key ingredient in the proofs of bounds for bilinear estimates Lemma 6.1, Propositions 7.1, 8.1, 8.2. In particular, in bounding localized bilinear expressions in $L^2$, we can “gain" the smallest of the parameters $\frac{p}{2},\frac{p_i}{2},\frac{q}{2}, \frac{q_i}{2}$, $i=1,2$.

#### Lemma 5.15

Let $f,g\in L^2$ and $\mathfrak {m}$ our multiplier. Then for a bilinear expression

$$\begin{aligned} \widehat{\mathcal {Q}_{\mathfrak {m}}(f,g)}(\xi )=\int _{\mathbb {R}^2} e^{-it\Phi }\widetilde{\chi }(\xi ,\eta )\mathfrak {m}(\xi ,\eta )\hat{f}(\xi -\eta )\hat{g}(\eta )d\eta , \end{aligned}$$

where $\widetilde{\chi }(\xi ,\eta )$ as in (2.17)

$$\begin{aligned} \left\Vert \mathcal {Q}_\mathfrak {m}(f,g)\right\Vert _{L^2}\lesssim \left\lvert S\right\rvert \left\Vert m\right\Vert _{L^\infty _{\xi ,\eta }}\left\Vert P_{k_1,p_1,q_1}f\right\Vert _{L^2}\left\Vert P_{k_2,p_2,q_2}g\right\Vert _{L^2} \end{aligned}$$

with

$$\begin{aligned} \left\lvert S\right\rvert := \min \{ 2^{\frac{k}{2}+\frac{p}{2}},2^{\frac{k_1}{2}+\frac{p_1}{2}},2^{\frac{k_2}{2}+\frac{p_2}{2}} \}\cdot \min \{ 2^{\frac{k}{2}+\frac{q}{2}},2^{\frac{k_1}{2}+\frac{q_1}{2}},2^{\frac{k_2}{2}+\frac{q_2}{2}} \}. \end{aligned}$$

#### Proof

Without loss of generality, we localize in the $\xi$ and $\xi -\eta$ variable and assume $2^{k+p}\lesssim 2^{k_1+p_1}$ and $2^{k_1+q_1}\lesssim 2^{k+q}$. For $h\in L^2$ we have

$$\begin{aligned} \left\lvert \langle \mathcal {Q}_\mathfrak {m}(f,g),h\rangle \right\rvert&\lesssim \left\Vert \mathfrak {m}\right\Vert _{L^\infty _{\xi ,\eta }}\Vert \hat{h}(\xi )\hat{f}(\xi -\eta )\Vert _{L^2_{\xi ,\eta }}\left\Vert \varphi _{k,p,q}(\xi )\varphi _{k_1,p_1,q_1}(\xi -\eta )\hat{g}(\eta )\right\Vert _{L^2_{\xi ,\eta }}\\&\lesssim \left\Vert \mathfrak {m}\right\Vert _{L^\infty _{\xi ,\eta }} \left\Vert f\right\Vert _{L^2}\left\Vert h\right\Vert _{L^2}2^{\frac{k}{2}+\frac{p}{2}}2^{\frac{k_1}{2}+\frac{q_1}{2}}\left\Vert g\right\Vert _{L^2}, \end{aligned}$$

where we have used the support properties of $\varphi _{k,p,q}$. The claim follows by exchanging the variables. $\square$

#### Remark 5.16

We note that we use the *q*-localization only in once instance in the proof of Proposition 8.2, Case D. Otherwise we will use the set-size estimate above with $\chi (\xi ,\eta )=\varphi _{k,p}(\xi )\varphi _{k_1,p_1}(\xi -\eta )\varphi _{k_2,p_2}(\eta )$.

### Normal Forms

In this section we present how to use normal forms in in proving bounds on bilinear expression. In particular, we discuss a way to split the analysis into two regions depending on the size of the phase $\Phi$. In the so-called non-resonant part, where a positive lower bound on $\left\lvert \Phi \right\rvert$ is available, one can integrate by parts in time and use the improved decay of the time derivative from Sect. 6, see (5.12). On the other hand, in the resonant part set-size estimates are available, see Lemma 5.17(3)–(4).

Let $\Phi$ be one of the phases defined in (2.5) and $\psi$ as in Sect. 2.3. For $\lambda >0$ to be appropriately chosen, we split the multiplier in a resonant and non-resonant part as follows:

$$\begin{aligned} \mathfrak {m}(\xi ,\eta )=\psi (\lambda ^{-1}\Phi )\mathfrak {m}(\xi ,\eta )+(1-\psi (\lambda ^{-1}\Phi ))\mathfrak {m}(\xi ,\eta )=: \mathfrak {m}^{res}(\xi ,\eta )+\mathfrak {m}^{nr}(\xi ,\eta ). \end{aligned}$$

This yields the following decomposition:

$$\begin{aligned} \mathcal {B}_{\mathfrak {m}}(f,g)=\mathcal {B}_{\mathfrak {m}^{res}}(f,g)+\mathcal {B}_{\mathfrak {m}^{nr}}(f,g). \end{aligned}$$

Furthermore, after integrating by parts, the non-resonant part can be written as follows:

5.12 $$\begin{aligned} \mathcal {B}_{\mathfrak {m}^{nr}}(f,g)=\mathcal {Q}_{\mathfrak {m}^{nr}\Phi ^{-1}}(f,g)+\mathcal {B}_{\mathfrak {m}^{nr}\Phi ^{-1}}(\partial _tf,g)+\mathcal {B}_{\mathfrak {m}^{nr}\Phi ^{-1}}(f,\partial _tg). \end{aligned}$$

The next lemma provides useful set-size estimates for the terms in the decomposition above.

#### Lemma 5.17

Let $\lambda >0$, functions $\chi ,\; \widetilde{\chi }$ as in (2.17) and $f_j$ be localized profiles for $j=1,2$. Assume we have a splitting as in (5.12), then

1. The boundary term satisfies
  $$\begin{aligned} \left\Vert P_{k,p}\mathcal {Q}_{\mathfrak {m}^{nr}\Phi ^{-1}}(f_1,f_2)\right\Vert _{L^2}\lesssim 2^{k+p_{\max }}\lambda ^{-1}\left\lvert S\right\rvert \left\Vert f_1\right\Vert _{L^2}\left\Vert f_2\right\Vert _{L^2}. \end{aligned}$$
2. If $\lambda >0$ is chosen such that $\left\lvert \Phi \chi \right\rvert \ge \lambda \gtrsim 1$ then $\mathfrak {m}^{res}=0$ and $\mathfrak {m}=\mathfrak {m}^{nr}$ with the following bound
  $$\begin{aligned} \left\Vert P_{k,p}\mathcal {Q}_{\mathfrak {m}^{nr}\Phi ^{-1}}(f_1,f_2)\right\Vert _{L^2}\lesssim 2^{k+p_{\max }}\min \{\left\Vert e^{it\Lambda }f_1\right\Vert _{L^\infty }\left\Vert f_2\right\Vert _{L^2},\left\Vert f_1\right\Vert _{L^2}\left\Vert e^{it\Lambda }f_2\right\Vert _{L^\infty }\}. \end{aligned}$$
3. If $\left\lvert \partial _{\eta _1}\Phi \chi \right\rvert \gtrsim K>0$ or $\left\lvert \partial _{\xi _1}\Phi \chi \right\rvert \gtrsim K>0$, then
  $$\begin{aligned}&\left\Vert P_{k,p}\mathcal {Q}_{\mathfrak {m}^{res}}(f_1,f_2)\right\Vert _{L^2}\lesssim 2^{k+p_{\max }}\lambda ^{\frac{1}{2}}K^{-\frac{1}{2}}\min \{2^{\frac{k_1+p_1}{2}},2^{\frac{k_2+p_2}{2}} \}\left\Vert f_1\right\Vert _{L^2}\left\Vert f_2\right\Vert _{L^2}. \end{aligned}$$
4. If $\left\lvert \partial _{\eta _2}\Phi \widetilde{\chi }\right\rvert \gtrsim K>0$ or $\left\lvert \partial _{\xi _2}\Phi \widetilde{\chi }\right\rvert \gtrsim K>0$, then there holds
  $$\begin{aligned}&\left\Vert P_{k,p,q}\mathcal {Q}_{\mathfrak {m}^{res}}(f_1,f_2)\right\Vert _{L^2}\lesssim 2^{k+p_{\max }+q_{\max }}\lambda ^{\frac{1}{2}}K^{-\frac{1}{2}}\min \{2^{\frac{k_1+q_1}{2}},2^{\frac{k_2+q_2}{2}} \}\left\Vert f_1\right\Vert _{L^2}\left\Vert f_2\right\Vert _{L^2}. \end{aligned}$$
5. The analogous bounds (1)–(3) hold when additionally localizing in $q, q_i,$ $i=1,2,$ with $\widetilde{\chi }$ as in (2.17).

#### Proof

The claims (1) and (2) follow by the set-size estimate Lemma 5.15 and multiplier bounds Lemmas A.2, A.3. We prove the third claim. Assume $\left\lvert \partial _{\eta _1}\Phi \right\rvert \chi \gtrsim K$ and without loss of generality that $2^{k_2+p_2}\lesssim 2^{k_1+p_1}$ (otherwise exchange *h* and $\hat{f}$). For any $h\in L^2$ there holds:

$$\begin{aligned} |\langle {\mathcal {Q}}_{\mathfrak {m}^{res}} (f,g),h\rangle |&\lesssim \int _{\mathbb {R}^2}\int _{\mathbb {R}^2} \left\lvert \mathfrak {m}(\xi ,\eta )\right\rvert \chi (\xi ,\eta )\varphi (\lambda ^{-1}\Phi )|\hat{f}(\xi -\eta )\hat{g}(\eta )|\left\lvert h(\xi )\right\rvert d\xi d\eta \\&\lesssim \left\Vert \mathfrak {m}\right\Vert _{L^\infty _{\xi ,\eta }}\Vert \hat{f}(\xi -\eta )\hat{g}(\eta )\Vert _{L^{2}_{\xi ,\eta }}\left\Vert \chi (\xi ,\eta )\varphi (\lambda ^{-1}\Phi ) h(\xi )\right\Vert _{L^{2}_{\xi ,\eta }}. \end{aligned}$$

It remains to bound the last term and the claim. Observe that

$$\begin{aligned} \left\Vert \chi (\xi ,\eta )\varphi (\lambda ^{-1}\Phi ) h(\xi )\right\Vert _{L^{2}_{\xi ,\eta }}^2\lesssim \sup _{\xi }\int _{\eta \in \mathbb {R}^2} |\chi (\xi ,\eta )\varphi (\lambda ^{-1}\Phi )|^2d\eta \left\Vert h\right\Vert _{L^2_\xi }^2. \end{aligned}$$

It remains to prove that

$$\begin{aligned} \sup _{\xi } \int _{\eta \in \mathbb {R}^2} |\chi (\xi ,\eta )\varphi (\lambda ^{-1}\Phi )|^2d\eta \lesssim 2^{k_2+p_2}\lambda K^{-1}. \end{aligned}$$

To that end, we do a change of variables $\eta \mapsto (\Phi (\xi ,\eta ),\eta _2)=:\zeta$ and use the fact that $|\det {\frac{\partial \zeta }{\partial \eta }}|=\left\lvert \partial _{\eta _1}\Phi \right\rvert$ to obtain, for a fixed $\xi$, that

$$\begin{aligned} \int _{\mathbb {R}^2}|\chi (\xi ,\eta )\varphi (\lambda ^{-1}\Phi )|^2d \eta&\lesssim \int _{\mathbb {R}^2} \left\lvert \chi (\xi ,\zeta )\varphi (\lambda ^{-1}\zeta _1)\right\rvert ^2 \left\lvert \det {\frac{\partial \zeta }{\partial \eta }}\right\rvert ^{-1}d\zeta \lesssim 2^{k_2+p_2} \lambda K^{-1}. \end{aligned}$$

In case $\left\lvert \partial _{\xi _1}\Phi \right\rvert \gtrsim K$, use the change of variables $\xi \mapsto (\Phi (\xi ,\eta ),\xi _2)$ instead. The proof of the fourth statement is analogous using a change of variables $\eta \mapsto (\eta _1,\Phi (\xi ,\eta ))$ if $\left\lvert \partial _{\eta _2}\Phi \right\rvert \gtrsim K$ and $\xi \mapsto (\xi _1, \Phi (\xi ,\eta ))$ if $\left\lvert \partial _{\xi _2}\Phi \right\rvert \gtrsim K$. $\square$

## Bounds on $\partial _tS^NF$ in $L^2$

In this section we prove time decay of the time derivative of profiles in $L^2$, which will be used in subsequent sections when performing normal forms.

### Lemma 6.1

Let $F\in \{\mathcal {Z}_\pm ,\Theta \}$ and assume the bootstrap assumption (2.26) holds. Then for $\delta =2M^{-1}>0$, $m\in \mathbb {N}$ and $t\in [2^m,2^{m+1}[\cap [0,T]$

$$\begin{aligned} \Vert \partial _tP_kS^bF(t)\Vert _{L^2}\lesssim 2^{\frac{3}{4}k}2^{-2k^+}2^{(-\frac{3}{4}+2\delta ) m}\varepsilon ^2. \end{aligned}$$

The lemma is proved following the scheme discussed in Sect. 1.1: Using the energy estimates in **Case A**, we can reduce to proving the claim (6.4) for parameter-localized interactions. **Case B** deals with the “gap in *p*" setting when we can integrate by parts using Lemma 5.10. When this is not feasible, we take advantage of the smallness of the set over which we integrate to obtain the claim via Lemma 5.15. The “no gap" setting is handled in **Case C** using the linear decay estimate from Sect. 3. Throughout the proof we employ the multiplier bound from Lemma 5.5.

### Proof

By the Duhamel formulations (2.10), (2.11) and Lemma 2.8, it suffices to suitably bound the sums $\sum _{k_1,k_2\in \mathbb {Z}}\Vert P_k\mathcal {Q}_\mathfrak {m}(P_{k_1}S^{b_1}F_1,P_{k_2}S^{b_2}F_2)\Vert _{L^2}$, where, $b_1+b_2\le N$ and $F_i\in \{\mathcal {Z}_\pm ,\Theta \},\, i=1,2$.

**Case A: Simple cases.** The set size estimate Lemma 5.15 and the multiplier bound Lemma 5.5 yield

$$\begin{aligned}&\Vert P_k\mathcal {Q}_\mathfrak {m}(P_{k_1}S^{b_1}F_1,P_{k_2}S^{b_2}F_2)\Vert _{L^2} \\&\quad \lesssim \left\lvert S\right\rvert \left\Vert \mathfrak {m}\right\Vert _{L^\infty _{\xi ,\eta }}\Vert P_{k_1}S^{b_1}F_1\Vert _{L^2}\Vert P_{k_2}S^{b_2}F_2\Vert _{L^2}\\&\quad \lesssim 2^{k_{\min }}2^{\frac{p_{\min }}{2}}2^{k}2^{p_{\max }}2^{-N_0(k_1^++k_2^+)}\Vert P_{k_1}S^{b_1}F_1\Vert _{H^{N_0}}\Vert P_{k_2}S^{b_2}F_2\Vert _{H^{N_0}}. \end{aligned}$$

Hence if $k_{\max }\ge \delta _0m:= 2N^{-1}_0m$ or $k_{\min }\le -2m$ and since $N_0>4$, we obtain with the bootstrap assumption (2.26) that

6.1 $$\begin{aligned} \begin{aligned} \sum _{\begin{array}{c} k_1,k_2\in \mathbb {Z}\\ k_{\max }\ge \delta _0m \text { or } k_{\min }\le -2m \end{array} }\Vert P_k\mathcal {Q}_\mathfrak {m}(P_{k_1}S^{b_1}F_1,P_{k_2}S^{b_2}F_2)\Vert _{L^2}\lesssim 2^k2^{-2k^+}2^{-m}\varepsilon ^2, \end{aligned} \end{aligned}$$

and it remains to bound

$$\begin{aligned} \sum _{\begin{array}{c} k_1,k_2 \in \mathbb {Z}\\ -2m<k_1,k_2<\delta _0m \end{array}}\Vert P_k\mathcal {Q}_\mathfrak {m}(P_{k_1}S^{b_1}F_1,P_{k_2}S^{b_2}F_2)\Vert _{L^2}. \end{aligned}$$

Localizing further in $p,\,p_i$ and $l_i$, $i=1,2$, and writing $f_i=P_{k_i,p_i}R_{l_i}S^{b_i}F_i$ we have

6.2 $$\begin{aligned} \Vert P_k\mathcal {Q}_\mathfrak {m}(P_{k_1}S^{b_1}F_1,P_{k_2}S^{b_2}F_2)\Vert _{L^2}=\sum _{p\in \mathbb {Z}^-}\sum _{\begin{array}{c} p_1\in \mathbb {Z}^-, l_1\in \mathbb {Z}^+\\ p_1+l_1\ge 0 \end{array}}\sum _{\begin{array}{c} p_2\in \mathbb {Z}^-, l_2\in \mathbb {Z}^+\\ p_2+l_2\ge 0 \end{array}} \left\Vert P_{k,p}\mathcal {Q}_{\mathfrak {m}}(f_1,f_2)\right\Vert _{L^2}. \end{aligned}$$

Observe that for $\max \{l_1,l_2\}\ge 2m$

$$\begin{aligned}&\sum _{\begin{array}{c} p_i\in \mathbb {Z}^-, l_i\in \mathbb {Z}^+\\ \max \{l_1,l_2\}\ge 2m \end{array}}\left\Vert P_{k,p}\mathcal {Q}_{\mathfrak {m}}(f_1,f_2)\right\Vert _{L^2} \\&\quad \lesssim \hspace{-9pt}\sum _{\begin{array}{c} p_i\in \mathbb {Z}^-, l_i\in \mathbb {Z}^+\\ \max \{l_1,l_2\}\ge 2m \end{array}}\hspace{-9pt}2^{k_{\min }}2^{\frac{p_{\min }}{2}}2^k2^{-4k_1^+}2^{-l_1}2^{-\frac{p_1}{2}}\left\Vert f_1\right\Vert _X2^{-4k_2^+}2^{-l_2}2^{-\frac{p_2}{2}}\left\Vert f_2\right\Vert _X\\&\quad \lesssim 2^{k}2^{-2k^+}2^{(-1+\delta )m}\varepsilon ^2. \end{aligned}$$

Therefore, it remains to bound bilinear terms for the following localization parameters:

6.3 $$\begin{aligned}&-2m<k,\;k_1,\;k_2<\delta _0m,  &   -2m< p_1,\;p_2\le 0,  &   0\le l_1,\;l_2< 2m. \end{aligned}$$

Observe that each sum on the right-hand side of (6.1), (6.2) ranges over an interval of order $m\lesssim 2^{\gamma m}$ for all $\gamma >0$. Thus, it suffices to prove

6.4 $$\begin{aligned} \left\Vert P_{k,p}\mathcal {Q}_{\mathfrak {m}}(f_1,f_2)\right\Vert _{L^2}\lesssim 2^{\frac{3}{4}k}2^{-2k^+}2^{(-\frac{3}{4}+\delta )m}\varepsilon ^2, \end{aligned}$$

for the localization parameters as in (6.3) and $\delta =2 M^{-1}$.

**Case B: Gap in** *p* **with** $p_{\min }\ll p_{\max }$**.** In this part we assume without loss of generality that $k_1\le k_2$. Then $k_{\min }\in \{k,k_1\}$ and $k_{\max }\in \{k,k_2\}$ and so in particular $2^{k_{\max }+k_{\min }}\sim 2^{k+k_1}.$ In this case by Proposition 5.8 there holds $\left\lvert \sigma \right\rvert \gtrsim 2^{p_{\max }}2^{k_1+k}$. With the condition $k_1\le k_2$, we have two further subcases to cover, namely $k_{\min }=k_1$ and $k_{\min }=k$.

**Case B.1:** $k_{\min }=k_1$, then $2^{k}\sim 2^{k_2}$. If

6.5 $$\begin{aligned} -p_{\max }+2l_1\le (1-\delta )m, \end{aligned}$$

we obtain the claim by integrating by parts $M\gg N$ times along $S_{\eta }$. Indeed, by Lemma 5.10(1) and the energy estimates (2.27)

$$\begin{aligned} \left\Vert P_{k,p}\mathcal {Q}_{\mathfrak {m}}(f_1,f_2)\right\Vert _{L^2}&\lesssim 2^k \left\Vert \mathcal {F}(\mathcal {Q}_\mathfrak {m}(f_1,f_2))\right\Vert _{L^\infty }\\&\lesssim 2^k2^{k_{\max }} [2^{-m}2^{-p_1}2^{k_{1}-k-p_{\max }}(1+2^{k_2-k_1}2^{l_1})]^M \\&\hspace{1cm} \cdot \Vert P_{k_1,p_1}R_{l_1}(1,S)^Mf_1\Vert _{L^2}\Vert P_{k_2,p_2}R_{l_2}(1,S)^Mf_2\Vert _{L^2}\\&\lesssim 2^{k}2^{-{3}k^+}[2^{-m}2^{-p_1-p_{\max }}2^{l_1}]^M\varepsilon ^2\\&\lesssim 2^{k}2^{-{3}k^+}2^{-2m}\varepsilon ^2, \end{aligned}$$

where $\delta :=2M^{-1}\ll 1$. Similarly, integration by parts along $S_{\xi -\eta }$ as per Lemma 5.10(1) yields the claim (6.4) if

6.6 $$\begin{aligned} \max \{ k-k_1-p_{\max }+l_2, 2l_2-p_{\max } \}<(1-\delta )m. \end{aligned}$$

Indeed, from Lemma 5.10(1) with $2^{k_1-k_2}\lesssim 1$, $2^{k_2}\sim 2^k$ and $2^{-p_2}\lesssim 2^{l_2}$, the contribution at each iteration is

$$\begin{aligned} 2^{-m}2^{2k_2}2^{-p_2}2^{-p_{\max }-{k_1}-{k}}(1+2^{k_1-k_2}2^{l_2})\lesssim 2^{-m}2^{k-k_1}2^{-p_{\max }}2^{l_2}+2^{-m}2^{-p_{\max }}2^{2l_2}. \end{aligned}$$

Assume now that (6.5) and (6.6) don’t hold, then we consider two cases depending on which term on the right-hand side of (6.6) is larger.

**1.** Assume first that $-2l_1<-(1-\delta )m-p_{\max }$ and $-l_2<-(1-\delta )m-p_{\max }+k-k_1$. By the set size estimate Lemma 5.15 with $\left\lvert S\right\rvert \lesssim 2^{k_1+\frac{p_1}{2}}$ we obtain:

$$\begin{aligned} \left\Vert P_{k,p}\mathcal {Q}_{\mathfrak {m}}(f_1,f_2)\right\Vert _{L^2}&\lesssim 2^{k}2^{p_{\max }} 2^{k_{\min }+\frac{p_{\min }}{2}}2^{-4k_1^+}2^{-{l_1}}2^{-\frac{p_1}{2}}\left\Vert f_1\right\Vert _X2^{-4k^+}2^{-\frac{l_2}{2}}\left\Vert f_2\right\Vert _X\\&\lesssim 2^{k+p_{\max }}2^{k_1}2^{-4k_1^+-4k^+}2^{(-1+\delta )m}2^{-p_{\max }}2^{\frac{k-k_1}{2}}\varepsilon ^2\\&\lesssim 2^k2^{-3k^+}2^{(-1+\delta )m}\varepsilon ^2. \end{aligned}$$

**2.** Assume $\max \{-2l_1,-2l_2\}<-(1-\delta )m-p_{\max }$. Again using the size set estimate Lemma 5.15 we obtain

$$\begin{aligned} \left\Vert P_{k,p}\mathcal {Q}_{\mathfrak {m}}(f_1,f_2)\right\Vert _{L^2}&\lesssim 2^{k}2^{p_{\max }} 2^{k_{\min }+\frac{p_{\min }}{2}}2^{-4k_1^+}2^{-l_1-\frac{p_1}{2}}\left\Vert f_1\right\Vert _X2^{-4k^+}2^{-\frac{l_2}{2}}\left\Vert f_2\right\Vert _X\\&\lesssim 2^k2^{-2k^+}2^{p_{\max }}2^{(-\frac{3}{4}+\frac{3}{4}\delta )m}2^{-\frac{3}{4}p_{\max }}\varepsilon ^2\\&\lesssim 2^k2^{-2k^+}2^{(-\frac{3}{4}+\delta )m}\varepsilon ^2. \end{aligned}$$

**Case B.2:** $k_{\min }=k$ and therefore $2^{k_2}\sim 2^{k_1}$. We obtain the claim (6.4) through integration by parts along $S_{\eta }$ if $k_1-k-p_{\max }+2l_1\le (1-\delta )m.$ Indeed,

$$\begin{aligned} \left\Vert P_{k,p}\mathcal {Q}_{\mathfrak {m}}(f_1,f_2)\right\Vert _{L^2}&\lesssim 2^k \left\Vert \mathcal {F}(\mathcal {Q}_\mathfrak {m}(f_1,f_2))\right\Vert _{L^\infty }\\&\lesssim 2^{k+k_{\max }} [2^{-m}2^{-p_1}2^{k_1-k-p_{\max }}(1+2^{k_2-k_1}2^{l_1})]^M \\&\hspace{1cm} \cdot \left\Vert P_{k_1,p_1}R_{l_1}(1,S)^Mf_1\right\Vert _{L^2}\left\Vert P_{k_2,p_2}R_{l_2}(1,S)^Mf_2\right\Vert _{L^2}\\&\lesssim 2^{k}2^{-3k^+}[2^{-m}2^{k_1-k}2^{2l_1}2^{-p_{\max }}]^M\varepsilon ^2\\&\lesssim 2^{k}2^{-3k^+}2^{-2m}\varepsilon ^2. \end{aligned}$$

Similarly, by integrating by parts in $S_{\xi -\eta }$, we obtain the claim (6.4) if $k_1-k-p_{\max }+2l_2\le (1-\delta )m$. Otherwise if $\max \{-2l_1,-2l_2\}< -(1-\delta )m+k_1-k-p_{\max }$, we estimate as in **Case B.1**:

$$\begin{aligned} \left\Vert P_{k,p}\mathcal {Q}_{\mathfrak {m}}(f_1,f_2)\right\Vert _{L^2}&\lesssim 2^{k}2^{p_{\max }} 2^{k_{\min }+\frac{p_{\min }}{2}}2^{-8k_1^+}2^{-l_1-\frac{p_1}{2}}\left\Vert f_1\right\Vert _X2^{-\frac{l_2}{2}}\left\Vert f_2\right\Vert _X\\&\lesssim 2^{k+p_{\max }}2^{\frac{k_1+k}{2}}2^{-8k_1^+}2^{(-\frac{3}{4}+\delta )m}2^{-\frac{3}{4}p_{\max }}2^{\frac{3}{4}(k_1-k)}\varepsilon ^2\\&\lesssim 2^{\frac{3}{4}k}2^{-2k^+}2^{(-\frac{3}{4}+\delta )m}\varepsilon ^2. \end{aligned}$$

**Case C: No gaps with** $p\sim {p_1}\sim {p_2}$**.** Assume without loss of generality that $f_1$ has fewer vector fields than $f_2$, i.e. $b_1\le b_2$. Then we can use Proposition 3.2 on $f_1$ such that for $0<\beta '<\beta$ we have the decomposition

$$\begin{aligned} P_{k_1,p_1}e^{it\Lambda }f_1=I_{k_1,p_1}(f_1)+II_{k_1,p_1}(f_1). \end{aligned}$$

Moreover, we can apply Lemma 3.4 with $\kappa \ll \beta '$ on $f_2$, so that the following bounds hold

$$\begin{aligned} \left\| I_{k_1,p_1}(f_1)\right\| _{L^\infty }&\lesssim 2^{\frac{3}{4}k_1}2^{-\frac{15}{4}k_1^+}2^{-p}2^{(-1+\delta )m}\left\| f_1\right\| _D, \\\quad \left\| II_{k_1,p_1}(f_1)\right\| _{L^2}&\lesssim 2^{-4k_1^+}2^{-\frac{p}{2}}2^{-\frac{m}{2}} \left\| f_1\right\| _D,\\\Vert P_{k_2,p_2}e^{it\Lambda }f_2\Vert _{L^\infty }&\lesssim 2^{\frac{3}{4}k_2}2^{-3k_2^+}2^{(-\frac{1}{2}+\kappa )m}\varepsilon ^2. \end{aligned}$$

With these, we obtain the claim (6.4):

$$\begin{aligned} \left\| P_{k,p}\mathcal {Q}_{\mathfrak {m}}(f_1,f_2)\right\| _{L^2}&\lesssim \left\| \mathfrak {m}I_{k_1,p_1}(f_1)e^{it\Lambda }P_{k_2,p_2}f_2\right\| _{L^2}+\left\| \mathfrak {m}II_{k_1,p_1}(f_1)e^{it\Lambda }P_{k_2,p_2}f_2\right\| _{L^2}\\  &\lesssim 2^{k+p}(\left\| I_{k_1,p_1}(f_1)\right\| _{L^\infty }\left\| P_{k_2,p_2}e^{it\Lambda }f_2\right\| _{L^2} \\  &\hspace{1cm} +\left\| II_{k_1,p_1}(f_1)\right\| _{L^2}\left\| P_{k_2,p_2}e^{it\Lambda }f_2\right\| _{L^\infty })\\  &\lesssim 2^{k+p}[2^{\frac{3}{4}k_1}2^{-\frac{15}{4}k_1^+}2^{-p}2^{(-1+\delta )m}\left\| f_1\right\| _D2^{-4k_2^+}2^{\frac{p}{2}}\left\| f_2\right\| _B\\  &\hspace{1cm}+ 2^{-4k_1^+}2^{-(\frac{1}{2}+2\beta ')p}2^{(-\frac{1}{2}-\beta ')m}\left\| f_1\right\| _D2^{\frac{3}{4}k_2-3k_2^+}2^{(-\frac{1}{2}+\kappa )m}\varepsilon ]\\  &\lesssim 2^k 2^{-2k_1^+-2k_2^+}[2^{(-1+\delta )m}+2^{(-\frac{1}{2}-\beta ')m}2^{(-\frac{1}{2}+\kappa )m}]\varepsilon ^2\\  &\lesssim 2^k2^{-2k^+}2^{(-1+\delta )m}\varepsilon ^2. \end{aligned}$$

This concludes the proof of the lemma. $\square$

## Bounds on the *B*-Norm

In this section we prove bounds on the *B*-norm of bilinear terms needed in the proof of Proposition 2.7. As explained in (2.29), (2.30) it suffices to suitably bound $\mathcal {F}\mathcal {B}_\mathfrak {m}(F_1,F_2)(t,\xi )=\int _0^t \mathcal {Q}_\mathfrak {m}(F_1,F_2)(s,\xi )ds,$

where $\mathcal {Q}_\mathfrak {m}(F_1,F_2)$ is a bilinear expression as in (5.8) and $\mathcal {B}_\mathfrak {m}$ is localized on a time interval $t\in [2^m,2^{m+1}[$, $m\in \mathbb {N}$.

### Proposition 7.1

In the setting of Proposition 2.7, and in particular under the bootstrap assumptions (2.26), the following holds true: For $\mathfrak {m}\in \left\{ \mathfrak {m}_0,\mathfrak {m}_\pm ^{\mu \nu } \mid \mu ,\nu \in {\{+,-\}} \right\} ,$ $t\in [2^m,2^{m+1}[\cap [0,T]$ and $\delta =2M^{-\frac{1}{2}}$, there holds that

$$\begin{aligned} \left\Vert \mathcal {B}_\mathfrak {m}(F_1,F_2)\right\Vert _B\lesssim 2^{(\frac{1}{6}+2\delta ) m}\varepsilon ^2 +2^{(\frac{1}{4}+3\delta ) m}\varepsilon ^3, \end{aligned}$$

where $$F_i\in \{S^{b_i}\mathcal {Z}_{\pm },S^{b_i}\Theta \}$$, $$0\le b_1+b_2\le N$$, $$i=1,2$$.

The proof follows the outline presented in Sect. 1.1 and expands on the arguments already employed in Sect. 6. The *simple cases* and the *no gaps* cases (**Cases A, D** below) follow along similar lines to the proof in Sect. 6. The *gap in p* case is split into two parts: if $\max \{p,p_1,p_2\}\sim 0$ (**Case B** below), the claim follows using integration by parts or else the *B*- and *X*-norms and set-size estimates. On the other hand, if (**Case C** below) $\max \{p,p_1,p_2\}\ll 0$ and thus $\left\lvert \Phi \right\rvert \gtrsim 1$, we can employ normal forms, a new and central feature compared to the proof of Lemma 6.1. This allows for the localized bilinear expressions to be bounded by (see (5.12)):

$$\begin{aligned} \left\Vert P_{k,p}\mathcal {B}_{\mathfrak {m}}(f_1,f_2)\right\Vert _{L^2}\lesssim&\left\Vert P_{k,p}\mathcal {Q}_{\mathfrak {m}\Phi ^{-1}}(f_1,f_2)\right\Vert _{L^2}+\left\Vert P_{k,p}\mathcal {B}_{\mathfrak {m}\Phi ^{-1}}(\partial _tf_1,f_2)\right\Vert _{L^2} \\&+\left\Vert P_{k,p}\mathcal {B}_{\mathfrak {m}\Phi ^{-1}}(f_1,\partial _tf_2)\right\Vert _{L^2}, \end{aligned}$$

where $f_i,$ $i=1,2$ are profiles localized both in frequency and space. The first boundary term is relatively easy to estimate as we have one time parameter less to “gain". The other two terms are handled using the bound on the time derivative Lemma 6.1.

### Proof

By the definition of the *B*-norm in (2.22) we have, after localizing in $k_i$, $i=1,2$,

$$\begin{aligned} \left\Vert \mathcal {B}_\mathfrak {m}(F_1,F_2)\right\Vert _B=\sup _{k\in \mathbb {Z},p\in \mathbb {Z}^-}2^{4k^+}2^{-\frac{k^-}{2}}2^{-\frac{p}{2}}\sum _{k_1,k_2\in \mathbb {Z}}\left\Vert P_{k,p}\mathcal {B}_\mathfrak {m}(P_{k_1}F_1,P_{k_2}F_2)\right\Vert _{L^2}. \end{aligned}$$

**Case A: Simple cases.** If for $\delta _0:=2N_0^{-1}$ there holds that $k_{\max }\ge \delta _0m$ or $k_{\min }\le -4m$, then the claim is obtained using Lemma 5.15, the multiplier bound Lemma 5.5 and the bootstrap assumption (2.26) together with the energy estimates (2.27), by summing the following bound over $k_1,\;k_2$ within this range:

$$\begin{aligned}&2^{4k^+}2^{-\frac{k^-}{2}}2^{-\frac{p}{2}}\left\Vert P_{k,p}\mathcal {B}_\mathfrak {m}(P_{k_1}F_1,P_{k_2}F_2)\right\Vert _{L^2} \\&\quad \lesssim 2^m2^{\frac{9}{2}k^+}2^{-\frac{k}{2}}2^{-\frac{p}{2}}\left\lvert S\right\rvert \left\Vert \mathfrak {m}\chi \right\Vert _{L^\infty }\Vert P_{k_1}F_1\Vert _{L^2}\Vert P_{k_2}F_2\Vert _{L^2}\\&\quad \lesssim 2^m2^{\frac{k_{\min }}{2}+k+\frac{9}{2}k^+}2^{-N_0k_1^+}2^{-N_0k_2^+}\Vert P_{k_1}F_1\Vert _{H^{N_0}}\Vert P_{k_2}F_2\Vert _{H^{N_0}}\\&\quad \lesssim 2^m2^{\frac{k_{\min }}{2}}2^{-(N_0-6)k_1^+}2^{-(N_0-6)k_2^+}\varepsilon ^2. \end{aligned}$$

Thus, from now on we can assume $-4m<k,k_1,k_2<\delta _0m$. We localize further in $p_i,\,l_i$ with $p_i+l_i\ge 0$, $i=1,2$ and let $f_i=P_{k_i,p_i}R_{l_i}F_i$. If $\max \{l_1,l_2\}\ge 4m$ we obtain with the set size estimate Lemma 5.15 and the bootstrap assumption:

$$\begin{aligned} {2^{4k^+}}2^{-\frac{k^-}{2}}2^{-\frac{p}{2}}\left\Vert P_{k,p}\mathcal {B}_\mathfrak {m}(f_1,f_2)\right\Vert _{L^2}&\lesssim 2^{m+2\delta _0m}2^{-l_1-l_2}2^{-\frac{p_1}{2}}2^{-\frac{p_2}{2}}\left\Vert f_1\right\Vert _X\left\Vert f_2\right\Vert _X\\&\lesssim 2^{(-1+2\delta _0)m}2^{-\frac{l_1+p_1}{2}}2^{-\frac{l_2+p_2}{2}}\varepsilon ^2. \end{aligned}$$

Thus, as explained in **Case A** in the proof of Lemma 6.1, see also (6.1)–(6.2), it suffices to establish the claim

7.1 $$\begin{aligned} \sup _{k,p}2^{4k^+}2^{-\frac{k^-}{2}}2^{-\frac{p}{2}}\left\Vert P_{k,p}\mathcal {B}_{\mathfrak {m}}(f_1,f_2)\right\Vert _{L^2}\lesssim 2^{(\frac{1}{6}+\delta ) m}\varepsilon ^2 +2^{(\frac{1}{4}+\frac{5}{2}\delta ) m}\varepsilon ^3 \end{aligned}$$

for the following localization parameters:

$$\begin{aligned}&-4m<k,k_i<\delta _0 m ,  &   -p_i\le l_i\le 4m,  &   -4m\le p_i\le 0,  &   i=1,2. \end{aligned}$$

**Case B: Gap in** *p* **with** $p_{\min }\ll p_{\max }\sim 0$**.** We assume w.l.o.g. that $p_1\le p_2$. Observe that by Proposition 5.8 there holds $\left\lvert \sigma \right\rvert \sim 2^{k_{\min }+k_{\max }}$. Moreover the multiplier bound $\left\Vert \mathfrak {m}\chi \right\Vert _{L^\infty }\lesssim 2^{k}$ holds by Lemma 5.5. Repeated integration by parts in $S_\eta$ or $S_{\xi -\eta }$ as per Lemma 5.10(1) yields the claim if

7.2 $$\begin{aligned} \begin{aligned} S_{\eta }:\hspace{1cm} 2^{2k_1}2^{-p_1}2^{-k_{\max }-k_{\min }}(1+2^{k_2-k_1}2^{l_1})&\le 2^{(1-\delta )m}, \\ S_{\xi -\eta }:\hspace{1cm} 2^{2k_2}2^{-p_2}2^{-k_{\max }-k_{\min }}(1+2^{k_1-k_2}2^{l_2})&\le 2^{(1-\delta )m}, \end{aligned} \end{aligned}$$

where $\delta :=2M^{-\frac{1}{2}}\gg 2M^{-1}\gg 2N_0^{-1}=\delta _0$. Indeed, if the first condition above holds, integration by parts in $S_\eta$ with $\left\Vert \varphi _{k,p}\right\Vert _{L^2}\lesssim 2^{k}2^{\frac{p}{2}}$ and Lemma 5.10(1) give

7.3 $$\begin{aligned} \begin{aligned} 2^{4k^+-\frac{k^-}{2}}2^{-\frac{p}{2}}\left\| P_{k,p}\mathcal {B}_\mathfrak {m}(f_1,f_2)\right\| _{L^2}&\lesssim 2^{\frac{9}{2}k^+}2^{-\frac{k}{2}}2^{-\frac{p}{2}}\left\| \varphi _{k,p}\right\| _{L^2}\Vert \widehat{\mathcal {B}_\mathfrak {m}(f_1,f_2)}\Vert _{L^\infty }\\  &\lesssim 2^{5k^+}2^{k_{\max }}2^m [2^{-m}2^{2k_1}2^{-p_1}2^{-k_{\max }-k_{\min }}(1+2^{k_2-k_1}2^{l_1})]^M\\  &\quad \qquad \cdot \left\| P_{k_1,p_1}R_{l_1}(1,S)^Mf_1\right\| _{L^2}\left\| P_{k_2,p_2}R_{l_2}(1,S)^Mf_2\right\| _{L^2}\\  &\lesssim 2^{-m}\varepsilon ^2. \end{aligned} \end{aligned}$$

With a similar computation, we obtain the claim when integrating by parts along $S_{\xi -\eta }$.

Otherwise, if neither condition in (7.2) holds, we consider several cases that are organized according to Lemma 5.13.

**Case B.1:** $p_{\min }= p\ll p_{\max }$**.** From Lemma 5.13 and under the constraint $p_1\le p_2$, there are two further geometrical settings to consider.

**Case B.1(a):** $2^{k_1}\sim 2^{k_2}$. Then $p\ll p_1\sim p_2$, $k_{\max }+k_{\min }\sim k+k_1$ and moreover $\min \{l_1,l_2\}>(1-\delta )m+k-k_1$. From Lemma 5.15 with $\left\lvert S\right\rvert \lesssim 2^{k+\frac{p}{2}}$ and the bootstrap assumption (2.26) we obtain

$$\begin{aligned} 2^{4k^+}2^{-\frac{k^-}{2}}2^{-\frac{p}{2}}\left\| P_{k,p}\mathcal {B}_\mathfrak {m}(f_1,f_2)\right\| _{L^2}&\lesssim 2^{\frac{9}{2}k^+}2^m 2^{\frac{3}{2}k}2^{-8k_1^+}2^{-l_1}2^{-\frac{l_2}{2}}\left\| f_1\right\| _X\left\| f_2\right\| _X \\  &\lesssim 2^{(-\frac{1}{2}+4\delta )m}\varepsilon ^2. \end{aligned}$$

**Case B.1(b):** $2^{k_2}\ll 2^{k_1}\sim 2^{k}$**.** Then $p\le p_1\ll p_2=p_{\max }\sim 0$ and $k_{\max }+k_{\min }\sim k_2+k$. In this setting (7.2) for $S_{\xi -\eta }$ doesn’t hold if $l_2>(1-\delta )m$. The claim follows from Lemma 5.15 with $\left\lvert S\right\rvert \lesssim 2^{k+\frac{p}{2}}$ and (2.26):

$$\begin{aligned}&2^{4k^+-\frac{k^-}{2}}2^{-\frac{p}{2}}\left\Vert P_{k,p}\mathcal {B}_\mathfrak {m}(f_1,f_2)\right\Vert _{L^2} \lesssim 2^{4k^+-\frac{k^-}{2}}2^m2^{2k}2^{-4k_1^++\frac{k_1^-}{2}}\left\Vert f_1\right\Vert _B 2^{-(1+\beta )l_2}\left\Vert f_2\right\Vert _X \\&\quad \lesssim 2^{-\frac{\beta }{2}m}\varepsilon ^2. \end{aligned}$$

The last estimate follows since $\beta \gg \delta = 2M^{-1/2}$.

**Case B.2:** $p_{\min }= p_1\ll p_{\max }$**.** By Lemma 5.13, we have three subcases to consider.

**Case B.2(a):** $2^{k_1}\lesssim 2^{k}\sim 2^{k_2}$**.** Then $p_1\ll p\sim p_2\sim 0$ and $k_{\max }+k_{\min }\sim k_1+k$. If neither condition in (7.2) holds, we can assume that

$$\begin{aligned}&l_1-p_1>(1-\delta )m  &   \text {and}  &   \max \{k_2-k_1,l_2\}> (1-\delta )m. \end{aligned}$$

First let $\max \{k_2-k_1,l_2\}=l_2> (1-\delta )m$. Then it follows from Lemma 5.15 with $\left\lvert S\right\rvert \lesssim 2^{k_1+\frac{p_1}{2}}$:

$$\begin{aligned}&2^{4k^+}2^{-\frac{k^-}{2}}2^{-\frac{p}{2}}\left\Vert P_{k,p}\mathcal {B}_\mathfrak {m}(f_1,f_2)\right\Vert _{L^2} \\&\quad \lesssim 2^{4k^+}2^{-\frac{k^-}{2}}2^m2^{k_1+\frac{p_1}{2}}2^k2^{-4k_1^+}2^{-\frac{l_1}{2} } \left\Vert f_1\right\Vert _X 2^{-4k_2^+}2^{-(1+\beta )l_2}\left\Vert f_2\right\Vert _X\\&\quad \lesssim 2^{k^+}2^m2^{-(\frac{3}{2}+\beta )(1-\delta )m}\varepsilon ^2\\&\quad \lesssim 2^{(-\frac{1}{2}-\frac{\beta }{2})m}\varepsilon ^2, \end{aligned}$$

since $\beta \gg \delta$. Now assume $\max \{k_2-k_1,l_2\}=k_2-k_1> (1-\delta )m$ and $l_1-p_1>(1-\delta )m$, then the claim (7.1) follows from Lemma 5.15 with $\left\lvert S\right\rvert \lesssim 2^{k_1+\frac{p_1}{2}}$:

$$\begin{aligned} 2^{4k^+}2^{-\frac{k^-}{2}}2^{-\frac{p}{2}}\left\Vert P_{k,p}\mathcal {B}_\mathfrak {m}(f_1,f_2)\right\Vert _{L^2}&\lesssim 2^{4k^+}2^{-\frac{k^-}{2}}2^m 2^{k_1+\frac{p_1}{2}}2^{k_2}2^{-\frac{l_1}{2}}\left\Vert f_1\right\Vert _X 2^{-4k_2^+}2^{\frac{k_2^-}{2}}\left\Vert f_2\right\Vert _B\\&\lesssim 2^{m}2^{-\frac{3}{2}(1-\delta )m}\varepsilon ^2\\&\lesssim 2^{(-\frac{1}{2}+2\delta )m}\varepsilon ^2. \end{aligned}$$

**Case B.2(b):** $2^k\ll 2^{k_2}\sim 2^{k_1}.$ Then there holds $p_1\le p_2 \ll p\sim 0$, $k_{\max }+k_{\min }\sim k+k_2$. Moreover, by Lemma 5.13 there holds $2^{k}\lesssim 2^{k_2+p_2}$. Assume the second condition in (7.2) doesn’t hold, that is $-l_2<-(1-\delta )m -p_2+k_2-k.$ Then we obtain the claim (7.1) from Lemma 5.15 with $\left\lvert S\right\rvert \lesssim 2^{\frac{k}{2}+\frac{k_2+p_2}{2}}$ and $2^{k}\lesssim 2^{k_2+p_2}$:

$$\begin{aligned} 2^{4k^+}2^{-\frac{k^-}{2}}2^{-\frac{p}{2}}\left\Vert P_{k,p}\mathcal {B}_\mathfrak {m}(f_1,f_2)\right\Vert _{L^2}&\lesssim 2^{\frac{9}{2}k^+}2^m 2^{k}2^{\frac{k_2+p_2}{2}}2^{-8k_2^+}2^\frac{p_{1}}{2}\left\Vert f_1\right\Vert _X2^{-\frac{5}{6}l_2}2^{-\frac{1}{3}p_2}\left\Vert f_2\right\Vert _X\\&\lesssim 2^{-3k_2^+}2^m2^{k}2^{\frac{2}{3}p_2}2^{-\frac{5}{6}(1-\delta )m}2^{-\frac{5}{6}(p_2+k-k_2)}\varepsilon ^2\\&\lesssim 2^{(\frac{1}{6}+\delta )m}\varepsilon ^2. \end{aligned}$$

**Case B.2(c):** $2^{k_2}\ll 2^{k}\sim 2^{k_1}$**.** Then $p_1\le p\ll p_2\sim 0$ and $k_{\max }+k_{\min }\sim k+k_2$. If $l_2>(1-\delta )m$ (cf. (7.2)) it follows from Lemma 5.15 with $\left\lvert S\right\rvert \lesssim 2^{k+\frac{p}{2}}$:

$$\begin{aligned} 2^{4k^+}2^{-\frac{k^-}{2}}2^{-\frac{p}{2}}\left\Vert P_{k,p}\mathcal {B}_\mathfrak {m}(f_1,f_2)\right\Vert _{L^2}&\lesssim 2^{-\frac{p}{2}}2^m 2^{2k+\frac{p}{2}}\left\Vert f_1\right\Vert _B2^{-(1+\beta )l_2}\left\Vert f_2\right\Vert _X\lesssim 2^{-\frac{\beta }{2}m}\varepsilon ^2. \end{aligned}$$

This concludes **Case B**.

**Case C: Gap in** *p* **with** $p_{\min }\ll p_{\max }\ll 0$. Then $\left\lvert \Phi \right\rvert \ge \frac{1}{10}$ and we can do a decomposition $\mathfrak {m}=\mathfrak {m}^{res}+\mathfrak {m}^{nr}$ as presented in Sect. 5.7 with $\lambda =\frac{1}{100}$. In this case, $\mathfrak {m}^{res}=0$, and thus $\mathfrak {m}=\mathfrak {m}^{nr}$ with

$$\begin{aligned} \left\Vert P_{k,p}\mathcal {B}_\mathfrak {m}(f_1,f_2)\right\Vert _{L^2}\lesssim \left\Vert P_{k,p}\mathcal {Q}_{\mathfrak {m}\Phi ^{-1}}(f_1,f_2)\right\Vert _{L^2}&+\left\Vert P_{k,p}\mathcal {B}_{\mathfrak {m}\Phi ^{-1}}(\partial _tf_1,f_2)\right\Vert _{L^2}\\&+\left\Vert P_{k,p}\mathcal {B}_{\mathfrak {m}\Phi ^{-1}}(f_1,\partial _tf_2)\right\Vert _{L^2}. \end{aligned}$$

We prove the claim (7.1) for the last two terms with Lemma 5.15 with $\left\lvert S\right\rvert \lesssim 2^{k+\frac{p}{2}}$ and Lemmas A.3 and 6.1:

$$\begin{aligned} 2^{4k^+} 2^{-\frac{k^-}{2}}2^{-\frac{p}{2}}\left\Vert P_{k,p}\mathcal {B}_{\mathfrak {m}\Phi ^{-1}}(\partial _tf_1,f_2)\right\Vert _{L^2}&\lesssim 2^{\frac{9}{2}k^+}2^{-\frac{k}{2}}2^{-\frac{p}{2}}2^{k+\frac{p}{2}}2^{k}2^m\left\Vert \partial _tf_1\right\Vert _{L^2}\left\Vert f_2\right\Vert _{L^2}\\&\lesssim 2^{\frac{9}{2}k^+}2^{\frac{3}{2}k}2^m2^{(-\frac{3}{4}+2\delta ) m}\varepsilon ^3\\&\lesssim 2^{(\frac{1}{4}+\frac{5}{2}\delta )m}\varepsilon ^3. \end{aligned}$$

The term containing $\partial _tf_2$ is bounded analogously. For the boundary term, by Lemma 5.15 with $\left\lvert S\right\rvert \lesssim 2^{k+\frac{p}{2}}$ we obtain

$$\begin{aligned} 2^{4k^+}2^{-\frac{k^-}{2}}2^{-\frac{p}{2}}\left\Vert P_{k,p}\mathcal {Q}_{\mathfrak {m}\Phi ^{-1}}(f_1,f_2)\right\Vert _{L^2}&\lesssim 2^{\frac{9}{2}k^+}2^{-\frac{k}{2}}2^{-\frac{p}{2}}2^{k+\frac{p}{2}}2^{-4k_1^+}\left\Vert f_1\right\Vert _B 2^{-4k_2^+}\left\Vert f_2\right\Vert _B\\&\lesssim 2^{\delta m}\varepsilon ^2. \end{aligned}$$

**Case D: No gaps with** $p_1\sim p_2\sim p$**.** Assume without loss of generality that $b_1\le b_2$, that is $f_2$ has more vector fields and we can apply Proposition 3.2 on $f_1$ and Lemma 3.4 on $f_2$. With the decomposition $P_{k_1,p_1}e^{it\Lambda }f_1=I_{k_1,p_1}(f_1)+II_{k_1,p_1}(f_1)$ the following decay bounds hold:

$$\begin{aligned} \left\| I_{k_1,p_1}(f_1)\right\| _{L^\infty }&\lesssim 2^{\frac{3}{4}k_1}2^{-\frac{15}{4}k_1^+}2^{-p}2^{(-1+\delta )m}\left\| f_1\right\| _D, \\ \left\| II_{k_1,p_1}(f_1)\right\| _{L^2}&\lesssim 2^{-4k_1^+}2^{-\frac{p}{2}}2^{-\frac{m}{2}}\left\| f\right\| _D,\\\Vert P_{k_2,p_2}e^{it\Lambda }f_2\Vert _{L^\infty }&\lesssim 2^{\frac{3}{4}k_2}2^{-3k_2^+}2^{(-\frac{1}{2}+\kappa )m}\varepsilon . \end{aligned}$$

Then, for the *B*-norm,

$$\begin{aligned}&2^{4k^+}2^{-\frac{k^-}{2}}2^{-\frac{p}{2}}\left\| P_{k,p}\mathcal {B}_\mathfrak {m}(f_1,f_2)\right\| _{L^2}\\  &\quad \lesssim 2^{\frac{9}{2}k^{+}}2^m2^{\frac{p}{2}+\frac{k}{2}}\Big [\left\| I_{k_1,p_1}(f_1)\right\| _{L^\infty }\left\| P_{k_2,p_2}f_2\right\| _{L^2}+\left\| II_{k_1,p_1}(f_1)\right\| _{L^2}\left\| e^{it\Lambda }P_{k_2,p_2}f_2\right\| _{L^\infty }\Big ]\\  &\quad \lesssim 2^{5k^{+}}2^{\frac{p}{2}}2^m\Big [2^{-3k_1^+}2^{-p}2^{(-1+\delta )m}2^{-4k_2^+}2^{\frac{k_2^-}{2}}2^{\frac{p}{2}} +2^{-4k_1^+}2^{-\frac{p}{2}}2^{-\frac{m}{2}}2^{\frac{3}{4}k_2}2^{-3k_2^+}2^{(-\frac{1}{2}+\kappa ) m}\Big ]\varepsilon ^2\\  &\quad \lesssim 2^{4k^+}(2^{\delta m}+2^{\kappa m})\varepsilon ^2\\  &\quad \lesssim 2^{\beta m}\varepsilon ^2, \end{aligned}$$

where $\kappa \ll \beta$ in Lemma 3.4. This is an admissible contribution and finishes the proof of the proposition. $\square$

## Bounds on the *X*-Norm

We prove the *X*-norm bounds in two steps depending on the size of *l* relative to *m*.

### *X*-Norm Bounds for $l>(1+\delta )m$

#### Proposition 8.1

In the setting of Proposition 2.7, and in particular under the bootstrap assumptions (2.26), the following holds true: For $\mathfrak {m}\in \big \{\mathfrak {m}_0,\mathfrak {m}_\pm ^{\mu \nu }|\mu ,\nu \in \{+,-\}\big \}$, $t\in [2^m,2^{m+1}[\cap [0,T]$ and $\delta =2M^{-\frac{1}{2}}$, there holds that

$$\begin{aligned} \sup _{\begin{array}{c} k,l,p\\ l+p\ge 0, l>(1+\delta )m \end{array}}2^{4k^+}2^{(1+\beta )l}2^{\beta p}2^{\frac{p}{2}}\left\Vert P_{k,p}R_l\mathcal {B}_\mathfrak {m}(F_1,F_2)\right\Vert _{L^2}\lesssim 2^{(\frac{1}{2}-\frac{\delta }{10}) m }\varepsilon ^2+2^{(\frac{1}{4}+7\beta )m}\varepsilon ^3, \end{aligned}$$

where $$F_i\in \{S^{b_i}\mathcal {Z}_{\pm },S^{b_i}\Theta \}$$, $$0\le b_1+b_2\le N$$, $$i=1,2$$.

This result corresponds to an approximate “finite speed of propagation” and implies that the time parameter *m* is the largest one in the problem. The proof of Proposition 8.1 is structured in two parts. In **Part 1**, we assume additionally that $l+p<\delta m$. Here we note that the weight in the norm is bounded by a factor $2^{-p/2}$, which is similar to the *B*-norm. Thus, the claim (8.1) follows via energy estimates, integration by parts along *S* and normal forms. In **Part 2**, where $l+p>\delta m$, to overcome the “large” parameter *l* we invoke the properties of *W* on bilinear terms, as established in Lemmas 5.4 and 5.9. Combined with the iterated action of *W* on bilinear terms as developed in Lemma 5.12 to “gain” negative *l* parameters in the bilinear terms $\mathcal {B}_{\mathfrak {m}}$, we can restrict the possible size of *l* in terms of other parameters. In the remaining cases we can rely on the well-established tools: integration by parts, normal forms and linear decay.

#### Proof

We split the proof in two main parts.

**Part 1:** $l+p<\delta m$**.** Similarly to the proof of Proposition 7.1 we want to show that the energy estimates that we get from the bootstrap assumption allow us to restrict the range of the localisation parameters.

**Case A: Simple cases.** Using the set size estimate Lemma 5.15 with $\left\lvert S\right\rvert \lesssim 2^{\frac{k_{\min }+k+p}{2}}$ and the bootstrap assumption (2.26) we have

$$\begin{aligned}&2^{4k^+}2^{(1+\beta )l}2^{\beta p}2^{\frac{p}{2}}\left\Vert P_{k,p}R_l\mathcal {B}_{\mathfrak {m}}(F_1,F_2)\right\Vert _{L^2} \\&\quad \lesssim 2^{4k^+}2^{(1+\beta )l}2^{\beta p}2^{\frac{p}{2}}2^m\left\lvert S\right\rvert \left\Vert \mathfrak {m}\chi \right\Vert _{L^\infty }\left\Vert P_{k_1,p_1}F_1\right\Vert _{L^2}\left\Vert P_{k_2,p_2}F_2\right\Vert _{L^2}\\&\quad \lesssim 2^{(1+2\delta )m}2^{\frac{k_{\min }}{2}}2^{-(N_0-5)(k_1^++k_2^+)}\left\Vert P_{k_1}F_1\right\Vert _{H^{N_0}}\left\Vert P_{k_2}F_2\right\Vert _{H^{N_0}} \end{aligned}$$

Therefore, we can assume $-4m\le k,k_1,k_2\le \delta _0 m$, with $\delta _0:=2N_0^{-1}\ll \delta$. Localizing further in $p_i,\; l_i$ and letting $f_i=P_{k_i,p_i}R_{l_i}F_i$, we can restrict the $l_i,\; p_i$ parameters using Lemma 5.15 with $\left\lvert S\right\rvert \lesssim 2^{k+\frac{p}{2}}$, and the bootstrap assumption (2.26):

$$\begin{aligned}&2^{4k^+}2^{(1+\beta )l}2^{\beta p}2^{\frac{p}{2}}\left\Vert P_{k,p}R_l\mathcal {B}_\mathfrak {m}(F_1,F_2)\right\Vert _{L^2} \\&\quad \lesssim 2^{(1+(1+\beta )\delta +5\delta _0)m}2^{-\frac{l_1}{2}-\frac{l_2}{2}}2^{-\frac{l_1+p_1}{2}}2^{-\frac{l_2+p_2}{2}}\left\Vert f_1\right\Vert _X\left\Vert f_2\right\Vert _X\\&\quad \lesssim 2^{(1+2\delta )m}2^{-\frac{l_1}{2}-\frac{l_2}{2}}2^{-\frac{l_1+p_1}{2}}2^{-\frac{l_2+p_2}{2}}\varepsilon ^2. \end{aligned}$$

Hence, the *X*-norm remains bounded if $\max \{l_1,l_2\}\ge 4m$. Thus, analogous to **Case A** in the proof of Lemma 7.1 it suffices to prove

8.1 $$\begin{aligned} \sup _{k,l+p<\delta m,l>(1+\delta )m} 2^{4k^+}2^{(1+\beta )l}2^{\beta p}2^{\frac{p}{2}}\left\Vert P_{k,p}R_l\mathcal {B}_{\mathfrak {m}}(f_1,f_2)\right\Vert _{L^2}\lesssim 2^{3\delta m}\varepsilon ^2+ 2^{(\frac{1}{4}+4\delta )m}\varepsilon ^3, \end{aligned}$$

for the following localisation parameters

$$\begin{aligned}&-4m<k,k_i<\delta _0 m ,  &   -p_i\le l_i\le 4m,  &   -4m\le p_i\le 0,  &   i=1,2. \end{aligned}$$

Observe that $2^p\ll 1$ since $l+p<\delta m$ and $l>(1+\delta )m$. Hence we have the following two cases to consider:

**Case B:** $2^{p_1}+2^{p_2}\ll 1$**.** Then $\left\lvert \Phi \right\rvert > \frac{1}{10}$ and we can do a splitting of the multiplier in the resonant and non-resonant parts as in Sect. 5.7 with $\lambda =\frac{1}{100}$. Observe that $\mathfrak {m}^{res}=0$ and so $\mathfrak {m}=\mathfrak {m}^{nr}$ with

$$\begin{aligned} \left\Vert P_{k,p}R_l\mathcal {B}_{\mathfrak {m}}(f_1,f_2)\right\Vert _{L^2}&\lesssim \left\Vert P_{k,p}R_l\mathcal {Q}_{\mathfrak {m}\Phi ^{-1}}(f_1,f_2)\right\Vert _{L^2}+\left\Vert P_{k,p}R_l\mathcal {B}_{\mathfrak {m}\Phi ^{-1}}(\partial _tf_1,f_2)\right\Vert _{L^2}\\&\quad + \left\Vert P_{k,p}R_l\mathcal {B}_{\mathfrak {m}\Phi ^{-1}}(f_1,\partial _tf_2)\right\Vert _{L^2}. \end{aligned}$$

We bound each term in the decomposition with Lemmas 5.5, 6.1, A.3 and 5.15 with $\left\lvert S\right\rvert \lesssim 2^{k+\frac{p}{2}}$:

$$\begin{aligned} 2^{4k^+}2^{(1+\beta )l}2^{\beta p}2^{\frac{p}{2}}\left\Vert P_{k,p}R_l\mathcal {B}_{\mathfrak {m}\Phi ^{-1}}(\partial _tf_1,f_2)\right\Vert _{L^2}&\lesssim 2^{4k^+}2^{(1+(1+\beta )\delta ) m}2^{2k}\left\Vert \partial _tf_1\right\Vert _{L^2}\left\Vert f_2\right\Vert _{L^2}\\&\lesssim 2^{(1+(1+\beta )\delta +6\delta _0)m}2^{(-\frac{3}{4}+2\delta )m}\varepsilon ^3\\&\lesssim 2^{(\frac{1}{4}+4\delta ) m}\varepsilon ^3. \end{aligned}$$

The same holds by symmetry for the last term in the splitting above. Now it remains to estimate the boundary term. From Lemma 5.15 with $\left\lvert S\right\rvert \lesssim 2^{k+\frac{p}{2}}$ and (2.26) we obtain

$$\begin{aligned} 2^{4k^+}2^{(1+\beta )l}2^{\beta p}2^{\frac{p}{2}}\left\| P_{k,p}\mathcal {Q}_{\mathfrak {m}\Phi ^{-1}}(f_1,f_2)\right\| _{L^2}&\lesssim 2^{4k^+}2^{(1+\beta )(l+p)}2^{2k}2^{-4k_1^+-4k_2^+}\left\| f_1\right\| _B\left\| f_2\right\| _B\\  &\lesssim 2^{3\delta m}\varepsilon ^2, \end{aligned}$$

which gives the claim and closes **Case B**.

**Case C:** $\max \{2^{p_1},2^{p_2}\}\sim 1$**.** Assume w.l.o.g. that $p_2\le p_1$, so that $p_1=p_{\max }$ and then $k_{\max }\in \{k_2,k\}$. Thus by Proposition 5.8 there holds $\left\lvert \sigma \right\rvert \sim 2^{k_1+k}$. Integration by parts along $S_\eta$ gives the claim analogously to (7.2)–(7.3) if $l_1+k_2-k\le (1-\delta )m$ and $\delta =2M^{-\frac{1}{2}}$. Otherwise, if

$$\begin{aligned} -l_1-k_2+k<-(1-\delta )m, \end{aligned}$$

by Lemma 5.15 with $\left\lvert S\right\rvert \lesssim 2^{k+\frac{p}{2}}$ and the bootstrap assumption (2.26) there holds:

$$\begin{aligned}&2^{4k^+}2^{(1+\beta )l}2^{\beta p}2^{\frac{p}{2}}\left\Vert P_{k,p}R_l\mathcal {B}_{\mathfrak {m}}(f_1,f_2)\right\Vert _{L^2}\\&\quad \lesssim 2^{4k^+}2^{(1+\beta )(l+p)}2^m2^{2k}2^{-4k_1^+}2^{-(1+\beta )l_1}\left\Vert f_1\right\Vert _X2^{-4k_2^+}\left\Vert f_2\right\Vert _B\\&\quad \lesssim 2^{(-\beta +(1+\beta )\delta )m}2^{2k}2^{4(k^+-k_1^+-k_2^+)}2^{(1+\beta )(k_2-k)}\varepsilon ^2\\&\quad \lesssim 2^{-\frac{\beta }{2}m}\varepsilon ^2, \end{aligned}$$

since $\delta _0\ll \delta \ll \beta$. This finishes the proof of **Part 1**.

**Part 2:** $$l+p>\delta m$$**.**

**Case A: Simple cases.** As in **Part 1**, we can restrict the localisation parameters. Using Lemma 5.15, the energy estimates and $l>(1+\delta )m$, we can bound

$$\begin{aligned} 2^{4k^+}2^{(1+\beta )l}2^{\beta p}2^{\frac{p}{2}}\left\Vert P_{k,p}R_l\mathcal {B}_\mathfrak {m}(f_1,f_2)\right\Vert _{L^2}&\lesssim 2^{5\max \{k_1^+,k_2^+\}}2^{(2+\beta )l}2^{-\delta m}2^{k_{\min }}2^{-N_0k_1^+-N_0k_2^+}\varepsilon ^2. \end{aligned}$$

Hence we obtain the claim if $k_{\min }\le -3l$ or if $k_{\max }\ge \delta _0 l$, with $\delta _0=3N_0^{-1}$. Localising further in $p_i,l_i$, $i=1,2$ and estimating using the $X-$norm, we obtain the claim if $\max \{l_1,l_2\}\ge (4+4\beta )l$:

$$\begin{aligned} 2^{4k^+}2^{(1+\beta )l}2^{\beta p}2^{\frac{p}{2}}\left\| P_{k,p}R_l\mathcal {B}_\mathfrak {m}(f_1,f_2)\right\| _{L^2}&\lesssim 2^{(2+\beta )l}2^{-\delta m}2^{k+k_{\min }}2^{-\frac{l_1+l_2}{2}}\left\| f_1\right\| _X\left\| f_2\right\| _X\\  &\lesssim 2^{-\delta m}2^{(-\beta +2\delta _0) l}\varepsilon ^2. \end{aligned}$$

In the following, we will prove

$$\begin{aligned} \sup _{k,l+p>\delta m,l>(1+\delta )m} 2^{4k^+}2^{(1+\beta )l}2^{\beta p}2^{\frac{p}{2}}\left\Vert P_{k,p}R_l\mathcal {B}_\mathfrak {m}(f_1,f_2)\right\Vert _{L^2}\lesssim 2^{(\frac{1}{2}-\frac{\delta }{8}) m}\varepsilon ^2+2^{(\frac{1}{4}+6\beta )m}\varepsilon ^3, \end{aligned}$$

for the parameters

$$\begin{aligned} -3l\le k,k_i\le \delta _0l,  &   -(4+4\beta )l\le p_i \le 0,  &   -p_i\le l_i\le (4+4\beta )l,  &   i=1,2. \end{aligned}$$

**Case B.** We employ Lemma 5.12 with $N\in \mathbb {N}$ such that $N\delta ^2>(2+\beta )$. From this we see that if

8.2 $$\begin{aligned} 2^m(2^p+2^{k-k_i}2^{p_i})+2^{k-k_i+l_i}< 2^{(1-\delta ^2)l}, \qquad i=1 \text { or }i=2, \end{aligned}$$

we obtain an acceptable bound on the *X*-norm:

$$\begin{aligned} 2^{4k^+}2^{(1+\beta )l}2^{\beta p}2^{\frac{p}{2}}\left\| P_{k,p}R_l\mathcal {B}_\mathfrak {m}(f_1,f_2)\right\| _{L^2}&\lesssim 2^{6\delta _0m}2^{(2+\beta )l}[ 2^{-N\delta ^2l}2^{-N\delta m} +2^{-3l}]\varepsilon ^2\\  &\lesssim 2^{-\delta ^2l}2^{5\delta m}\varepsilon ^2. \end{aligned}$$

Assume w.l.o.g. that $k_2\le k_1$ and recall $2^{m}\lesssim 2^{l-\delta m}$, then condition (8.2) (and thus the claim) holds if

8.3 $$\begin{aligned} l_1\le (1-\delta ^2)l \quad \text {or} \quad k-k_2 +\max \{m+p_2,l_2\}\le (1-\delta ^2)l. \end{aligned}$$

Otherwise if (8.3) doesn’t hold, we distinguish two further cases: $m+p_2\le l_2$ or $m+p_2>l_2$.

**B.1:** $m+p_2\le l_2$**,** $l_1>(1-\delta ^2)l$ **and** $k-k_2+l_2>(1-\delta ^2)l$**.** We proceed with the by now standard scheme of proof.

**B.1(a): Gap in** *p***:** $p_{\min }\ll p_{\max }$**.** Based on Lemma 5.13 and with the constraint $k_2\le k_1$, we have the following cases:

**B.1(a.1):** $p_{\min }= p\ll p_{\max }$**.** From Lemma 5.13 we have two settings for the $k,k_1,k_2$ parameters. If $2^{k_1}\sim 2^{k_2}$, then $p\ll p_1\sim p_2=p_{\max }$ and $2^{k}\lesssim 2^{k_2}$. From Lemma 5.15 with $\left\lvert S\right\rvert \lesssim 2^{k_2+\frac{p_2}{2}}$ we bound

$$\begin{aligned}&2^{4k^+}2^{(1+\beta )l}2^{\beta p}2^{\frac{p}{2}}\left\Vert P_{k,p}R_l\mathcal {B}_\mathfrak {m}(f_1,f_2)\right\Vert _{L^2} \\&\quad \lesssim 2^{-\delta m}2^{(2+\beta )l}2^{(2+\beta )p_2}2^{2k_2}2^{-(1+\beta )(l_1+l_2)}2^{-(1+2\beta )p_2}\left\Vert f_1\right\Vert _X\left\Vert f_2\right\Vert _X\\&\quad \lesssim 2^{-\delta m}2^{(-\beta +3\delta ^2)l}2^{(1-\beta )k_2}2^{(1+\beta )k}\varepsilon ^2\\&\quad \lesssim 2^{-\frac{\delta }{2}m}2^{-\frac{\beta }{2}l}\varepsilon ^2. \end{aligned}$$

If on the other hand $2^{k_2}\ll 2^{k_1}\sim 2^{k}$, then $p\le p_1\ll p_2$ and moreover $2^{m}\lesssim 2^{\frac{l_2-p_2}{2}+\frac{l-\delta m}{2}}$. Thus from Lemma 5.15 with $\left\lvert S\right\rvert \lesssim 2^{k_2+\frac{p_2}{2}}$ and using the *X*-norms on $f_1,\,f_2$ we have

$$\begin{aligned}&2^{4k^+}2^{(1+\beta )l}2^{\beta p}2^{\frac{p}{2}}\left\Vert P_{k,p}R_l\mathcal {B}_\mathfrak {m}(f_1,f_2)\right\Vert _{L^2} \\&\quad \lesssim 2^{-\frac{\delta }{2}m}2^{(\frac{3}{2}+\beta )l}2^{k+k_2}2^{\frac{3}{2}p_{2}} 2^{-(1+\beta )l_1}2^{-4k_2^+}2^{-(\frac{1}{2}+\beta )l_2}2^{-(1+\beta ) p_2}\varepsilon ^2\\&\quad \lesssim 2^{-\frac{\delta }{2}m}2^{(-\beta +2\delta ^2)l}2^{(\frac{1}{2}-\beta )k_2}2^{(\frac{1}{2}+\beta )k}\varepsilon ^2\\&\quad \lesssim 2^{-\frac{\delta }{4}m}2^{-\frac{\beta }{2}l}\varepsilon ^2, \end{aligned}$$

since $$\delta _0\ll \delta \ll \beta $$.

**B.1(a.2):** $p_{\min }\sim p_1 \ll p_{\max }$**.** If $2^{k}\sim 2^{k_2}$, then $2^p\sim 2^{p_2}$ and so $p_1\ll p \sim p_2$. Moreover, ${-l_2}< {-(1-\delta ^2)l}$ and by Lemma 5.15 with $\left\lvert S\right\rvert \lesssim 2^{k_1+\frac{p_1}{2}}$ we obtain

$$\begin{aligned}&2^{4k^+}2^{(1+\beta )l}2^{\beta p}2^{\frac{p}{2}}\left\Vert P_{k,p}R_l\mathcal {B}_\mathfrak {m}(f_1,f_2)\right\Vert _{L^2}\\&\quad \lesssim 2^m 2^{(1+\beta )l}2^{(\frac{3}{2}+\beta )p_2}2^{k+k_1+\frac{p_1}{2}}2^{-4k_1^+}2^{-l_1-\frac{p_1}{2}}\left\Vert f_1\right\Vert _X 2^{-(1+\beta )l_2}2^{-(\frac{1}{2}+\beta )p_2}\left\Vert f_2\right\Vert _X\\&\quad \lesssim 2^{(\beta +\delta _0) m}2^{\beta p_2}2^{(-\beta +2\delta ^2)l}\varepsilon ^2. \end{aligned}$$

Next, if $2^{k}\ll 2^{k_2}\sim 2^{k_1}$, then $p_1\le p_2\ll p$ and $2^{p+k}\lesssim 2^{p_2+k_2}$. Moreover, ${-l_2}<{-(1-\delta ^2)l}$ and we estimate with $\left\lvert S\right\rvert \lesssim 2^{k_2+\frac{p_1}{2}}$:

$$\begin{aligned}&2^{4k^+}2^{(1+\beta )l}2^{\beta p}2^{\frac{p}{2}}\left\Vert P_{k,p}R_l\mathcal {B}_\mathfrak {m}(f_1,f_2)\right\Vert _{L^2}\\&\quad \lesssim 2^{ m}2^{(1+\beta )l}2^{(\frac{3}{2}+\beta )p_2}2^{k}2^{k_2+\frac{p_1}{2}} 2^{-4k_2^+}2^{-l_1}2^{-\frac{p_1}{2}} \left\Vert f_1\right\Vert _X2^{-(1+\beta )l_2}2^{-(\frac{1}{2}+\beta )p_2}\left\Vert f_2\right\Vert _X\\&\quad \lesssim 2^{m}2^{(-1+3\delta ^2)l}2^{p_2}\varepsilon ^2\\&\quad \lesssim 2^{(\beta -\frac{\delta }{2}) m}2^{(-\beta +3\delta ^2)l}\varepsilon ^2. \end{aligned}$$

Finally, if $2^{k_2}\ll 2^{k}\sim 2^{k_1}$, then $p_1\le p\ll p_2$ and we obtain with $\left\lvert S\right\rvert \lesssim 2^{\frac{k+k_2}{2}+\frac{p_1}{2}}$:

$$\begin{aligned}&2^{4k^+}2^{(1+\beta )l}2^{\beta p}2^{\frac{p}{2}}\left\Vert P_{k,p}R_l\mathcal {B}_\mathfrak {m}(f_1,f_2)\right\Vert _{L^2}\\&\quad \lesssim 2^{(\frac{1}{2}-\frac{\delta }{2})m}2^{(\frac{3}{2}+\beta )l}2^{\frac{3}{2}k}2^{k_{2}+\frac{p_{1}}{2}}2^{-l_1}2^{-\frac{p_1}{2}}\varepsilon 2^{-\frac{2}{3}l_2}2^{-\frac{1}{3}p_2}\left\Vert f_2\right\Vert _X^{\frac{2}{3}} 2^{\frac{k_2^-}{6}}2^{\frac{p_2}{6}}\left\Vert f_2\right\Vert _B^{\frac{1}{3}}\\&\quad \lesssim 2^{(\frac{1}{2}-\frac{\delta }{2})m}2^{(-\frac{1}{6}+\beta +2\delta ^2)l}2^{\frac{3}{2}k}2^{\frac{2}{3}k_2}2^{-\frac{2}{3}(k_2-k)}\varepsilon ^2\\&\quad \lesssim 2^{(\frac{1}{2}-\frac{\delta }{4})m}2^{(-\frac{1}{6}+2\beta )l}\varepsilon ^2, \end{aligned}$$

which is an acceptable contribution.

**B.1(a.3):** $p_{\min }= p_2\ll p_{\max }$**.** Then by Lemma 5.13 we have two possibilities for the parameters $k,k_1,k_2$. First, if $2^k\sim 2^{k_1}$, then $p_2\ll p\sim p_1$ and $2^{-l_2}\lesssim 2^{-(1-\delta ^2)l}$. Using Lemma 5.15 with $\left\lvert S\right\rvert \lesssim 2^{k_2+\frac{p_2}{2}}$ and the *X*-norms of $f_1,f_2$, we have

$$\begin{aligned}&2^{4k^+}2^{(1+\beta )l}2^{\beta p}2^{\frac{p}{2}}\left\Vert P_{k,p}R_l\mathcal {B}_\mathfrak {m}(f_1,f_2)\right\Vert _{L^2} \\&\quad \lesssim 2^{(\beta -\frac{\delta }{2})m}2^{2l}2^{(\frac{1}{2}+\beta )p_1}2^{\frac{p_{2}}{2}} 2^{-(1+\beta )l_1}2^{-(\frac{1}{2}+\beta )p_1}2^{-l_2}2^{-\frac{p_2}{2}}\varepsilon ^2\\&\quad \lesssim 2^{(\beta -\frac{\delta }{2}) m}2^{(-\beta +3\delta ^2)l}\varepsilon ^2. \end{aligned}$$

If on the other hand $2^k\ll 2^{k_1}\sim 2^{k_2}$, then $2^{p_1}\ll 2^p$ and hence $p_2\le p_1\ll p$ and $2^{p+k}\lesssim 2^{p_1+k_2}$. With $\left\lvert S\right\rvert \lesssim 2^{k_2+\frac{p_2}{2}}$

$$\begin{aligned}&2^{4k^+}2^{(1+\beta )l}2^{\beta p}2^{\frac{p}{2}}\left\Vert P_{k,p}R_l\mathcal {B}_\mathfrak {m}(f_1,f_2)\right\Vert _{L^2} \\&\quad \lesssim 2^{(\beta -\frac{\delta }{2})m}2^{2l}2^{(\frac{1}{2}+\beta )p}2^{k+k_2}2^{-(1+\beta )l_1}2^{-(\frac{1}{2}+\beta )p_1}2^{-4k_2^+}2^{-l_2}\varepsilon ^2\\&\quad \lesssim 2^{(\beta -\frac{\delta }{2})m}2^{(-\beta +3\delta ^2)l}2^{(\frac{1}{2}+\beta )(k_2-k)}2^{2k}2^{-4k_2^+}\\&\quad \lesssim 2^{(\beta -\frac{\delta }{2}) m}2^{(-\beta +3\delta ^2)l}\varepsilon ^2. \end{aligned}$$

**B.1(b): No gap in** *p***:** $p\sim p_1\sim p_2$**.** With Lemma 5.15 and $m\le l_2-p$ we obtain

$$\begin{aligned}&2^{4k^+}2^{(1+\beta )l}2^{\beta p}2^{\frac{p}{2}}\left\Vert P_{k,p}R_l\mathcal {B}_\mathfrak {m}(f_1,f_2)\right\Vert _{L^2} \\&\quad \lesssim 2^{m}2^{(1+\beta )l}2^{(2+\beta )p}2^{k+k_{\min }} 2^{-(1+\beta )l_1}2^{-(1+\beta )l_2}2^{-(1+2\beta )p}\varepsilon ^2\\&\quad \lesssim 2^{\beta m}2^{(1-\beta )p}2^{2\delta ^2l}2^{(1-\beta )p}2^{k+k_{\min }}2^{-2\beta l_2}\varepsilon ^2\\&\quad \lesssim 2^{2\beta m}2^{-\beta l }\varepsilon ^2. \end{aligned}$$

This finishes **Case B.1**.

**B.2:** $l_2<m+p_2$**,** $l_1>(1-\delta ^2)l$ **and** $k-k_2+m+p_2>(1-\delta ^2)l$**.** This is the other possibility if (8.3) doesn’t hold. Here we have

8.4 $$\begin{aligned}&(1+\delta )m<l<(1+2\delta ^2)(k-k_2+m+p_2), \nonumber \\  &-l_1<-(1-\delta ^2)l, \qquad -p_2-k+k_2<-\frac{\delta }{2}m. \end{aligned}$$

**B.2(a): No gaps with** $p\sim p_1\sim p_2$**.** An $L^2-L^\infty$ estimate using Lemma 3.4 on $f_2$ and (8.4) gives:

$$\begin{aligned}&2^{4k^+}2^{(1+\beta )l}2^{\beta p}2^{\frac{p}{2}}\left\Vert P_{k,p}R_l\mathcal {B}_\mathfrak {m}(f_1,f_2)\right\Vert _{L^2} \\&\quad \lesssim 2^{m}2^{(1+\beta )l}2^{(\frac{3}{2}+\beta )p}2^{k}2^{-(1+\beta )l_1}2^{-(\frac{1}{2}+\beta )p}2^{\frac{3}{4}k_2}2^{(-\frac{1}{2}+\kappa )m}\varepsilon ^2\\&\quad \lesssim 2^{(\frac{1}{2}+\kappa )m}2^{2\delta ^2(1+2\delta ^2)(k-k_2+m+p)}2^{p}2^{k+\frac{3}{4}k_2}\varepsilon ^2\\&\quad \lesssim 2^{(\frac{1}{2}+\kappa +3\delta ^2)m}2^{(\frac{3}{4}-3\delta ^2)k_2}2^{(1+3\delta ^2)k}\varepsilon ^2\\&\quad \lesssim 2^{(\frac{1}{2}-\frac{\delta }{8})m}\varepsilon ^2, \end{aligned}$$

since $\delta _0\ll \delta ^2\ll \delta$ and we can choose $\kappa \ll \delta ^2\ll \beta$. Note that in the last step we used the third condition in (8.4) on $k_2$.

**B.2(b): Gap in** *p***:** $$p_{\min }\ll p_{\max }$$**.**

We can integrate by parts along $S_{\xi -\eta }$ via Lemma 5.10(1) and using (8.4), obtain the claim if

8.5 $$\begin{aligned} \max \{k_2-k-p_2-p_{\max } ,-p_2-p_{\max }+l_2\}\le (1-\delta )m. \end{aligned}$$

Assume now that (8.5) does not hold. Still (8.4) holds and we proceed with two cases depending on which term on the right-hand side of (8.5) is the largest. If $k_2-k-p_2-p_{\max }> (1-\delta )m$, then we obtain with $\left\lvert S\right\rvert \lesssim 2^{\frac{k+k_2}{2}+\frac{p_2}{2}}$ in Lemma 5.15:

$$\begin{aligned}&2^{4k^+}2^{(1+\beta )l}2^{\beta p}2^{\frac{p}{2}}\left\Vert P_{k,p}R_l\mathcal {B}_\mathfrak {m}(f_1,f_2)\right\Vert _{L^2} \\&\quad \lesssim 2^{m}2^{(1+\beta )l}2^{(\frac{3}{2}+\beta )p_{\max }}2^{\frac{3}{2}k}2^{\frac{k_2}{2}+\frac{p_{2}}{2}}2^{-\frac{l_1}{2}}\left\Vert f_1\right\Vert _X2^{\frac{k_2}{2}}2^{\frac{p_2}{2}}\left\Vert f_2\right\Vert _B\\&\quad \lesssim 2^{m}2^{(\frac{1}{2}+\beta +\frac{\delta ^2}{2})(1+2\delta ^2)(k-k_2+m+p_2)}2^{\frac{3}{2}k+k_2}2^{\frac{3}{2}p_{\max }+p_2}\varepsilon ^2\\&\quad \lesssim 2^{(\frac{3}{2}+\beta +2\delta ^2)m}2^{(2+\beta +2\delta ^2)k}2^{(\frac{1}{2}-\beta -2\delta ^2)k_2}2^{(\frac{3}{2}+\beta )(p_2+p_{\max })}\varepsilon ^2\\&\quad \lesssim 2^{\delta m}\varepsilon ^2. \end{aligned}$$

If, on the other hand, $-p_2-p_{\max }+l_2> (1-\delta )m$, we have two settings.

**B.2(b.1): Gap in** *p* **with** $p_{\max }\sim 0$**.** If $2^{p_1}\sim 1$, then by Lemma 5.15 with $\left\lvert S\right\rvert \lesssim 2^{k_2+\frac{p_2}{2}}$ and (8.4), we obtain

$$\begin{aligned}&2^{4k^+}2^{(1+\beta )l}2^{\beta p}2^{\frac{p}{2}}\left\Vert P_{k,p}R_l\mathcal {B}_{\mathfrak {m}} (f_1,f_2)\right\Vert _{L^2} \\&\quad \lesssim 2^m2^{(1+\beta )l}2^{k}2^{k_2+\frac{p_{2}}{2}} 2^{-(1+\beta )l_1}\left\Vert f_1\right\Vert _X2^{-l_2}2^{-\frac{p_2}{2}}\left\Vert f_2\right\Vert _X\\&\quad \lesssim 2^{\delta m} 2^{3\delta ^2(k-k_2+m+p_2)}2^{k+k_2}2^{-p_2}\varepsilon ^2\\&\quad \lesssim 2^{(\delta +3\delta ^2)m}2^{(1+3\delta ^2)k}2^{(1-3\delta ^2)k_2}2^{-(1-3\delta ^2)p_2}\varepsilon ^2\\&\quad \lesssim 2^{2\delta m}\varepsilon ^2. \end{aligned}$$

Now let $2^{p_2}\sim 1$, then from (8.4) we have $-l_2<-(1-\delta )m$. Using Lemma 5.15 with $\left\lvert S\right\rvert \lesssim 2^{\frac{k_2+k_1+p_1}{2}}$ we obtain

$$\begin{aligned}&2^{4k^+}2^{(1+\beta )l}2^{\beta p}2^{\frac{p}{2}}\left\Vert P_{k,p}R_l\mathcal {B}_{\mathfrak {m}} (f_1,f_2)\right\Vert _{L^2}\\&\quad \lesssim 2^m2^{(1+\beta )l}2^{k}2^{\frac{k_2+k_1+p_{1}}{2}} 2^{-l_1}2^{-\frac{p_1}{2}}2^{-(1+\beta )l_2}\varepsilon ^2\\&\quad \lesssim 2^{(-\beta +2\delta )m}2^{(\beta +2\delta ^2)(k-k_2+m)}2^{k+\frac{k_1+k_2}{2} } \varepsilon ^2\\&\quad \lesssim 2^{5\delta m}\varepsilon ^2. \end{aligned}$$

Finally, if $2^{p}\sim 1$, with Lemma 5.13 and with the constraint $k_2\le k_1$ we have just two possibilities: either $2^{k_2}\ll 2^{k}\sim 2^{k_1}$ and $p_2\ll p\sim p_1\sim 0$ which was handled above, or $2^{k}\ll 2^{k_2}\sim 2^{k_1}$ which implies $p_1\le p_2\ll p\sim 0$ and in particular $2^{k}\lesssim 2^{k_2+p_2}$. From this the claim follows using Lemma 5.15 with $\left\lvert S\right\rvert \lesssim 2^{k_2+\frac{p_1}{2}}$ and (8.4):

$$\begin{aligned}&2^{4k^+}2^{(1+\beta )l}2^{\beta p}2^{\frac{p}{2}}\left\Vert P_{k,p}R_l\mathcal {B}_{\mathfrak {m}} (f_1,f_2)\right\Vert _{L^2} \\&\quad \lesssim 2^m2^{(1+\beta )l}2^{k}2^{k_2+\frac{p_1}{2}}2^{-l_1}2^{-\frac{p_1}{2}}\left\Vert f_1\right\Vert _X2^{-l_2} 2^{-\frac{p_2}{2}}\left\Vert f_2\right\Vert _X\\&\quad \lesssim 2^m2^{(\beta +3\delta ^2)(k-k_2+m+p_2)}2^{2k_2+p_2}2^{-(1-\delta )m}2^{-\frac{3}{2}p_2}\varepsilon ^2\\&\quad \lesssim 2^{(\beta +\delta +3\delta ^2)m}2^{(\beta +2\delta ^2)k}2^{(2-\beta -2\delta ^2)k_2}2^{-\frac{p_2}{2}}\varepsilon ^2\\&\quad \lesssim 2^{2\beta m}\varepsilon ^2. \end{aligned}$$

**B.2(b.2): Gap in** *p* **with** $p_{\min }\ll p_{\max }\ll 0$**.** Then $\left\lvert \Phi \right\rvert >\frac{1}{10}$ and we split the analysis in the resonant and non-resonant parts as presented in Sect. 5.7. By choosing $\lambda =\frac{1}{100}$, we have $\mathfrak {m}^{res}=0$ and so we can do a normal form as in Lemma 5.17 with $\mathfrak {m}^{nr}=\mathfrak {m}$. For the boundary term on the right-hand side of (5.12) there holds using (8.4) and $\left\lvert S\right\rvert \lesssim 2^{\frac{k_2+k_1+p_1}{2}}$

$$\begin{aligned} 2^{4k^+}2^{(1+\beta )l}2^{\beta p}2^{\frac{p}{2}}\left\Vert P_{k,p}R_l\mathcal {Q}_{\mathfrak {m}} (f_1,f_2)\right\Vert _{L^2}&\lesssim 2^{(1+\beta )l}2^{k}2^{\frac{k_1+k_2+p_{1}}{2}}2^{-l_1}2^{-\frac{p_1}{2} }\left\Vert f_1\right\Vert _X\left\Vert f_2\right\Vert _B\\&\lesssim 2^{(\beta +2\delta ^2)(k-k_2+m+p_2)}2^{k+\frac{k_1+k_2}{2}}\varepsilon ^2\\&\lesssim 2^{2\beta m}\varepsilon ^2, \end{aligned}$$

since $\delta _0\ll \delta ^2\ll \beta$. Next we estimate the remaining terms using Lemma 6.1, $\left\lvert S\right\rvert \lesssim 2^{k_2+\frac{p_2}{2}}$, condition (8.4) and the setting that (8.5) doesn’t hold. We note that here we balance the $X-$ and $B-$norms on $f_2$ to overcome the loss in $k_2$ and obtain a bounded $X-$norm:

$$\begin{aligned}&2^{4k^+}2^{(1+\beta )l}2^{\beta p}2^{\frac{p}{2}}\left\| P_{k,p}R_l\mathcal {B}_{\mathfrak {m}} (\partial _tf_1,f_2)\right\| _{L^2}\\  &\quad \lesssim 2^m2^{(1+\beta )l}2^{k+p_{\max }}2^{k_{2}+\frac{p_{2}}{2}}\left\| \partial _t f_1\right\| _{L^2}\left\| f_2\right\| _{L^2}\\  &\quad \lesssim 2^{(\frac{1}{4}+2\delta )m}2^{(1+\beta +3\delta ^2)(k-k_2+m+p_2)} 2^{k+p_{\max }}2^{k_2+\frac{p_2}{2}}\varepsilon ^2 2^{-(1-4\beta )l_2}2^{-(1-4\beta )\frac{p_2}{2}} \\  &\qquad \qquad \left\| f_2\right\| _{X}^{1-4\beta }2^{2k_2}2^{2\beta p_2}\left\| f_2\right\| _B^{4\beta }\\  &\quad \lesssim 2^{(\frac{1}{4}+5\beta +3\delta )m}2^{9\beta p_2}2^{4\beta p_{\max }} 2^{(2+\beta +3\delta ^2)k}2^{(\beta -3\delta ^2)k_2}\varepsilon ^3\\  &\quad \lesssim 2^{(\frac{1}{4}+6\beta )m}\varepsilon ^3. \end{aligned}$$

For the last term,

$$\begin{aligned} 2^{4k^+}2^{(1+\beta )l}2^{\beta p}2^{\frac{p}{2}}\left\| P_{k,p}R_l\mathcal {B}_{\mathfrak {m}} (f_1,\partial _tf_2)\right\| _{L^2}&\lesssim 2^m2^{(1+\beta )l}2^{k+\frac{k_2+k_1+p_1}{2}}\left\| f_1\right\| _{L^2}\left\| \partial _t f_2\right\| _{L^2}\\  &\lesssim 2^{(\frac{1}{4}+2\delta )m}2^{(1+\beta )l}2^{k+\frac{k_2+k_1+p_1}{2}}2^{-l_1}2^{-\frac{p_1}{2}}\left\| f_1\right\| _X\varepsilon ^2\\  &\lesssim 2^{(\frac{1}{4}+2\beta )m}\varepsilon ^3. \end{aligned}$$

This concludes all the cases and the proof of **Part 2**, and thus the proof of the proposition. $\square$

### *X*-Norm Bounds for $l<(1+\delta )m$

#### Proposition 8.2

In the setting of Proposition 2.7, and in particular under the bootstrap assumptions (2.26), the following holds true: For $\mathfrak {m}\in \left\{ \mathfrak {m}_0,\mathfrak {m}_\pm ^{\mu \nu } \mid \mu {,}\nu \in {\{{+}{,}{-}\}} \right\}$, $t\in [2^m,2^{m{+}1}[\cap [0,T]$ and $\delta =2M^{-\frac{1}{2}}$, there holds that

$$\begin{aligned} \sup _{\begin{array}{c} k,l,p\\ l+p\ge 0, l<(1+\delta )m \end{array}}2^{4k^+}2^{(1+\beta )l}2^{\beta p}2^{\frac{p}{2}}&\left\| P_{k,p}R_l\mathcal {B}_\mathfrak {m}(F_1,F_2)\right\| _{L^2}\lesssim 2^{(\frac{1}{2}-\frac{3}{4}\delta ) m }\varepsilon ^2+2^{(1-\beta )m}\varepsilon ^3, \end{aligned}$$

where $$F_i\in \{S^{b_i}\mathcal {Z}_{\pm },S^{b_i}\Theta \}$$, $$0\le b_1+b_2\le N$$, $$i=1,2$$.

This is the most challenging result of our article. Similarly to the proofs of Lemma 6.1 and Propositions 7.1, 8.1, we can use the energy estimates to treat very large or small frequencies. Otherwise, alongside previously used tools such as integration by parts along *S*, set-size estimates and normal forms, we need a more refined analysis in certain settings. The most delicate part of the proof concerns **Case B.2** when there holds that $p_1\le p_2\ll p$. This leads to large losses for integration by parts along *S*, while normal forms are not generally beneficial since $\left\lvert \Phi \right\rvert$ may be very small. To handle this, we use refined versions of the aforementioned tools adapted to the precise geometry of frequency interactions at hand, in particular also through set-size estimates as in Lemma 5.17(3). Moreover, in the “no gap” case (**Case D** below), the linear decay estimates alone do not suffice to obtain the claim, and instead we need to introduce additional localizations $q,q_1,q_2$ in the horizontal direction $\xi _1\left\lvert \xi \right\rvert ^{-1}$ (see Sect. 2.3). This allows for integration by parts in the vector field *S* and the use of normal forms that include the aforementioned parameters to conclude the proof.

#### Proof

**Case A: Simple cases.** As in the **Cases A** in the proofs of Propositions 7.1, 8.1, and with additional localizations $f_i=P_{k_i,p_i}R_{l_i}F_i$, we can treat most of the frequencies using the energy bounds obtained from the bootstrap assumption (2.26). Thus it suffices to prove

$$\begin{aligned}&\sup _{k,l<(1+\delta )m, l+p\ge 0} 2^{4k^+}2^{(1+\beta +2\delta )m}2^{\beta p}2^{\frac{p}{2}}\left\Vert P_{k,p}R_l\mathcal {B}_\mathfrak {m}(f_1,f_2)\right\Vert _{L^2}\\&\qquad \qquad \qquad \qquad \qquad \qquad \qquad \qquad \quad \lesssim 2^{(\frac{1}{2}-\frac{4}{5}\delta ) m} \varepsilon ^2+2^{(1-\frac{\beta }{2})m}\varepsilon ^3, \end{aligned}$$

for the following parameters:

$$\begin{aligned}&-4m<k,k_i<\delta _0m,  &   -p_i \le l_i\le 4m,  &   -4m\le p_i\le 0,  &   i=1,2. \end{aligned}$$

Note that in this setting there holds $2^{(1+\beta )l}\lesssim 2^{(1+\beta +2\delta )m}$. We proceed with several cases.

**Case B: Gap in** *p* **with** $p_{\max }\sim 0$ **and** $p_{\min }\ll p_{\max }\sim 0$**.** Here there holds $\left\lvert \sigma \right\rvert \sim 2^{k_{\max }+k_{\min }}$. Integration by parts along *S* via Lemma 5.10(1) yields the claim if

8.6 $$\begin{aligned} \begin{aligned} S_\eta : \hspace{1cm} 2^{2k_1}2^{-p_1}2^{-k_{\max }-k_{\min }}(1+2^{k_2-k_1}2^{l_1})&\le 2^{(1-\delta )m}, \\ S_{\xi -\eta }: \hspace{1cm} 2^{2k_2}2^{-p_2}2^{-k_{\max }-k_{\min }}(1+2^{k_1-k_2}2^{l_2})&\le 2^{(1-\delta )m}, \end{aligned} \end{aligned}$$

where $\delta =2M^{-\frac{1}{2}}$. Assume now that (8.6) doesn’t hold and that w.l.o.g. $p_1\le p_2$ and treat several cases based on Lemma 5.13.

**Case B.1:** $p_{\min }\sim p\ll p_{\max }\sim 0$**.** By Lemma 5.5 the multiplier bound reads $\Vert \mathfrak {m}\chi \Vert _{L^\infty }\lesssim 2^k$. By Lemma 5.13 and under the constraint $p_1\le p_2$, we have two further cases to consider.

**Case B.1(a):** $2^{k_1}\sim 2^{k_2}$. Then $p\ll p_1\sim p_2\sim 0$ and condition (8.6) doesn’t hold if $\max \{-l_1,-l_2\}< -(1-\delta )m-k+k_1$. We use the set size estimate Lemma 5.15 with $\left\lvert S\right\rvert \lesssim 2^{k}$ and the bootstrap assumption (2.26):

$$\begin{aligned}&2^{4k^+}2^{(1+\beta +2\delta )m}2^{\beta p}2^{\frac{p}{2}}\left\| P_{k,p}R_l\mathcal {B}_\mathfrak {m}(f_1,f_2)\right\| _{L^2} \\  &\quad \lesssim 2^{4k^+}2^{(2+\beta +2\delta )m}2^{2k}\left\| P_{k_1,p_1}f_1\right\| _{L^2}\left\| P_{k_2,p_2}f_2\right\| _{L^2}\\  &\quad \lesssim 2^{-4k_1^+}2^{(2+\beta +2\delta )m}2^{2k}2^{-(l_1+l_2)}\left\| f_1\right\| _X\left\| f_2\right\| _X\\  &\quad \lesssim 2^{(\beta +4\delta )m}2^{2k}2^{-3k_1^+}2^{2(-k+k_1)}\varepsilon ^2\\  &\quad \lesssim 2^{2\beta m}\varepsilon ^2. \end{aligned}$$

**Case B.1(b):** $2^{k_2}\ll 2^{k_1}\sim 2^k$. Then $p \le p_1\ll p_2\sim 0$, ${k_{\max }+k_{\min }}\sim {k_1+k_2}$ and $2^{k_2}\lesssim 2^{p_1+k}$. In this case we have $-l_1<-(1-\delta )m-p_1+k-k_2$ and $-l_2<-(1-\delta )m$ (cf. (8.6)). We obtain the claim from Lemma 5.15 with $\left\lvert S\right\rvert \lesssim 2^{k_2+\frac{p_2}{2}}\lesssim 2^{k_2}$:

$$\begin{aligned}&2^{4k^+}2^{(1+\beta +2\delta )m}2^{\beta p}2^{\frac{p}{2}}\left\Vert P_{k,p}R_l\mathcal {B}_\mathfrak {m}(f_1,f_2)\right\Vert _{L^2} \\&\quad \lesssim 2^{(2+\beta +2\delta )m}2^{(\frac{1}{2}+\beta ) p_1}2^{k+k_2}\left\Vert P_{k_1,p_1}f_1\right\Vert _{L^2}\left\Vert P_{k_2,p_2}f_2\right\Vert _{L^2}\\&\quad \lesssim 2^{(2+\beta +2\delta )m}2^{(\frac{1}{2}+\beta ) p_1}2^{k+k_2}2^{-\frac{5}{8}l_1}2^{-\frac{p_1}{8} }2^{-(1+\beta )l_2}\varepsilon ^2\\&\quad \lesssim 2^{(\frac{3}{8}+3\delta )m}2^{\frac{13}{8}k+\frac{3}{8}k_2}2^{(-\frac{1}{4}+\beta ) p_1} \varepsilon ^2\\&\quad \lesssim 2^{(\frac{3}{8}+4\delta )m}\varepsilon ^2. \end{aligned}$$

**Case B.2:** $p_{\min }\sim p_1\ll p_{\max }\sim 0$**.** By Lemma 5.13 we have the following three cases to consider.

**Case B.2(a):** $2^{k}\sim 2^{k_2}$**.** Then $p_1\ll p\sim p_2\sim 0$ and $k_{\max }+k_{\min }\sim {k_1+k_2}$. Condition (8.6) doesn’t hold if

$$\begin{aligned}&l_1-p_1>(1-\delta )m  &   \text { and }  &   \max \{ k_2-k_1,l_2\}>(1-\delta )m. \end{aligned}$$

**1.** If $\max \{ k_2-k_1,l_2\}=k_2-k_1>(1-\delta )m$, we obtain an admissible bound from Lemma 5.15 with $\left\lvert S\right\rvert \lesssim 2^{k_1+\frac{p_1}{2}}$:

$$\begin{aligned}&2^{4k^+}2^{(1+\beta +2\delta )m}2^{\beta p}2^{\frac{p}{2}}\left\Vert P_{k,p}R_l\mathcal {B}_\mathfrak {m}(f_1,f_2)\right\Vert _{L^2}\\&\quad \lesssim 2^{(2+\beta +2\delta )m}2^{k_1+\frac{p_1}{2}}2^{k}2^{-\frac{l_1}{2}} 2^{\frac{k_1^-}{4}}\left\Vert f_1\right\Vert _X^{\frac{1}{2}}\left\Vert f_1\right\Vert _{B}^{\frac{1}{2}}\left\Vert f_2\right\Vert _B\\&\quad \lesssim 2^{(\frac{3}{2}+\beta +3\delta )m}2^{\frac{5}{4}k_1}2^{k}\varepsilon ^2\\&\quad \lesssim 2^{(\frac{1}{4}+2\beta )m}\varepsilon ^2. \end{aligned}$$

**2.** If on the other hand $\max \{ k_2-k_1,l_2\}=l_2>(1-\delta )m$ we compute with $\left\lvert S\right\rvert \lesssim 2^{k_1+\frac{p_1}{2}}$

$$\begin{aligned}&2^{4k^+}2^{(1+\beta +2\delta )m}2^{\beta p}2^{\frac{p}{2}}\left\Vert P_{k,p}R_l\mathcal {B}_\mathfrak {m}(f_1,f_2)\right\Vert _{L^2} \\&\quad \lesssim 2^{(2+\beta +2\delta )m}2^{k+k_1+\frac{p_1}{2}}2^{-(1+\beta )(l_1+l_2)} 2^{-(\frac{1}{2}+\beta ) p_1}\left\Vert f_1\right\Vert _X\left\Vert f_2\right\Vert _X\\&\quad \lesssim 2^{-4k_1^+}2^{(-\beta +4\delta )m}2^{k_1+k}2^{-2(\frac{1}{2}+\beta )p_1}\varepsilon ^2\\&\quad \lesssim 2^{-4k_1^+}2^{(-\beta +5\delta )m}2^{(\frac{1}{2}+\beta )(k_1-k-2p_1)}\varepsilon ^2. \end{aligned}$$

The claim follows if $k_1-k-2p_1\le (1-2\beta )m$. Otherwise, if

8.7 $$\begin{aligned} k\le -(1-2\beta )m-2p_1+k_1, \end{aligned}$$

we do a splitting as presented in Sect. 5.7 with $\lambda =2^{-4\beta m}$. Thus, we have the following decomposition

$$\begin{aligned} \left\Vert P_{k,p}R_l\mathcal {B}_{\mathfrak {m}}(f_1,f_2)\right\Vert _{L^2}\lesssim \left\Vert P_{k,p}R_l\mathcal {B}_{\mathfrak {m}^{res}}(f_1,f_2)\right\Vert _{L^2}+ \left\Vert P_{k,p}R_l\mathcal {B}_{\mathfrak {m}^{nr}}(f_1,f_2)\right\Vert _{L^2}. \end{aligned}$$

Observe that with (8.7) and the definition of the phase (2.5) with $\mu ,\nu \in \{+,-\}$, we have

$$\begin{aligned} \left\lvert \partial _{\eta _1}\Phi _{\pm }^{\mu \nu }(\xi ,\eta )\right\rvert&=\left\lvert \mu \frac{(\xi _2-\eta _2)^2}{\left\lvert \xi -\eta \right\rvert ^3}+\nu \frac{\eta _2^2}{\left\lvert \eta \right\rvert ^3}\right\rvert \gtrsim \left\lvert 2^{2p_2}2^{-k_2}-2^{2p_1}2^{-k_1}\right\rvert \gtrsim 2^{-k}=:K. \end{aligned}$$

The resonant term can be treated via Lemma 5.17(3) with $\lambda =2^{-4\beta m}$ and $K=2^{-k}$:

$$\begin{aligned}&2^{4k^+}2^{(1+\beta +2\delta )m}2^{\beta p}2^{\frac{p}{2}}\left\Vert P_{k,p}R_l\mathcal {B}_{\mathfrak {m}^{res}}(f_1,f_2)\right\Vert _{L^2} \\&\quad \lesssim 2^{(2+\beta +2\delta )m}(\lambda K^{-1})^{\frac{1}{2}}2^{k+\frac{p_1}{2}}2^{\frac{p_1}{2}}2^{-(1+\beta )l_2}\left\Vert f_1\right\Vert _B\left\Vert f_2\right\Vert _X\\&\quad \lesssim 2^{(\frac{1}{2}-\frac{\beta }{2})m}\varepsilon ^2. \end{aligned}$$

On the non-resonant part, we can do a normal form as (5.12) and bound the $L^2$-norm of each term using Lemma 5.17. For the boundary term in (5.12) we have

$$\begin{aligned}&2^{4k^+}2^{(1+\beta +2\delta )m}2^{\beta p}2^{\frac{p}{2}}\left\Vert P_{k,p}R_l\mathcal {Q}_{\mathfrak {m}^{nr}\Phi ^{-1}}(f_1,f_2)\right\Vert _{L^2} \\&\quad \lesssim 2^{(1+\beta +2\delta )m}\lambda ^{-1}2^{2k+\frac{p_1}{2}}2^{\frac{p_1}{2}}\left\Vert f_1\right\Vert _B2^{-(1+\beta )l_2}\left\Vert f_2\right\Vert _X\\&\quad \lesssim 2^{(-\frac{1}{2}+2\beta )m }\varepsilon ^2. \end{aligned}$$

For the second term in the splitting (5.12), we use Lemma 6.1 and $\left\lvert S\right\rvert \lesssim 2^{k_1+\frac{p_1}{2}}$ to get that

$$\begin{aligned}&2^{4k^+}2^{(1+\beta +2\delta )m}2^{\beta p}2^{\frac{p}{2}}\left\Vert P_{k,p}R_l\mathcal {B}_{\mathfrak {m}^{nr}\Phi ^{-1}}(\partial _tf_1,f_2)\right\Vert _{L^2}\\&\quad \lesssim 2^{4k^+}2^{(2+\beta +2\delta )m}2^{k}\lambda ^{-1}\left\lvert S\right\rvert \left\Vert \partial _tf_1\right\Vert _{L^2}\left\Vert f_2\right\Vert _{L^2}\\&\quad \lesssim 2^{(2+\beta +2\delta )m}2^{k+k_1}\lambda ^{-1}2^{\frac{p_1}{2}}2^{(-\frac{3}{4}+2\delta )m}\varepsilon ^22^{-(1+\beta )(1-\delta )m}\left\Vert f_2\right\Vert _X\\&\quad \lesssim 2^{5\beta m}\varepsilon ^3. \end{aligned}$$

Similarly, for the last term in (5.12) with Lemma 6.1 and $\left\lvert S\right\rvert \lesssim 2^{k_1+\frac{p_1}{2}}$ we have

$$\begin{aligned}&2^{4k^+}2^{(1+\beta +2\delta )m}2^{\beta p}2^{\frac{p}{2}}\left\Vert P_{k,p}R_l\mathcal {B}_{\mathfrak {m}^{nr}\Phi ^{-1}}(f_1,\partial _tf_2)\right\Vert _{L^2} \\&\quad \lesssim 2^{4k^+}2^{(2+\beta +2\delta )m}2^{k}\lambda ^{-1}\left\lvert S\right\rvert \left\Vert f_1\right\Vert _{L^2}\left\Vert \partial _tf_2\right\Vert _{L^2}\\&\quad \lesssim 2^{(2+\beta +2\delta )m}2^{2k}\lambda ^{-1}2^{{p_1}}\left\Vert f_1\right\Vert _B2^{(-\frac{3}{4}+2\delta )m}\varepsilon ^2\\&\quad \lesssim 2^{(\frac{3}{4}+6\beta )m}\varepsilon ^3. \end{aligned}$$

**Case B.2(b):** $2^{k}\ll 2^{k_1}\sim 2^{k_2}$ **and** $p_1\le p_2\ll p=p_{\max }\sim 0.$ By Lemma 5.13 there holds $2^{k}\lesssim 2^{p_2+k_2}\sim 2^{p_2+k_1}$. We obtain the claim via integration by parts if $l_1\le (1-\delta )m+p_1+k-k_1$ or $l_2\le (1-\delta )m+p_2+k-k_1$, see (8.6). Hence we may assume

8.8 $$\begin{aligned}&-l_1<-(1-\delta )m -p_1-k+k_1  &   \text { and }  &   -l_2<-(1-\delta )m -p_2-k+k_1. \end{aligned}$$

In this setting, we treat two different parts based on the signs in the phase and on the relative size of $p_1$ to $p_2$. Recall the definition of the phases (2.5), i.e.,

$$\begin{aligned} \Phi _\pm ^{\mu \nu }=\pm \Lambda (\xi )-\mu \Lambda (\xi -\eta )-\nu \Lambda (\eta ),&\hspace{1.5cm} \mu ,\nu \in \{+,-\}. \end{aligned}$$

**Case B.2(b.1):** Assume $\mu =\nu$ and $p_1\sim p_2\ll p\sim 0$.

**1.** If $$\Lambda (\xi -\eta )\Lambda (\eta )>0$$, since $$2^{p_1}\sim 2^{p_2}\ll 1$$

$$\begin{aligned} \left\lvert \Lambda (\xi -\eta )+\Lambda (\eta )\right\rvert \ge \frac{3}{2}. \end{aligned}$$

This implies in particular that the phase is large:

$$\begin{aligned} \left\lvert \Phi _\pm ^{\mu \mu }\right\rvert =\left\lvert \pm \Lambda (\xi )-\mu \Lambda (\xi -\eta )-\mu \Lambda (\eta )\right\rvert \ge \left\lvert \left\lvert \Lambda (\xi -\eta )+\Lambda (\eta )\right\rvert -\left\lvert \Lambda (\xi )\right\rvert \right\rvert \ge \frac{1}{2}. \end{aligned}$$

With this observation and $\lambda =10^{-2}$, we note that a splitting as in Sect. 5.7 contains only the non-resonant part. That is $\mathcal {B}_{\mathfrak {m}}(f_1,f_2)=\mathcal {B}_{\mathfrak {m}^{nr}}(f_1,f_2)$ and we can apply Lemma 5.17 to bound each term in (5.12) in $L^2$. We proceed with the boundary term and with Lemmas 3.4, 5.17(2) and (8.8) obtain that

$$\begin{aligned} 2^{4k^+}2^{(1+\beta +2\delta )m}\left\Vert P_{k,p}R_l\mathcal {Q}_{\mathfrak {m}^{nr}\Phi ^{-1}}(f_1,f_2)\right\Vert _{L^2}&\lesssim 2^{4k^+}2^{(1+\beta +2\delta )m}2^{k}\left\Vert e^{it\Lambda }f_1\right\Vert _{L^\infty }\left\Vert f_2\right\Vert _{L^2}\\&\lesssim 2^{(\frac{1}{2}+\beta +\kappa +2\delta )m}2^k2^{\frac{l_2}{2}}\varepsilon ^2\\&\lesssim 2^{(\beta +\kappa +3\delta )m}2^{\frac{k_1}{2}}\varepsilon ^2. \end{aligned}$$

For the terms in (5.12) containing the time derivative we use Lemmas 5.17(1), 6.1 and with $\left\lvert S\right\rvert \lesssim 2^{\frac{k+k_1+p_2}{2}}$ obtain that

$$\begin{aligned}&2^{4k^+}2^{(1+\beta +2\delta )m} \left\Vert P_{k,p}R_l\mathcal {B}_{\mathfrak {m}^{nr}\Phi ^{-1}}(\partial _tf_1,f_2)\right\Vert _{L^2}\\&\quad \lesssim 2^{4k^+}2^{(2+\beta +2\delta )m}2^{\frac{3k+k_1+p_2}{2}}\left\Vert \partial _tf_1\right\Vert _{L^2}\left\Vert f_2\right\Vert _{L^2}\\&\quad \lesssim 2^{(\frac{5}{4}+\beta +2\delta )m}2^{\frac{3k+k_1+p_2}{2}}2^{-\frac{l_2}{2}}\varepsilon ^3\\&\quad \lesssim 2^{(\frac{3}{4}+2\beta )m}\varepsilon ^3. \end{aligned}$$

The third term is bounded similarly by symmetry using (8.8) on $l_1$ instead.

**Fig. 2:**
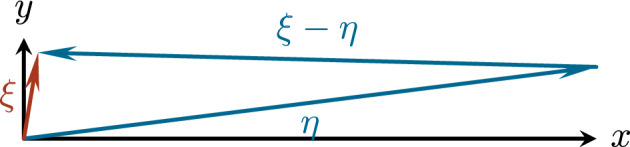
A sample setting of Case 2

**2.** Assume $\Lambda (\xi -\eta )\Lambda (\eta )<0$. We assume w.l.o.g. that $\Lambda (\xi -\eta )<0$ and $\Lambda (\eta )>0$ (see Figure 2 for illustration).

First we observe that

$$\begin{aligned} \left\lvert \partial _{\eta _1}\Phi _{\pm }^{\mu \mu }\right\rvert \chi =\left\lvert \Pi (\xi -\eta )-\Pi (\eta )\right\rvert \chi ,\qquad \Pi (\zeta ):={\zeta _2^2}/{\left\lvert \zeta \right\rvert ^3}, \qquad \zeta \in \mathbb {R}^2. \end{aligned}$$

Moreover, that

$$\begin{aligned} \nabla _{\zeta }\Pi (\zeta )=\frac{\zeta _2}{\left\lvert \zeta \right\rvert ^3}\Big (-\frac{3\zeta _1\zeta _2}{\left\lvert \zeta \right\rvert ^2}, \frac{-\zeta _2^2+2\zeta _1^2}{\left\lvert \zeta \right\rvert ^2} \Big )^T, \end{aligned}$$

and by the mean value theorem together with the condition $2^{k}\lesssim 2^{p_1+k_1}$ we obtain

$$\begin{aligned} \left\lvert \partial _{\eta _1}\Phi _{\pm }^{\mu \mu }\right\rvert \chi = \left\lvert \Pi (\xi -\eta )-\Pi (\eta )\right\rvert \chi (\xi ,\eta )\ge \inf _{(\zeta ,\upsilon ) \in {{\,\textrm{supp}\,}}\chi }\left\lvert (\nabla \Pi )(\zeta )\cdot \upsilon \right\rvert \gtrsim 2^{p_1-2k_1+k}. \end{aligned}$$

Then we can integrate by parts in $D_{\eta }=\left\lvert \eta \right\rvert \partial _{\eta _1}$ using Lemma 5.11 with $L=2^{p_1-2k_1+k}$ and obtain the claim if

$$\begin{aligned} 2^{-p_1-k+k_1}(2^{l_1+p_1}+2^{l_2+p_2})<2^{(1-\delta )m}. \end{aligned}$$

That is, we may assume that

$$\begin{aligned} \max \{2^{l_1-k+k_1},2^{l_2-k+k_1}\}>2^{(1-\delta )m}. \end{aligned}$$

Without loss of generality, we assume $2^{l_1-k+k_1}>2^{(1-\delta )m}$ and that (8.8) holds for $l_2$. The other case is treated by symmetry. To handle this case, we do a splitting $\mathfrak {m}=\mathfrak {m}^{nr}+\mathfrak {m}^{res}$ as in Sect. 5.7 with $\lambda =2^{4\beta k}2^{(1+8\beta )p_1}$. On the non-resonant part we bound each term in (5.12) and using $2^{k}\lesssim 2^{p_1+k_1}$ and the *X*-norm on $f_1$, *B*-norm on $f_2$, we obtain

$$\begin{aligned}&2^{4k^+}2^{(1+\beta +2\delta )m}\left\Vert P_{k,p}R_l\mathcal {Q}_{\mathfrak {m}^{nr}\Phi ^{-1}}(f_1,f_2)\right\Vert _{L^2} \\&\quad \lesssim 2^{4k^+}2^{(1+\beta +2\delta )m}2^{\frac{3k+k_1+p_1}{2}}\lambda ^{-1}\left\Vert f_1\right\Vert _{L^2}\left\Vert f_2\right\Vert _{L^2}\\&\quad \lesssim 2^{(1+\beta +2\delta )m}2^{\frac{3k+k_1+p_1}{2}}\lambda ^{-1} 2^{-(\frac{1}{2}+2\beta )l_1}2^{-2\beta p_1}2^{-4k_1^+}\varepsilon ^2\\&\quad \lesssim 2^{(\frac{1}{2}-\beta +3\delta )m}2^{(\frac{3}{2}-\frac{1}{2}-6\beta )k}2^{(1+2\beta )k_1}2^{(-\frac{1}{2}-10\beta )p_1}2^{-4k_1^+}\varepsilon ^2\\&\quad \lesssim 2^{(\frac{1}{2}-\frac{\beta }{2})m}\varepsilon ^2, \end{aligned}$$

where we have used $2^{k}\lesssim 2^{p_1+k_1}$. Next, we bound using Lemma 6.1, $\left\lvert S\right\rvert \lesssim 2^{\frac{k+k_1+p_1}{2}}$ and the *X*-norm :

$$\begin{aligned}&2^{4k^+}2^{(1+\beta +2\delta )m}\left\Vert P_{k,p}R_l\mathcal {B}_{\mathfrak {m}^{nr}\Phi ^{-1}}(\partial _tf_1,f_2)\right\Vert _{L^2} \\&\quad \lesssim 2^{4k^+}2^{(2+\beta +2\delta )m}2^{\frac{3k+k_1+p_1}{2}}\lambda ^{-1}\left\Vert \partial _tf_1\right\Vert _{L^2}\left\Vert f_2\right\Vert _{L^2}\\&\quad \lesssim 2^{(\frac{5}{4}+\beta +3\delta )m}2^{(\frac{3}{2}-4\beta )k}2^{(-\frac{1}{2}-8\beta )p_1}2^{-(\frac{1}{4}+2\beta )l_2}2^{(\frac{1}{4}-2\beta )p_1}\varepsilon ^3\\&\quad \lesssim 2^{(1-\beta +3\delta )m}2^{(\frac{5}{4}-6\beta )k}2^{(-\frac{1}{2}-12\beta )p_1}\varepsilon ^3\\&\quad \lesssim 2^{(1-\frac{\beta }{2})m}\varepsilon ^3. \end{aligned}$$

Finally, for the third term, with Lemma 6.1 and $\left\lvert S\right\rvert \lesssim 2^{\frac{k+k_1+p_1}{2}}$ there holds:

$$\begin{aligned}&2^{4k^+}2^{(1+\beta +2\delta )m}\left\Vert P_{k,p}R_l\mathcal {B}_{\mathfrak {m}^{nr}\Phi ^{-1}}(f_1,\partial _tf_2)\right\Vert _{L^2} \\&\quad \lesssim 2^{4k^+}2^{(2+\beta +2\delta )m}2^{\frac{3k+k_1+p_1}{2}}\lambda ^{-1}\left\Vert f_1\right\Vert _{L^2}\left\Vert \partial _tf_2\right\Vert _{L^2}\\&\quad \lesssim 2^{(\frac{5}{4}+\beta +3\delta )m}2^{(\frac{3}{2}-4\beta )k}2^{\frac{k_1}{2}}2^{(-\frac{1}{2}-8\beta )p_1}2^{-\frac{l_1}{2}}\varepsilon ^3\\&\quad \lesssim 2^{(\frac{3}{4}+\beta +3\delta )m}2^{(1-4\beta )k}2^{k_1}2^{(-\frac{1}{2}-8\beta )p_1}\varepsilon ^3\\&\quad \lesssim 2^{(\frac{3}{4}+2\beta )m}\varepsilon ^3. \end{aligned}$$

On the resonant set, we observe that

$$\begin{aligned} \left\lvert \partial _{\xi _1}\Phi _{\pm }^{\mu \nu }\right\rvert =\left\lvert \pm \frac{\xi _2^2}{\left\lvert \xi \right\rvert ^3}-\mu \frac{(\xi _2-\eta _2)^2}{\left\lvert \xi -\eta \right\rvert ^3}\right\rvert \gtrsim \left\lvert 2^{2p-k}-2^{2p_1-k_1}\right\rvert \gtrsim 2^{-k}=:K. \end{aligned}$$

Therefore, we can employ Lemma 5.17(3) with $\lambda$ and $K=2^{-k}$ and (8.11) on $l_2$ to obtain

$$\begin{aligned}&2^{4k^+}2^{(1+\beta +2\delta )m}\left\Vert P_{k,p}R_l\mathcal {B}_{\mathfrak {m}^{res}}(f_1,f_2)\right\Vert _{L^2}\\&\quad \lesssim 2^{4k^+}2^{(2+\beta +2\delta )m}2^{k} (\lambda K^{-1})^{\frac{1}{2}}2^{\frac{k_1+p_1}{2}}\left\Vert f_1\right\Vert _{L^2}\left\Vert f_2\right\Vert _{L^2}\\&\quad \lesssim 2^{(2+\beta +2\delta )m} 2^{(\frac{3}{2}+2\beta )k}2^{(1+4\beta )p_1}2^{\frac{k_1}{2}}2^{-(1+\beta )l_1}2^{-(\frac{1}{2}+\beta )p_1} 2^{-4k_1^+}2^{-(\frac{1}{2}+\beta )l_2}2^{-2\beta p_1}\varepsilon ^2\\&\quad \lesssim 2^{(\frac{1}{2}-\beta +4\delta )m}\varepsilon ^2. \end{aligned}$$

This concludes the proof of **Case B.2(b.1)**.

**Case B.2(b.2):** Assume $\mu =-\nu$ or $p_1\ll p_2$. In this setting, we can split the analysis in the resonant and non-resonant parts as explained in Sect. 5.7 with $\lambda =2^{(\frac{3}{2}-6\beta )p_2}$. On the non-resonant part we have the three terms in (5.12). With Lemma 5.17(1), Lemma 5.15 with $\left\lvert S\right\rvert \lesssim 2^{\frac{k+k_1+p_1}{2}}$ and (8.8) for the boundary term we obtain using the *X*-norms on $f_i$:

$$\begin{aligned}&2^{4k^+}2^{(1+\beta +2\delta )m}\left\Vert P_{k,p}R_l\mathcal {Q}_{\mathfrak {m}^{nr}\Phi ^{-1}}(f_1,f_2)\right\Vert _{L^2} \\&\quad \lesssim 2^{4k^+}2^{(1+\beta +2\delta )m}2^{\frac{3k+k_1+p_1}{2}}\lambda ^{-1}\left\Vert f_1\right\Vert _{L^2}\left\Vert f_2\right\Vert _{L^2}\\&\quad \lesssim 2^{(1+\beta +2\delta )m}2^{\frac{3k+k_1+p_1}{2}}\lambda ^{-1} 2^{-\frac{l_1}{2}}2^{-4k_1^+}2^{-2\beta l_2}2^{(\frac{1}{2}-2\beta )p_2}\varepsilon ^2\\&\quad \lesssim 2^{(\frac{1}{2}-\beta +3\delta )m}2^{(1-2\beta )k}2^{(1+2\beta )k_1}\lambda ^{-1}2^{(\frac{1}{2}-4\beta )p_2}2^{-4k_1^+}\varepsilon ^2\\&\quad \lesssim 2^{(\frac{1}{2}-\beta +3\delta )m} 2^{(\frac{3}{2}-6\beta )p_2}2^{2k_1}\lambda ^{-1}2^{-4k^+_1}\varepsilon ^2. \end{aligned}$$

Note that we have used $2^{-\frac{l_2}{2}}\le 2^{-2\beta l_2}2^{(\frac{1}{2}-2\beta )p_2}$ since $l_2+p_2\ge 0$. For the other two terms in (5.12) we obtain with Lemma 6.1, $\left\lvert S\right\rvert \lesssim 2^{\frac{k+k_1+p_2}{2}}$ and (8.8):

$$\begin{aligned}&2^{4k^+}2^{(1+\beta +2\delta )m}\left\Vert P_{k,p}R_l\mathcal {B}_{\mathfrak {m}^{nr}\Phi ^{-1}}(\partial _tf_1,f_2)\right\Vert _{L^2}\\&\quad \lesssim 2^{4k^+}2^{(2+\beta +2\delta )m}2^{\frac{3k+k_1+p_2}{2}}\lambda ^{-1}\left\Vert \partial _tf_1\right\Vert _{L^2}\left\Vert f_2\right\Vert _{L^2}\\&\quad \lesssim 2^{(\frac{5}{4}+\beta +3\delta )m}2^{\frac{3k+k_1+p_2}{2}}\lambda ^{-1}2^{-(\frac{1}{4}+2\beta )l_2}2^{(\frac{1}{4}-2\beta )p_2}2^{-4k^+_1}\varepsilon ^3\\&\quad \lesssim 2^{(1-\beta +3\delta )m}2^{\frac{3k+k_1+p_2}{2}}\lambda ^{-1}2^{-4\beta p_2}2^{-(\frac{1}{4}+2\beta )k_1}2^{(\frac{1}{4}+2\beta )k_1}\varepsilon ^3\\&\quad \lesssim 2^{(1-\beta +3\delta )m}2^{(\frac{5}{4}-2\beta )k}2^{(\frac{3}{4}+2\beta )k_1}2^{(-1+2\beta )p_2}\varepsilon ^3\\&\quad \lesssim 2^{(1-\frac{\beta }{2})m}\varepsilon ^3, \end{aligned}$$

since $\delta _0\ll \delta \ll \beta$. The third term in (5.12) is bounded analogously by using Lemma 6.1 on $f_2$ and the $X-$norm on $f_1$.

On the resonant part, we first observe

8.9 $$\begin{aligned} \left\lvert \partial _{\xi _1}\Phi _{\pm }^{\mu \nu }\right\rvert =\left\lvert \pm \frac{\xi _2^2}{\left\lvert \xi \right\rvert ^3}-\mu \frac{(\xi _2-\eta _2)^2}{\left\lvert \xi -\eta \right\rvert ^3}\right\rvert \gtrsim \left\lvert 2^{2p-k}-2^{2p_1-k_1}\right\rvert \gtrsim 2^{-k}=:K. \end{aligned}$$

Next recall

$$\begin{aligned} \left\lvert \partial _{\eta _1}\Phi _\pm ^{\mu \nu }\right\rvert =\left\lvert \mu \frac{(\xi _2-\eta _2)^2}{\left\lvert \xi -\eta \right\rvert ^3}-\nu \frac{\eta _2^2}{\left\lvert \eta \right\rvert ^3}\right\rvert , \end{aligned}$$

and observe that if $\mu =-\nu$ or $p_1\ll p_2$ there holds

$$\begin{aligned} \left\lvert \partial _{\eta _1}\Phi ^{\mu \nu }_\pm \right\rvert \gtrsim 2^{2p_2-k_2}. \end{aligned}$$

In this case we can integrate by parts using Lemma 5.11 with $L=2^{2p_2}$ and obtain the claim if $\max \{{l_1+p_1-2p_2},{l_2-p_2}\}<{(1-\delta )m}$.

Now we assume that

8.10 $$\begin{aligned} \max \{{l_1+p_1-2p_2},{l_2-p_2}\}>{(1-\delta )m}, \end{aligned}$$

and note that this is an improvement compared to (8.8) since we do not have losses in *k*. We consider two cases based on which term in (8.10) is the largest.

**1.** Assume $l_1>(1-\delta )m-p_1+2p_2$ and $l_2$ satisfies (8.8). Therefore, with (8.9) we can bound the resonant part using Lemma 5.17(3) with $\lambda =2^{(\frac{3}{2}-6\beta )p_2}$ and $K=2^{-k}$ :

$$\begin{aligned}&2^{4k^+}2^{(1+\beta +2\delta )m}\left\Vert P_{k,p}R_l\mathcal {B}_{\mathfrak {m}^{res}}(f_1,f_2)\right\Vert _{L^2}\\&\quad \lesssim 2^{4k^+}2^{(2+\beta +2\delta )m}2^{k}(\lambda K^{-1})^{\frac{1}{2}}2^{\frac{k_1+p_1}{2}}\left\Vert f_1\right\Vert _{L^2}\left\Vert f_2\right\Vert _{L^2}\\&\quad \lesssim 2^{(2+\beta +2\delta )m}2^{\frac{3}{2}k}2^{(\frac{3}{4}-3\beta )p_2}2^{\frac{k_1+p_2}{2}}2^{-(1+\beta )l_1}2^{(-\frac{1}{2}-\beta )p_1} 2^{-(\frac{1}{2}+\frac{\beta }{2})l_2}2^{-\frac{\beta }{2}p_2}2^{-4k_1^+}\varepsilon ^2\\&\quad \lesssim 2^{(\frac{1}{2}-\frac{\beta }{2}+4\delta )m}2^{\frac{3k+k_1}{2}}2^{(\frac{5}{4}-3\beta )p_2}2^{\frac{p_1}{2}}2^{-2(1+\beta )p_2}2^{-(\frac{1}{2}+\beta )p_2}2^{-(\frac{1}{2}+\frac{\beta }{2})k}2^{(\frac{1}{2}+\frac{\beta }{2})k_1}2^{-4k_1^+}\varepsilon ^2\\&\quad \lesssim 2^{(\frac{1}{2}-\frac{\beta }{2}+4\delta )m}2^{(1-\frac{\beta }{2})k}2^{(1+\frac{\beta }{2})k_1}2^{(-\frac{3}{4}-6\beta )p_2}2^{4k_1^+}\varepsilon ^2\\&\quad \lesssim 2^{(\frac{1}{2}-\frac{\beta }{4})m}\varepsilon ^2. \end{aligned}$$

**2.** Assume $l_2-p_2>(1-\delta )m$ and $l_1$ satisfies (8.8). In this case we do another splitting

$$\begin{aligned} \mathfrak {m}^{res}(\xi ,\eta )&=\psi (\lambda _1^{-1}\Phi )\mathfrak {m}^{res}(\xi ,\eta )+(1-\psi (\lambda _1^{-1}\Phi ))\mathfrak {m}^{res}(\xi ,\eta )\\&=: \mathfrak {m}^{res,res}(\xi ,\eta )+\mathfrak {m}^{res,nr}(\xi ,\eta ) \end{aligned}$$

with $\lambda _1:=\lambda 2^{-20\delta m}<\lambda$ and obtain a decomposition of the bilinear term

$$\begin{aligned} \mathcal {B}_{\mathfrak {m}^{res}}(f_1,f_2)=\mathcal {B}_{\mathfrak {m}^{res,res}}(f_1,f_2)+\mathcal {B}_{\mathfrak {m}^{res,nr}}(f_1,f_2). \end{aligned}$$

We can estimate the first term as follows using Lemma 5.17(3) with $K=2^{-k}$, see (8.9), and $\lambda _1$:

$$\begin{aligned}&2^{4k^+}2^{(1+\beta +2\delta )m}\left\Vert P_{k,p}R_l\mathcal {B}_{\mathfrak {m}^{res,res}}(f_1,f_2)\right\Vert _{L^2}\\&\quad \lesssim 2^{4k^+}2^{(2+\beta +2\delta )m}2^{k}(\lambda _1 K^{-1})^{\frac{1}{2}}2^{\frac{k_1+p_1}{2}}\left\Vert f_1\right\Vert _{L^2}\left\Vert f_2\right\Vert _{L^2}\\&\quad \lesssim 2^{(2+\beta +2\delta )m}2^{\frac{3k+k_1+p_1}{2}}2^{(\frac{3}{4}-3\beta )p_2}2^{-10\delta m}2^{-\frac{l_1}{2}} 2^{-4k_1^+}2^{-(1+\beta )l_2}2^{-(\frac{1}{2}+\beta )p_2}\varepsilon ^2\\&\quad \lesssim 2^{(\frac{1}{2}-6\delta )m}2^{k+k_1}2^{(\frac{3}{4}-3\beta )p_2}2^{(-\frac{3}{2}-2\beta )p_2}2^{-4k_1^+}\varepsilon ^2\\&\quad \lesssim 2^{(\frac{1}{2}-6\delta )m}2^{(\frac{1}{4}-5\beta )k}2^{(\frac{7}{4}+5\beta )k_1}2^{-4k_1^+}\varepsilon ^2\\&\quad \lesssim 2^{(\frac{1}{2}-6\delta )m}\varepsilon ^2. \end{aligned}$$

We bound the terms arising in the non-resonant part as in (5.12). Using Lemma 5.17(1) with $\lambda _1$ and A.3 we obtain using the *X*-norm on $f_1$ and $f_2$:

$$\begin{aligned}&2^{4k^+}2^{(1+\beta +2\delta )m}\left\Vert P_{k,p}R_l\mathcal {Q}_{\Phi ^{-1}\mathfrak {m}^{res,nr}}(f_1,f_2)\right\Vert _{L^2}\\&\quad \lesssim 2^{4k^+}2^{(1+\beta +2\delta )m}2^{\frac{3k+k_1+p_1}{2}}\lambda _1^{-1}\left\Vert f_1\right\Vert _{L^2}\left\Vert f_2\right\Vert _{L^2}\\&\quad \lesssim 2^{(1+\beta +22\delta )m}2^{\frac{3k+k_1+p_1}{2}}2^{-(\frac{3}{2}-6\beta )p_2}2^{-\frac{l_1+l_2}{2}}2^{-4k_1^+}\varepsilon ^2\\&\quad \lesssim 2^{(\frac{1}{2}+\beta +23\delta )m}2^{\frac{3k+k_1+p_1}{2}}2^{-(2-6\beta )p_2} 2^{-4k_1^+}2^{-2\beta l_1}2^{(\frac{1}{2}-2\beta )p_1}\varepsilon ^2\\&\quad \lesssim 2^{(\frac{1}{2}-\beta +24\delta )m}2^{(\frac{3}{2}-2\beta )k}2^{(\frac{1}{2}+2\beta )k_1} 2^{(1-4\beta )p_1}2^{(-2+6\beta )p_2}2^{-4k_1^+}\varepsilon ^2\\&\quad \lesssim 2^{(\frac{1}{2}-\beta +24\delta )m}2^{(\frac{3}{2}-2\beta )k}2^{(\frac{1}{2}+2\beta )k_1} 2^{(-1+2\beta )p_2}2^{-4k_1^+}\varepsilon ^2\\&\quad \lesssim 2^{(\frac{1}{2}-\frac{\beta }{2})m}\varepsilon ^2. \end{aligned}$$

For the terms containing the time derivative we obtain with Lemma 6.1:

$$\begin{aligned}&2^{4k^+}2^{(1+\beta +2\delta )m}\left\Vert P_{k,p}R_l\mathcal {B}_{\Phi ^{-1}\mathfrak {m}^{res,nr}}(\partial _tf_1,f_2)\right\Vert _{L^2} \\&\quad \lesssim 2^{4k^+}2^{(2+\beta +2\delta )m}2^{\frac{3k+k_1+p_1}{2}}\lambda _1^{-1}\left\Vert \partial _tf_1\right\Vert _{L^2}\left\Vert f_2\right\Vert _{L^2}\\&\quad \lesssim 2^{(\frac{5}{4}+\beta +23\delta )m}2^{\frac{3k+k_1+p_1}{2}}2^{-(\frac{3}{2}-6\beta )p_2}2^{-\frac{l_2}{2}}\varepsilon ^3\\&\quad \lesssim 2^{(\frac{3}{4}+\beta +24\delta )m}2^{\frac{3k+k_1}{2}}2^{-(\frac{3}{2}-6\beta )p_2}\varepsilon ^3\\&\quad \lesssim 2^{(\frac{3}{4}+2\beta )m}\varepsilon ^3. \end{aligned}$$

Finally that

$$\begin{aligned}&2^{4k^+}2^{(1+\beta +2\delta )m}\left\Vert P_{k,p}R_l\mathcal {B}_{\Phi ^{-1}\mathfrak {m}^{res,nr}}(f_1,\partial _tf_2)\right\Vert _{L^2}\\&\quad \lesssim 2^{4k^+}2^{(2+\beta +2\delta )m}2^{\frac{3k+k_1+p_1}{2}}\lambda _1^{-1}\left\Vert f_1\right\Vert _{L^2}\left\Vert \partial _tf_2\right\Vert _{L^2}\\&\quad \lesssim 2^{(\frac{5}{4}+\beta +23\delta )m}2^{\frac{3k+k_1+p_1}{2}}2^{-(\frac{3}{2}-6\beta )p_2}2^{-\frac{l_1}{2}}\varepsilon ^3\\&\quad \lesssim 2^{(\frac{5}{4}+\beta +23\delta )m}2^{\frac{3k+k_1+p_1}{2}}2^{-(\frac{3}{2}-6\beta )p_2}2^{-(\frac{1}{4}+2\beta )l_1}2^{(\frac{1}{4}-2\beta )p_1}\varepsilon ^3\\&\quad \lesssim 2^{(1-\beta +24\delta )m}2^{(\frac{5}{4}-2\beta )k}2^{(\frac{3}{4}+2\beta )k_1}2^{-(\frac{3}{2}-6\beta )p_2}2^{(\frac{1}{2}-4\beta )p_1}\varepsilon ^3\\&\quad \lesssim 2^{(1-\beta +24\delta )m}2^{\frac{p_2}{4}}2^{2k_1}\varepsilon ^3, \end{aligned}$$

which gives an acceptable contribution as $\delta _0\ll \delta \ll \beta \ll 1$.

**Case B.2(c):** $2^{k_2}\ll 2^k\sim 2^{k_1}$ **and** $p_1\ll p\ll p_2\sim 0$**.** By Lemma 5.13 we have $2^{p_2+k_2}\sim 2^{p+k}$ and $k_{\max }+k_{\min }\sim k_2+k_1$. We obtain the claim if $l_1\le (1-\delta )m +p_1+k_2-k$, or $l_2\le (1-\delta )m$, see (8.6). Hence we may assume

8.11 $$\begin{aligned} -l_1<-(1-\delta )m-p_1+k_1-k_2  &   \text { and }  &   -l_2<-(1-\delta )m. \end{aligned}$$

Using the set size estimate Lemma 5.15 with $\left\lvert S\right\rvert \lesssim 2^{\frac{k+p_1+k_2}{2}}$ we bound

$$\begin{aligned} \left\Vert P_{k,p}R_l\mathcal {B}_{\mathfrak {m}}(f_1,f_2)\right\Vert _{L^2}&\lesssim 2^m 2^{\frac{3}{2}k+\frac{p_1}{2}}2^{\frac{k_2}{2}}2^{-4k^+}2^{-\frac{l_1}{2}}2^{-4k_2^+}2^{-(1+\beta )l_2}2^{-\beta p_2-\frac{p_2}{2}}\varepsilon ^2\\&\lesssim 2^m2^{\frac{3}{2}k+\frac{k_2}{2}+\frac{p_1}{2}}2^{-\frac{(1-\delta )m}{2}-\frac{p_1}{2}+\frac{k-k_2}{2}}2^{-4k^+-4k_2^+}2^{-(1+\beta )(1-\delta )m}\varepsilon ^2\\&\lesssim 2^{(-\frac{1}{2}-\beta +2\delta )m}2^{2k}2^{-4k^+}\varepsilon ^2. \end{aligned}$$

Thus for the *X*-norm and with $2^p\sim 2^{k_2-k}$ that

$$\begin{aligned}&2^{4k^+}2^{(1+\beta +2\delta )m}2^{\beta p} 2^{\frac{p}{2}}\left\Vert P_{k,p}R_l\mathcal {B}_{\mathfrak {m}}(f_1,f_2)\right\Vert _{L^2}&\lesssim 2^{(\frac{1}{2}+4\delta )m}2^{2k}2^{(\frac{1}{2}+\beta )(k_2-k)}\varepsilon ^2 \\&\quad \lesssim 2^{(\frac{1}{2}+6\delta )m}2^{(\frac{1}{2}+\beta ) k_2}\varepsilon ^2, \end{aligned}$$

and the claim follows if $k_2\le -20\delta m$. Assume now that $k_2>-20\delta m$. We decompose the multiplier into the resonant and non-resonant part as in Sect. 5.7 with $\lambda =2^{-100\delta m}$. For $\Phi _{\pm }^{\mu \nu }$ on the support of the resonant set

$$\begin{aligned} \left\lvert \partial _{\eta _1}\Phi _{\pm }^{\mu \nu }\right\rvert&= \left\lvert \mu 2^{2p_2-k_2}-\nu 2^{2p_1-k_1}\right\rvert \gtrsim \left\lvert 2^{2p_2-k_2}-2^{2p_2+2k_2-3k}\right\rvert \gtrsim 2^{-k_2}=:K>0. \end{aligned}$$

Using Lemma 5.17(3) with $K=2^{-k_2}$ and $\lambda$ we estimate

$$\begin{aligned}&2^{4k^+}2^{(1+\beta +2\delta )m}2^{\beta p} 2^{\frac{p}{2}} \left\Vert P_{k,p}R_l\mathcal {B}_{\mathfrak {m}^{res}}(f_1,f_2)\right\Vert _{L^2} \\&\quad \lesssim 2^{4k^+}2^{(2+\beta +2\delta )m}2^{\frac{3}{2}k}2^{\frac{p_1}{2}}(\lambda K^{-1})^{\frac{1}{2}}\left\Vert f_1\right\Vert _{L^2}\left\Vert f_2\right\Vert _{L^2}\\&\quad \lesssim 2^{(2+\beta +2\delta )m}2^{\frac{3}{2}k}2^{\frac{p_1}{2}}(\lambda K^{-1})^{\frac{1}{2}} 2^{-\frac{l_1}{2}}2^{-(1+\beta )l_2}\varepsilon ^2\\&\quad \lesssim 2^{(\frac{1}{2}-40\delta ) m}\varepsilon ^2. \end{aligned}$$

Now we turn to the non-resonant term and do a normal form as in (5.12). For the boundary term we obtain with Lemma 5.17(1) and (8.11):

$$\begin{aligned}&2^{4k^+}2^{(1+\beta +2\delta )m}2^{\beta p} 2^{\frac{p}{2}}\left\Vert P_{k,p}R_l\mathcal {Q}_{\Phi ^{-1}\mathfrak {m}^{nr}}(f_1,f_2)\right\Vert _{L^2}\\&\quad \lesssim 2^{4k^+}2^{(1+\beta +2\delta )m}2^{\frac{3}{2}k}\lambda ^{-1}2^{\frac{p_1+k_2}{2}}\left\Vert f_1\right\Vert _{L^2}\left\Vert f_2\right\Vert _{L^2}\\&\quad \lesssim 2^{(1+\beta +2\delta )m}2^{\frac{3}{2}k}\lambda ^{-1}2^{\frac{p_1+k_2}{2}}2^{-\frac{l_1}{2}}\left\Vert f_1\right\Vert _Xt2^{-(1+\beta )l_2}2^{-(\frac{1}{2}+\beta )p_2}\left\Vert f_2\right\Vert _X\\&\quad \lesssim 2^{(-\frac{1}{2}+110\delta )m}\varepsilon ^2. \end{aligned}$$

For the other terms in the non-resonant decomposition (5.12) we estimate using Lemma 6.1:

$$\begin{aligned}&2^{4k^+}2^{(1+\beta +2\delta )m}2^{\beta p} 2^{\frac{p}{2}}\left\Vert P_{k,p}R_l\mathcal {B}_{\mathfrak {m}^{nr}\Phi ^{-1}}(f_1,\partial _tf_2)\right\Vert _{L^2} \\&\quad \lesssim 2^{4k^+}2^{(2+\beta +2\delta )m} 2^{\frac{3}{2}k} \lambda ^{-1}2^{\frac{p_1+k_2}{2}}\left\Vert f_1\right\Vert _{L^2}\left\Vert \partial _tf_2\right\Vert _{L^2}\\&\quad \lesssim 2^{(2+\beta +2\delta )m} 2^{\frac{3}{2}k}\lambda ^{-1}2^{\frac{p_1+k_2}{2}}2^{-\frac{l_1}{2}}\left\Vert f_1\right\Vert _X2^{(-\frac{3}{4}+2\delta )m}\varepsilon ^2\\&\quad \lesssim 2^{(\frac{3}{4}+2\beta )m}\varepsilon ^3. \end{aligned}$$

Finally, with $\left\lvert S\right\rvert \lesssim 2^{\frac{k+k_2}{2}}$ and the *X*-norm on $f_2$ we obtain that

$$\begin{aligned}&2^{4k^+}2^{(1+\beta +2\delta )m} 2^{\beta p} 2^{\frac{p}{2}} \left\Vert P_{k,p}R_l\mathcal {B}_{\mathfrak {m}^{nr}\Phi ^{-1}}(\partial _tf_1,f_2)\right\Vert _{L^2} \\&\quad \lesssim 2^{4k^+}2^{(2+\beta +2\delta )m} 2^{\frac{3}{2}k} 2^{\frac{k_2}{2}}\lambda ^{-1}\left\Vert \partial _tf_1\right\Vert _{L^2}\left\Vert f_2\right\Vert _{L^2}\\&\quad \lesssim 2^{4k^+}2^{(2+\beta +2\delta )m} 2^{\frac{3}{2}k}\lambda ^{-1}2^{(-\frac{3}{4}+2\delta )m} 2^{-(1+\beta )l_2}\varepsilon ^3\\&\quad \lesssim 2^{(\frac{1}{4}+110\delta )m}\varepsilon ^3, \end{aligned}$$

which is more than enough for the claim of the proposition.

**Case C:** $p_{\max }\ll 0$**.** In this case we have that the phase $\Phi$ is large $\left\lvert \Phi \right\rvert >\frac{1}{10}$ and we can do a splitting as per Sect. 5.7 with $\lambda =\frac{1}{100}$ and thus $\mathfrak {m}^{res}=0$. So we have $\mathcal {B}_{\mathfrak {m}}(f_1,f_2)=\mathcal {B}_{\mathfrak {m}^{nr}}(f_1,f_2)$ and we can split the bilinear term as in (5.12). For the last two terms, using Lemmas 5.17(2) and 3.4, we have that

$$\begin{aligned}&2^{4k^+}2^{(1+\beta +2\delta )m}2^{\beta p}2^{\frac{p}{2}} \left\Vert P_{k,p}R_l\mathcal {B}_{\mathfrak {m}^{nr}\Phi ^{-1}}(\partial _tf_1,f_2)\right\Vert _{L^2} \\&\quad \lesssim 2^{(2+\beta +3\delta )m}2^{\beta p}2^{\frac{p}{2}}2^{k+p_{\max }}\left\Vert \partial _tf_1\right\Vert _{L^2}\left\Vert e^{it\Lambda }f_2\right\Vert _{L^\infty }\\&\quad \lesssim 2^{(\frac{3}{4}+2\beta )m}\varepsilon ^3, \end{aligned}$$

assuming $\kappa \ll \beta$ in Lemma 3.4. The other term is symmetric in this estimate and is bounded analogously. As for the boundary term, assume w.l.o.g. $p_1\le p_2$, then we have the multiplier bound from Lemma 5.5. We distinguish two cases.

**Case C.1:** If $f_2$ has fewer vector fields than $f_1$, then we can decompose $f_2$ according to Proposition 3.2$P_{k_2,p_2}e^{it\Lambda }f_2=I_{k_2,p_2}(f_2)+II_{k_2,p_2}(f_2)$ with bounds as in (3.2)–(3.3) and use Lemma 3.4 on $f_1$. With $\log (t)\lesssim 2^{\delta m}$ and using Lemmas 3.4 and 5.17(2) we obtain

$$\begin{aligned}&\left\Vert P_{k,p}R_l\mathcal {Q}_{\mathfrak {m}\Phi ^{-1}}(f_1,f_2)\right\Vert _{L^2}\\&\quad \lesssim 2^{k}\big [\left\Vert f_1\right\Vert _{L^2}\left\Vert I_{k_2,p_2}(f_2)\right\Vert _{L^\infty }+\Vert e^{it\Lambda }f_1\Vert _{L^\infty }\left\Vert II_{k_2,p_2}(f_2)\right\Vert _{L^2} \big ]\\&\quad \lesssim 2^{k}\big [ 2^{-4k_1^+}2^{\frac{p_1}{2}}\left\Vert f_1\right\Vert _B 2^{\frac{3}{4}k_2}2^{-\frac{15}{4}k_2^+}\min \{2^{-p_2}2^{-m},2^{p_2}\}2^{\delta m}\varepsilon \\&\qquad +2^{-2k_1^+}2^{(-\frac{1}{2}+\kappa )m}\varepsilon 2^{-4k_2^+}2^{-\frac{m}{2}}\varepsilon \big ]\\&\quad \lesssim 2^{k}[2^{-3k_2^+-4k_1^+}2^{(-\frac{3}{4}+\delta )m}+2^{-4k_2^+-2k_1^+}2^{(-1+\kappa ) m}]\varepsilon ^2\\&\quad \lesssim 2^{(-\frac{3}{4}+\delta )m} \varepsilon ^2. \end{aligned}$$

For the *X*-norm we then obtain that

$$\begin{aligned} 2^{4k^+}2^{(1+\beta +2\delta )m}2^{\beta p}2^{\frac{p}{2}}\left\Vert P_{k,p}R_l\mathcal {Q}_{\mathfrak {m}\Phi ^{-1}}(f_1,f_2)\right\Vert _{L^2}&\lesssim 2^{(\frac{1}{4}+\beta +6\delta )m}\varepsilon ^2, \end{aligned}$$

which gives the claim.

**Case C.2** If $f_2$ has more vector fields than $f_1$, then by Proposition 3.2 there holds $\Vert e^{it\Lambda }f_1\Vert _{L^\infty }\lesssim 2^{\frac{3}{4}k_1-3k_1^+}2^{-\frac{m}{2}}\varepsilon$. Moreover, notice that since $\left\lvert \Phi \right\rvert >\frac{1}{10}$, there holds:

$$\begin{aligned} \left\lvert \frac{\mathfrak {m}\chi S_{\xi -\eta }(\Phi ^{-1})}{S_{\xi -\eta } \Phi }\right\rvert =\left\lvert \frac{\mathfrak {m}\chi \Phi ^{-2}S_{\xi -\eta } \Phi }{S_{\xi -\eta } \Phi }\right\rvert \lesssim \left\lvert \mathfrak {m}\chi \right\rvert . \end{aligned}$$

Therefore, we can integrate by parts along $S_{\xi -\eta }$, see proof of Lemma 5.10. This gives the claim if

8.12 $$\begin{aligned} 2^{-p_2-p_{\max }}2^{2k_2-k_{\max }-k_{\min }}(1+2^{k_1-k_2}2^{l_2})<2^{(1-\delta )m}. \end{aligned}$$

Otherwise we distinguish different cases and do an $L^2-L^{\infty }$ estimate:

**Case C.2(a):** $k_{\min }+k_{\max }\sim k_1+k_2$**.** Assume $k_2-k_1<l_2$, then (8.12) doesn’t hold if $-l_2<-(1-\delta )m-p_2-p_{\max }$, then we have, for the boundary term,

$$\begin{aligned}&2^{4k^+}2^{(1+\beta +2\delta )m}2^{\beta p}2^{\frac{p}{2}} \left\Vert P_{k,p}R_l\mathcal {Q}_{\mathfrak {m}\Phi ^{-1}}(f_1,f_2)\right\Vert _{L^2}\\&\quad \lesssim 2^{4k^+}2^{(1+\beta +2\delta )m}2^{k+p_{\max }}2^{-3k_1^+}2^{-\frac{m}{2}}\varepsilon \left\Vert f_2\right\Vert _{L^2}\\&\quad \lesssim 2^{(\frac{1}{2}+\beta +3\delta )m} 2^{k+p_{\max }}\varepsilon 2^{-\frac{l_2}{4}}2^{\frac{p_2}{4}}\left\Vert f_2\right\Vert _X\\&\quad \lesssim 2^{(\frac{1}{4}+\beta +6\delta )m}\varepsilon ^2. \end{aligned}$$

Otherwise, if $$k_1-k_2<-(1-\delta )m-p_2-p_{\max }$$

$$\begin{aligned}&2^{4k^+}2^{(1+\beta +2\delta )m}2^{\beta p}2^{\frac{p}{2}} \left\Vert P_{k,p}R_l\mathcal {Q}_{\mathfrak {m}\Phi ^{-1}}(f_1,f_2)\right\Vert _{L^2} \\&\quad \lesssim 2^{4k^+}2^{(1+\beta +2\delta )m}2^{k+p_{\max }}2^{\frac{3k_1}{4}}2^{-3k_1^+}2^{-\frac{m}{2}}\varepsilon 2^{\frac{p_2}{2}}\left\Vert f_2\right\Vert _B\\&\quad \lesssim 2^{2\beta m}\varepsilon ^2. \end{aligned}$$

**Case C.2(b):** $k_{\min }\sim k$**.** Then (8.12) doesn’t holds if $-l_2<-(1-\delta )m-p_2-p_{\max }-k+k_1$. Otherwise:

$$\begin{aligned}&2^{4k^+}2^{(1+\beta +2\delta )m}2^{\beta p}2^{\frac{p}{2}} \left\Vert P_{k,p}R_l\mathcal {Q}_{\mathfrak {m}\Phi ^{-1}}(f_1,f_2)\right\Vert _{L^2} \\&\quad \lesssim 2^{4k^+}2^{(1+\beta +2\delta )m}2^{k+p_{\max }}2^{-\frac{m}{2}}\varepsilon 2^{-6k_2^+}2^{-\frac{l_2}{4}}2^{\frac{p_2}{4}}\left\Vert f_2\right\Vert _X\\&\quad \lesssim 2^{(\frac{1}{2}+\beta +3\delta )m}2^{k+p_{\max }}2^{-\frac{(1-\delta )m}{4}-\frac{p_2+p_{\max }}{4}+\frac{k_2-k}{4}}2^{\frac{p_2}{4}}\varepsilon ^2\\&\quad \lesssim 2^{(\frac{1}{4}+2\beta )m}\varepsilon ^2. \end{aligned}$$

This finished **Case C**.

**Case D: No gaps with** $p\sim p_1\sim p_2\sim 0$**.** As the linear decay is not enough to obtain the claim, in this instance we introduce the $q,q_1,q_2$ localizations:

$$\begin{aligned} P_{k,p}R_lB_{\mathfrak {m}}(f_1,f_2)=\sum _{q,q_1,q_2\in \mathbb {Z}^-}P_{k,p,q}R_lB_{\mathfrak {m}}(P_{k_1,p_1,q_1}f_1,P_{k_2,p_2,q_2}f_2), \end{aligned}$$

where by abuse of notation we let $f_i=P_{k_i,p_i,q_i}R_{l_i}f_i$ for $i=1,2$. Moreover, recall the notation $\widetilde{\chi }$ from (2.17) and note that from Lemma 5.5 there holds $\left\Vert \mathfrak {m}\widetilde{\chi }\right\Vert _{L^\infty }\lesssim 2^{k+p_{\max }+q_{\max }}$.

Using the set size estimate 5.15, it suffices to bound $\left\Vert P_{k,p,q}B_{\mathfrak {m}}\left( P_{k_1,p_1,q_1}f_1,\right. \right\Vert \left. P_{k_2,p_2,q_2}f_2\right) _X$ for finitely many $q,\;q_1,\;q_2\in \mathbb {Z}^-$. Indeed,

$$\begin{aligned} \left\Vert P_{k,p}R_lB_{\mathfrak {m}}(f_1,f_2)\right\Vert _{L^2}&\lesssim 2^m2^k \sum _{q,q_1,q_2\in \mathbb {Z}^-}\left\Vert P_{k,p,q}R_lB_{\mathfrak {m}}(f_1,f_2)\right\Vert _{L^2}\\&\lesssim 2^m2^{2k_{\max }} \sum _{q,q_1,q_2\in \mathbb {Z}^-} 2^{\frac{q_{\min }}{2}}2^{-N_0k_1^+}2^{-N_0k_2^+}\left\Vert f_1\right\Vert _{H^{N_0}}\left\Vert f_2\right\Vert _{H^{N_0}}. \end{aligned}$$

Therefore with the energy estimates obtained from the bootstrap assumption (2.26) we obtain the claim if $q_{\min }<-12m$. There in what follows, we assume that $q, q_1, q_2>-12m$ and prove that

$$\begin{aligned} \left\Vert P_{k,p,q}R_lB_{\mathfrak {m}}(f_1,f_2)\right\Vert _{L^2}\lesssim 2^{(-\frac{1}{2}-\frac{3}{2}\beta +3\delta )m}\varepsilon ^2. \end{aligned}$$

Therefore, since $\log (t)\sim m\lesssim 2^{\delta m}$, we obtain the claim of the proposition:

$$\begin{aligned} \left\Vert P_{k,p}R_lB_{\mathfrak {m}}(f_1,f_2)\right\Vert _{L^2}&\lesssim \sum _{\begin{array}{c} q,q_1,q_2\in \mathbb {Z}^-\\ q_{\min }<-12m \end{array}} \left\Vert P_{k,p,q}R_lB_{\mathfrak {m}}(f_1,f_2)\right\Vert _{L^2} \\&\quad +\sum _{\begin{array}{c} q,q_1,q_2\in \mathbb {Z}^-\\ q_{\min }\ge -12m \end{array}} \left\Vert P_{k,p,q}R_lB_{\mathfrak {m}}(f_1,f_2)\right\Vert _{L^2}\\&\lesssim 2^{-2m}\varepsilon ^2+ \sum _{q,q_1,q_2\ge -12m}2^{(-\frac{1}{2}-\frac{3}{2}\beta +3\delta )m}\varepsilon ^2\\&\lesssim 2^{(-\frac{1}{2}-\frac{5}{4}\beta )m}\varepsilon ^2. \end{aligned}$$

To begin with, we split the analysis $\mathfrak {m}=\mathfrak {m}^{res}+\mathfrak {m}^{nr}$ as described in Sect. 5.7 with $\lambda = 2^{q_{\max }-10}$. On the non-resonant part we do a normal transform as in (5.12) and treat each term separately. Observe that by Lemma A.3,

$$\begin{aligned} \left\lvert \mathfrak {m}^{nr}\Phi ^{-1}\right\rvert \lesssim \left\Vert \mathfrak {m}^{nr}\right\Vert _W\Vert \Phi ^{-1}\Vert _W\lesssim 2^{k+q_{\max }}2^{-q_{\max }}\lesssim 2^k. \end{aligned}$$

Moreover, assume w.l.o.g. that $f_1$ has fewer vector fields than $f_2$. Then from Proposition 3.2 since $p\sim p_i\sim 0$, we have $f_1=P_{q_1}I_{k_1,p_1}(f_1)+P_{q_1}II_{k_1,p_1}(f_1)$ with the following estimates:

8.13 $$\begin{aligned} \left\Vert I_{k_1,p_1}(f_1)\right\Vert _{L^\infty }\lesssim 2^{\frac{3k_1}{4}}2^{-\frac{15}{4}k_1^+}2^{(-1+\delta )m}\varepsilon ,  &   \left\Vert II_{k_1,p_1}(f_1)\right\Vert _{L^2}\lesssim 2^{-4k_1^+}2^{-(\frac{1}{2}+\frac{\beta }{2})m}\varepsilon . \end{aligned}$$

Hence for the boundary term in the normal form (5.12) by Lemma A.1 and Lemma 3.4 with $\kappa \ll \beta /2$ on $f_2$, we obtain

8.14 $$\begin{aligned} \begin{aligned}&\left\Vert P_{k,p,q}R_l\mathcal {Q}_{\mathfrak {m}^{nr}\Phi ^{-1}}(f_1,f_2)\right\Vert _{L^2} \\&\quad \lesssim 2^k[\left\Vert I_{k_1,p_1}(f_1)\right\Vert _{L^\infty }\left\Vert f_2\right\Vert _{L^2}+\left\Vert II_{k_1,p_1}(f_1)\right\Vert _{L^2}\Vert e^{it\Lambda }f_2\Vert _{L^\infty }]\\&\quad \lesssim 2^{k}[ 2^{-3k_1^+}2^{(-1+\delta )m}2^{-4k_2^+}\varepsilon ^2+ 2^{-4k_1^+}2^{-(\frac{1}{2}+\frac{\beta }{2})m} 2^{-2k_2^+}2^{(-\frac{1}{2}+\kappa )m}\varepsilon ^2]\\&\quad \lesssim 2^{(-1+\delta )m}\varepsilon ^2. \end{aligned} \end{aligned}$$

Thus for the *X*-norm we obtain

$$\begin{aligned} 2^{4k^+}2^{(1+\beta +2\delta )m}\left\Vert P_{k,p,q}R_l\mathcal {Q}_{\mathfrak {m}^{nr}\Phi ^{-1}}(f_1,f_2)\right\Vert _{L^2}&\lesssim 2^{2\beta m}\varepsilon ^2, \end{aligned}$$

which is an acceptable bound. Next using Lemma 6.1 we compute that

8.15 $$\begin{aligned} \begin{aligned}&2^{4k^+}2^{(1+\beta +2\delta )m}\left\Vert P_{k,p,q}R_l\mathcal {B}_{\mathfrak {m}^{nr}\Phi ^{-1}}(\partial _tf_1,f_2)\right\Vert _{L^2}\\&\quad \lesssim 2^{4k^+}2^{(2+\beta +2\delta )m}2^k\left\Vert \partial _tf_1\right\Vert _{L^2}\Vert e^{it\Lambda }f_2\Vert _{L^\infty }\\&\quad \lesssim 2^{(\frac{3}{4}+2\beta )m}\varepsilon ^3. \end{aligned} \end{aligned}$$

Similarly we obtain the claim for the other term in (5.12), where we have even a better bound using the decomposition (8.13) on $f_1$. This concludes the non-resonant part.

As for the resonant case, we observe that if $\left\lvert \Phi \right\rvert <2^{q_{\max }-10}$ then by Proposition 5.8 there holds $\left\lvert \sigma \right\rvert >2^{k_{\min }+k_{\max }+q_{\max }}$. Here we consider several cases based on the sizes of $q,\; q_i$.

**Case D.1:** $q_1\ge q_{\max }-50$ **or** $q_2\ge q_{\max }-50$**.** We integrate by parts along *S* using Lemma 5.10(2) when feasible. Observe that

$$\begin{aligned} \left\lvert \frac{\mathfrak {m}\widetilde{\chi }S_\eta \psi (\lambda ^{-1}\Phi )}{s S_\eta \Phi }\right\rvert \lesssim \left\lvert \mathfrak {m}\widetilde{\chi }\psi '(\lambda ^{-1}\Phi )\lambda ^{-1}s^{-1}\right\rvert . \end{aligned}$$

Therefore, we can integrate by parts using Lemma 5.10(2) and obtain the claim if

8.16 $$\begin{aligned}&S_\eta :\hspace{0.3em}\nonumber \\&\quad \max \{2^{k_2-k_1-q_1},2^{2k_1}2^{-k_{\min }-k_{\max }-q_{\max }}(1+2^{k_2-k_1}(2^{q_2-q_1}+2^{l_1})),2^{-q_{\max }}\}\nonumber \\&\quad <2^{(1-\delta )m}, \end{aligned}$$

8.17 $$\begin{aligned}&S_{\xi -\eta }:\nonumber \\&\quad \max \{2^{k_1-k_2-q_2},2^{2k_2}2^{-k_{\min }-k_{\max }-q_{\max }}(1+2^{k_1-k_2}(2^{q_1-q_2}+2^{l_2})),2^{-q_{\max }}\}\nonumber \\&\quad <2^{(1-\delta )m}. \end{aligned}$$

Otherwise we proceed with several cases **D.1(a)–(d)** below. In the cases **D.1(a)–(c)** we can assume w.l.o.g. that $q_1\ge q_{\max }-50$ and use *only* the integration by parts in $S_\eta$ (8.16). The claim for $q_2\ge q_{\max }-50$ follows analogously by integrating by parts in $S_{\xi -\eta }$ using (8.17) and the symmetric estimates are obtained with the roles of $q_1,q_2$ and $k_1,k_2$ interchanged. The **Case D.1(d)** is treated separately depending whether $q_1\ge q_{\max }-50$ or $q_2\ge q_{\max }-50$.

Let $q_1\ge q_{\max }-50$ and assume that (8.16) does not hold.

**Case D.1(a):** If $2^{q_1}<2^{-(1-\delta )m}$, then

$$\begin{aligned} \left\Vert P_{k,p,q}R_l\mathcal {B}_{\mathfrak {m}^{res}}(f_1,f_2)\right\Vert _{L^2}&\lesssim 2^m2^{k+q_{1}}[\left\Vert I_{k_1,p_1}(f_1)\right\Vert _{L^\infty }\left\Vert f_2\right\Vert _{L^2} \\&\quad +\left\lvert S\right\rvert \left\Vert II_{k_1,p_1}(f_1)\right\Vert _{L^2}\left\Vert f_2\right\Vert _{L^2}]\\&\lesssim 2^{\delta m}[2^{(-1+\delta )m}+2^{k_{1}+\frac{q_{1}}{2}} 2^{(-\frac{1}{2}-\frac{\beta }{2})m}]\varepsilon ^2\\&\lesssim 2^{(-1+2\delta )m}\varepsilon ^2. \end{aligned}$$

This yields an admissible bound for the $X-$norm.

**Case D.1(b):** If $2^{k_1}<2^{-(1-\delta )m}2^{k_2-q_1}$ and $k_1\ll k_2\sim k$. Then we do another splitting $\mathfrak {m}^{res}=\mathfrak {m}^{res,nr}+\mathfrak {m}^{res,res}$ as per Sect. 5.7 with $\lambda _1=\lambda 2^{-4\beta m}<\lambda$. On the non-resonant part using Lemma A.3 we obtain that

$$\begin{aligned} \left\lvert \mathfrak {m}^{res,nr}\Phi ^{-1}\right\rvert \lesssim \left\Vert \mathfrak {m}^{res,nr}\right\Vert _W\left\Vert \Phi ^{-1}\right\Vert _W\lesssim 2^{k+4\beta m}. \end{aligned}$$

Then we can treat the terms arising from the normal form in (5.12) via Lemma 5.17(1) as in (8.14)–(8.15). The boundary term is estimated with Lemmas A.1, 3.4 and (8.13) as follows:

8.18 $$\begin{aligned} \begin{aligned}&\left\| P_{k,p,q}R_l\mathcal {Q}_{\mathfrak {m}^{res,nr}\Phi ^{-1}}(f_1,f_2)\right\| _{L^2} \\  &\quad \lesssim 2^{k+4\beta m}\Big [\left\| I_{k_1,p_1}(f_1)\right\| _{L^\infty }\left\| f_2\right\| _{L^2}+\left\| II_{k_1,p_1}(f_1)\right\| _{L^2}\Vert e^{it\Lambda }f_2\Vert _{L^\infty }\Big ]\\  &\quad \lesssim 2^{k+4\beta m}[ 2^{-3k_1^+}2^{(-1+\delta )m}2^{-4k_2^+}\varepsilon ^2+ 2^{-4k_1^+}2^{-(\frac{1}{2}+\frac{\beta }{2})m} 2^{-2k_2^+}2^{(-\frac{1}{2}+\kappa )m}\varepsilon ^2]\\  &\quad \lesssim 2^{(-1+4\beta +\delta )m}\varepsilon ^2. \end{aligned} \end{aligned}$$

This yields an admissible bound on the $X-$norm. For the terms in (5.12) involving the time derivative we apply Lemma 6.1 to obtain an admissible bound as follows:

$$\begin{aligned} \begin{aligned}&2^{4k^+}2^{(1+\beta +2\delta )m}\left\Vert P_{k,p,q}R_l\mathcal {B}_{\mathfrak {m}^{res,nr}\Phi ^{-1}}(\partial _tf_1,f_2)\right\Vert _{L^2}\\&\quad \lesssim 2^{4k^+}2^{(2+5\beta +2\delta )m}2^k\left\Vert \partial _tf_1\right\Vert _{L^2}\Vert e^{it\Lambda }f_2\Vert _{L^\infty }\\&\quad \lesssim 2^{(\frac{3}{4}+6\beta )m}\varepsilon ^3. \end{aligned} \end{aligned}$$

Similarly,

8.19 $$\begin{aligned} \begin{aligned}&2^{4k^+}2^{(1+\beta +2\delta )m}\left\Vert P_{k,p,q}R_l\mathcal {B}_{\mathfrak {m}^{res,nr}\Phi ^{-1}}(f_1,\partial _tf_2)\right\Vert _{L^2}\\&\quad \lesssim 2^{4k^+}2^{(2+5\beta +2\delta )m}2^k\Vert e^{it\Lambda }f_1\Vert _{L^\infty }\left\Vert \partial _tf_2\right\Vert _{L^2}\\&\quad \lesssim 2^{(\frac{3}{4}+6\beta )m}\varepsilon ^3. \end{aligned} \end{aligned}$$

On the resonant part we observe that since $k_1\ll k_2$,

$$\begin{aligned} |\partial _{\eta _1}\Phi _\pm ^{\mu \nu }|=|\mu 2^{2p_2-k_2}-\nu 2^{2p_1-k_1}|\gtrsim 2^{-k_1}=: K, \end{aligned}$$

and we can use Lemma 5.17(3),(5) with $K=2^{-k_1}$ and $\lambda _1$ to obtain that

$$\begin{aligned}&\left\| P_{k,p,q}R_l\mathcal {B}_{\mathfrak {m}^{res,res}}(f_1,f_2)\right\| _{L^2}\\  &\quad \lesssim 2^m2^{k+q_{1}}\Big [\left\| I_{k_1,p_1}(f_1)\right\| _{L^\infty }\left\| f_2\right\| _{L^2}+(\lambda _1 K^{-1})^{\frac{1}{2}}2^{\frac{k_1+q_1}{2}}\left\| II_{k_1,p_1}(f_1)\right\| _{L^2}\left\| f_2\right\| _{L^2}\Big ]\\  &\quad \lesssim 2^{(-\frac{1}{2}-2\beta )m}\varepsilon ^2. \end{aligned}$$

Observe that if $q_2\ge q_{\max }-50$, then we can use Lemma 5.17(3),(5) instead of the $L^\infty$ decay in the first term of the sum on the right-hand side above to obtain the claim.

**Case D.1(c):** Assume $2^{-k_1}<2^{-(1-\delta )m}2^{-k_{\min }-q_1}$ and $2^{k_2-k_1+l_1}\ll 1$. In particular, there holds $k_{\min }\sim k_2\ll k_1$. We additionally split the analysis in a resonant and non-resonant part with $\lambda _1=2^{-4\beta m}\lambda$. Indeed, the non-resonant part where $\left\lvert \Phi _\pm ^{\mu \nu }\right\rvert \gtrsim \lambda _1$ is handled as in (8.18)–(8.19) using the linear decay (8.13) and Lemma 5.17(1), while on the resonant part we have $\left\lvert \partial _{\eta _1}\Phi _\pm ^{\mu \nu }\right\rvert \gtrsim 2^{-k_2}$ and we can use Lemma 5.17(3),(5) with $L=2^{-k_2}$ and $\lambda _1$ as we have done in **Case D.1(b)**.

**Case D.1(d):** First let $q_2\ge q_{\max }-50$. The remaining case to treat if (8.17) doesn’t hold is when $2^{-l_2}<2^{-(1-\delta )m}2^{k_1+k_2-k_{\min }-k_{\max }-q_2}$. Then we obtain the claim using (8.13) and Lemma 5.15:

$$\begin{aligned}&\left\Vert P_{k,p,q}R_l\mathcal {B}_{\mathfrak {m}^{res}}(f_1,f_2)\right\Vert _{L^2} \\&\quad \lesssim 2^m2^{k+q_{2}}[\left\Vert I_{k_1,p_1}(f_1)\right\Vert _{L^\infty }\left\Vert f_2\right\Vert _{L^2}+\left\lvert S\right\rvert \left\Vert II_{k_1,p_1}(f_1)\right\Vert _{L^2}\left\Vert f_2\right\Vert _{L^2}]\\&\quad \lesssim 2^{m}2^{k+q_2}[2^{-(1-\delta )m}2^{-l_2}+2^{\frac{k_{\min }}{2}}2^{\frac{k_2+q_2}{2}}2^{(-\frac{1}{2}-\beta +\delta )m}2^{-(1+\beta )l_2}]\varepsilon \left\Vert f_2\right\Vert _X\\&\quad \lesssim 2^{(-\frac{1}{2}-2\beta +4\delta )m}\varepsilon ^2. \end{aligned}$$

Let now $q_1\ge q_{\max }-50$ and assume $2^{-l_1}<2^{-(1-\delta )m}2^{k_1+k_2-k_{\min }-k_{\max }-q_1}$. Here we obtain the claim by additionally integrating by parts in $S_{\xi -\eta }$ using (8.17). Observe that here in each of the terms in (8.17) we either have a “loss" in the parameter $q_1$ *or* $q_2$, cf.Lemma 5.10(2).

Assume now that (8.17) doesn’t hold. We consider several cases based on the relative size of the parameters $k,k_1,k_2$.

**Case D.1(d.1):** $2^{k_2}\ll 2^{k_1}$ **or** $2^{k_1}\ll 2^{k_2}$**.** In particular, there holds $2^{-l_1}<2^{-(1-\delta )m-q_1}$. Moreover:

$$\begin{aligned} \left\lvert \partial _{\eta _1}\Phi _\pm ^{\mu \nu }\right\rvert =\left\lvert \nu \partial _{\eta _1}\Lambda (\xi -\eta )\pm \nu \partial _{\eta _1}\Lambda (\eta )\right\rvert \gtrsim 2^{-\min \{k_1,k_2\}}=:K. \end{aligned}$$

We split the analysis further into the resonant and non-resonant part with $\lambda _1=\lambda 2^{-4\beta m}$. The non-resonant part is treated as in (8.18)–(8.19) using the linear decay (8.13) and Lemmas 5.17(1), 6.1 which are independent of the relative size of $k_1$ to $k_2$. On the resonant set, where $\left\lvert \Phi \right\rvert \lesssim \lambda _1$, we can use Lemma 5.17(3),(5) with $K=2^{-\min \{k_1,k_2\}}$, $\lambda _1$ and *q*-localizations to obtain the bound

8.20 $$\begin{aligned} \begin{aligned} \left\Vert P_{k,p,q}R_l\mathcal {B}_{\mathfrak {m}^{res,res}}(f_1,f_2)\right\Vert _{L^2}&\lesssim 2^m2^{k+q_{1}}(\lambda _1K^{-1})^{\frac{1}{2}}2^{\frac{\min \{k_1+q_1,k_2+q_2\}}{2}}2^{-l_1}\left\Vert f_1\right\Vert _X2^{-l_2}\left\Vert f_2\right\Vert _X\\&\lesssim 2^{(1-2\beta )m}2^{k+\frac{3}{2}q_1}2^{\frac{\min \{k_1,k_2\}}{2}}2^{\frac{\min \{k_1+q_1,k_2+q_2\}}{2}}2^{-l_1-l_2}\varepsilon ^2. \end{aligned} \end{aligned}$$

Now if $2^{q_1}<2^{-(1-\delta )m}$ the claim follows directly. If $2^{k_1-k_2-q_2}>2^{(1-\delta )m}$, then there holds $2^{\frac{k_2+q_2}{2}}\lesssim 2^{-\frac{1}{2}(1-\delta )m+\frac{k_1}{2}}$ and we can continue the estimate in (8.20) to obtain an admissible bound

8.21 $$\begin{aligned} \begin{aligned} \left\Vert P_{k,p,q}R_l\mathcal {B}_{\mathfrak {m}^{res,res}}(f_1,f_2)\right\Vert _{L^2}&\lesssim 2^{(1-2\beta )m}2^{\frac{3}{2}k_{\max }+\frac{3}{2}q_{1}}2^{\frac{k_2+q_2}{2}}2^{-l_1}\left\Vert f_1\right\Vert _X\left\Vert f_2\right\Vert _X\\&\lesssim 2^{(-\frac{1}{2}-2\beta +3\delta )m}\varepsilon ^2. \end{aligned} \end{aligned}$$

Similarly, if $2^{k_1+k_2-k_{\min }-k_{\max }-q_{2}}>2^{(1-\delta )m}$ then $2^{\frac{q_2}{2}}<2^{-\frac{1}{2}(1-\delta )m}$ and the claim follows from (8.21). Lastly, if $2^{2k_2-k_{\min }-k_{\max }-q_{1}+l_2}>2^{(1-\delta )m}$, then continuing the estimate (8.20) we obtain

$$\begin{aligned} \left\Vert P_{k,p,q}R_l\mathcal {B}_{\mathfrak {m}^{res,res}}(f_1,f_2)\right\Vert _{L^2}&\lesssim 2^{(1-2\beta )m}2^{k_{\max }+2q_{1}}2^{k_{\min }}2^{-l_1}2^{-l_2}\varepsilon ^2\\&\lesssim 2^{(-1-2\beta +3\delta )m}\varepsilon ^2. \end{aligned}$$

**Case D.1(d.2):** $2^{k_2}\sim 2^{k_1}$**.** Observe that $q_1\sim q_2\sim q_{\max }$ was treated at the beginning of **Case D.1(d)**. Thus we may assume $q_2< q_1-100$. The following bound holds for the resonant bilinear term:

$$\begin{aligned}&\left\| P_{k,p,q}R_l\mathcal {B}_{\mathfrak {m}^{res}}(f_1,f_2)\right\| _{L^2}\\  &\qquad \lesssim 2^m2^{k+q_{1}}\Big [\left\| I_{k_1,p_1}(f_1)\right\| _{L^\infty }\left\| f_2\right\| _{L^2}\\  &\qquad \qquad +\min \big \{\left\lvert S\right\rvert \left\| II_{k_1,p_1}(f_1)\right\| _{L^2}\left\| f_2\right\| _{L^2},\left\| II_{k_1,p_1}(f_1)\right\| _{L^2}\left\| e^{it\Lambda }f_2\right\| _{L^\infty }\big \}\Big ]. \end{aligned}$$

By considering each possible maximum on the left-hand side of (8.17) with $k_{\min }\sim k$ and $q_2\ll q_1$ we observe that we have two possibilities. Either $2^{-l_2}<2^{-(1-\delta )m}2^{-q_1-k+k_2}$ and we use (8.13) on $f_1$, the set size estimate with $\left\lvert S\right\rvert \lesssim 2^{\frac{k+k_1+q_1}{2}}$ and the $X-$norm on $f_2$ to obtain an admissible bound

$$\begin{aligned}&\left\Vert P_{k,p,q}R_l\mathcal {B}_{\mathfrak {m}^{res}}(f_1,f_2)\right\Vert _{L^2} \\&\quad \lesssim 2^{m}2^{k+q_1}[2^{(-1+\delta )m} 2^{-l_2}2^{-4k_2^+}+2^{\frac{k+k_1+q_1}{2}}2^{(-\frac{1}{2}-\frac{\beta }{2})m}2^{-(1+\beta )l_2}2^{-3k_2^+}]\varepsilon \left\Vert f_2\right\Vert _X\\&\quad \lesssim (2^{(-1+2\delta )m}+2^{(-\frac{1}{2}-\frac{3}{2}\beta +2\delta )m})\varepsilon ^2\\&\quad \lesssim 2^{(-\frac{1}{2}-\frac{3}{2}\beta +2\delta )m}\varepsilon ^2. \end{aligned}$$

Else there holds $2^{k+q_2}<2^{-(1-\delta )m+k_2}$. Recall that in this case we also have $2^{-l_1}<2^{-(1-\delta )m}2^{k_2-k-q_1}$. First of all, observe that if $-k<-4\beta m$ we obtain the claim using the set-size estimate Lemma 5.15 and the *X*-norm on $f_1$:

$$\begin{aligned} \left\| P_{k,p,q}R_l\mathcal {B}_{\mathfrak {m}^{res}}(f_1,f_2)\right\| _{L^2}&\lesssim 2^m2^{k+q_{1}}2^{\frac{k+k_2+q_2}{2}}\left\| f_1\right\| _{L^2}\left\| f_2\right\| _{L^2} \\  &\lesssim 2^m2^{k+q_{1}}2^{k_2+\frac{q_2}{2}}2^{-l_1}\varepsilon ^2\\  &\lesssim 2^{(-\frac{1}{2}-2\beta )m}\varepsilon ^2. \end{aligned}$$

Otherwise, if $k<4\beta m$ we do a further splitting $\mathfrak {m}^{res}=\mathfrak {m}^{res,nr}+\mathfrak {m}^{res,res}$ at the scale ${\lambda _2}=2^{-4\beta m}2^{k}\lambda <\lambda$. We bound each term in the non-resonant part (5.12) as in **Case D.1(b)** where $|\mathfrak {m}^{res,nr}\Phi ^{-1}|\lesssim \Vert \mathfrak {m}^{res,nr}\Vert _W \Vert \Phi ^{-1}\Vert _W\lesssim 2^{4\beta m}$ by Lemma A.3. For the boundary term, using Lemma 5.17(1) with $\lambda _2$ and (8.13), we obtain:

$$\begin{aligned}&\left\Vert P_{k,p,q}R_l\mathcal {Q}_{\mathfrak {m}^{res,nr}\Phi ^{-1}}(f_1,f_2)\right\Vert _{L^2} \\&\quad \lesssim 2^{4\beta m}[\left\Vert I_{k_1,p_1}(f_1)\right\Vert _{L^\infty }\left\Vert f_2\right\Vert _{L^2}+\left\Vert II_{k_1,p_1}(f_1)\right\Vert _{L^2}\Vert e^{it\Lambda }f_2\Vert _{L^\infty }]\\&\quad \lesssim 2^{4\beta m}[ 2^{-3k_1^+}2^{(-1+\delta )m}2^{-4k_2^+}+ 2^{-4k_1^+}2^{-(\frac{1}{2}+\frac{\beta }{2})m} 2^{-2k_2^+}2^{(-\frac{1}{2}+\kappa )m}]\varepsilon ^2\\&\quad \lesssim 2^{(-1+4\beta +\delta )m}\varepsilon ^2. \end{aligned}$$

The terms in (5.12) involving the time derivative follow using Lemma 6.1. Indeed,

$$\begin{aligned}&2^{4k^+}2^{(1+\beta +2\delta )m}\left\Vert P_{k,p,q}R_l\mathcal {B}_{\mathfrak {m}^{res,nr}\Phi ^{-1}}(\partial _tf_1,f_2)\right\Vert _{L^2} \\&\quad \lesssim 2^{4k^+}2^{(2+5\beta +2\delta )m}\left\Vert \partial _tf_1\right\Vert _{L^2}\Vert e^{it\Lambda }f_2\Vert _{L^\infty }\\&\quad \lesssim 2^{(\frac{3}{4}+6\beta )m}\varepsilon ^3. \end{aligned}$$

The analogous estimate holds for the term involving $\partial _t f_2$. On the resonant set observe that

$$\begin{aligned} \left\lvert \partial _{\eta _2}\Phi _{\pm }^{\mu \nu }\right\rvert&=\left\lvert \mu \partial _{\eta _2}\Lambda (\xi -\eta )\pm \nu \partial _{\eta _2}\Lambda (\eta )\right\rvert \\  &=\left\lvert \mu \frac{\eta _1\eta _2}{\left\lvert \eta \right\rvert ^3}\mp \nu \frac{(\xi _1-\eta _1)(\xi _2-\eta _2)}{\left\lvert \xi -\eta \right\rvert ^3}\right\rvert \\  &\gtrsim 2^{-k_2+q_1}=:K. \end{aligned}$$

Thus, we can apply Lemma 5.17(4) with $K=2^{-k_2+q_1}$ and $\lambda _2$ to obtain the claim on the $X-$norm as follows

$$\begin{aligned} \left\Vert P_{k,p,q}R_l\mathcal {B}_{\mathfrak {m}^{res,res}}(f_1,f_2)\right\Vert _{L^2}&\lesssim 2^m2^{k+q_{1}}(\lambda _2K^{-1})^{\frac{1}{2}}2^{\frac{\min \{k_1+q_1,k_2+q_2\}}{2}}\left\Vert f_1\right\Vert _{L^2}\left\Vert f_2\right\Vert _{L^2}\\&\lesssim 2^{(1-2\beta )m}2^{\frac{3}{2}k+q_1}2^{k_2+\frac{q_2}{2}}2^{-l_1}2^{-8k_2^+}\varepsilon ^2\\&\lesssim 2^{(-\frac{1}{2}-2\beta +2\delta )m}\varepsilon ^2. \end{aligned}$$

**Case D.3:** $q_{1}<q_{\max }-50$ **and** $q_{2}<q_{\max }-50$**.** In particular, there holds $q_{\max }=q-50> q_1,q_2$ and therefore $\left\lvert \Phi _{\pm }^{\mu \nu }\right\rvert \gtrsim 2^{q-10}$. Thus the splitting described at the beginning of the **Case D** with $\lambda =2^{q-10}$ contains only the non-resonant part $\mathfrak {m}=\mathfrak {m}^{nr}$ which was handled in (8.14)–(8.15). $\square$

## Data Availability

No datasets were generated or analyzed in the preparation of this manuscript.

## Appendix A

### A.1 Control of the Fourier transform in $L^\infty$

#### Proof of Lemma 3.1

By abuse of notation we consider *f* to be already localized, that is $\widehat{f}=\widehat{P_{k,p}f}=\varphi _{k,p}(\xi )\widehat{f}(\xi )$ and consider polar coordinates as in (2.20). Then, with (2.21), we can write for any $(\rho _0,\tau _0) \in {{\,\textrm{supp}\,}}{\varphi _{k,p}\widehat{f}}$ using the fundamental theorem of calculus

A.1 $$\begin{aligned} \begin{aligned} \varphi _{k,p}\widehat{f}(\rho ,\tau )&=\varphi _{k,p}\widehat{f}(\rho ,\tau _0)+\int _{\tau _0}^\tau \partial _\alpha (\varphi _{k,p}\widehat{f})(\rho ,\alpha )d\alpha \\&=\varphi _{k,p}\widehat{f}(\rho _0,\tau _0)+\int _{\rho _0}^\rho \partial _s(\varphi _{k,p}\widehat{f})(s,\tau _0)ds+\int _{\tau _0}^\tau \partial _\alpha (\varphi _{k,p}\widehat{f})(\rho _0,\alpha )d\alpha +\\&\hspace{1cm}+\int _{\rho _0}^\rho \int _{\tau _0}^\tau \partial _s\partial _\alpha (\varphi _{k,p}\widehat{f})(s,\alpha )dsd\alpha . \end{aligned} \end{aligned}$$

Now bound each of these terms on the right-hand side. Let $\overline{\varphi }_{k,p}$ be a function with similar support properties as $\varphi _{k,p}$ (cf. Remark 2.2) and observe that for the last term in (A.1)

$$\begin{aligned}&|\partial _\rho \partial _\tau (\varphi _{k,p}\widehat{f})(s,\alpha )| \\&\quad =|\partial _\rho (\partial _\tau \varphi _{k,p}\widehat{f}+\varphi _{k,p}\partial _\tau \widehat{f})(s,\alpha )|\\&\quad =|\partial _\rho \partial _\tau \varphi _{k,p}\widehat{f}(s,\alpha ) +\partial _\tau \varphi _{k,p}\partial _\rho \widehat{f}(s,\alpha )+\partial _\rho \varphi _{k,p}\partial _\tau \widehat{f}(s,\alpha )+\varphi _{k,p}\partial _\tau \partial _\rho \widehat{f}(s,\alpha )|\\&\quad \lesssim \overline{\varphi }_{k,p}\Big [ 2^{-k-p}|\widehat{f}(s,\alpha )|+2^{-p}|\partial _\rho \widehat{f}(s,\alpha )|+2^{-k}|\partial _\tau \widehat{f}(s,\alpha )|+|\partial _\rho \partial _\tau \widehat{f}(s,\alpha )| \Big ] \end{aligned}$$

Also for any $g\in L^2$, we observe that by the Cauchy-Schwarz inequality there holds

A.2 $$\begin{aligned} \begin{aligned} \left\lvert \int _{\tau _0}^\tau \int _{\rho _0}^{\rho }\overline{\varphi }_{k,p}\widehat{g}(s,\alpha )dsd\alpha \right\rvert ^2&\lesssim \int _{{{\,\textrm{supp}\,}}\overline{\varphi }_{k,p}}s^{-1}dsd\alpha \int _{{{\,\textrm{supp}\,}}\overline{\varphi }_{k,p}}\left\lvert \widehat{g}(s,\alpha )\right\rvert ^2sdsd\alpha \lesssim 2^p \left\Vert g\right\Vert _{L^2}^2. \end{aligned} \end{aligned}$$

With this and the previous observation, we can directly bound the last term in (A.1)

$$\begin{aligned}&\left\lvert \int _{\rho _0}^\rho \int _{\tau _0}^\tau \partial _s\partial _\alpha (\varphi _{k,p}\widehat{f})(s,\alpha )dsd\alpha \right\rvert \\&\quad \lesssim 2^{-k-p}\left\lvert \int _\alpha \int _s\overline{\varphi }_{k,p}\widehat{f} dsd\alpha \right\rvert +2^{-p}\left\lvert \int _\alpha \int _s \overline{\varphi }_{k,p} \partial _s \widehat{f} dsd\alpha \right\rvert \\&\quad \quad +2^{-k}\left\lvert \int _\alpha \int _s \overline{\varphi }_{k,p}\partial _\alpha \widehat{f} ds d\alpha \right\rvert +\left\lvert \int _\alpha \int _s \overline{\varphi }_{k,p} \partial _s\partial _\alpha \widehat{f} dsd\alpha \right\rvert \\&\quad \lesssim 2^{-k-\frac{p}{2}}[\left\Vert f\right\Vert _{L^2}+\left\Vert Sf\right\Vert _{L^2}]+2^{-k+\frac{p}{2}}[\left\Vert Wf\right\Vert _{L^2}+\left\Vert WSf\right\Vert _{L^2}], \end{aligned}$$

where we have used $S=s\partial _s$ and $W=\partial _\alpha$. Similarly, we can average in $\tau$ and obtain

$$\begin{aligned}&\left\lvert \int _{\rho _0}^\rho \partial _s(\varphi _{k,p}\widehat{f})(s,\tau _0)ds\right\rvert \\  &\qquad \qquad =\left\lvert \int _{\rho _0}^\rho \frac{1}{\left\lvert \tau \in {{\,\text {supp}\,}}\varphi _{k,p}\right\rvert } \int _{\tau \in {{\,\text {supp}\,}}\varphi _{k,p}} \partial _s(\varphi _{k,p}\widehat{f})(s,\tau _0)d\tau ds\right\rvert \\  &\qquad \qquad =\left\lvert \int _{\rho _0}^\rho \frac{1}{\left\lvert \tau \in {{\,\text {supp}\,}}\varphi _{k,p}\right\rvert } \int _{\tau }\Big [\int _{\tau _0}^\tau \partial _\alpha \partial _s(\varphi _{k,p}\widehat{f})(s,\alpha )d\alpha +\partial _s(\varphi _{k,p}\widehat{f})(s,\tau )\Big ]d\tau ds \right\rvert \\  &\qquad \qquad \lesssim \left\lvert \int _{\rho _0}^\rho \int _{\tau _0}^\tau \partial _\alpha \partial _s(\varphi _{k,p}\widehat{f})(s,\alpha )d\alpha ds\right\rvert + 2^{-p}\left\lvert \int _s\int _\tau \partial _s(\varphi _{k,p}\widehat{f})(s,\tau )d\tau ds\right\rvert . \end{aligned}$$

The first term in the sum on the right-hand side above is handled in the previous estimates. As for the second term, we can use (A.2) to obtain

$$\begin{aligned} \left\lvert \iint \partial _s(\varphi _{k,p}\widehat{f})(s,\tau )d\tau ds\right\rvert&\lesssim 2^{-k}\left\lvert \iint \overline{\varphi }_{k,p} \widehat{f}(s,\alpha ) d\alpha ds\right\rvert + 2^{-k}\left\lvert \iint \overline{\varphi }_{k,p} \partial _s\widehat{f}(s,\alpha )s d\alpha ds \right\rvert \\&\lesssim 2^{-k+\frac{p}{2}}[\left\Vert f\right\Vert _{L^2}+\left\Vert Sf\right\Vert _{L^2}]. \end{aligned}$$

Altogether, we obtain

$$\begin{aligned} \left\lvert \int _{\rho _0}^\rho \partial _s(\varphi _{k,p}\widehat{f})(s,\tau _0)ds\right\rvert \lesssim 2^{-k-\frac{p}{2}}[\left\Vert f\right\Vert _{L^2}+\left\Vert Sf\right\Vert _{L^2}]+2^{-k+\frac{p}{2}}[\left\Vert Wf\right\Vert _{L^2}+\left\Vert WSf\right\Vert _{L^2}]. \end{aligned}$$

Similarly, we estimate the remaining integral in (A.1):

$$\begin{aligned}&\left\lvert \int _{\tau _0}^\tau \partial _\alpha (\varphi _{k,p}\widehat{f})(\rho _0,\alpha )d\alpha \right\rvert \\  &\qquad \qquad =\left\lvert \int _{\tau _0}^\tau \frac{1}{\left\lvert \rho \in {{\,\text {supp}\,}}\varphi _{k,p}\right\rvert }\int _{\rho \in {{\,\text {supp}\,}}\varphi _{k,p}}\partial _\alpha (\varphi _{k,p}\widehat{f})(\rho _0,\alpha )d\rho d\alpha \right\rvert \\  &\qquad \qquad =\left\lvert \int _{\tau _0}^\tau \frac{1}{\left\lvert \rho \in {{\,\text {supp}\,}}\varphi _{k,p}\right\rvert }\int _\rho \Big [ \int _{\rho _0}^{\rho }\partial _s\partial _\alpha (\varphi _{k,p}\widehat{f})(s,\alpha )ds +\partial _\alpha (\varphi _{k,p}\widehat{f})(\rho ,\alpha ) \Big ] d\rho d\alpha \right\rvert \\  &\qquad \qquad \lesssim \left\lvert \int _{\rho _0}^\rho \int _{\tau _0}^\tau \partial _\alpha \partial _s(\varphi _{k,p}\widehat{f})(s,\alpha )d\alpha ds\right\rvert +2^{-k}\left\lvert \int _\alpha \int _\rho \partial _\alpha (\varphi _{k,p}\widehat{f})(\rho ,\alpha )d\rho d\alpha \right\rvert \\  &\qquad \qquad \lesssim \left\lvert \int _{\rho _0}^\rho \int _{\tau _0}^\tau \partial _\alpha \partial _s(\varphi _{k,p}\widehat{f})(s,\alpha )d\alpha ds\right\rvert + 2^{-k-p}\left\lvert \int _\alpha \int _\rho \overline{\varphi }_{k,p} \widehat{f}(\rho ,\alpha )d\rho d\alpha \right\rvert \\  &\qquad \qquad \qquad +2^{-k}\left\lvert \int _\alpha \int _\rho \overline{\varphi }_{k,p} \partial _\alpha \widehat{f}(\rho ,\alpha )d\rho d\alpha \right\rvert . \end{aligned}$$

Thus, with (A.2), we obtain

$$\begin{aligned} \left\lvert \int _{\tau _0}^\tau \partial _\alpha (\varphi _{k,p}\widehat{f})(\rho _0,\alpha )d\alpha \right\rvert&\lesssim 2^{-k-\frac{p}{2}}[\left\| f\right\| _{L^2}+\left\| Sf\right\| _{L^2}]\\  &\quad +2^{-k+\frac{p}{2}}[\left\| Wf\right\| _{L^2}+\left\| WSf\right\| _{L^2}]. \end{aligned}$$

Finally, we estimate that

$$\begin{aligned}&|\varphi _{k,p}\widehat{f}(\rho _0,\tau _0)| \\  &\quad =\left\lvert \frac{1}{\left\lvert \tau \in {{\,\text {supp}\,}}\varphi _{k,p}\right\rvert } \int _{\tau \in {{\,\text {supp}\,}}\varphi _{k,p}}\varphi _{k,p}\widehat{f}(\rho _0,\tau _0)d\tau \right\rvert \\  &\quad =\frac{1}{\left\lvert \tau \in {{\,\text {supp}\,}}\varphi _{k,p}\right\rvert }\left\lvert \int _\tau \Bigg [ \int _{\tau _0}^\tau \partial _\alpha (\varphi _{k,p}\widehat{f})(\rho _0,\alpha )d\alpha +(\varphi _{k,p}\widehat{f})(\rho _0,\tau ) \Big ]d\tau \right\rvert \\  &\quad \lesssim \left\lvert \int _{\tau _0}^\tau \partial _\tau (\varphi _{k,p}\widehat{f})(\rho _0,\alpha )d\alpha \right\rvert \\  &\quad \quad +\frac{1}{\left\lvert \tau \in {{\,\text {supp}\,}}\varphi _{k,p}\right\rvert } \left\lvert \int _\tau \frac{1}{\left\lvert \rho \in {{\,\text {supp}\,}}\varphi _{k,p}\right\rvert }\Big [\int _{\rho _0}^\rho \partial _s(\varphi _{k,p}\widehat{f})(s,\tau )ds +\varphi _{k,p}\widehat{f}(\rho ,\tau ) \Big ] d\rho d\tau \right\rvert \\  &\quad \lesssim 2^{-k-\frac{p}{2}}[\left\| f\right\| _{L^2}+\left\| Sf\right\| _{L^2}]+2^{-k+\frac{p}{2}}[\left\| Wf\right\| _{L^2}+\left\| WSf\right\| _{L^2}] +\\  &\quad \quad + 2^{-k-p}\left\lvert \int _\rho \int _\tau \varphi _{k,p}\widehat{f}(\rho ,\tau )d\tau d\rho \right\rvert \\  &\quad \lesssim 2^{-k-\frac{p}{2}}[\left\| f\right\| _{L^2}+\left\| Sf\right\| _{L^2}]+2^{-k+\frac{p}{2}}[\left\| Wf\right\| _{L^2}+\left\| WSf\right\| _{L^2}] \end{aligned}$$

Recalling the definition of the $B-$norm (2.22), we obtain, from (A.1),

$$\begin{aligned} \Vert \widehat{P_{k,p}f}\Vert _{L^\infty }&\lesssim 2^{-k-\frac{p}{2}}[\left\| P_{k,p}f\right\| _{L^2}+\left\| SP_{k,p}f\right\| _{L^2}]+2^{-k+\frac{p}{2}}[\left\| WP_{k,p}f\right\| _{L^2} \\  &\quad +\left\| WSP_{k,p}f\right\| _{L^2}]\\  &\lesssim 2^{-4k^+}2^{\frac{k^-}{2}}2^{-k}[\left\| f\right\| _B+\left\| Sf\right\| _B]\\  &\quad +2^{-k+\frac{p}{2}} \sum _{l\in \mathbb {Z}^+,l+p\ge 0}[\left\| WP_{k,p}R_lf\right\| _{L^2}+\left\| WSP_{k,p}R_lf\right\| _{L^2}]. \end{aligned}$$

The claim follows by noting that with Proposition 2.3 and the definition of the $X-$norm (2.23)

$$\begin{aligned} 2^{\frac{p}{2}}\sum _{l\in \mathbb {Z}^+,l+p\ge 0}\left\Vert WP_{k,p}R_lf\right\Vert _{L^2}&\lesssim \sum _{l\in \mathbb {Z}^+,l+p\ge 0}2^{l}2^{\frac{p}{2}}\left\Vert P_{k,p}R_lf\right\Vert _{L^2}\\&\lesssim \sum _{l\in \mathbb {Z}^+,l+p\ge 0} 2^{l}2^{\frac{p}{2}}2^{-3k^+}2^{-(1+\beta )l}2^{-\beta p}2^{-\frac{p}{2}}\left\Vert f\right\Vert _X\\&\lesssim 2^{-4k^+} \sum _{l\in \mathbb {Z}^+,l+p\ge 0} 2^{-\beta (l+p)}\left\Vert f\right\Vert _X\lesssim 2^{-4k^+} \left\Vert f\right\Vert _X. \end{aligned}$$

$$\square $$

### A.2 Symbol bounds

#### Lemma A.1

Consider a multiplier $\mathfrak {m}\in L^{1}_{loc}(\mathbb {R}^2\times \mathbb {R}^2)$ and $\chi$ as in (2.17). Then for bilinear expressions as in (5.8)

$$\begin{aligned}&\left\Vert \mathcal {Q}_{\mathfrak {m}\chi }(f,g)\right\Vert _{L^r}\lesssim \Vert \mathfrak {m}\Vert _{W}\left\Vert f\right\Vert _{L^{p}}\left\Vert g\right\Vert _{L^{q}},  &   \frac{1}{r}=\frac{1}{p}+\frac{1}{q}, \end{aligned}$$

and where $$\Vert \mathfrak {m}\Vert _W:=\sup _{k,p,k_i,p_i, i=1,2}\left\Vert \mathcal {F}(\mathfrak {m}\chi )\right\Vert _{L^1(\mathbb {R}^2\times \mathbb {R}^2)}$$.

The proof of Lemma A.1 follows by the Minkowski and Hölder inequalities. Moreover, note the following property: letting $\mathfrak {m}_1$, $\mathfrak {m}_2\in L^{1}_{loc}(\mathbb {R}^2\times \mathbb {R}^2)$, then

A.3 $$\begin{aligned} \left\Vert \mathfrak {m}_1\cdot \mathfrak {m}_2\right\Vert _{W}\lesssim \left\Vert \mathfrak {m}_1\right\Vert _W\left\Vert \mathfrak {m}_2\right\Vert _W. \end{aligned}$$

This follows from the convolution property of the Fourier transform. Next we prove that we have the same bound for $\left\Vert \mathfrak {m}\right\Vert _W$ as in Lemma 5.5.

#### Lemma A.2

For $$\mathfrak {m}\in \{\mathfrak {m}_0,\mathfrak {m}_\pm ^{\mu \nu }\}$$

$$\begin{aligned} \left\Vert \mathfrak {m}\right\Vert _W\lesssim 2^{k+p_{\max }}. \end{aligned}$$

This follows by establishing an $L^1$-bound on $\mathcal {F}(\mathfrak {m}\chi )=\iint e^{-ix\cdot \xi }e^{-iy\cdot \eta }\mathfrak {m}(\xi ,\eta )\chi (\xi ,\eta )d\xi d\eta$ using a suitable change of variables and integration by parts, see [25, Lemma A.13] for an analogous computation in three dimensions.

In Sect. 5.7 and in particular in Lemma 5.17(1), we want to bound $\mathfrak {m}^{nr}\Phi ^{-1}\chi$. By the algebra property (A.3) and Lemma A.2 it suffices to establish the following lemma, whose proof is a straightforward adaptation to two dimensions of the proof of [25, Lemma A.15].

#### Lemma A.3

Let $\Phi \in \left\{ \Phi _{\pm }^{\mu \nu } \mid \mu ,\nu \in \{+,-\} \right\}$ and $\chi$ as in (2.17). Then

$$\begin{aligned} \left\Vert \phi ^{-1}(1-\psi (\lambda ^{-1}\Phi ))\chi \right\Vert _W\lesssim \lambda ^{-1}. \end{aligned}$$

## Footnotes

1 In particular, such configurations are *spectrally stable*, as the eigenvalues of the linearized operator of (1.3) around zero are purely imaginary [24].

2 Observe that if $(u,\rho )$ solve (1.3) on a time interval [0, *T*], then for $\lambda >0$, the rescaled functions $(\lambda u(\lambda t,x), \lambda \rho (\lambda t,x))$ solve (1.3) on $[0,\lambda ^{-1}T]$ with an additional “strength of gravity” constant $\lambda$ in front of the linear terms.

3 The relevance of including at least two copies of *S* in this norm in order to obtain the linear decay can be seen in **Case B** in the proof of Proposition 3.2, for example.
